# Allylic hydroxylation of enones useful for the functionalization of relevant drugs and natural products

**DOI:** 10.1038/s41467-023-38154-9

**Published:** 2023-04-26

**Authors:** Cheng-Yu Zheng, Jian-Min Yue

**Affiliations:** 1 State Key Laboratory of Drug Research, Shanghai Institute of Materia Medica, Chinese Academy of Sciences, 555 Zuchongzhi Road, Shanghai, 201203 China; 2 University of Chinese Academy of Sciences, No.19A Yuquan Road, Beijing, 100049 China; 3 Research Units of Discovery of New Drug Lead Molecules, Chinese Academy of Medical Sciences, Shanghai, 201203 China

**Keywords:** Photocatalysis, Synthetic chemistry methodology

## Abstract

Enones are privileged structural motifs in bioactive natural products and pharmaceuticals, but the γ-hydroxylation of enones is challenging. Here we show a mild and efficient method for the direct C(sp^3^)–H hydroxylation of enones via visible-light-induced hydrogen-atom transfer (HAT), which facilitates γ-hydroxylation of primary, secondary, and tertiary C–H bonds of different enones without involving metal and peroxide. The mechanism study shows that Na_2_-eosin Y serves as both the photocatalyst and the source of catalytic bromine radical species in the HAT-based catalytic cycle, and finally sacrifices itself completely by oxidative degradation to produce bromine radical and a major product phthalic anhydride in an environmentally friendly way. This scalable method was demonstrated by plenty of substrates (41 examples) including 10 clinical drugs and 15 natural products to be useful for the late-stage functionalization of enone-containing compounds, and, in particular, has potential application in industry for large-scale production.

## Abstract (web summary)

Enones are privileged structural motifs in bioactive natural products and pharmaceuticals, but the γ-hydroxylation of enones is challenging. Here, the authors report on a mild and efficient method for the direct C(sp3)–H hydroxylation of enones via visible-light-induced hydrogen-atom transfer, without the need for a metal and peroxide.

## Introduction

Methods for site-specific hydroxylation of C(sp^3^)–H bond have provided a versatile toolbox for synthetic chemists to access valuable products^1^ and conduct the late-stage functionalization of drugs and drug-like compounds^2,3^. Significant advances in this area have been achieved chemically by employing catalytic methods based on metal complex^4–7^ and electrochemical approaches^8^. The enzymatic hydroxylation has been demonstrated as an alternative way for the direct conversion of C(sp^3^)–H to C–O bond^9,10^.

**Fig. 1:**
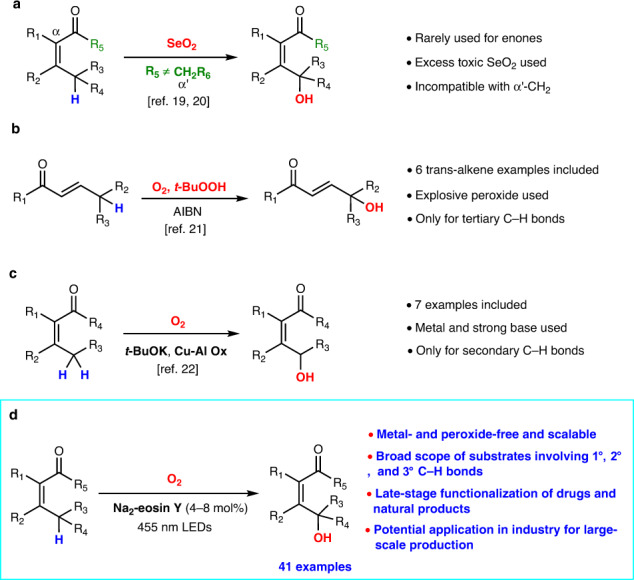
Methods for hydroxylation of γ-C–H bonds of enones. **a** Riley oxidation of enones. **b** γ-Hydroxylation of tertiary C–H bonds of enones. **c** γ-Hydroxylation of secondary C–H bonds of enones. **d** This work: γ-Hydroxylation of primary, secondary and tertiary C–H bonds of enones. AIBN azodiisobutyronitrile. Cu-Al Ox copper–aluminum mixed oxide.

Enone constituting a privileged structural motif for many bioactive natural products and pharmaceuticals is one of the most fascinating and useful organic moieties in organic chemistry. The site-specific introduction of a hydroxy group to enone will provide a profound impact on its physical and biological properties^11^. However, the γ-hydroxylation of enones has proved to be challenging, which was often achieved via a much detour strategy^12–14^. By contrast, the methods for the γ-ketonization of secondary γ-C(sp^3^)–H bonds of enones have been developed and widely applied in organic synthesis^15–18^. The direct γ-hydroxylation of enones by using Riley oxidation was found to be inefficient and incompatible with the presence of α‘-CH_2_ in the enone molecules (Fig. 1a)^19,20^. In 1988, Watt and co-workers developed the radical hydroxylation of tertiary γ-C(sp^3^)–H bonds of enones by using an excess of *t*-BuOOH in the presence of AIBN and oxygen, in which six substrates were tested, and most of them gave low yields (Fig. 1b)^21^. In 2013, Guerra and co-workers reported the hydroxylation of secondary γ-C(sp^3^)–H bonds of enones by using stoichiometric *t*-BuOK and O_2_ in the presence of a copper–aluminum mixed oxide, and seven substrates were evaluated (Fig. 1c)^22^. The aforementioned strategies for the γ-hydroxylation of enones were found to suffer from the limited scope of substrates and the heavy use of metals, peroxides, and strong bases. Hence, the development of more reliable and practical methods with broad scope of substrates for the direct γ-hydroxylation of enones is in great demand for the community of organic chemistry. Auspiciously, the main motivation from our recent synthesis of a natural product involving such a key issue has led to the achievement of a mild and effective method for the direct C(sp^3^)–H hydroxylation of enones for the primary, secondary, and tertiary C–H bonds of diverse enones without involving metal and peroxide (Fig. 1d).

Inspired by the biosynthetic hydroxylation catalyzed by P450 enzymes in the late-stage modification of natural molecules^23^ and the recent advances in C(sp^3^)–H functionalization adopting visible-light-induced HAT strategies^24–29^, our effort was thus made to develop a mild and efficient method for the direct C(sp^3^)–H hydroxylation of enones, in which we envisaged that: (1) a suitable HAT catalyst is required to abstract the hydrogen atom of γ-C(sp^3^)–H bond of an enone to generate a C-centered radical that will be further transformed to a peroxyl radical by trapping O_2_; (2) the resulting peroxyl radical then becomes a hydroperoxide by taking a H-atom from the HAT catalyst; (3) the hydroperoxide is finally converted into the desired alcohol product through a reduction procedure.

Herein, we report the realized visible-light-induced HAT approach to direct γ-C(sp^3^)–H hydroxylation of enones under meta- and peroxide-free conditions at ambient temperature and atmosphere, which has been demonstrated to be compatible for the late-stage functionalization of drugs and natural products, and to be particularly useful in industry for large-scale production.

## Results and discussion

### Reaction development

In order to develop a practical and reliable approach for the late-stage functionalization of drugs and natural products, spironolactone (**1a**), an antihypertensive drug, was taken as a model substrate under various conditions, as summarized in Supplementary Tables 1–3. After extensive investigation of various photocatalysts, solvents, and light sources, the combination of spironolactone (**1a**) and Na_2_-eosin Y (7 mol%) in MeCN (10 mL) under irradiation of 50 W 455 nm LEDs and an oxygen atmosphere for 12 h at room temperature, followed by treatment with thiourea (1.2 equiv.) and MeOH (10 mL), was found to provide the best results (Table 1). Under this optimal condition, **2a** and **3a** were isolated in 90% combined yield in the ratio of 20:1. With the utilization of other xanthene dyes^27^, including K_2_-eosin Y, eosin Y (neutral), eosin B and phloxine B, the reaction also occurred but with less efficiency. Notably, the employment of TBADT as the catalyst resulted in decomposition of **1a**, and only a trace amount of product was detected in the case of AQ or PT as the catalyst (Table 1, entries 1–9). Acetonitrile was found to be the optimal solvent for the reaction and the applications of acetone and EtOAc also gave good yields (Table 1, entries 10–13). The light source screening showed that the reactions were highly sensitive to the wavelengths of light, in which the 455 nm LED was identified as the optimal light source (Table 1, entries 14–18). Our control experiments indicated that HAT catalyst, O_2_ and 455 nm LED are all the critical reaction elements (Table 1, entries 19–21).

**Table 1:** Optimization of reaction conditions for the direct C(sp^3^)-H hydroxylation^a^


Entry	Deviation from the standard conditions	Yield (2a + 3a, %)^b^	Ratio (2a:3a)^c^
1	None	90	20:1
2	Eosin Y (neutral)	71	5.6:1
3	K_2_-eosin Y	84	12.5:1
4	Eosin B	68	20:1
5	Phloxine B	76	20:1
6	Erythrosine	n.d.	–
7^d^	AQ	trace	–
8	PT	trace	–
9^e^	TBADT	decomposed	–
10	Acetone	89	14.3:1
11	DCE	trace	–
12	MeOH	n.d.	–
13	EtOAc	86	3.6:1
14	420 nm LEDs	71	6.3:1
15	400 nm LEDs	20	1.2:1
16	385 nm LEDs	9	1:1.3
17	535 nm LEDs	n.d.	–
18	65 W CFL	n.d.	–
19	No catalyst	n.d.	–
20	No O_2_	n.d.	–
21	No light	n.d.	–

^a^Standard conditions: **1a** (0.20 mmol), Na_2_-eosin Y (7 mol%), O_2_ balloon, and MeCN (10 mL) at r.t. under the irradiation of 50 W 455 nm LEDs for 12 h; then thiourea (0.24 mmol, 1.2 equiv.) and MeOH (10 mL) were added and stirred for 4 h.

^b^Isolated yield.

^c^Determined by NMR analysis of the mixture of **2a** and **3a** after flash column chromatography.

^d^420 nm LEDs were used.

^e^365 nm LEDs were used.

*AQ* anthraquinone, *PT* 5,7,12,14-pentacenetetrone, *TBADT* tetrabutylammonium decatungstate, *DCE* 1,2-dichloroethane, *n.d.* not detected, *CFL* compact fluorescent lamp.

### Evaluation of substrate scope

**Fig. 2:**
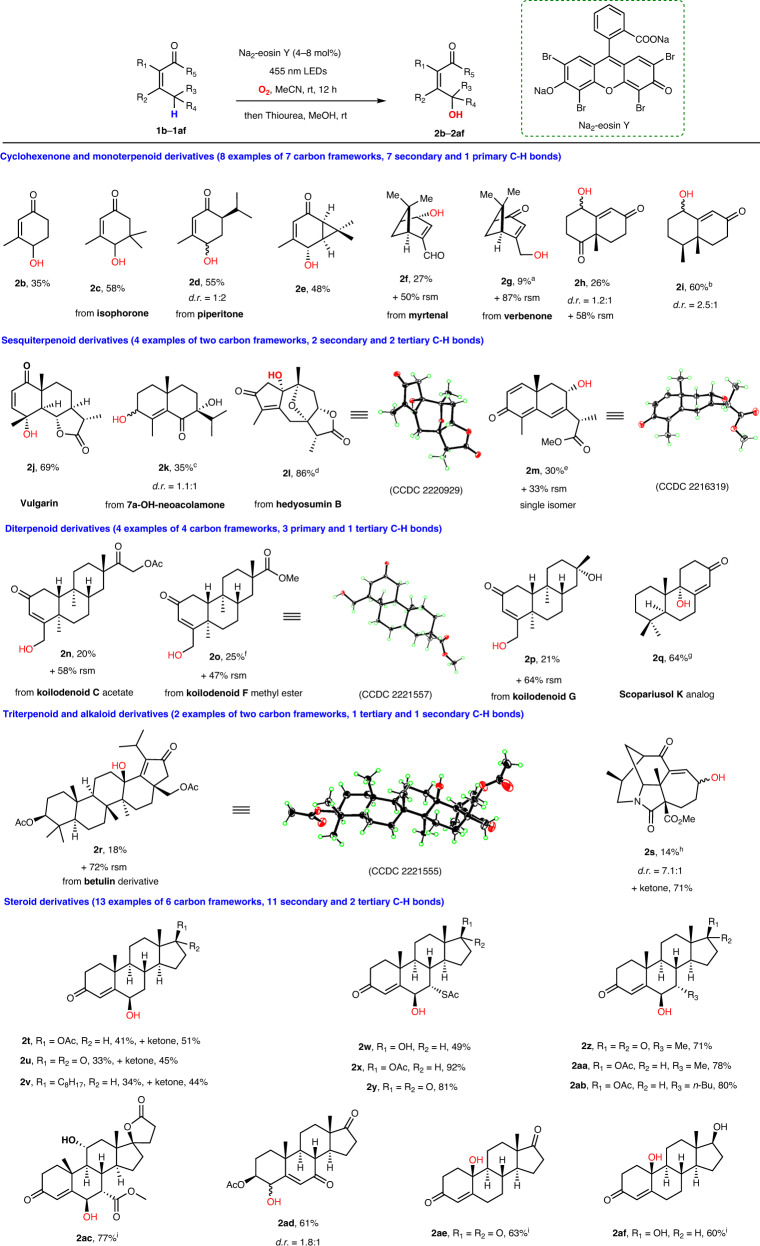
Scope of the primary, secondary, and tertiary C(sp^3^)–H hydroxylation of varied structural types of enones enabled by visible-light-induced hydrogen-atom transfer. Reaction conditions: enone (0.20 mmol), Na_2_-eosin Y (7 mol%), O_2_ balloon, and MeCN (10 mL) at r.t. under the irradiation of 50 W 455 nm LEDs for 12 h; then thiourea (0.24 mmol, 1.2 equiv.) and MeOH (10 mL) were added and stirred for 4 h unless otherwise noted; Isolated yields are reported. ^a^Irradiation time: 15 h; Acetone (10 mL) as solvent. ^b^On 0.18 mmol scale; Irradiation time: 5 h. ^c^On 0.08 mmol scale; 4 mol% Na_2_-eosin Y. ^d^8 mol% Na_2_-eosin Y; irradiation time:15 h. ^e^On 2.04 mmol scale; MeCN (80 mL); MeOH (30 mL). ^f^On 0.12 mmol scale. ^g^On 0.085 mmol scale, 6 mol% Na_2_-eosin Y, Irradiation time: 9 h. ^h^On 0.063 mmol scale; 5 mol% Na_2_-eosin Y; Irradiation time: 8 h. ^i^4 mol% Na_2_-eosin Y. rt room temperature. rsm recovered starting material.

With the optimal conditions in hand, we subjected varied structural types of enones including cyclohexenones, monoterpenoids, sesquiterpenoids, diterpenoids, triterpenoids, steroids and an alkaloid to the reaction procedures (Fig. 2), which showed an excellent site-selectivity and a high level of functional group tolerance. We first examined the γ-hydroxylation of a scope of cyclohexanone- and monoterpenoid-derived substrates, and all the examples gave synthetically useful yields. Notably, the one-step effective synthesis of compound **2b** from the readily available 3-methyl-2-cyclohexenone has made a robust demonstration for our approach as compared to the 4-steps preparation of **2b** from 3-methylanisole in a recent total synthesis of longeracinphyllin A by Li group^12^. Two natural products isophorone (**1c**) and piperitone (**1d**), the main components of essential oils, were smoothly hydroxylated to afford **2c** and **2d** in good yields. The 3-carene-derived enone (**1e)** was successfully oxidized to give **2e** in 48% yield and no radical rearrangement product was detected^30^. The oxidation-sensitive myrtenal was selectively hydroxylated to afford the corresponding alcohol **2f**. The γ-hydroxyverbenone **2g**, previously prepared in two steps using vinylogous Rubottom oxidation^14^, was prepared in one step from verbenone (**1g**) in 9% isolated yield along with 87% recovered starting material. Wieland-Miescher ketone **1h** was hydroxylated to afford **2h** in synthetically useful yield (26%), along with the recovered **1h** (58%), and especially its analog **1i** underwent excellent γ-hydroxylation to afford **2i** in 60% yield. The preparation of **2i** by this method is noteworthy because it was made previously by a detour strategy as the key intermediate in the course of the total synthesis of eremophilane-type sesquiterpenoids by Liu group after the failure of a variety of direct oxidation approaches to hydroxylate **1i** by using *t*-BuOOH/AIBN, Ph(SeO)_2_O, KOH/O_2_, Pd(TFA)_2_/BQ, White catalyst, and SeO_2_^13^. Sesquiterpenoids and diterpenoids are structurally interesting and biologically significant natural products^31,32^. An natural eudesmane-type sesquiterpenoid, vulgarin (**2j**) was prepared as a single diastereomer in 69% yield. The other natural eudesmane-type sesquiterpenoid, 7α-hydroxyneoacolamone underwent the γ-hydroxylation to afford **2k** in 35% yield. Hedyosumin B, a guaiane-type sesquiterpenoid, was successful oxidized to give **2l** in 86% yield and the absolute configuration of **2l** was determined by single crystal X-ray diffraction (CCDC 2220929). Notably, our protocol can also be used for the vinylogous γ-hydroxylation of **1m** to afford **2m** in 30% yield as a single isomer. The absolute configuration of **2m** was assigned by single crystal X-ray diffraction (CCDC 2216319). Three dolabrane-type diterpenoids koilodenoid C (**1n**, as its acetate ester), koilodenoid F (**1o**, as its methyl ester) and koilodenoid G (**1p**)^33^ were successfully hydroxylated to give the corresponding primary alcohols **2n**, **2o** and **2p** in 20%, 25% and 21% yields, along with 58%, 47% and 64% recovered starting materials, respectively. The absolute configuration of **2o** was determined by single crystal X-ray diffraction (CCDC 2221557). A synthetic podocarpane-type diterpenoid **1q** underwent smooth hydroxylation to provide **2q** in 64% yield. The hydroxylation of triterpenoids could improve the poor properties such as the water solubility and pharmacokinetics^34^. Betulin derivative **1r**, an important triterpenoid intermediate in the synthesis of a HIV maturation inhibitor^35^ was oxidized to afford a single product **2r** in 18% yield along with 72% recovered starting material. The absolute configuration of **2r** was confirmed by single crystal X-ray diffraction (CCDC 2221555). Notably, an alkaloid derivative **1s**, which served as the key intermediate in the total synthesis of longeracinphyllin A by Li group^12^, was oxidized to give the required secondary alcohol **2s** in 14% yield and the corresponding ketone in 71% yield. Encouraged by above results, we started to explore the hydroxylation of steroids that are privileged structures in drug discovery. A number of steroids were then studied and the synthetically useful yields were obtained in all the tested cases. For the steroids with a CH_2_−7 group, this protocol afforded significant quantities of ketone rather than secondary alcohol products (**2t**–**2v**). By contrast, the steroids bearing CHR-7 (R = SAc, Me, *n*-Bu, CO_2_Me) as the case of spironolactone were oxidized smoothly to afford selectively secondary alcohol products in good yields (**2w**–**2ac**). It is likely that the presence of a substituent at C7 will stabilize the hydroperoxide intermediate and finally result in the formation of alcohol products^36^ (see Supplementary Fig. 8 for details). Similarly, the selective hydroxylation at C4 in enone steroid **1ad** afforded the secondary alcohol product **2ad** in 61% yield due to the presence of an acetoxyl group at C3. While, oxidation of 19-norandrostenedione(**1ae**) and nandrolone(**1af**) selectively produced the C10−OH products **2ae** and **2af** in 63% and 60% yield, respectively.

The product yields and the regioselectivity of the aforementioned reaction examples indicated that (1) the secondary γ-C–H bonds have priority to the primary γ-C–H bonds (as illustrated by the cases of **1b**−**1e** and **1k**), and the tertiary γ-C–H bonds are the most reactive ones and take precedence over the two formers (see the cases of **1l,** **1q,** **1ae** and **1af**); (2) the presence of a neighboring substituent at the secondary γ-C–H bonds in the enones will likely stabilize the hydroperoxide intermediate and result in the selective formation of alcohol products (see the cases of **1w**–**1ac**); (3) although it is a tertiary γ-C–H bond, the γ-hydroxylation will not take place at the bridgehead position, as showed in the case of **1g**.

**Fig. 3:**
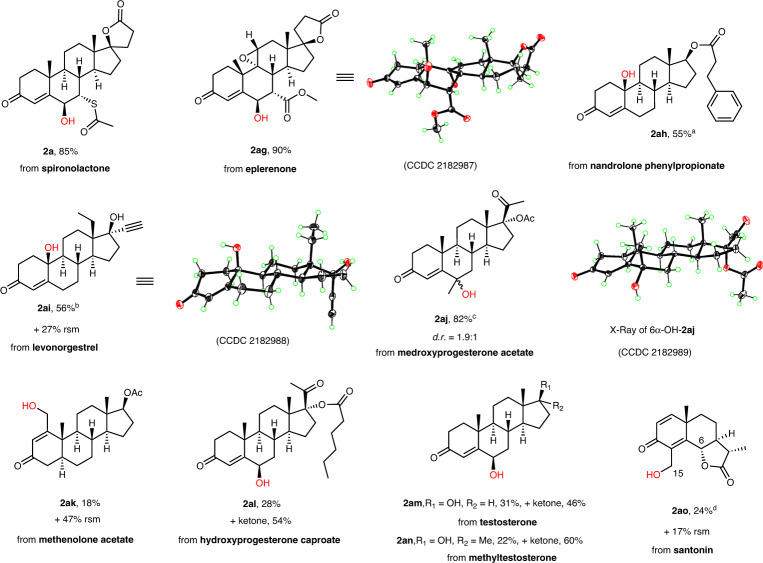
Modification of clinical drugs (10 examples of 9 carbon frameworks, which were not included in Fig. 2). Reaction conditions: enone (0.20 mmol), Na_2_-eosin Y (7 mol%), O_2_ balloon, and MeCN (10 mL) at r.t. under the irradiation of 50 W 455 nm LEDs for 12 h; then thiourea (0.24 mmol, 1.2 equiv.) and MeOH (10 mL) were added and stirred for 4 h unless otherwise noted; Isolated yields are reported. ^a^4 mol% Na_2_-eosin Y. ^b^Acetone (10 mL) as solvent. ^c^On 0.50 mmol scale. ^d^On 1.00 mmol scale; MeCN (25 mL); MeOH (20 mL); Thiourea (1.5 equiv.). rsm = recovered starting material.

We then explored the ability of this method with the hopes to provide a general platform for directly installing hydroxy groups into the hydrocarbon cores of complex drug molecules (Fig. 3), which would likely improve the biological and physicochemical properties of the clinical drugs from the views of medicinal chemistry. The reaction of antihypertensive drug eplerenone, which contains an α-to-O C–H bond, provided **2ag** in 90% yield with remarkably high site- and diastereoselectivity. The absolute configuration of **2ag** was confirmed by single crystal X-ray diffraction (CCDC 2182987). The reaction of nandrolone phenylpropionate that is used to treat breast neoplasms afforded the hydroxylated product **2ah** in 55% yield, indicating that the reaction of the tertiary allylic C–H bonds in preference to both the allylic and benzylic secondary C–H bonds. Levonorgestrel, which is used in contraception and hormone therapy, was successful oxidized with the terminal alkyne moiety intact to afford a single hydroxylation product **2ai** in 56% yield. The structure of **2ai** was verified by single crystal X-ray diffraction (CCDC 2182988). Although the microbial transformation as the only way was reported for the direct hydroxylation of the C10–H bond in the steroid **1ai**, the reaction efficiency was much low^37,38^. Medroxyprogesterone acetate, which helps to regulate ovulation and menstrual periods, underwent smooth hydroxylation to give **2aj** in 82% yield. The reaction of methenolone acetate that is used to treat anemia gave the hydroxylated product **2ak** in 18% yield along with 47% recovered starting material. Three steroid drugs hydroxyprogesterone caproate (**1al**), testosterone (**1am**), and methyltestosterone (**1an**) were also successfully hydroxylated to produce the expected alcohol and ketone products (**2al**, **2am** and **2an**) in useful yields. Notably, santonin, an ascarid repellent, underwent smooth oxidation to give a C-15 hydroxylation product **2ao** in 24% yield and the expected C-6 hydroxylation product was undetected. To the best of our knowledge, the biotransformation is the only way reported to directly hydroxylate the C15–H bond of santonin^39^.

**Fig. 4:**
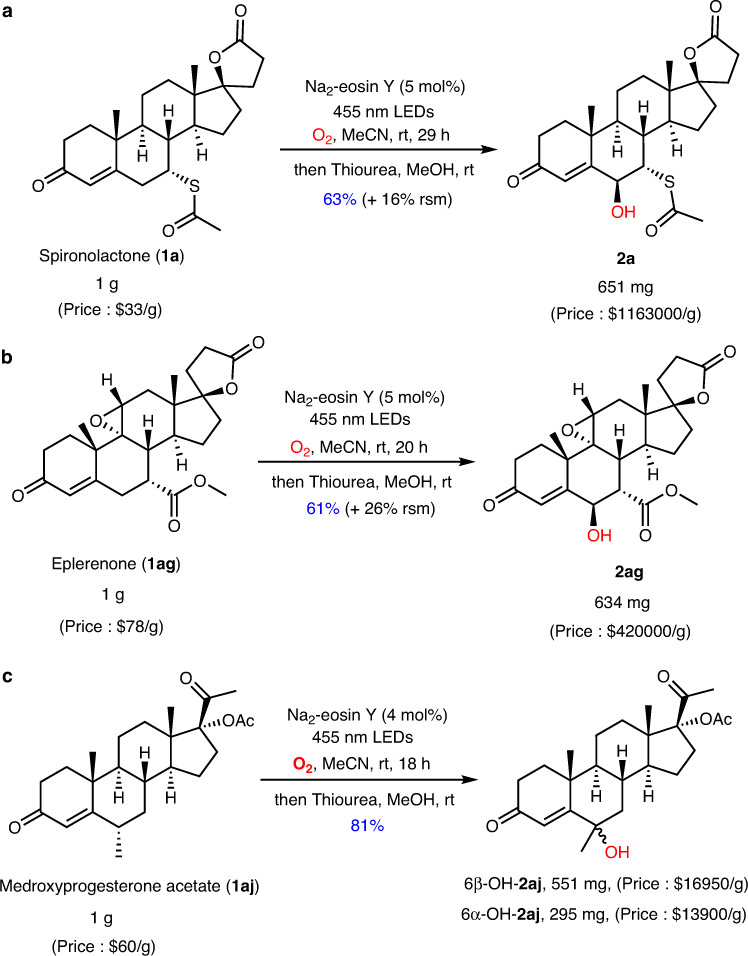
Gram-scale hydroxylation of enone examples in standard condition with slight modifications. **a** Hydroxylation of **1a** on gram scale. **b** Hydroxylation of **1ag** on gram scale. **c** Hydroxylation of **1aj** on gram scale. See the Supplementary Information for details of the prices of reagents. rt room temperature, rsm recovered starting material.

**Fig. 5:**
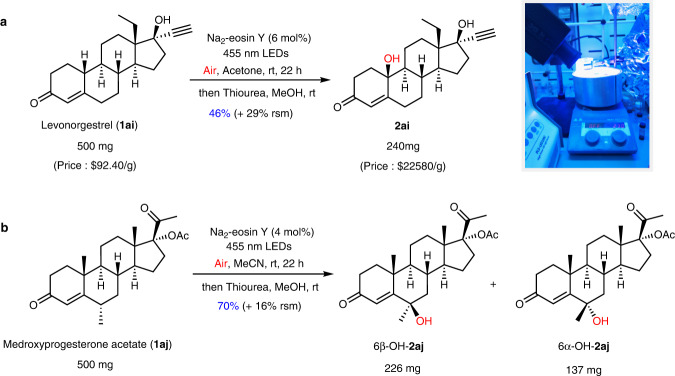
500 mg-scale hydroxylation of enone examples at ambient temperature and atmosphere with air as the oxidant. **a** Hydroxylation of **1ai** on 500 mg scale. **b** Hydroxylation of **1aj** on 500 mg scale. See the Supplementary Information for details of the prices of reagents. rt room temperature, rsm recovered starting material.

**Fig. 6:**
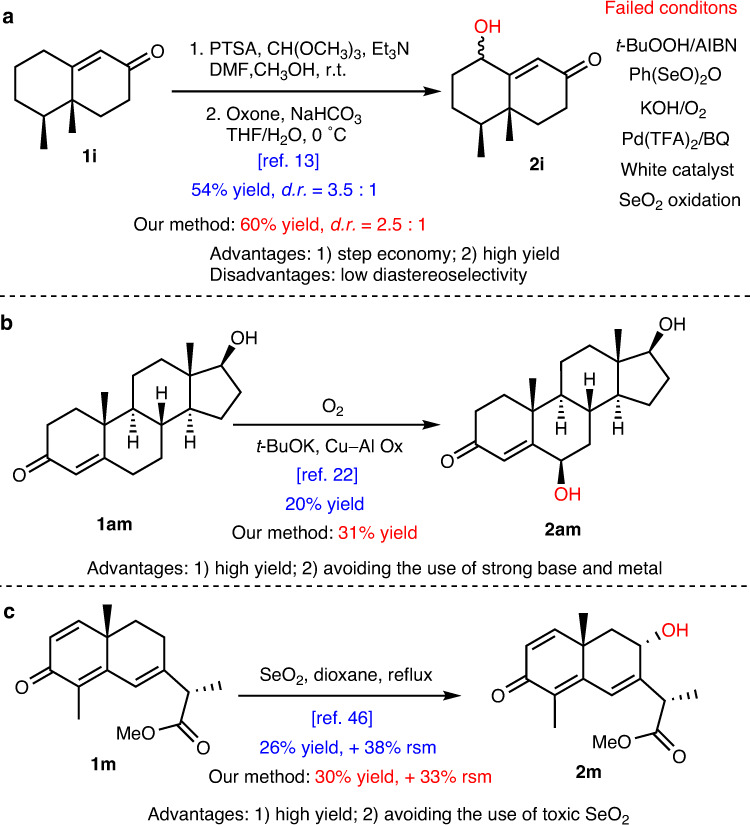
Comparation of our method with the published ones for the hydroxylation of enones. **a** The reported method for hydroxylation of **1i**. **b** The reported method for hydroxylation of **1am**. **c** The reported method for hydroxylation of **1m**^46^. PTSA p-Toluenesulfonic acid. Cu-Al Ox copper–aluminum mixed oxide.

The potential synthetic practicality of this methodology was further demonstrated by the gram-scale syntheses of **2a,** **2ag**, and **2aj** (Fig. 4). The reagent prices of the reaction products are dramatically increased as compared with the original ones used as the starting materials as illustrated in Fig. 4, unambiguously indicating the robust value of this method. Notably, the 500 mg-scale oxidation of levonorgestrel (**1ai**) and medroxyprogesterone acetate (**1aj**) in ambient temperature and atmosphere with air as the oxidant produced the required products **2ai** and **2aj** in 46% and 70% yields, respectively (Fig. 5). Notably, a head-to-head comparison with the published methods for enone hydroxylation was made (Fig. 6), indicating the merits of our method including step economy, mild reaction conditions and easy operation.

### Investigations of mechanism

**Fig. 7:**
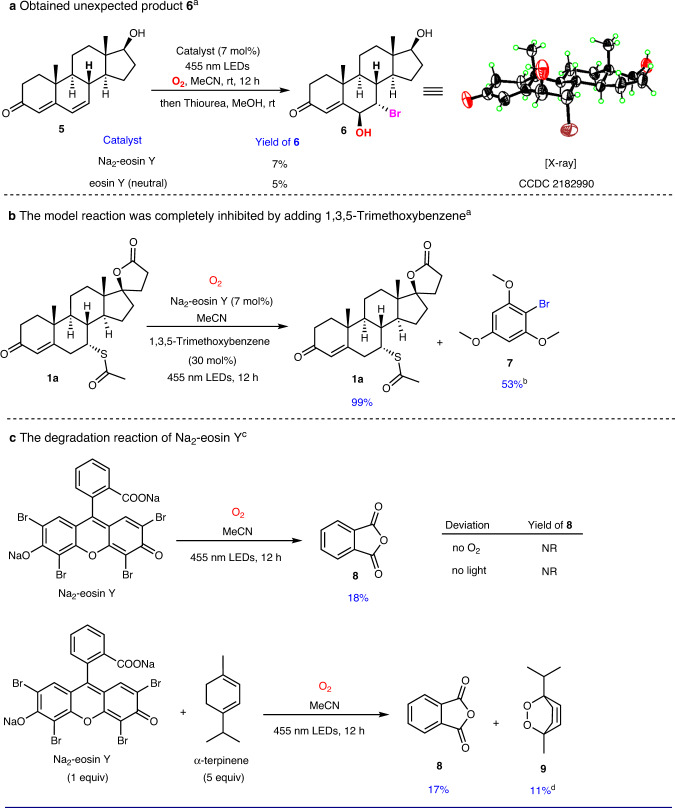
Selected mechanism studies. **a** Obtained unexpected bromide product **6**. **b** Bromine trapping experiment. **c** The degradation reaction of Na_2_-eosin Y. ^a^Reactions performed on 0.20 mmol scale with dimethyl terephthalate as an internal standard (^1^H NMR yield). ^b^Yield relative to 1,3,5-trimethoxybenzene. ^c^Reactions performed on 0.06 mmol scale with dimethyl terephthalate as an internal standard (^1^H NMR yield). ^d^Yield relative to α-terpinene. NR no reaction.

Next, we focused on the study of the reaction mechanism, and conducted a series of control experiments as listed in Table 2. In the presence of 10 mol% of 2,2,6,6-tetramethyl-1-piperinedinyloxy (TEMPO) or 3 equiv. of 2,6-di-*tert*-butyl-4-methylphenol (BHT), C–H oxidation was completely inhibited under the standard conditions with a radical adduct **4** being detected by high resolution mass spectrometry (HRMS), indicating the involvement of the allylic radical of enone (Table 2, entries 2–3). When one equiv. of PhSiH_3_ was added to the reaction mixture as a competitive hydrogen atom donor^40^, the model reaction was completely inhibited, suggesting that a hydrogen-atom-transfer pathway might be engaged (Table 2, entry 4). The yield slightly decreased by the addition of one equiv. of *tert*-butanol as a hydroxy radical scavenger, indicating that a hydroxy radical species may not be involved in the reaction (Table 2, entry 5). Furthermore, superoxide dismutase (SOD) was adopted to quench the superoxide anion, and the reaction was not influenced, indicating that a superoxide anion species may not be involved in the reaction (Table 2, entry 6). When 10 mol% of DABCO was added to the reaction mixture as a singlet oxygen quencher, the desired product **2a** was obtained in 36% yield (vs 85% without DABCO), indicating that singlet oxygen may be involved in the reaction (Table 2, entry 7). To examine the impact of light, we then conducted light on/off experiment for model reaction (Supplementary Fig. 5). The nature of the graph reveals that constant irradiation is necessary for this reaction as no conversion was observed in the dark period and it does not necessarily rule out the possibility of a radical chain process^41,42^. The Stern-Volmer quenching studies demonstrated that spironolactone **1a** was unable to quench the excited state of Na_2_-eosin Y (Supplementary Figs. 6 and 7), which ruled out the electron-transfer or energy-transfer process between the excited Na_2_-eosin Y and enones. We next sought to understand the detailed nature of the HAT step. First, the oxidation of 6-dehydrotestosterone **5** under standard conditions afforded an unexpected but interesting product **6** in 7% yield and no expected C-8 hydroxylation product was detected (Fig. 7a). The structure of compound **6** was confirmed by single crystal X-ray diffraction. From the view of reaction mechanism, compound **6** may be formed through a bromine radical addition and a subsequent oxidation pathway (Supplementary Fig. 9). In addition, the parallel tests of Na_2_-eosin Y and neutral eosin Y in this transformation indicated that both reagents underwent debromination reaction under standard conditions and they were likely not the key species responsible for the HAT from enones. Given the fact that bromine radical has been proved as a versatile HAT agent^43–45^, we envisioned that bromine radical may play an essential role in the reaction system. To evaluate the feasibility of the bromine radical initiating HAT in enones, a series of comparative experiments with various bromine sources as the catalyst were conducted (Supplementary Table 4). The results showed that Br_2_, NBS, and DBH could also generate the desired product, but in much lower yields, indicating that bromine radical could abstract hydrogen atoms from γ-C–H bonds of enones. Bromine trapping experiments were thus conducted to further understand the role of the bromine radical in the reactions (Fig. 7b). When 30 mol% of 1,3,5-trimethoxybenzene was added to the reaction mixture as the bromine trapper, the model reaction was completely inhibited, providing the solid support for the essential role of bromine radical. To shed light on the mechanism aspect of the debromination process of Na_2_-eosin Y, several experiments were further performed (Supplementary Figs. 10–14). Irradiating Na_2_-eosin Y in MeCN using 50 W 455 nm LEDs under an O_2_ atmosphere for 12 h resulted in the complete decomposition of Na_2_-eosin Y and the production of phthalic anhydride **8** as the only detectable degradation product (Fig. 7c). Control experiments indicated that O_2_ and light were both critical for the degradation reaction. When 5 equiv. of α-terpinene was added to the reaction system, the peroxidation product **9** was formed in 11% yield, confirming the involvement of singlet oxygen in the reaction system, which may be the cause of the oxidative debromination and degradation of Na_2_-eosin Y. On the basis of the aforementioned experiments and observations, a plausible reaction mechanism was proposed, and the discussions were included in the Supplementary Information (Supplementary Fig. 15).

**Table 2:** Quenching experiments for the hydroxylation of spironolactone (1a)^a^


Entry	Quencher	Equiv.	Yield of 2a (%)^b^
1	None	–	85
2	TEMPO	10 mol%	0
3	BHT	3 equiv.	0
4	PhSiH_3_	1 equiv.	0
5	*t*-BuOH	1 equiv.	76
6	Superoxide Dismutase	17 mg	82
7	DABCO	10 mol%	36

^a^Reaction conditions: **1a** (0.20 mmol), Na_2_-eosin Y (7 mol%), quencher, O_2_ balloon, and MeCN (10 mL) at r.t. under the irradiation of 50 W 455 nm LEDs for 12 h; then thiourea (0.24 mmol, 1.2 equiv.) and MeOH (10 mL) were added and stirred for 4 h.

^b^Isolated yield. TEMPO = 2,2,6,6-tetramethyl-1-piperinedinyloxy. BHT = 2,6-Di-*tert*-butyl-4-methylphe-nol. DABCO = 1,4-Diazabicyclo[2.2.2]octane. Superoxide Dismutase: recombinant, expressed in *E. coli*, 1636 units/mg.

In summary, we have developed a visible-light-induced catalytic platform for the allylic hydroxylation of enones under mild, metal- and peroxide-free reaction conditions. This method employs inexpensive and commercially available Na_2_-eosin Y as the photocatalyst and molecular oxygen (or ambient air) as a sustainable oxidant. Preliminary mechanism studies suggest that Na_2_-eosin Y serves as both the photocatalyst and the bromine radical precursor. This practical method exhibited the merits of broad substrates, high level of functional group tolerance, and easy experimental operation, which is much useful to install hydroxy group into the complex natural products and drugs bearing an enone moiety for late-stage functionalization. Moreover, this practical and scalable method has the potential to be adopted in large-scale industrial production.

## Methods

### General procedure for allylic hydroxylation of enones

Enone **1** (0.20 mmol, 1.0 equiv.), Na_2_-eosin Y (0.014 mmol, 7 mol%) and MeCN (10 mL) were added to a 100 mL eggplant-shaped bottle. After purging the flask with vacuum, O_2_ from a balloon was bubbled through the reaction mixture for 3 min. Then the reaction mixture was stirred for 5−15 h under 50 W 455 nm LED irradiation (PLS-100C, Beijing Perfectlight®, distance ~5 cm) under an O_2_ atmosphere at room temperature. When the reaction finished (monitored by TLC), the reaction solution was concentrated in vacuo, then thiourea (0.24 mmol, 1.2 equiv.) and MeOH (10 mL) were added to the mixture and stirred for 4 h. Then the reaction solution was concentrated in vacuo to afford a crude product, which was then partitioned with EtOAc (3 × 15 mL) in water (10 mL). The combined organic layers were washed with saturated aq. NaHCO_3_ and brine, and dried over anhydrous Na_2_SO_4_. After filtration, the solvent was removed under reduced pressure. The product was then purified by flash chromatography on silica gel to furnish the desired product.

### Reporting summary

Further information on research design is available in the Nature Portfolio Reporting Summary linked to this article.

## Supplementary information

Supplementary Information

Description of Additional Supplementary Files

Supplementary Data 1

Reporting Summary

## Data Availability

The crystallographic data generated in this study have been deposited in the Cambridge Crystallographic Data Centre under accession codes CCDC 2220929 (**2** **l**), CCDC 2216319 (**2m**), CCDC 2221557 (**2o**), CCDC 2221555 (**2r**), CCDC 2182987 (**2ag**), CCDC 2182988 (**2ai**), CCDC 2182989 (**2aj-2**), and CCDC 2182990 (**6**). These data can be obtained free of charge via www.ccdc.cam.ac.uk/data_request/cif. Crystal data and CheckCif reports for compounds **2l**, **2m**, **2o**, **2r, 2ag, 2ai, 2aj-2**, and **6** are available in Supplementary Data 1. The authors declare that all other data supporting the findings of this study are available within the main text and its supplementary information files.
