# Supplementary material for: Allylic hydroxylation of enones useful for the functionalization of relevant drugs and natural products
Source: Nat Commun. 2023 Apr 26;14:2399. doi: 10.1038/s41467-023-38154-9 (PMC10133259; doi:10.1038/s41467-023-38154-9)
Supplement: Supplementary file 1 — Supplementary Information [file 41467_2023_38154_MOESM1_ESM.pdf]

Supplementary Information

Allylic Hydroxylation of Enones Useful for the Functionalization of Relevant Drugs and Natural Products

Cheng-Yu Zheng[1,2] & Jian-Min Yue[1,2*]

[1]State Key Laboratory of Drug Research, Shanghai Institute of Materia Medica, Chinese Academy of Sciences, 555 Zuchongzhi Road, Shanghai 201203, China.

[2]University of Chinese Academy of Sciences, No.19A Yuquan Road, Beijing 100049, China.

*E-mail: jmyue@simm.ac.cn

Table of Contents

1. Supplementary Methods 1
   1.1 General Information 1
2. Supplementary Discussion 2
   2.1 Photocatalyst Screening 2
   2.2 Solvent Screening 4
   2.3 Light source Screening 5
   2.4 General Procedure for C–H Hydroxylation of Enones on 0.20 mmol Scale 5
   2.5 Procedures for C–H Hydroxylation of Enones on Gram scale 6
   2.6 Procedures for C–H Hydroxylation of Enones with Ambient Air as the Oxidant on 500 mg Scale 8
   2.7 HRMS data Analysis 11
   2.8 Light on/off Experiments 12
   2.9 Stern-Volmer Fluorescence Quenching Studies 13
   2.10 One Possible Explanation for the Effects of C7 Substituent in Steroids 14
   2.11 Proposed Mechanism for the Formation of Compound 6 15
   2.12 Bromine Source Screening 16
   2.13 Bromine Trapping Experiments 17
   2.14 The Degradation Reaction of Na_2-eosin Y 17
   2.15 The Proposed Mechanism 22
   2.16. X-Ray Crystallographic Data 23
   2.17 Preparation of Starting Materials 31
   2.18. Product Characterization 36
   2.19 NMR Spectral Data 58
3. Supplementary Notes 113
   3.1 Details of the prices of reagents 113
4. Supplementary References 114

1. Supplementary Methods

1.1 General Information

Unless otherwise noted, all reactions were carried out under an atmosphere of O_2 in oven-dried glassware. Commercially available reagents were used without further purification, unless otherwise noted.

NMR data were obtained on Bruker AVANCE Ⅲ 400, AVANCE Ⅲ 500, and/or Ascend 600 NMR spectrometers and were calibrated using residual undeuterated solvent ($CHCl_3$ at 7.26 ppm ^{1}H NMR, 77.16 ppm ^{13}C NMR; CH_3OH at 3.31 ppm ^{1}H NMR, 49.0 ppm ^{13}C NMR).

Reactions were monitored by thin layer chromatography (TLC) and column chromatography purifications were carried out using silica gel (300–400 mesh).

The X-ray diffraction analysis was performed on a Bruker SMART CCD detector employing graphite monochromated Cu-Kα radiation.

ESIMS and HRESIMS were implemented on a Bruker Daltonics Esquire 3000 plus and Waters-Micromass Q-TOF Ultima Global mass spectrometer, respectively.

High power LED light source (365 nm, 385 nm, 400 nm, 420 nm, 455nm and 535 nm) were purchased from Beijing Perfectlight Technology Co., Ltd. (PLS-LED100C).

Fluorescence quenching studies were performed using a Techcomp FL970 Fluorescence Spectrometer.

2. Supplementary Discussion

2.1 Photocatalyst Screening

Supplementary Table 1. Investigation of photocatalysts[a].

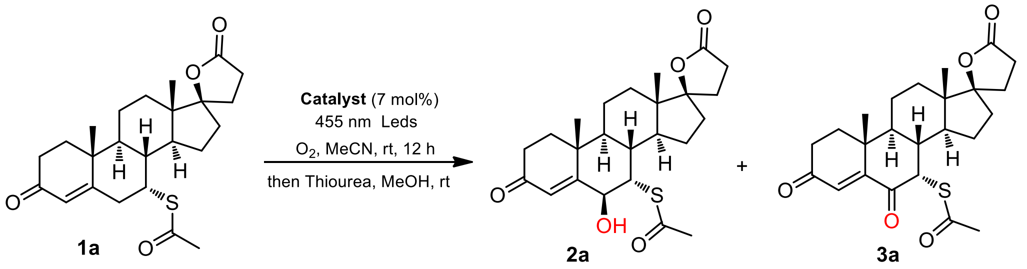

Entry	Catalyst	Yield (**2a** + **3a**, %)[b]	Ratio (**2a**:**3a**)[c]
1	Fluorescein	n.d.	-
2	Rose bengal	n.d.	-
3	Rose bengal lactone	n.d.	-
4	Erythrosine	n.d.	-
5	Mes-AcrClO_4	n.d.	-
6	4-MeO-TPT	n.d.	-
7	Eosin Y (neutral)	71	5.6:1
8	Na_2-eosin Y	90	20:1
9	K_2Eosin Y	84	12.5:1
10	Eosin B	68	20:1
11	Phloxine B	76	20:1
12[d]	TBADT	decomposed	-
13[e]	AQ	trace	-
14	PT	trace	-

[a]Standard conditions: **1a** (0.2 mmol), **catalyst** (7 mol%), O_2 balloon, and MeCN (10 mL) at r.t. under the irradiation of 50 W 455 nm LED for 12 h; then thiourea (0.24 mmol, 1.2 equiv) and MeOH (10 mL) were added and stirred for 4 h. [b]Isolated yield. [c]Determined by NMR analysis of the mixture of **2a** and **3a** after purification by flash chromatography. [d]A 365 nm LED was used. [e]A 420 nm LED was used. AQ = anthraquinone. PT = 5,7,12,14-pentacenetetrone. TBADT = tetrabutylammonium decatungstate. DCE = 1,2-dichloroethane. n.d. = not detected.

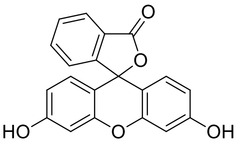

Fluorescein
2321-07-5

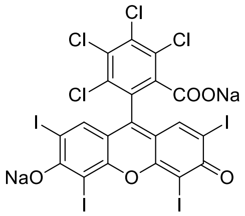

Rose bengal
632-69-9

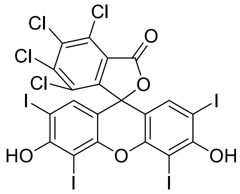

Rose bengal lactone
4159-77-7

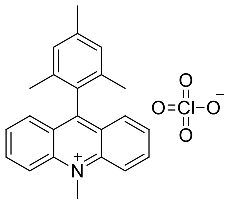

Mes-AcrClO_4
674783-97-2

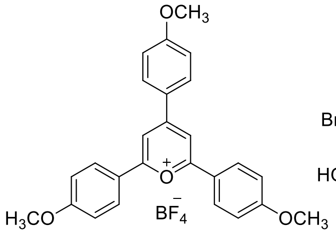

4-MeO-TPT
580-34-7

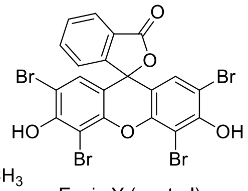

Eosin Y (neutral)
15086-94-9

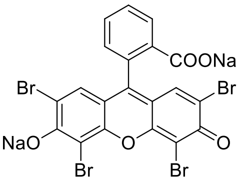

Na_2-eosin Y
17372-87-1

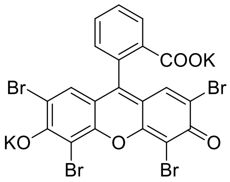

K_2-eosin Y
56897-54-2

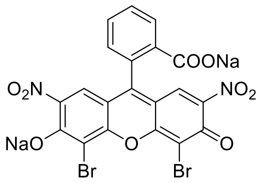

Eosin B
548-24-3

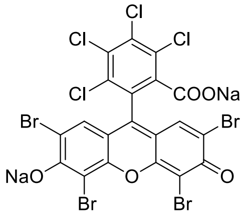

Phloxine B
18472-87-2

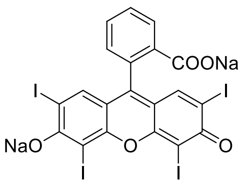

Erythrosine
16423-68-0

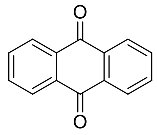

AQ
84-65-1

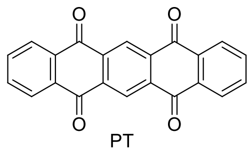

PT
23912-79-0

Tetrabutylammonium decatungstate

TBADT
68109-03-5

Supplementary Fig. 1. Structures of photocatalysts.

2.2 Solvent Screening

Supplementary Table 2. Investigation of solvents[a].

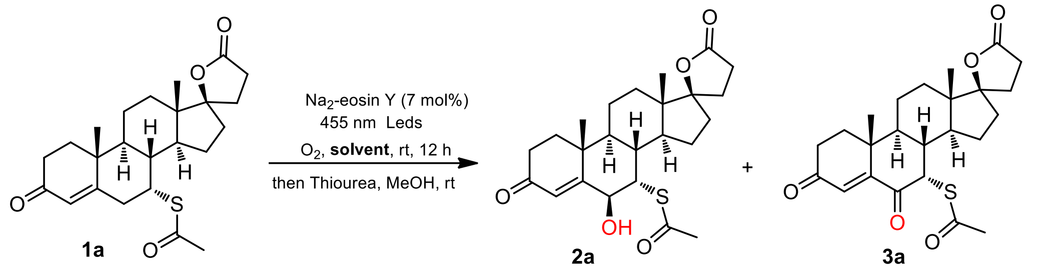

Entry	Solvent	Yield (**2a** + **3a**, %)[b]	Ratio (**2a**:**3a**)[c]
1	Acetone	89	14.3:1
2	DCE	trace	-
3	*t*-BuOH	n.d.	-
4	MeOH	n.d.	-
5	EtOAc	86	3.6:1
6	$CHCl_3$	n.d.	-
7	MeCN	90	20:1

[a]Standard conditions: **1a** (0.2 mmol), Na_2-eosin Y (7 mol%), O_2 balloon, and **Solvent** (10 mL) at r.t. under the irradiation of 50 W 455 nm LED for 12 h; then thiourea (0.24 mmol, 1.2 equiv) and MeOH (10 mL) were added and stirred for 4 h. [b]Isolated yield. [c]Determined by NMR analysis of the mixture of **2a** and **3a** after purification by flash chromatography. n.d. = not detected.

2.3 Light source Screening

Supplementary Table 3. Investigation of light sources[a].

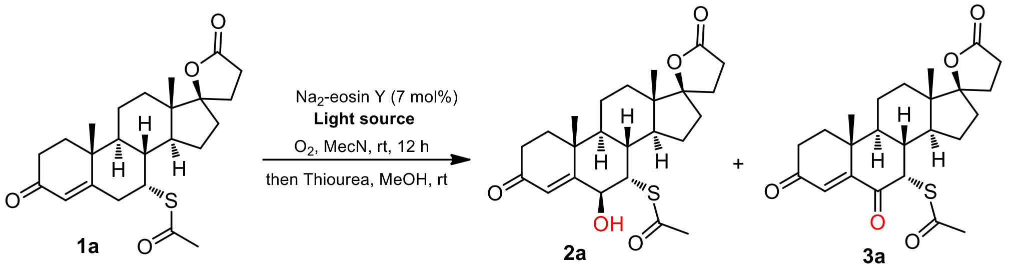

Entry	Light source	Yield (**2a** + **3a**, %)[b]	Ratio (**2a**:**3a**)[c]
1	535 nm LED	n.d.	-
2	455 nm LED	90	20:1
3	420 nm LED	71	6.3:1
4	400 nm LED	20	1.2:1
5	385 nm LED	9	1:1.3
6	65 W CFL	n.d.	-

[a]Standard conditions: **1a** (0.2 mmol), Na_2-eosin Y (7 mol%), O_2 balloon, and MeCN (10 mL) at r.t. under the irradiation of **light source** for 12 h; then thiourea (0.24 mmol, 1.2 equiv) and MeOH (10 mL) were added and stirred for 4 h. [b]Isolated yield. [c]Determined by NMR analysis of the mixture of **2a** and **3a** after purification by flash chromatography. n.d. = not detected.

2.4 General Procedure for C–H Hydroxylation of Enones on 0.20 mmol Scale

Enone **1** (0.20 mmol, 1.0 equiv), Na_2-eosin Y (0.014 mmol, 7 mol%) and MeCN (10 mL) were added to a 100 mL eggplant-shaped bottle. After purging the flask with vacuum, O_2 from a balloon was bubbled through the reaction mixture for 3 min. Then the reaction mixture was stirred for 5–15 h under 50 W 455 nm LED irradiation (PLS-100C, Beijing Perfectlight®, distance ~ 5 cm) under an O_2 atmosphere at room temperature. When the reaction finished (monitored by TLC), the reaction solution was concentrated in vacuo, then thiourea (0.24 mmol, 1.2 equiv) and MeOH (10 mL) were added to the mixture and stirred for 4 h. Then the reaction solution was concentrated in vacuo to afford a crude product, which was then partitioned with EtOAc (3 x 15 mL) in water (10 mL). The combined organic layers were washed with

saturated aq. $NaHCO_3$ and brine, and dried over anhydrous Na_2SO_4. After filtration, the solvent was removed under reduced pressure. The product was then purified by flash chromatography on silica gel to furnish the desired product.

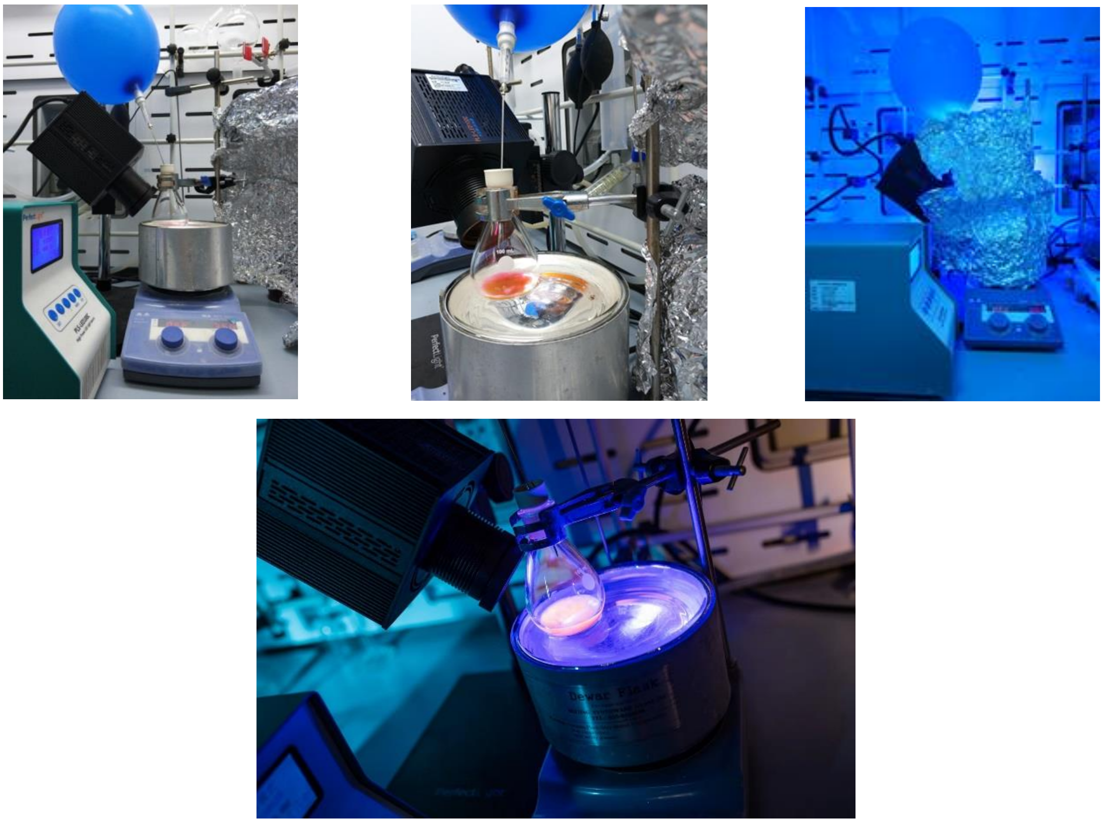

Supplementary Fig. 2. Pictures of the reaction irradiation setup (under an O_2 balloon).

2.5 Procedures for C–H Hydroxylation of Enones on Gram scale

6β- Hydroxyspironolactone (**2a**):

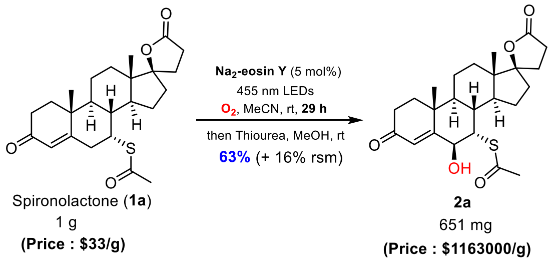

Spironolactone (**1a**) (1.0 g, 2.40 mmol, 1.0 equiv), Na_2-eosin Y (83 mg, 0.12 mmol, 5 mol%) and MeCN (70 mL) were added to a 200 mL eggplant-shaped bottle. After purging the flask with vacuum, O_2 from a balloon was bubbled through the reaction mixture for 3 min. Then the reaction mixture was stirred for 29 h under 50 W 455 nm LED irradiation (PLS-100C, Beijing Perfectlight®, distance ~ 5 cm) under an O_2

atmosphere at room temperature. The reaction solution was concentrated in vacuo, then thiourea (219 mg, 2.88 mmol, 1.2 equiv) and MeOH (30 mL) were added to the mixture and stirred for 4 h. Then the reaction solution was concentrated in vacuo and water (30 mL) was added. Finally, the mixture was extracted with EtOAc (3 x 20 mL). The combined organic layers were washed with saturated aq. $NaHCO_3$ and brine, dried over anhydrous Na_2SO_4, filtered, and concentrated under reduced pressure. The resulting crude product was then purified by flash chromatography on silica gel (PE/EtOAc = 1/1) to furnish a mixture of **2a** (651 mg, 1.51 mmol, 63%) and **3a** (52 mg, 0.12 mmol, 5%) and pure recovered **1a** (163 mg, 0.39 mmol, 16%).

6β-Hydroxyeplerenone (**2ag**):

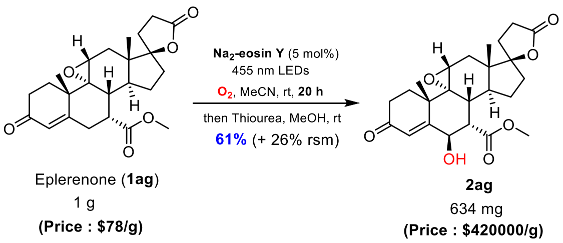

Eplerenone (**1ag**) (1.0 g, 2.41 mmol, 1.0 equiv), Na_2-eosin Y (83 mg, 0.12 mmol, 5 mol%) and MeCN (70 mL) were added to a 200 mL eggplant-shaped bottle. After purging the flask with vacuum, O_2 from a balloon was bubbled through the reaction mixture for 3 min. Then the reaction mixture was stirred for 20 h under 50 W 455 nm LED irradiation (PLS-100C, Beijing Perfectlight®, distance ~ 5 cm) under an O_2 atmosphere at room temperature. The reaction solution was concentrated in vacuo, then thiourea (220 mg, 2.89 mmol, 1.2 equiv) and MeOH (30 mL) were added to the mixture and stirred for 4 h. Then the reaction solution was concentrated in vacuo and water (30 mL) was added. Finally, the mixture was extracted with EtOAc (3 x 20 mL). The combined organic layers were washed with saturated aq. $NaHCO_3$ and brine, dried over anhydrous Na_2SO_4, filtered, and concentrated under reduced pressure. The resulting crude product was then purified by flash chromatography on silica gel (PE/EtOAc = 1/5) to furnish **2ag** (634 mg, 1.47 mmol, 61%) and recovered **1ag** (262 mg, 0.63 mmol, 26%).

6-Hydroxymedroxyprogesterone acetate (**2aj**):

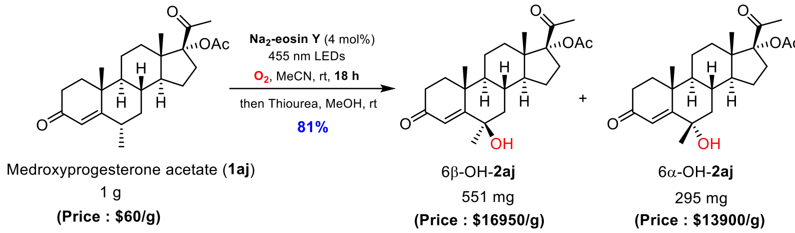

Medroxyprogesterone acetate (**1aj**) (1.0 g, 2.59 mmol, 1.0 equiv), Na$_2$-eosin Y (72 mg, 0.104 mmol, 4 mol%) and MeCN (75 mL) were added to a 200 mL eggplant-shaped bottle. After purging the flask with vacuum, O_2 from a balloon was bubbled through the reaction mixture for 3 min. Then the reaction mixture was stirred for 18 h under 50 W 455 nm LED irradiation (PLS-100C, Beijing Perfectlight®, distance ~ 5 cm) under an O_2 atmosphere at room temperature. The reaction solution was concentrated in vacuo, then thiourea (237 mg, 3.11 mmol, 1.2 equiv) and MeOH (30 mL) were added to the mixture and stirred for 4 h. Then the reaction solution was concentrated in vacuo and water (30 mL) was added. Finally, the mixture was extracted with EtOAc (3 x 20 mL). The combined organic layers were washed with saturated aq. $NaHCO_3$ and brine, dried over anhydrous Na_2SO_4, filtered, and concentrated under reduced pressure. The resulting crude product was then purified by flash chromatography on silica gel (PE/EtOAc = 2/3) to furnish 6β-hydroxymedroxyprogesterone acetate (**2aj-1**) (551 mg, 1.37 mmol, 53%) and 6α-hydroxymedroxyprogesterone acetate (**2aj-2**) (295 mg, 0.73 mmol, 28%).

2.6 Procedures for C–H Hydroxylation of Enones with Ambient Air as the Oxidant on 500 mg Scale

10β-Hydroxylevonorgestrel (**2ai**):

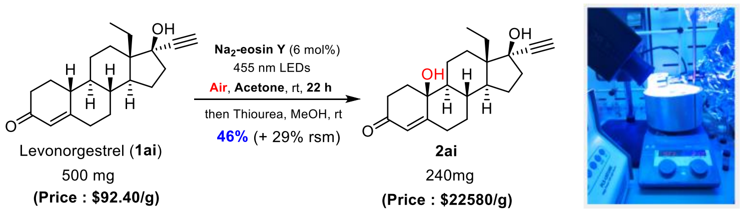

Levonorgestrel (**1ai**) (500 mg, 1.60 mmol, 1.0 equiv), Na$_2$-eosin Y (66 mg, 0.096 mmol, 6 mol%) and Acetone (80 mL) were added to a 100 mL beaker. Then the

reaction mixture was stirred open to air for 22 h under 50 W 455 nm LED irradiation (PLS-100C, Beijing Perfectlight®, distance ~ 5 cm) at room temperature. The reaction solution was concentrated in vacuo, then thiourea (146 mg, 1.92 mmol, 1.2 equiv) and MeOH (30 mL) were added to the mixture and stirred for 4 h. Then the reaction solution was concentrated in vacuo and water (30 mL) was added. Finally, the mixture was extracted with EtOAc (3 x 20 mL). The combined organic layers were washed with saturated aq. $NaHCO_3$ and brine, dried over anhydrous Na_2SO_4, filtered, and concentrated under reduced pressure. The resulting crude product was then purified by flash chromatography on silica gel (PE/EtOAc = 1/1) to furnish **2ai** (240 mg, 0.73 mmol, 46%) and recovered **1ai** (144 mg, 0.46 mmol, 29%).

6-Hydroxymedroxyprogesterone acetate (**2aj**):

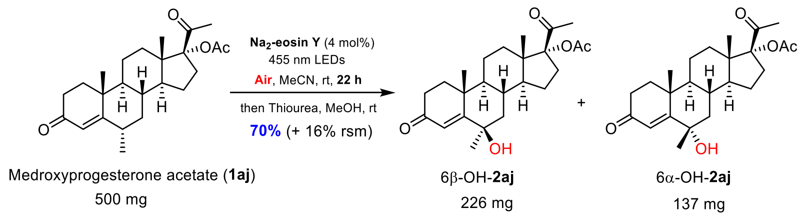

Medroxyprogesterone acetate (**1aj**) (500 mg, 1.29 mmol, 1.0 equiv), Na_2-eosin Y (36 mg, 0.052 mmol, 4 mol%) and MeCN (37 mL) were added to a 100 mL beaker. Then the reaction mixture was stirred open to air for 22 h under 50 W 455 nm LED irradiation (PLS-100C, Beijing Perfectlight®, distance ~ 5 cm) at room temperature. The reaction solution was concentrated in vacuo, then thiourea (118 mg, 1.55 mmol, 1.2 equiv) and MeOH (20 mL) were added to the mixture and stirred for 4 h. Then the reaction solution was concentrated in vacuo and water (30 mL) was added. Finally, the mixture was extracted with EtOAc (3 x 20 mL). The combined organic layers were washed with saturated aq. $NaHCO_3$ and brine, dried over anhydrous Na_2SO_4, filtered, and concentrated under reduced pressure. The resulting crude product was then purified by flash chromatography on silica gel (PE/EtOAc = 2/3) to furnish 6β-hydroxymedroxyprogesterone acetate (**2aj-1**) (226 mg, 0.56 mmol, 44%) and 6α-hydroxymedroxyprogesterone acetate (**2aj-2**) (137 mg, 0.34 mmol, 26%) and recovered **1aj** (79 mg, 0.20 mmol, 16%).

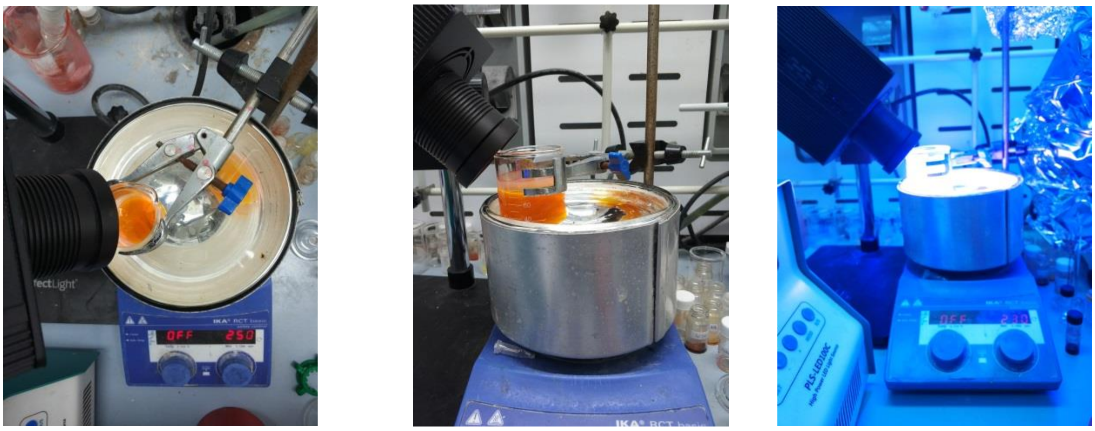

Supplementary Fig. 3. Pictures of the reaction irradiation setup (open to air).

2.7 HRMS data Analysis

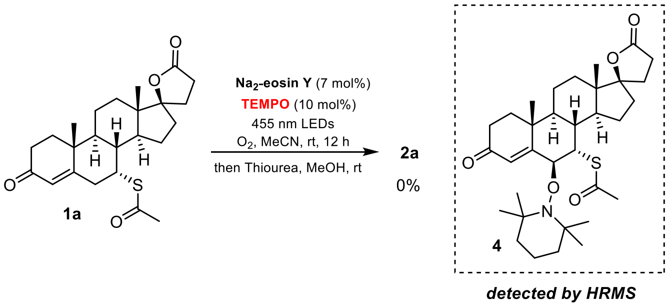

m/z: $[M+H]^+$ Calcd for $C_{33}H_{50}NO_5S$ 572.3404, found 572.3403

Qualitative Analysis Report

**Data Filename**	ESIH202103117-1.d	**Sample Name**	D4-TEM1
**Sample ID**		**Position**	P1-B4
**Instrument Name**	Agilent G6520 Q-TOF	**Acq Method**	20160322_MS_ESIH_POS_1min.m
**Acquired Time**	6/22/2021 17:48:12	**IRM Calibration Status**	Success
**DA Method**	small molecular data analysis method.m	**Comment**	ESIH by zhuzhenyun

User Spectra

Fragmentor Voltage	Collision Energy	Ionization Mode
135	0	ESI

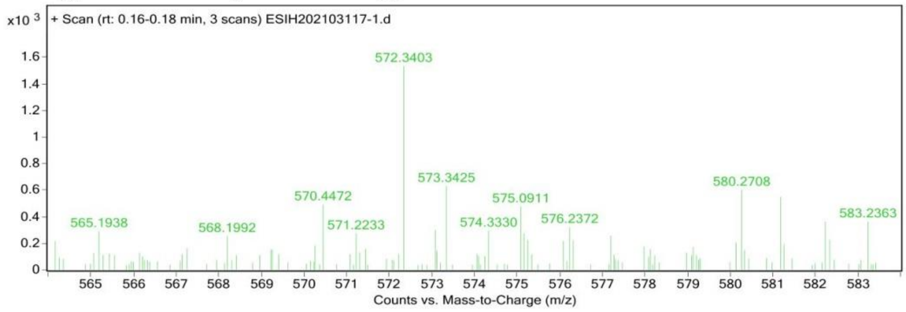

Formula Calculator Results

m/z	Calc m/z	Diff (mDa)	Diff (ppm)	Ion Formula	Ion
572.3403	572.3404	0.07	0.13	C33 H50 N O5 S	(M+H)+

--- End Of Report ---

Supplementary Fig. 4. The HRMS analysis.

2.8 Light on/off Experiments

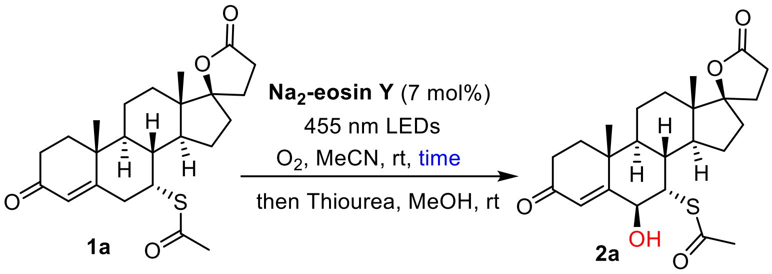

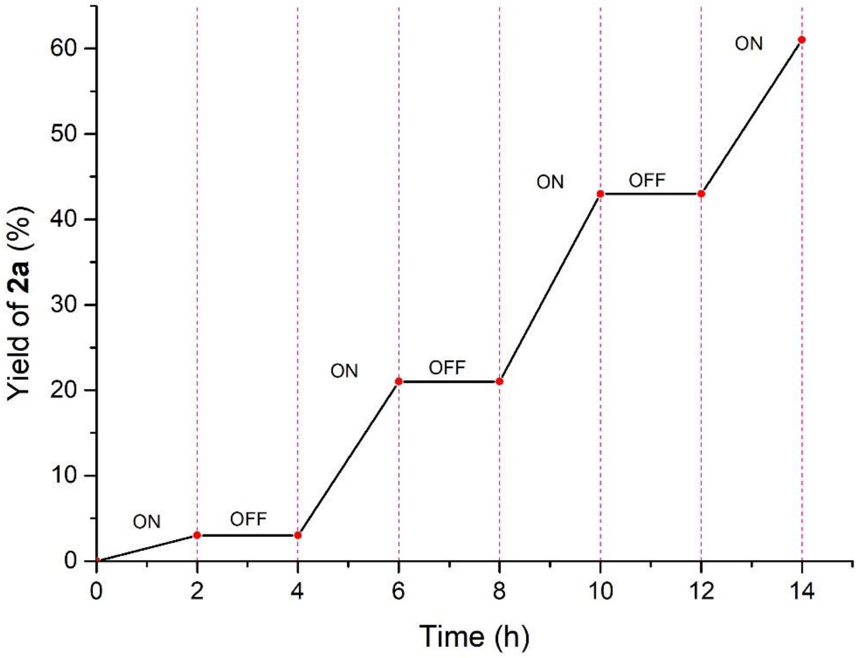

Supplementary Fig. 5. Light on/off experiments.

To examine the impact of light, we conducted light on/off experiment for model reaction. The nature of the graph reveals that constant irradiation is necessary for this reaction as no conversion was observed in the dark period and it does not necessarily rule out the possibility of a radical chain process.

2.9 Stern-Volmer Fluorescence Quenching Studies

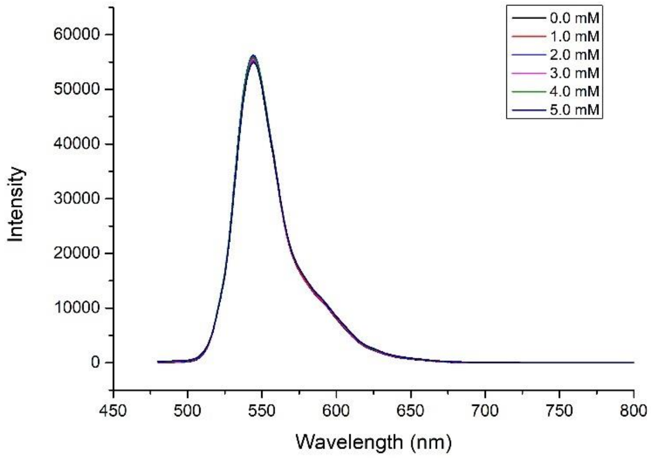

Supplementary Fig. 6. Na_2-eosin Y emission quenching by **1a**.

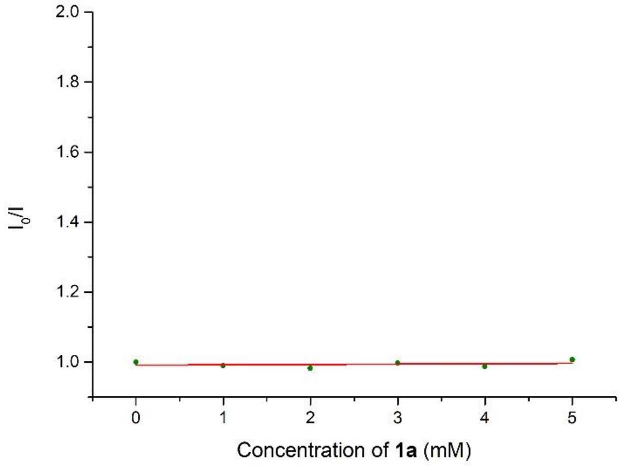

Supplementary Fig. 7. Stern-Volmer Plot for the quenching study using **1a** as a quencher.

Fluorescence quenching studies were performed using a Techcomp FL970 Fluorescence Spectrometer. In each experiment, the photocatalyst and varying concentrations of quencher were dissolved in CH_3CN in screw-top 1.0 cm quartz cuvettes and degassed by sparging with argon for 20 minutes.

For the emission quenching of Na_2-eosin Y, the photocatalyst concentration was 0.21 μM, the solution was irradiated at 455 nm and the emission intensity was observed at 544 nm. Plots were constructed according to the Stern–Volmer equation $I_0/I = 1 + k_q\tau_0[Q]$. The Stern-Volmer quenching studies demonstrated that spironolactone 1a was unable to quench the excited state of Na_2-eosin Y. These results ruled out the electron-transfer or energy-transfer process between the excited Na_2-eosin Y and enones.

2.10 One Possible Explanation for the Effects of C7 Substituent in Steroids

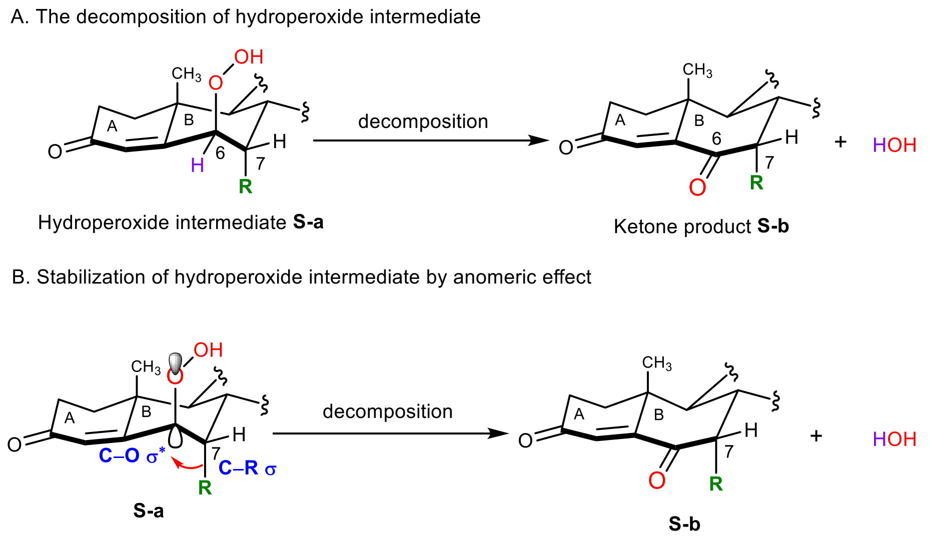

Supplementary Fig. 8. One possible explanation for the effects of C7 substituent in steroids.

For steroids bearing a CH_2-7 group, this protocol afforded significant quantities of ketone rather than secondary alcohol products (**2t**–**2v**). By contrast, the steroids bearing CHR-7 (R = SAc, Me, *n*-Bu, CO_2Me) as the case of spironolactone were oxidized smoothly to afford selectively secondary alcohol products in good yields (**2w**–**2ac**).

We propose the following possible explanation for the effects of C7 substituent in steroids. As illustrated in Supplementary Fig. 8A, during the oxidation process of steroid substrates at C6, the generated hydroperoxide intermediate **S-a** is reactive and tends to decompose to afford ketone product **S-b** and H_2O. Conformational analysis of **S-a** shows that the C7–R σ can overlap with C6–O σ* and electron density can move from C7–R bond into the C6–O bond, which can help stabilize the molecule (Supplementary Fig. 8B). Larger substituent R than H can make the B ring more rigid to help the C7–R σ and C6–O σ* lie parallel to stabilize the molecule better, preventing it from decomposing to form ketone products.

2.11 Proposed Mechanism for the Formation of Compound 6

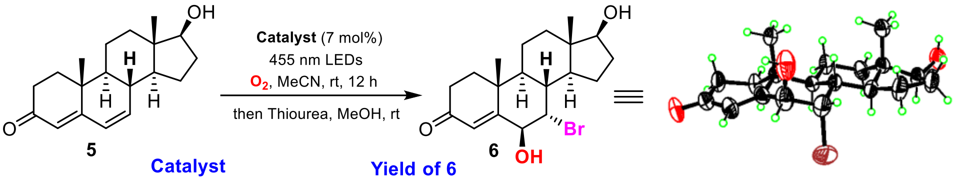

Catalyst	Yield of 6
Na_2-eosin Y	7%
Eosin Y (neutral)	5%

[X-ray]

General procedure for the synthesis of compound **6**

6-dehydrotestosterone **5** (0.20 mmol, 1.0 equiv), **catalyst** (0.014 mmol, 7 mol%) and MeCN (10 mL) were added to a 100 mL eggplant-shaped bottle. After purging the flask with vacuum, O_2 from a balloon was bubbled through the reaction mixture for 3 min. Then the reaction mixture was stirred for 12 h under 50 W 455 nm LED irradiation (PLS-100C, Beijing Perfectlight®, distance ~ 5 cm) under an O_2 atmosphere at room temperature. Then the reaction solution was concentrated in vacuo, then thiourea (0.24 mmol, 1.2 equiv) and MeOH (10 mL) were added to the mixture and stirred for 4 h. Then the reaction solution was concentrated in vacuo and water (10 mL) was added. Finally, the mixture was extracted with EtOAc (3 x 15 mL). The combined organic layers were washed with saturated aq. $NaHCO_3$ and brine, dried over anhydrous Na_2SO_4, filtered, and concentrated under reduced pressure. The yield was determined by ^{1}H NMR analysis using dimethyl terephthalate as an internal standard. The crude product was purified by flash chromatography (PE/EtOAc = 1/1) to furnish **6** as a white crystalline solid. The structure of **6** was confirmed by single crystal X-ray diffraction.

Proposed mechanism:

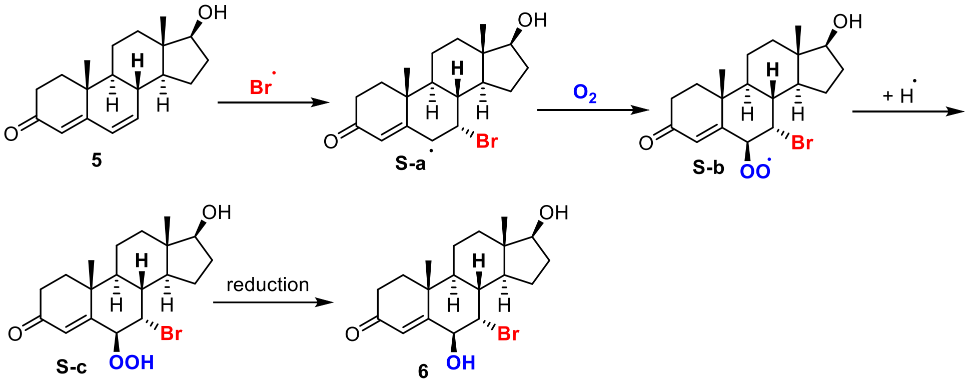

Supplementary Fig. 9. Proposed mechanism for the formation of compound **6**.

2.12 Bromine Source Screening

Supplementary Table 4. Investigation of different bromine source[a].

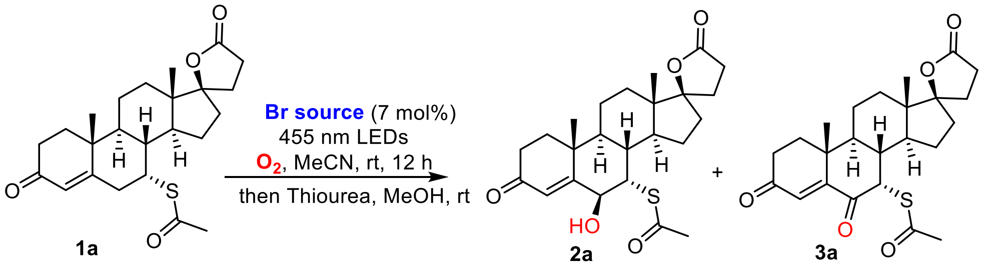

Entry	Br source	Yield of **2a**[b]	Yield of **3a**[b]
1	Br_2	4%	8%
2	NBS	5%	9%
3	CBr_4	n.d.	n.d.
4	DBH	5%	6%
5	TBAB	n.d.	n.d.

[a]Standard conditions: **1a** (0.2 mmol), Br source (7 mol%), O_2 balloon, and MeCN (10 mL) at r.t. under the irradiation of 50 W 455 nm LEDs for 12 h; then thiourea (0.24 mmol, 1.2 equiv) and MeOH (10 mL) were added and stirred for 4 h. [b]Yield was determined by ^{1}H NMR analysis using dimethyl terephthalate as an internal standard. NBS = N-Bromosuccinimide. DBH = 1,3-Dibromo-5,5-dimethylhydantoin. TBAB = Tetrabutylammonium bromide. n.d. = not detected.

The detailed procedure for the bromine source screening experiments

1a (0.20 mmol, 1.0 equiv), **Br source** (0.014 mmol, 7 mol%) and MeCN (10 mL) were added to a 100 mL eggplant-shaped bottle. After purging the flask with vacuum, O_2 from a balloon was bubbled through the reaction mixture for 3 min. Then the reaction mixture was stirred for 12 h under 50 W 455 nm LED irradiation (PLS-100C, Beijing Perfectlight®, distance ~ 5 cm) under an O_2 atmosphere at room temperature. Then the reaction solution was concentrated in vacuo, then thiourea (0.24 mmol, 1.2 equiv) and MeOH (10 mL) were added to the mixture and stirred for 4 h. Then the reaction solution was concentrated in vacuo and water (10 mL) was added. Finally, the mixture was extracted with EtOAc (3 x 15 mL). The combined organic layers were washed with saturated aq. $NaHCO_3$ and brine, dried over anhydrous Na_2SO_4, filtered, and concentrated under reduced pressure. The yields were determined by ^{1}H NMR analysis using dimethyl terephthalate as an internal standard.

2.13 Bromine Trapping Experiments

Supplementary Table 5. Bromine trapping experiments[a].

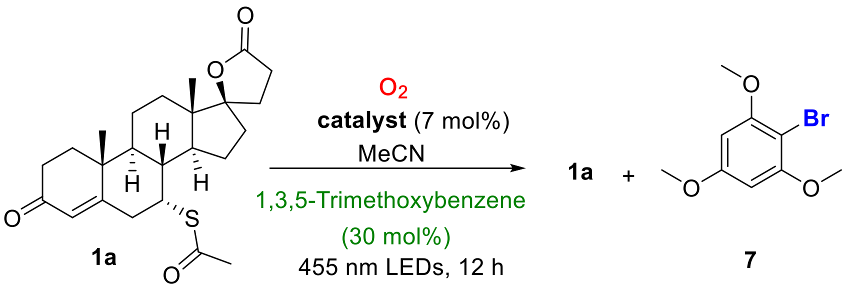

Entry	Catalyst	Yield of **1a**	Yield of **7**[b]
1	Na_2-eosin Y	99%	53%
2	Eosin Y (neutral)	99%	45%

[a]Reaction conditions: **1a** (0.2 mmol), catalyst (7 mol%), 1,3,5-trimethoxybenzene (30 mol%), O_2 balloon, and MeCN (10 mL) at r.t. under the irradiation of 50 W 455 nm LEDs for 12 h; Yield was determined by ^1H NMR analysis using dimethyl terephthalate as an internal standard. [b]Yield relative to 1,3,5-trimethoxybenzene.

The detailed procedure for the bromine trapping experiments

1a (0.20 mmol, 1.0 equiv), **catalyst** (0.014 mmol, 7 mol%), 1,3,5-trimethoxybenzene (0.06 mmol, 30 mol%) and MeCN (10 mL) were added to a 100 mL eggplant-shaped bottle. After purging the flask with vacuum, O_2 from a balloon was bubbled through the reaction mixture for 3 min. Then the reaction mixture was stirred for 12 h under 50 W 455 nm LED irradiation (PLS-100C, Beijing Perfectlight®, distance ~ 5 cm) under an O_2 atmosphere at room temperature. Then the reaction solution was concentrated in vacuo, and the yields were determined by ^1H NMR analysis using dimethyl terephthalate as an internal standard. The crude product was purified by flash chromatography (PE/EtOAc = 10/1) to furnish **7** as a white solid. Characterization data are in agreement with reported literature values.[1]

2.14 The Degradation Reaction of Na_2-eosin Y

Supplementary Table 6. The degradation reaction of Na_2-eosin Y[a].

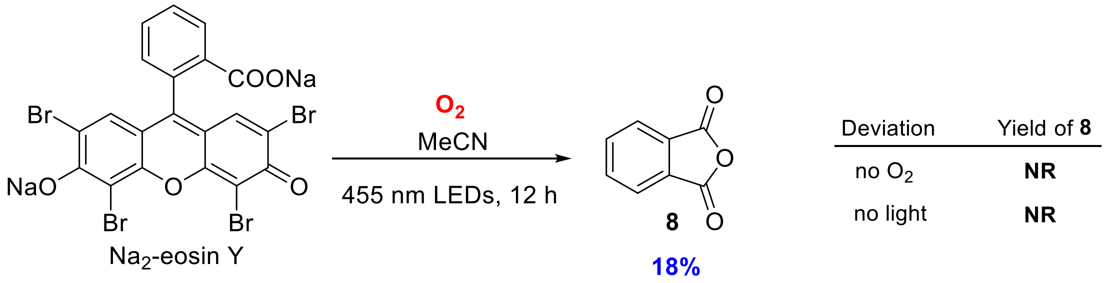

[a]Reaction conditions: Na_2-eosin Y (0.06 mmol), O_2 balloon, and MeCN (10 mL) at r.t. under the irradiation of 50 W 455 nm LEDs for 12 h; Yield was determined by ^1H NMR analysis using dimethyl terephthalate as an internal standard. NR = no reaction.

The detailed procedure for the degradation reaction of Na_2-eosin Y

Na_2-eosin Y (0.06 mmol) and MeCN (30 mL) were added to a 100 mL eggplant-shaped bottle. After purging the flask with vacuum, O_2 from a balloon was

bubbled through the reaction mixture for 3 min. Then the reaction mixture was stirred for 12 h under 50 W 455 nm LED irradiation (PLS-100C, Beijing Perfectlight®, distance ~ 5 cm) under an O_2 atmosphere at room temperature. Then the reaction solution was concentrated in vacuo, and DMSO-d_6 was added for ^1H NMR analysis. The yield was determined by ^1H NMR analysis using dimethyl terephthalate as an internal standard.

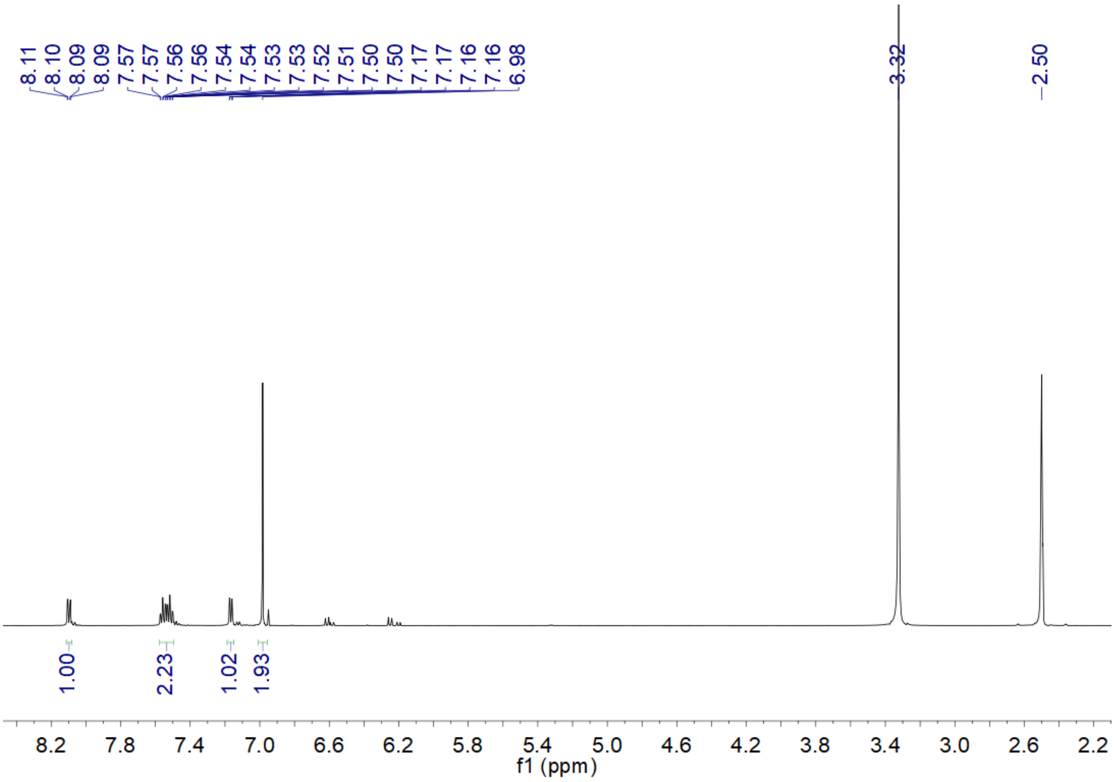

Supplementary Fig. 10. ^{1}H NMR (500 MHz, DMSO-d_6) of the reaction mixture (MeCN was removed *in vacuo*) before irradiation.

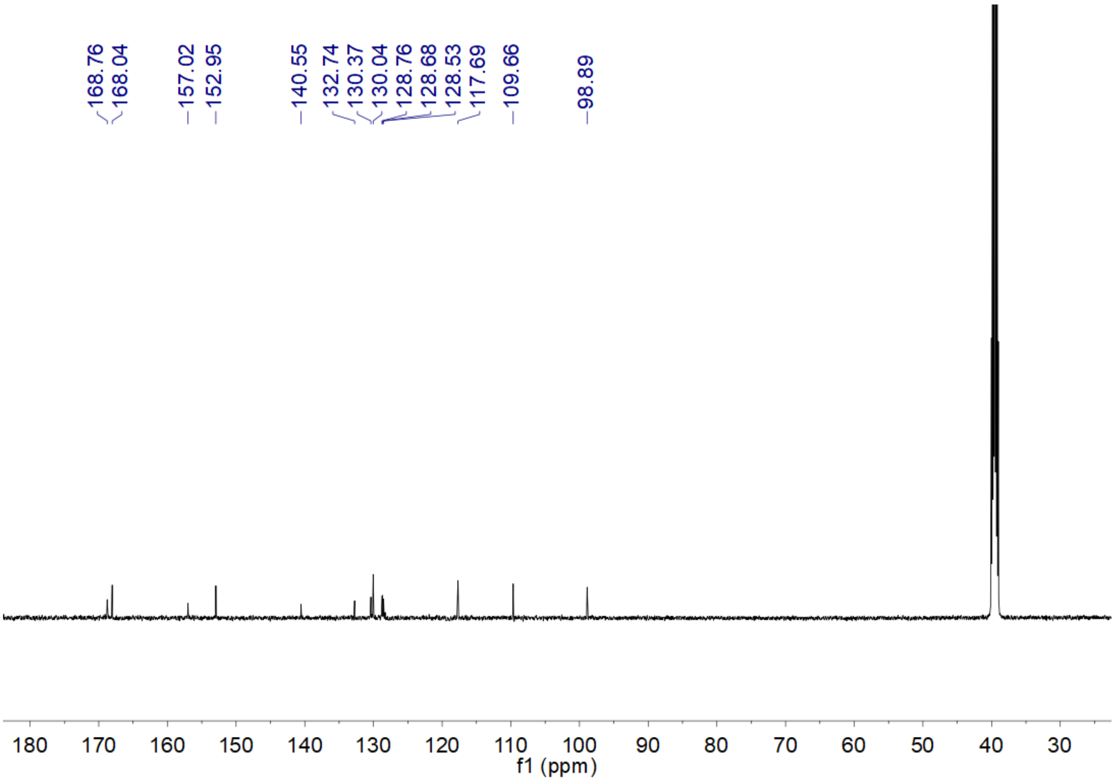

Supplementary Fig. 11. ^{13}C NMR (126 MHz, DMSO-d_6) of the crude reaction mixture (MeCN was removed *in vacuo*) before irradiation.

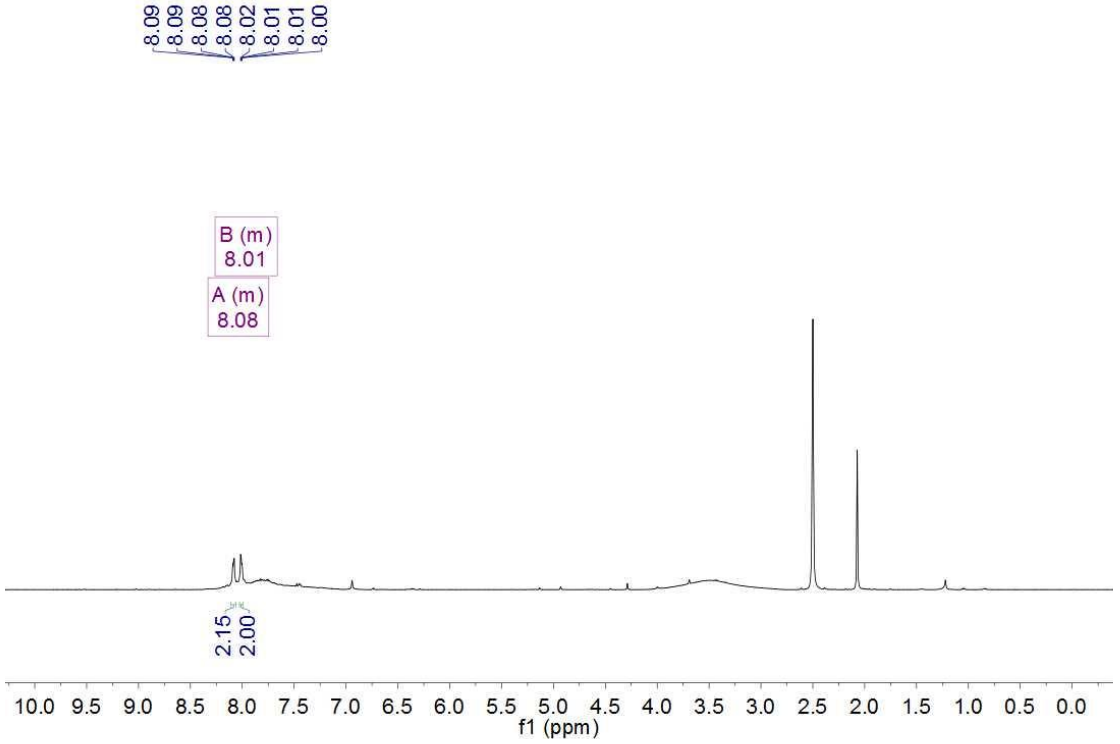

Supplementary Fig. 12. ^{1}H NMR (600 MHz, DMSO-d_6) of the crude reaction mixture (MeCN was removed *in vacuo*) after irradiation for 12 h.

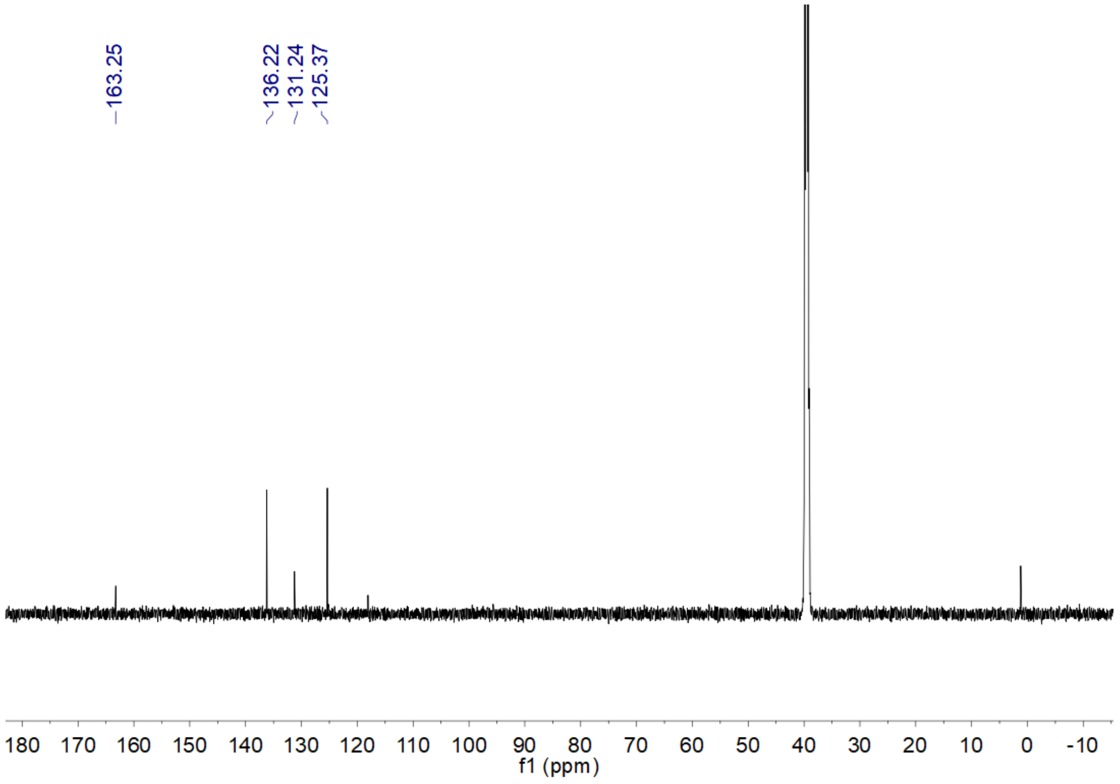

Supplementary Fig. 13. ^{13}C NMR (151 MHz, DMSO-d_6) of the crude reaction mixture (MeCN was removed *in vacuo*) after irradiation for 12 h.

The crude NMR analysis (Supplementary Figs. 10–13) showed that Na_2-eosin Y underwent complete degradation and the only detectable degradation product was

phthalic anhydride **8**, the NMR data of which are in agreement with reported literature values.[2]

Trapping of the singlet oxygen by α-terpinene

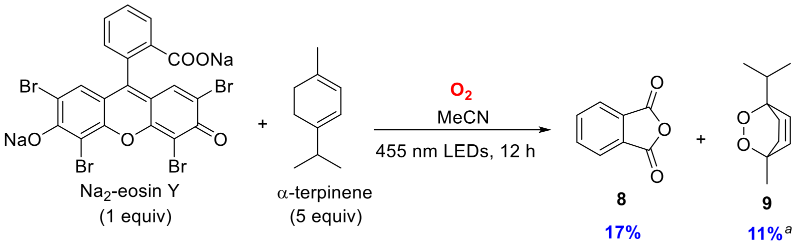

Supplementary Fig. 14. Trapping singlet oxygen using α-terpinene. Reaction conditions: Na_2-eosin Y (0.06 mmol, 1 equiv), α-terpinene (0.30 mmol, 5 equiv), O_2 balloon, and MeCN (30 mL) at r.t. under the irradiation of 50 W 455 nm LEDs for 12 h. Yield was determined by ^{1}H NMR analysis using dimethyl terephthalate as an internal standard. [a]Yield relative to α-terpinene.

The detailed procedure for the singlet oxygen trapping experiment

Na_2-eosin Y (0.06 mmol), α-terpinene (0.30 mmol, 5 equiv), and MeCN (30 mL) were added to a 100 mL eggplant-shaped bottle. After purging the flask with vacuum, O_2 from a balloon was bubbled through the reaction mixture for 3 min. Then the reaction mixture was stirred for 12 h under 50 W 455 nm LED irradiation (PLS-100C, Beijing Perfectlight®, distance ~ 5 cm) under an O_2 atmosphere at room temperature. Then the reaction solution was concentrated in vacuo, and the yields were determined by ^{1}H NMR analysis using dimethyl terephthalate as an internal standard. The crude product was purified by flash chromatography (PE/EtOAc = 10/1) to furnish **9** as a colorless oil. Characterization data are in agreement with reported literature values.[3]

2.15 The Proposed Mechanism

On the basis of the above experiments and observations, a plausible mechanism is proposed in Supplementary Fig. 15. Upon irradiation, the excited Na_2-eosin Y* undergoes energy transfer with $^3O_2$ to generate $^1O_2$, which then may react with Na_2-eosin Y to deliver Br radicals. Because the generated bromine radicals will readily transform into Br_2, the constant light irradiation is thus required for the homolysis of the undesired Br_2 to the radical form. The Br radical then abstracts a hydrogen atom from the enone **1b** to deliver an allylic radical **A** and HBr. The resultant allylic radical **A** is subsequently trapped by $^3O_2$ to afford a peroxyl radical **B**, which likely undergoes a reversed HAT with HBr to regenerate a Br radical and deliver a hydroperoxide **C**. Finally, the reduction of hydroperoxide **C** by thiourea will afford the required alcohol product **2b**; and the decomposition of hydroperoxide **C** would afford the ketone product **3b**. Likely, a Russell-type disproportionation of peroxyl radical **B** is also present to afford alcohol and ketone products.

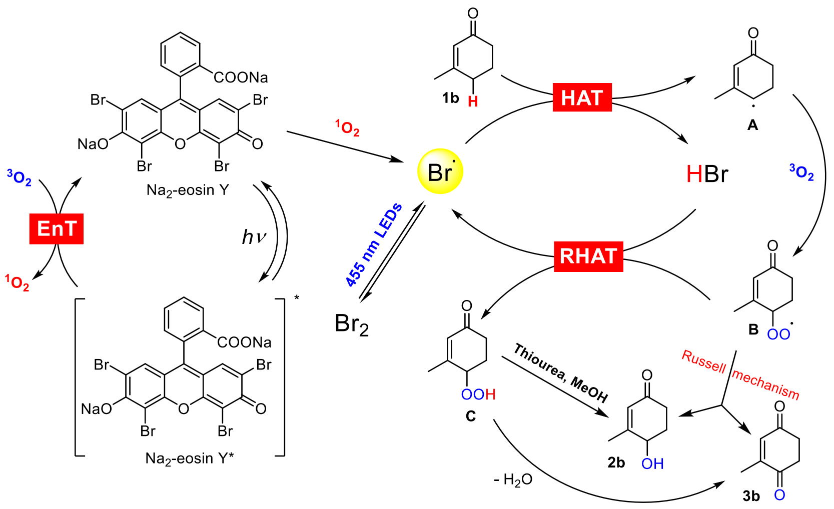

Supplementary Fig. 15. Proposed mechanism for visible-light-induced allylic hydroxylation of enones.

2.16. X-Ray Crystallographic Data

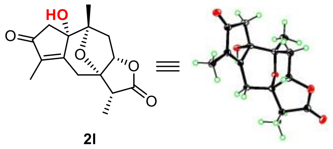

2l

from Hedyosumin B (CCDC 2220929)

Supplementary Fig. 16. The X-ray crystal structure of **2l** with thermal ellipsoids at the 40% probability level.

Supplementary Table 7. Crystal data and structure refinement for **2l**.

Identification code	K
Empirical formula	$C_{15}H_{18}O_5$
Formula weight	278.29
Temperature/K	100(2)
Crystal system	orthorhombic
Space group	$P2_12_12$
a/Å	12.1371(6)
b/Å	24.5157(11)
c/Å	9.1298(4)
α/°	90
β/°	90
γ/°	90
Volume/Å^3	2716.6(2)
Z	8
ρ_{calc}g/cm^3	1.361
μ/mm^{-1}	0.848
F(000)	1184.0
Crystal size/mm^3	0.11 × 0.06 × 0.05
Radiation	CuKα (λ = 1.54178)
2Θ range for data collection/°	7.212 to 149.46
Index ranges	$-15 \leq h \leq 15, -29 \leq k \leq 30, -11 \leq l \leq 11$
Reflections collected	34980
Independent reflections	5535 [R_{int} = 0.0586, R_{sigma} = 0.0344]
Data/restraints/parameters	5535/0/369
Goodness-of-fit on F^2	1.034
Final R indexes [I>=2σ (I)]	R_1 = 0.0339, wR_2 = 0.0838
Final R indexes [all data]	R_1 = 0.0360, wR_2 = 0.0858
Largest diff. peak/hole / e Å^{-3}	0.23/-0.21
Flack parameter	0.11(7)

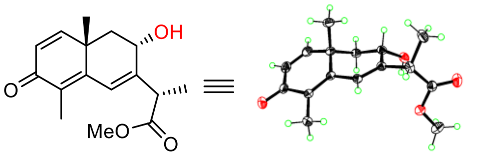

2m (CCDC 2216319)

Supplementary Fig. 17. The X-ray crystal structure of **2m** with thermal ellipsoids at the 40% probability level.

Supplementary Table 8. Crystal data and structure refinement for **2m**.

Identification code	ZZ
Empirical formula	$C_{16}H_{20}O_4$
Formula weight	276.32
Temperature/K	170.00
Crystal system	orthorhombic
Space group	$P2_12_12_1$
a/Å	10.2733(2)
b/Å	10.3627(2)
c/Å	13.3957(3)
α/°	90
β/°	90
γ/°	90
Volume/Å^3	1426.09(5)
Z	4
ρ_{calc}g/cm^3	1.287
μ/mm^{-1}	0.748
F(000)	592.0
Crystal size/mm^3	0.15 × 0.08 × 0.05
Radiation	CuKα (λ = 1.54178)
2Θ range for data collection/°	10.794 to 149.438
Index ranges	$-12 \leq h \leq 12$, $-12 \leq k \leq 12$,<br>$-16 \leq l \leq 16$
Reflections collected	14626
Independent reflections	2912 [R_{int} = 0.0411, R_{sigma} = 0.0277]
Data/restraints/parameters	2912/0/186
Goodness-of-fit on F^2	1.066
Final R indexes [I>=2σ (I)]	R_1 = 0.0325, wR_2 = 0.0849
Final R indexes [all data]	R_1 = 0.0342, wR_2 = 0.0869
Largest diff. peak/hole / e Å^{-3}	0.22/-0.18
Flack parameter	-0.07(9)

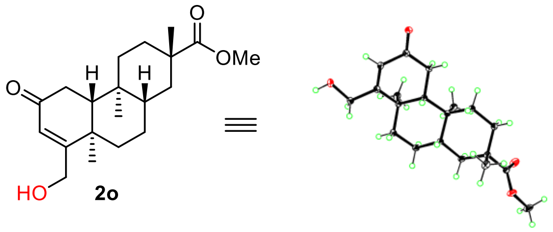

from **Koilodenoid F** methyl ester (CCDC 2221557)

Supplementary Fig. 18. The X-ray crystal structure of **2o** with thermal ellipsoids at the 40% probability level.

Supplementary Table 9. Crystal data and structure refinement for **2o**.

Identification code	cu_20221487_0m
Empirical formula	$C_{20}H_{30}O_4$
Formula weight	334.44
Temperature/K	100.00
Crystal system	orthorhombic
Space group	$P2_12_12_1$
a/Å	7.6687(3)
b/Å	14.4724(6)
c/Å	15.8033(7)
α/°	90
β/°	90
γ/°	90
Volume/Å^3	1753.92(13)
Z	4
ρ_{calc}g/cm^3	1.267
μ/mm^{-1}	0.692
F(000)	728.0
Crystal size/mm^3	0.12 × 0.08 × 0.05
Radiation	CuKα (λ = 1.54178)
2Θ range for data collection/°	8.284 to 149.136
Index ranges	$-9 \le h \le 9$, $-16 \le k \le 17$,<br>$-19 \le l \le 19$
Reflections collected	14466
Independent reflections	3500 [R_{int} = 0.0567, R_{sigma} = 0.0405]
Data/restraints/parameters	3500/0/222
Goodness-of-fit on F^2	1.066
Final R indexes [I>=2σ (I)]	R_1 = 0.0330, wR_2 = 0.0817
Final R indexes [all data]	R_1 = 0.0360, wR_2 = 0.0898
Largest diff. peak/hole / e Å^{-3}	0.19/-0.20
Flack parameter	0.01(10)

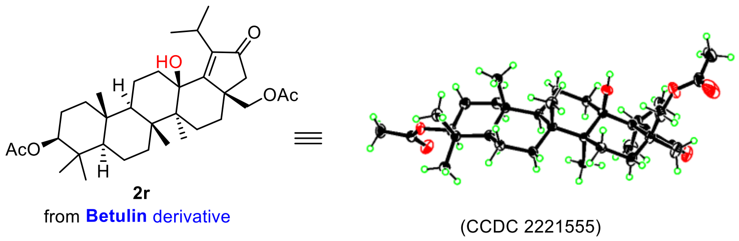

Supplementary Fig. 19. The X-ray crystal structure of **2r** with thermal ellipsoids at the 40% probability level.

Supplementary Table 10. Crystal data and structure refinement for **2r**.

Identification code	cu_20221475_0m
Empirical formula	$C_{34}H_{52}O_6$
Formula weight	556.75
Temperature/K	170.00
Crystal system	monoclinic
Space group	$P2_1$
a/Å	15.3660(5)
b/Å	11.8114(3)
c/Å	34.1656(10)
α/°	90
β/°	95.902(2)
γ/°	90
Volume/Å^3	6168.0(3)
Z	8
ρ_{calc}g/cm^3	1.199
μ/mm^{-1}	0.637
F(000)	2432.0
Crystal size/mm^3	0.12 × 0.08 × 0.05
Radiation	CuKα (λ = 1.54178)
2Θ range for data collection/°	5.2 to 149.39
Index ranges	$-16 \leq h \leq 19$, $-14 \leq k \leq 14$, $-41 \leq l \leq 42$
Reflections collected	56200
Independent reflections	23262 [R_{int} = 0.0599, R_{sigma} = 0.0691]
Data/restraints/parameters	23262/1/1481
Goodness-of-fit on F^2	1.030
Final R indexes [I>=2σ (I)]	R_1 = 0.0818, wR_2 = 0.2368
Final R indexes [all data]	R_1 = 0.0936, wR_2 = 0.2487
Largest diff. peak/hole / e Å^{-3}	0.47/-0.35
Flack parameter	-0.01(9)

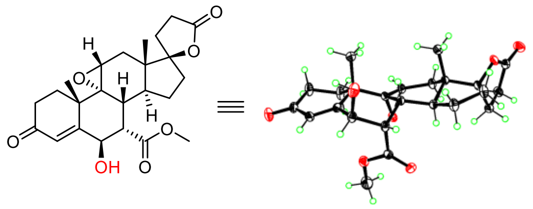

Supplementary Fig. 20. The X-ray crystal structure of **2ag** with thermal ellipsoids at the 40% probability level.

Supplementary Table 11. Crystal data and structure refinement for **2ag**.

Identification code	cu_20211237_0m
Empirical formula	$C_{24}H_{30}O_7$
Formula weight	430.48
Temperature/K	170
Crystal system	orthorhombic
Space group	$C222_1$
a/Å	8.4317(3)
b/Å	12.4549(3)
c/Å	40.4362(10)
α/°	90
β/°	90
γ/°	90
Volume/Å^3	4246.4(2)
Z	8
ρ_{calc}g/cm^3	1.347
μ/mm^{-1}	0.811
F(000)	1840.0
Crystal size/mm^3	0.15 × 0.08 × 0.05
Radiation	CuK_α (λ = 1.54178)
2Θ range for data collection/°	4.37 to 148.696
Index ranges	$-10 \leq h \leq 10$, $-14 \leq k \leq 15$, $-48 \leq l \leq 49$
Reflections collected	19471
Independent reflections	4232 [R_{int} = 0.0406, R_{sigma} = 0.0309]
Data/restraints/parameters	4232/0/284
Goodness-of-fit on F^2	1.047
Final R indexes [I>=2σ (I)]	R_1 = 0.0361, wR_2 = 0.0919
Final R indexes [all data]	R_1 = 0.0392, wR_2 = 0.0953
Largest diff. peak/hole / e Å^{-3}	0.48/-0.18
Flack parameter	0.07(9)

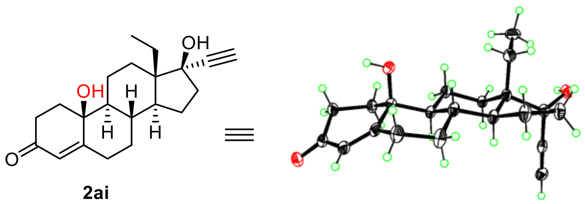

2ai

(CCDC 2182988)

Supplementary Fig. 21. The X-ray crystal structure of **2ai** with thermal ellipsoids at the 40% probability level.

Supplementary Table 12. Crystal data and structure refinement for **2ai**.

Identification code	cu_20211748_0m
Empirical formula	$C_{21}H_{28}O_3$
Formula weight	328.43
Temperature/K	150.0
Crystal system	orthorhombic
Space group	$P2_12_12_1$
a/Å	6.52260(10)
b/Å	13.4030(3)
c/Å	19.9851(5)
α/°	90
β/°	90
γ/°	90
Volume/Å^3	1747.15(6)
Z	4
ρ_{calc}g/cm^3	1.249
μ/mm^{-1}	0.645
F(000)	712.0
Crystal size/mm^3	0.15 × 0.08 × 0.05
Radiation	CuKα (λ = 1.54178)
2Θ range for data collection/°	7.942 to 149.21
Index ranges	$-7 \le h \le 8$, $-16 \le k \le 16$, $-24 \le l \le 24$
Reflections collected	23200
Independent reflections	3566 [R_{int} = 0.0444, R_{sigma} = 0.0259]
Data/restraints/parameters	3566/0/220
Goodness-of-fit on F^2	1.040
Final R indexes [I>=2σ (I)]	R_1 = 0.0328, wR_2 = 0.0789
Final R indexes [all data]	R_1 = 0.0367, wR_2 = 0.0822
Largest diff. peak/hole / e Å^{-3}	0.18/-0.14
Flack parameter	0.06(9)

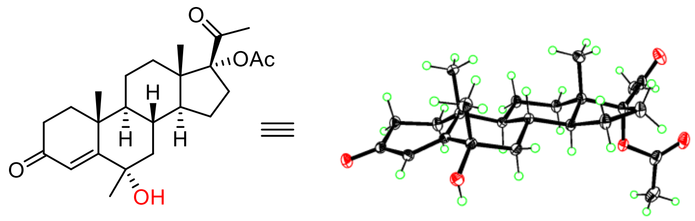

2aj-2

(CCDC 2182989)

Supplementary Fig. 22. The X-ray crystal structure of **2aj-2** with thermal ellipsoids at the 40% probability level.

Supplementary Table 13. Crystal data and structure refinement for **2aj-2**.

Identification code	cu_20211565_0m
Empirical formula	$C_{24}H_{34}O_5$
Formula weight	402.51
Temperature/K	150.0
Crystal system	monoclinic
Space group	$P2_1$
a/Å	6.41550(10)
b/Å	11.2173(2)
c/Å	15.4042(3)
α/°	90
β/°	101.5380(10)
γ/°	90
Volume/Å^3	1086.16(3)
Z	2
ρ_{calc}g/cm^3	1.231
μ/mm^{-1}	0.681
F(000)	436.0
Crystal size/mm^3	0.15 × 0.08 × 0.05
Radiation	CuKα (λ = 1.54178)
2Θ range for data collection/°	5.856 to 149.078
Index ranges	$-7 \leq h \leq 8$, $-14 \leq k \leq 13$, $-18 \leq l \leq 18$
Reflections collected	10794
Independent reflections	4216 [R_{int} = 0.0315, R_{sigma} = 0.0340]
Data/restraints/parameters	4216/1/268
Goodness-of-fit on F^2	1.044
Final R indexes [I>=2σ (I)]	R_1 = 0.0302, wR_2 = 0.0734
Final R indexes [all data]	R_1 = 0.0322, wR_2 = 0.0750
Largest diff. peak/hole / e Å^{-3}	0.20/-0.14
Flack parameter	0.05(8)

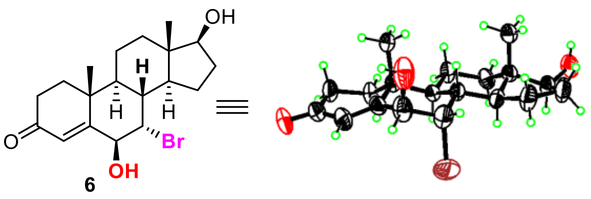

Supplementary Fig. 23. The X-ray crystal structure of **6** with thermal ellipsoids at the 40% probability level.

Supplementary Table 14. Crystal data and structure refinement for **6**.

Identification code	cu_20211147_0m
Empirical formula	$C_{19}H_{27}BrO_3$
Formula weight	383.31
Temperature/K	170
Crystal system	monoclinic
Space group	$P2_1$
a/Å	10.2815(9)
b/Å	6.5838(6)
c/Å	26.449(2)
α/°	90
β/°	90.101(4)
γ/°	90
Volume/Å^3	1790.4(3)
Z	4
ρ_{calc}g/cm^3	1.422
μ/mm^{-1}	3.226
F(000)	800.0
Crystal size/mm^3	0.15 × 0.08 × 0.05
Radiation	CuKα (λ = 1.54178)
2Θ range for data collection/°	6.684 to 149.856
Index ranges	$-12 \le h \le 12, -7 \le k \le 7, -32 \le l \le 33$
Reflections collected	16690
Independent reflections	6751 [R_{int} = 0.0374, R_{sigma} = 0.0496]
Data/restraints/parameters	6751/1/423
Goodness-of-fit on F^2	1.068
Final R indexes [I>=2σ (I)]	R_1 = 0.0573, wR_2 = 0.1486
Final R indexes [all data]	R_1 = 0.0633, wR_2 = 0.1524
Largest diff. peak/hole / e Å^{-3}	0.42/-0.53
Flack parameter	0.125(11)

2.17 Preparation of Starting Materials

Compounds **1a**–**1d**, **1f**–**1h**, **1t**–**1v**, **1ac** and **1ae**–**1ao** were purchased at the highest commercial quality and used without further purification, unless otherwise stated.
Compound **1i** was generously obtained from the Liu lab.
Natural products 7α-OH-neoacolamone (**1k**) and hedyosumin B (**1l**) were isolated from *Hedyosmum orientale*.
Natural products koilodenoid C, koilodenoid F and koilodenoid G (**1p**) were isolated from *Koilodepas hainanense*.
Compound **1s** was generously obtained from the Li lab.

Preparation of compound 1e

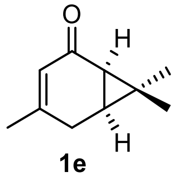

1e

Compound **1e** was prepared by following literature protocol[4]. Spectral data matched reported values therein.

Preparation of compound 1j

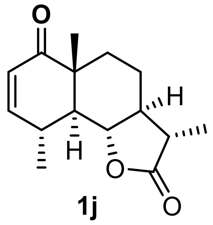

1j

Compound **1j** was prepared by following literature protocol[5]. Spectral data matched reported values therein.

Preparation of compound 1m

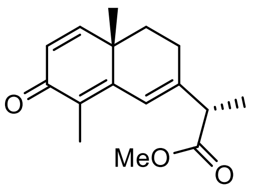

1m

Compound **1m** was prepared by following literature protocol[6]. Spectral data matched reported values therein.

Preparation of compound 1n

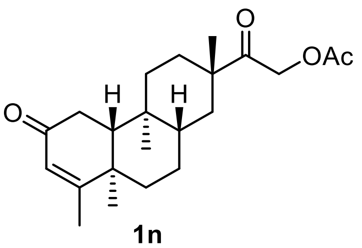

1n

Koilodenoid C (100 mg, 0.31 mmol) was dissolved in 3 mL acetic anhydride. Then 3 mg (0.025 mmol) DMAP and 0.3 mL pyridine were added. The mixture was stirred at room temperature for 2 h. The reaction was quenched with water and the mixture was extracted with ethyl acetate (3 x 10 mL). The combined organic extracts were washed with brine, dried over anhydrous Na_2SO_4, filtered, and concentrated under reduced pressure. The residue was purified by flash chromatography (PE/EtOAc = 1/1) to furnish compound **1n** (92 mg, 81% yield) as a colorless oil.

Characterization data for **1n**:

^{1}H NMR (600 MHz, $CDCl_3$): δ 5.68 (s, 1H), 4.88 – 4.81 (m, 2H), 2.41 (dd, *J* = 17.5, 3.7 Hz, 1H), 2.31 (dd, *J* = 17.5, 14.0 Hz, 1H), 2.14 (s, 3H), 1.86 (d, *J* = 1.4 Hz, 3H), 1.89 – 1.84 (m, 2H), 1.71 (dd, *J* = 14.0, 3.7 Hz, 1H), 1.61 (t, *J* = 13.3 Hz, 1H), 1.58 – 1.55 (m, 1H), 1.52 – 1.39 (m, 3H), 1.38 – 1.27 (m, 2H), 1.25 – 1.22 (m, 1H), 1.22 (s, 3H), 1.10 (s, 3H), 1.07 (dd, *J* = 13.5, 4.4 Hz, 1H), 0.82 (s, 3H).

^{13}C NMR (151 MHz, $CDCl_3$): δ 207.89, 200.21, 172.19, 170.47, 125.63, 64.55, 52.86, 46.24, 41.23, 40.20, 36.65, 36.14, 34.98, 34.48, 33.76, 27.64, 25.35, 20.62, 20.59, 19.06, 18.92, 12.19.

HRMS (ESI): Calculated for $C_{22}H_{33}O_4$ $(M+H)^+$: 361.2373 Found: 361.2367.

Preparation of compound 1o

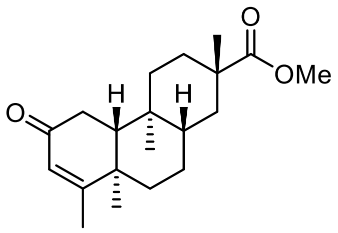

1o

To a flame-dried round bottom equipped with a stir bar under N_2 was added koilodenoid F (60 mg, 0.39 mmol), K_2CO_3 (45 mg, 0.33 mmol), and DMF (4 mL). The reaction mixture was stirred for 30 min at rt. Iodomethane (0.040 mL, 0.64 mmol) was then added, and the reaction mixture was stirred for 2 h. The reaction was quenched with water and the mixture was extracted with DCM (3 x 10 mL). The combined organic extracts were washed with brine, dried over anhydrous Na_2SO_4, filtered, and concentrated under reduced pressure. The residue was purified by flash chromatography (PE/EtOAc = 3/1) to furnish compound **1o** (38 mg, 60% yield) as a white solid.

Characterization data for **1o**:

^{1}H NMR (800 MHz, CDCl$_3$): δ 5.69 (t, *J* = 1.3 Hz, 1H), 3.66 (s, 3H), 2.43 (dd, *J* = 17.5, 3.6 Hz, 1H), 2.32 (dd, *J* = 17.5, 14.2 Hz, 1H), 1.91 (td, *J* = 14.1, 4.5 Hz, 1H), 1.87 (d, *J* = 1.4 Hz, 3H), 1.73 – 1.65 (m, 2H), 1.54 (ddd, *J* = 13.3, 4.6, 2.8 Hz, 1H), 1.51 – 1.42 (m, 3H), 1.34 – 1.26 (m, 4H), 1.21 (s, 3H), 1.11 (s, 3H), 1.06 (td, *J* = 13.6, 4.3 Hz, 1H), 0.85 (d, *J* = 0.9 Hz, 3H).

^{13}C NMR (201 MHz, CDCl$_3$): δ 200.44, 179.25, 172.34, 125.67, 52.95, 51.95, 41.99, 41.46, 40.27, 36.69, 36.24, 36.10, 34.56, 34.09, 28.82, 25.36, 21.49, 19.13, 18.97, 12.26.

HRMS (ESI): Calculated for $C_{20}H_{31}O_3$ $(M+H)^+$: 319.2268 Found: 319.2268.

Preparation of compound 1q

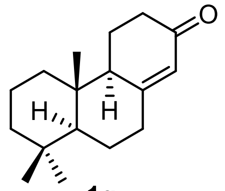

1q

Compound **1q** was prepared by following literature protocol[7]. Spectral data matched reported values therein.

Preparation of compound 1r

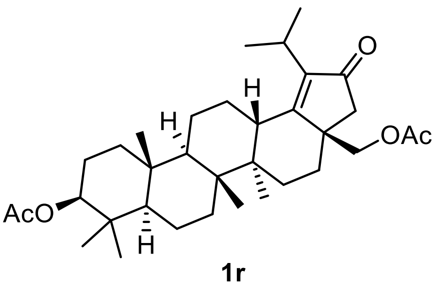

1r

Compound **1r** was prepared by following literature protocol[8]. Spectral data matched reported values therein.

Preparation of compound 1w

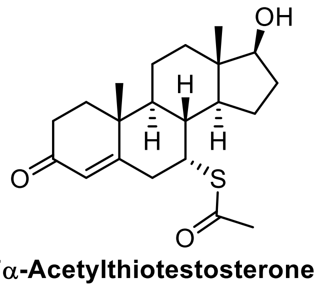

7α-Acetylthiotestosterone (1w)

Compound **1w** was prepared by following literature protocol[9]. Spectral data matched

reported values therein.

Preparation of compound 1x

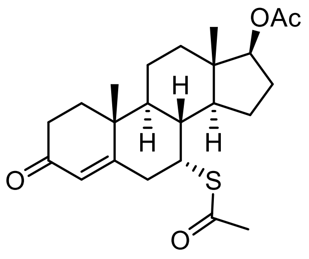

17α-Acetoxy-7β-acetylthio-androst-4-en-3-one (1x)

Compound **1x** was prepared by following literature protocol[10]. Spectral data matched reported values therein.

Preparation of compound 1y

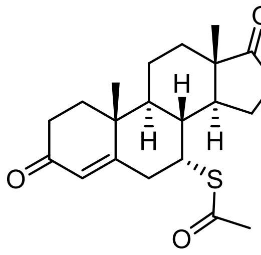

7α-acetylthio-androst-4-ene-3,17-dione (1y)

Compound **1y** was prepared by following literature protocol[11]. Spectral data matched reported values therein.

Preparation of compound 1z

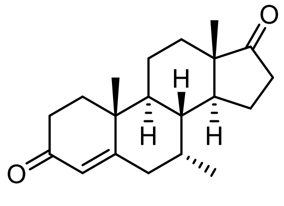

7α-methyl-4-androstene-3,17-dione (1z)

Compound **1z** was prepared by following literature protocol[12]. Spectral data matched reported values therein.

Preparation of compound 1aa

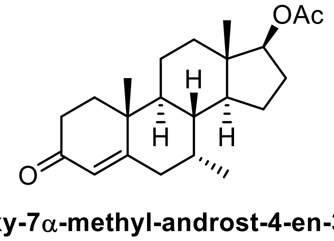

17β-Acetoxy-7α-methyl-androst-4-en-3-one (1aa)

Compound **1aa** was prepared by following literature protocol[13]. Spectral data matched reported values therein.

Preparation of compound 1ab

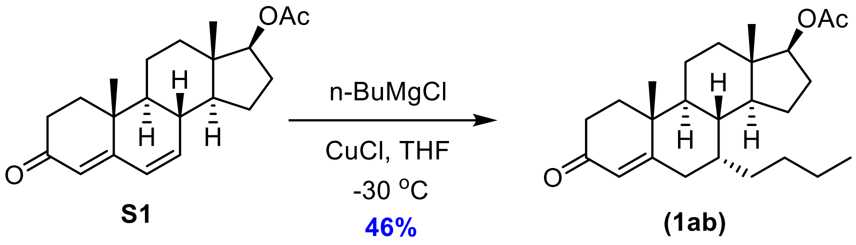

S1 (1ab)

0.64 mL of n-butyl magnesium chloride (2 M) in THF (1.27 mmol, 1.40 equiv) was added dropwise to a solution of the known compound **S1** (300 mg, 0.91 mmol, 1.0 equiv) and CuCl (16 mg, 0.16 mmol, 0.18 equiv) in THF (10 mL) under Ar at -30 °C. Then the reaction mixture was stirred at -30 °C for 4 h before being quenched by 10 mL of 50 vol% sulfuric acid. The mixture was extracted with ethyl acetate (3 × 15 mL). The combined organic extracts were washed with brine, dried over anhydrous Na_2SO_4, filtered, and concentrated under reduced pressure. The residue was purified by flash chromatography (SiO_2, PE/EtOAc = 5/1) to furnish compound **1ab** (160 mg, 46% yield) as a white solid.

Characterization data for **1ab**:

^{1}H NMR (600 MHz, $CDCl_3$): δ 5.72 – 5.68 (m, 1H), 4.58 (dd, *J* = 9.1, 7.9 Hz, 1H), 2.44 – 2.34 (m, 2H), 2.35 – 2.27 (m, 2H), 2.21 – 2.09 (m, 1H), 2.03 – 2.01 (m, 1H), 2.02 (s, 3H), 1.76 – 1.64 (m, 4H), 1.63 – 1.44 (m, 3H), 1.39 (td, *J* = 13.0, 4.0 Hz, 1H), 1.35 – 1.20 (m, 6H), 1.20 – 1.16 (m, 1H), 1.18 (s, 3H), 1.14 – 1.08 (m, 1H),1.05– 1.00 (m, 2H), 0.85 (t, *J* = 7.2 Hz, 3H), 0.81 (s, 3H).

^{13}C NMR (151 MHz, $CDCl_3$): δ 199.37, 171.27, 170.18, 125.91, 82.62, 47.09, 45.89, 42.52, 38.84, 38.71, 36.77, 36.50, 36.45, 36.01, 34.07, 29.75, 27.41, 24.85, 22.98, 22.98, 21.25, 20.84, 18.15, 14.25, 11.94.

HRMS (ESI): Calculated for $C_{25}H_{39}O_3$ $(M+H)^+$: 387.2894; Found: 387.2899.

Preparation of compound 1ad

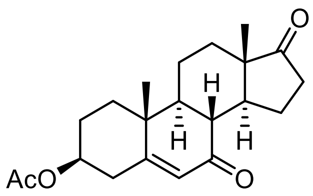

3β-acetoxy-5-androsten-7,17-dione (1ad)

Compound **1ad** was prepared by following literature protocol[14]. Spectral data matched reported values therein.

2.18. Product Characterization

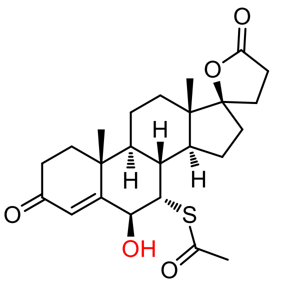

6β-Hydroxyspironolactone (2a)

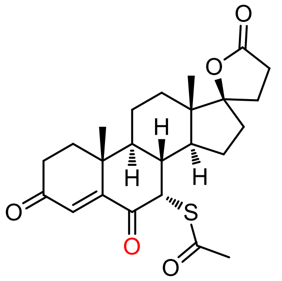

3a

7 mol% Na_2-eosin Y; Irradiation time: 12 h. Purification by flash chromatography on silica (PE/EtOAc = 1/1) to afford a mixture of **2a** and **3a** as a colorless oil (78 mg, 90% total yield, **2a**/**3a** = 20/1, determined by NMR analysis of the mixture of **2a** and **3a**). Purification by HPLC using MeOH/water (80:20, 3.0 mL/min) as eluent to afford pure **2a** as a colorless oil and **3a** as a white solid. Spectral data for **2a** matched reported values[15].

Characterization data for **2a**:
^{1}H NMR (500 MHz, $CDCl_3$): δ 5.80 (s, 1H), 4.17 (d, J = 2.9 Hz, 1H), 3.87 (t, J = 3.1 Hz, 1H), 2.59 – 2.45 (m, 4H), 2.41 – 2.36 (m, 2H), 2.34 (s, 3H), 2.25 (ddd, J = 15.1, 11.5, 3.9 Hz, 1H), 2.04 (ddt, J = 7.4, 4.9, 2.7 Hz, 1H), 1.93 (ddd, J = 12.9, 9.2, 7.2 Hz, 1H), 1.82 (ddd, J = 14.2, 9.2, 4.7 Hz, 1H), 1.72 (dd, J = 14.1, 4.4 Hz, 1H), 1.66 – 1.59 (m, 1H), 1.59 – 1.51 (m, 2H), 1.49 – 1.42 (m, 2H), 1.38 (s, 3H), 1.34 – 1.21 (m, 2H), 1.01 (s, 3H), 0.97 (dd, J = 11.8, 4.6 Hz, 1H)

^{13}C NMR (126 MHz, $CDCl_3$): δ 199.84, 194.34, 176.78, 164.62, 129.17, 95.84, 76.86, 49.31, 49.28, 45.69, 45.48, 38.11, 37.27, 35.35, 34.32, 32.48, 31.42, 31.40, 31.30, 29.36, 22.42, 20.70, 20.48, 14.75.

HRMS (ESI): Calculated for $C_{24}H_{32}NaO_5S$ $(M+Na)^+$: 455.1863; Found: 455.1874.

Characterization data for **3a**:
^{1}H NMR (600 MHz, $CDCl_3$): δ 6.12 (d, J = 1.0 Hz, 1H), 4.39 (d, J = 3.8 Hz, 1H), 2.61 – 2.38 (m, 5H), 2.37 (s, 3H), 2.33 (dd, J = 11.1, 3.9 Hz, 1H), 2.24 (ddd, J = 14.3, 12.2, 3.8 Hz, 1H), 2.15 (ddd, J = 13.3, 5.1, 2.7 Hz, 1H), 2.00 – 1.80 (m, 3H), 1.75 – 1.68 (m, 1H), 1.67 – 1.59 (m, 2H), 1.57 – 1.48 (m, 2H), 1.45 – 1.29 (m, 3H), 1.19 (s, 3H), 0.98 (s, 3H).

^{13}C NMR (151 MHz, $CDCl_3$): δ 198.50, 197.08, 190.76, 176.48, 159.46, 128.02, 95.31, 52.50, 47.53, 45.46, 44.64, 40.40, 37.33, 35.72, 35.12, 33.93, 31.19, 31.16, 30.94, 29.22, 22.31, 20.55, 18.19, 14.68.

HRMS (ESI): Calculated for $C_7H_{11}O_2$ $(M+H)^+$: 431.1887; Found: 431.1890.

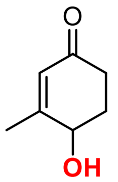

4-Hydroxy-3-methyl-cyclohex-2-en-1-one (2b)

7 mol% Na_2-eosin Y; Irradiation time: 12 h. Purification by flash chromatography on silica (PE/EtOAc = 1/2) to afford pure **2b** as a colorless oil (22 mg, 35% yield). Spectral data for **2b** matched reported values[16].

Characterization data for **2b**:
^{1}H NMR (400 MHz, $CDCl_3$): δ 5.83 (s, 1H), 4.36 (dd, J = 8.4, 4.5 Hz, 1H), 2.56 (m, 1H), 2.41 – 2.21 (m, 2H), 2.05 (s, 3H), 2.03 – 1.94 (m, 1H).

^{13}C NMR (101 MHz, $CDCl_3$): δ 199.36, 163.94, 126.94, 68.74, 34.98, 32.01, 20.78.

HRMS (ESI): Calculated for $C_7H_{11}O_2$ $(M+H)^+$: 127.0754; Found: 127.0750.

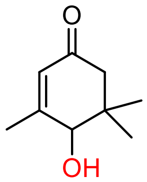

4-Hydroxyisophorone (2c)

7 mol% Na_2-eosin Y; Irradiation time: 12 h. Purification by flash chromatography on silica (PE/EtOAc = 2/1) to afford pure **2c** as a colorless oil (18 mg, 58% yield). Spectral data for **2c** matched reported values[17].

Characterization data for **2c**:
^{1}H NMR (400 MHz, $CDCl_3$): δ 5.86 (s, 1H), 4.03 (s, 1H), 2.40 (d, J = 16.3 Hz, 1H), 2.21 (d, J = 16.3 Hz, 1H), 2.04 (s, 3H), 1.07 (s, 3H), 1.01 (s, 3H).

^{13}C NMR (101 MHz, $CDCl_3$): δ 199.71, 161.97, 126.18, 76.84, 49.01, 38.63, 26.99, 21.66, 21.48.

HRMS (ESI): Calculated for $C_9H_{15}O_2$ $(M+H)^+$: 155.1067; Found: 155.1066.

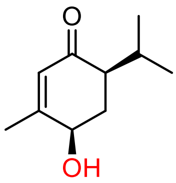

cis-6-Hydroxypiperitone (2d-1)

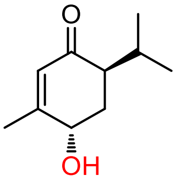

trans-6-Hydroxypiperitone (2d-2)

7 mol% Na_2-eosin Y; Irradiation time: 12 h. Purification by flash chromatography on silica (PE/EtOAc = 2/1) to afford pure **2d-1** as a colorless oil and **2d-2** as a colorless oil (18 mg, 55% total yield, **2d-1**/**2d-2** =1/2, determined by NMR analysis of the mixture of **2d-1** and **2d-2**). Spectral data for **2d-2** matched reported values[18].

Characterization data for **2d-1**:
^{1}H NMR (500 MHz, CDCl$_3$): δ 5.84 (t, J = 1.7 Hz, 1H), 4.62 – 4.22 (m, 1H), 2.53 (ddq, J = 10.2, 7.0, 3.5 Hz, 1H), 2.27 (dt, J = 12.1, 4.6 Hz, 1H), 2.19 (ddd, J = 13.7, 4.2, 3.2 Hz, 1H), 2.03 (d, J = 1.4 Hz, 3H), 1.77 – 1.68 (m, 2H), 0.96 (d, J = 7.1 Hz, 3H), 0.81 (d, J = 6.8 Hz, 3H).

^{13}C NMR (126 MHz, CDCl$_3$): δ 199.68, 162.97, 127.60, 70.65, 51.17, 32.81, 25.75, 20.50, 19.55, 17.63.

HRMS (ESI): Calculated for $C_{10}H_{17}O_2$ $(M+H)^+$: 169.1223; Found: 169.1225.

Characterization data for **2d-2**:

^{1}H NMR (500 MHz, CDCl$_3$): δ 5.80 (d, J = 1.6 Hz, 1H), 4.35 (t, J = 4.9 Hz, 1H), 2.37 (ddd, J = 9.0, 5.9, 4.7 Hz, 1H), 2.30 – 2.20 (m, 1H), 2.15 (ddd, J = 13.4, 9.0, 4.2 Hz, 1H), 2.08 – 2.03 (m, 1H), 2.03 (d, J = 1.7 Hz, 3H), 0.94 (d, J = 6.9 Hz, 3H), 0.89 (d, J = 6.8 Hz, 3H).

^{13}C NMR (126 MHz, CDCl$_3$): δ 200.81, 159.01, 127.55, 67.50, 48.50, 32.55, 26.49, 21.04, 20.80, 19.20.

HRMS (ESI): Calculated for $C_{10}H_{17}O_2$ $(M+H)^+$: 169.1223; Found: 169.1218.

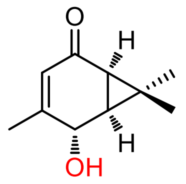

trans-3-Caren-5-on-2-ol (2e)

7 mol% Na_2-eosin Y; Irradiation time: 12 h. Purification by flash chromatography on silica (PE/EtOAc = 1/1) to afford pure **2e** as a colorless oil (16 mg, 48% yield).

Characterization data for **2e**:
^{1}H NMR (400 MHz, CDCl$_3$): δ 5.83 (d, J = 1.3 Hz, 1H), 4.12 (s, 1H), 2.03 (d, J = 1.4 Hz, 3H), 1.84 – 1.63 (m, 2H), 1.21 (s, 3H), 1.02 (s, 3H).

^{13}C NMR (101 MHz, CDCl$_3$): δ 196.10, 158.17, 127.80, 65.57, 35.66, 33.49, 28.90, 25.47, 21.34, 14.57.

HRMS (ESI): Calculated for $C_{10}H_{15}O_2$ $(M+H)^+$: 167.1067; Found: 167.1069.

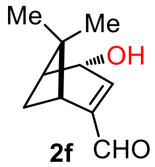

2f

7 mol% Na_2-eosin Y; Irradiation time: 12 h. Purification by flash chromatography on silica (PE/EtOAc = 5/1 to 1/1) to afford starting material **1f** (15 mg, 50%) and pure **2f** as a colorless oil (9 mg, 27% yield).

Characterization data for **2f**:

^{1}H NMR (400 MHz, CDCl$_3$): δ 9.53 (s, 1H), 6.78 – 6.38 (m, 1H), 4.58 (t, J = 3.1 Hz, 1H), 2.88 (td, J = 5.6, 1.4 Hz, 1H), 2.40 (dt, J = 9.7, 5.5 Hz, 1H), 2.27 (tdd, J = 5.6, 3.2, 1.9 Hz, 1H), 1.39 (s, 3H), 1.22 (d, J = 9.7 Hz, 1H), 0.78 (s, 3H).

^{13}C NMR (101 MHz, CDCl$_3$): δ 191.97, 151.87, 144.72, 70.12, 47.28, 46.39, 39.15, 28.58, 26.28, 20.61.

HRMS (ESI): Calculated for $C_{10}H_{15}O_2$ $(M+H)^+$: 167.1067; Found: 167.1070.

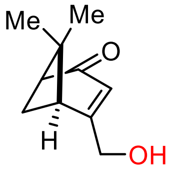

10-Hydroxyverbenone (2g)

7 mol% Na$_2$-eosin Y; Irradiation time: 15 h; Acetone (10 mL) as solvent. Purification by flash chromatography on silica (PE/EtOAc = 1/1) to afford pure **2g** as a colorless oil (3 mg, 9% yield) and starting material **1g** (26 mg, 87%). Spectral data for **2g** matched reported values[19].

Characterization data for **2g:**

^{1}H NMR (400 MHz, CDCl$_3$): δ 5.97 (t, J = 1.8 Hz, 1H), 4.37 (dd, J = 17.7, 2.0 Hz, 1H), 4.26 (dd, J = 17.7, 2.0 Hz, 1H), 2.85 (dt, J = 9.2, 5.5 Hz, 1H), 2.69 (td, J = 5.9, 1.8 Hz, 1H), 2.41 (td, J = 5.9, 1.4 Hz, 1H), 2.11 (d, J = 9.2 Hz, 1H), 1.51 (s, 3H), 1.01 (s, 3H).

^{13}C NMR (101 MHz, CDCl$_3$): δ 203.52, 171.44, 119.39, 64.23, 58.43, 54.10, 45.35, 41.12, 26.62, 22.27.

HRMS (ESI): Calculated for $C_{10}H_{14}NaO_2$ $(M+Na)^+$: 189.0886; Found: 189.0881.

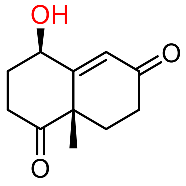

2h-1

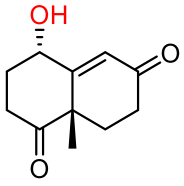

2h-2

7 mol% Na$_2$-eosin Y; Irradiation time: 12 h. Purification by flash chromatography on silica (PE/EtOAc = 2/3) to afford pure **2h-1** as a colorless oil and **2h-2** as a colorless oil (10 mg, 26% total yield, **2h-1**/**2h-2** =1.2/1, determined by NMR analysis of the mixture of **2h-1** and **2h-2**) and starting material **1h** (21 mg, 58%).

Characterization data for **2h-1**:

^{1}H NMR (500 MHz, CDCl$_3$): δ 5.92 (s, 1H), 4.54 (t, J = 3.3 Hz, 1H), 3.17 (ddd, J = 15.5, 13.5, 5.9 Hz, 1H), 2.56 – 2.47 (m, 2H), 2.37 (ddd, J = 15.5, 4.9, 3.3 Hz, 1H),

2.30 (ddt, J = 14.4, 6.0, 3.5 Hz, 1H), 2.15 (ddd, J = 14.0, 4.9, 3.0 Hz, 1H), 2.06 (td, J = 13.6, 6.2 Hz, 1H), 2.01 – 1.91 (m, 1H), 1.63 (s, 3H).

^{13}C NMR (126 MHz, CDCl$_3$): δ 211.38, 199.38, 165.06, 127.00, 71.40, 49.65, 33.93, 32.35, 31.15, 30.03, 25.51.

HRMS (ESI): Calculated for $C_{11}H_{15}O_3$ $(M+H)^+$: 195.1016; Found: 195.1011.

Characterization data for **2h-2**:

^{1}H NMR (500 MHz, CDCl$_3$): δ 6.26 (d, J = 1.9 Hz, 1H), 4.73 (ddd, J = 11.9, 5.5, 1.9 Hz, 1H), 2.79 (ddd, J = 16.1, 13.5, 6.3 Hz, 1H), 2.57 (ddd, J = 16.0, 5.2, 3.0 Hz, 1H), 2.50 – 2.43 (m, 2H), 2.43 – 2.36 (m, 1H), 2.20 – 2.06 (m, 2H), 1.86 – 1.66 (m, 1H), 1.45 (s, 3H).

^{13}C NMR (126 MHz, CDCl$_3$): δ 209.91, 198.65, 166.87, 122.06, 67.75, 49.76, 35.59, 33.70, 31.63, 30.63, 24.21.

HRMS (ESI): Calculated for $C_{11}H_{15}O_3$ $(M+H)^+$: 195.1016; Found: 195.1012.

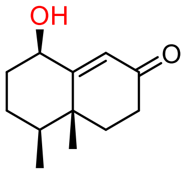

2i-1

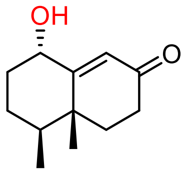

2i-2

7 mol% Na$_2$-eosin Y; On 0.18 mmol scale; Irradiation time: 5 h. Purification by flash chromatography on silica (PE/EtOAc = 1/1) to afford **2i-1** as a white solid (15 mg, 43% yield) and **2i-2** as a colorless oil (6 mg, 17% yield). Spectral data for **2i-1** and **2i-2** matched reported values[20].

Characterization data for **2i-1**:

^{1}H NMR (600 MHz, CDCl$_3$): δ 5.83 (s, 1H), 4.33 (s, 1H), 2.54 (ddd, J = 17.4, 15.0, 5.0 Hz, 1H), 2.46 – 2.34 (m, 1H), 2.07 – 1.95 (m, 2H), 1.92 – 1.83 (m, 1H), 1.77 – 1.63 (m, 2H), 1.47 – 1.37 (m, 2H), 1.30 (s, 3H), 0.95 (d, J = 6.6 Hz, 3H).

^{13}C NMR (151 MHz, CDCl$_3$): δ 200.67, 168.45, 126.77, 73.59, 43.22, 38.54, 37.08, 34.47, 33.02, 25.00, 18.19, 15.37.

HRMS (ESI): Calculated for $C_{12}H_{19}O_2$ $(M+H)^+$: 195.1380; Found: 195.1374.

Characterization data for **2i-2**:

^{1}H NMR (500 MHz, CDCl$_3$): δ 6.18 (d, J = 2.7 Hz, 1H), 4.30 (ddd, J = 11.7, 5.6, 1.9 Hz, 1H), 2.49 – 2.30 (m, 2H), 2.26 – 2.14 (m, 1H), 2.07 – 1.96 (m, 1H), 1.78 (td, J = 13.7, 4.9 Hz, 1H), 1.66 – 1.59 (m, 1H), 1.56 – 1.43 (m, 3H), 1.11 (s, 3H), 0.92 (d, J = 6.5 Hz, 3H).

^{13}C NMR (126 MHz, CDCl$_3$): δ 199.70, 171.49, 120.12, 69.29, 42.98, 39.46, 36.32, 36.18, 33.88, 28.85, 17.13, 15.01.

HRMS (ESI): Calculated for $C_{12}H_{19}O_2$ $(M+H)^+$: 195.1380; Found: 195.1377.

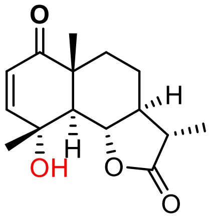

Vulgarin (2j)

7 mol% Na_2-eosin Y; Irradiation time: 12 h. Purification by flash chromatography on silica (PE/EtOAc = 1/1) to afford pure **2j** as a white solid (44 mg, 69% yield). Spectral data for **2j** matched reported values[21].

Characterization data for **2j**:

^{1}H NMR (400 MHz, CDCl$_3$): δ 6.59 (d, J = 10.4 Hz, 1H), 5.87 (d, J = 10.3 Hz, 1H), 4.15 (dd, J = 11.5, 10.4 Hz, 1H), 2.41 (d, J = 11.5 Hz, 1H), 2.34 (dq, J = 13.6, 6.9 Hz, 1H), 2.07 – 1.92 (m, 2H), 1.67 (tdd, J = 12.3, 10.4, 3.4 Hz, 1H), 1.59 – 1.45 (m, 2H), 1.54 (s, 3H), 1.24 (d, J = 6.9 Hz, 3H), 1.20 (s, 3H).

^{13}C NMR (126 MHz, CDCl$_3$): δ 201.78, 178.35, 151.86, 125.70, 79.66, 70.17, 54.67, 52.47, 46.36, 40.64, 34.31, 23.88, 22.78, 19.84, 12.54.

HRMS (ESI): Calculated for $C_{15}H_{20}NaO_4$ $(M+Na)^+$: 287.1254; Found: 287.1256.

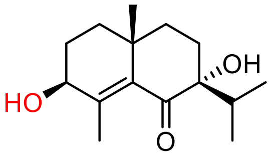

2k-1

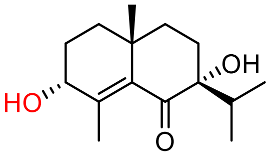

2k-2

On 0.08 mmol scale; 4 mol% Na_2-eosin Y; Irradiation time: 12 h. Purification by flash chromatography on silica (PE/EtOAc = 5/4) to afford **2k-1** as a colorless oil and **2k-2** as a colorless oil (7 mg, 35% total yield, **2k-1/2k-2** =1/1.1, determined by NMR analysis of the mixture of **2k-1** and **2k-2**).

Characterization data for **2k-1**:

^{1}H NMR (500 MHz, CDCl$_3$): δ 4.11 (dd, J = 8.2, 6.6 Hz, 1H), 2.29 – 2.21 (m, 1H), 2.03 – 1.98 (m, 1H), 1.96 – 1.87 (m, 2H), 1.81 (d, J = 1.0 Hz, 3H), 1.70 – 1.62 (m, 4H), 1.52 – 1.45 (m, 1H), 1.02 (s, 3H), 0.97 (d, J = 6.9 Hz, 3H), 0.93 (d, J = 6.7 Hz, 3H).

^{13}C NMR (126 MHz, CDCl$_3$): δ 203.83, 141.20, 140.25, 79.31, 71.61, 38.47, 36.15, 35.46, 32.11, 28.62, 27.09, 24.85, 18.07, 17.27, 16.20.

HRMS (ESI): Calculated for $C_{15}H_{24}NaO_3$ $(M+Na)^+$: 275.1618 Found: 275.1616.

Characterization data for **2k-2**:

^{1}H NMR (400 MHz, CDCl$_3$): δ 3.95 (t, J = 2.6 Hz, 1H), 2.29 – 2.16 (m, 1H), 1.97 – 1.85 (m, 2H), 1.83 (s, 3H), 1.79 – 1.74 (m, 3H), 1.61 – 1.52 (m, 1H), 1.48 – 1.39 (m, 1H), 1.07 (t, J = 6.7 Hz, 1H), 0.97 (d, J = 6.9 Hz, 3H), 0.94 (s, 3H), 0.93 (d, J = 6.6 Hz, 3H).

^{13}C NMR (126 MHz, CDCl$_3$): δ 204.66, 141.23, 138.45, 79.78, 68.45, 38.39, 35.26, 32.34, 31.83, 27.36, 27.27, 23.41, 18.64, 17.98, 16.41.

HRMS (ESI): Calculated for $C_{15}H_{24}NaO_3$ $(M+Na)^+$: 275.1618 Found: 275.1619.

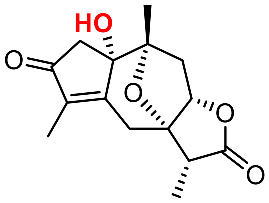

1α-Hydroxyhedyosumin B (2l)

8 mol% Na_2-eosin Y; Irradiation time: 15 h. Purification by flash chromatography on silica (PE/EtOAc = 2/3) to afford pure **2l** as a white solid (48 mg, 86% yield).

Characterization data for **2l:**

^{1}H NMR (600 MHz, CDCl$_3$): δ 4.41 (dd, J = 6.8, 1.8 Hz, 1H), 2.96 (d, J = 14.9 Hz, 1H), 2.71 – 2.54 (m, 2H), 2.44 (d, J = 18.8 Hz, 1H), 2.22 (d, J = 18.9 Hz, 1H), 2.01 (dd, J = 15.0, 6.7 Hz, 1H), 1.94 (dd, J = 15.0, 1.9 Hz, 1H), 1.71 (d, J = 1.8 Hz, 3H), 1.44 (s, 3H), 1.34 (d, J = 7.3 Hz, 3H).

^{13}C NMR (151 MHz, CDCl$_3$): δ 204.76, 176.58, 164.25, 138.33, 88.82, 88.58, 84.63, 77.94, 43.50, 42.33, 40.39, 31.24, 19.89, 8.29, 8.01.

HRMS (ESI): Calculated for $C_{15}H_{19}O_5$ $(M+H)^+$: 279.1227; Found: 279.1229.

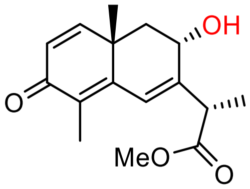

2m

On 2.04 mmol scale; 7 mol% Na_2-eosin Y; MeCN (80 mL); MeOH (30 mL); Irradiation time: 18 h. Purification by flash chromatography on silica (PE/EtOAc = 2/1) to afford pure **2m** as a white solid (168 mg, 30% yield) and starting material **1m** (174 mg, 33%).

Characterization data for **2m:**

^{1}H NMR (400 MHz, CDCl$_3$): δ 6.75 (d, J = 9.8 Hz, 1H), 6.57 (s, 1H), 6.23 (d, J = 9.8 Hz, 1H), 4.56 (t, J = 8.0 Hz, 1H), 3.69 (s, 3H), 3.68 (q, J = 8.1 Hz, 1H), 2.24 (dd, J = 12.5, 6.0 Hz, 1H), 1.94 (s, 3H), 1.50 (dd, J = 12.8, 2.0 Hz, 1H), 1.45 (d, J = 7.3 Hz,

3H), 1.13 (s, 3H).

^{13}C NMR (100 MHz, CDCl$_3$): δ 186.60, 175.20, 154.72, 152.66, 146.54, 129.84, 127.19, 123.46, 65.97, 52.38, 42.37, 42.24, 40.10, 25.79, 15.57, 10.36.

HRMS (ESI): Calculated for $C_{16}H_{21}O_4$ $(M+H)^+$: 277.1434 Found: 277.1435.

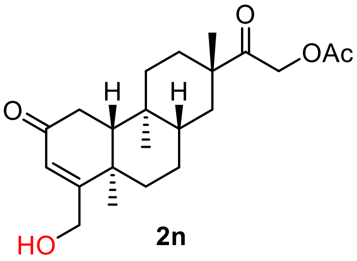

2n

7 mol% Na$_2$-eosin Y; Irradiation time: 12 h. Purification by flash chromatography on silica (PE/EtOAc = 2/3) to afford pure **2n** a white solid (15 mg, 20% yield) and starting material **1n** (42 mg, 58%).

Characterization data for **2n:**

^{1}H NMR (500 MHz, CDCl$_3$): *δ* 6.08 (s, 1H), 4.90 – 4.83 (m, 2H), 4.41 – 4.32 (m, 2H), 2.47 (dd, *J* = 17.7, 3.9 Hz, 1H), 2.41 – 2.30 (m, 1H), 2.22 (t, 7.7Hz, 0.43H), 2.16 (s, 3H), 1.95 – 1.88 (m, 1H), 1.81 – 1.74 (m, 2H), 1.69 – 1.55 (m, 4H), 1.51 – 1.42 (m, 3H), 1.24 (s, 3H), 1.19 (s, 3H), 1.15 – 1.06 (m, 2H), 0.85 (s, 3H).

^{13}C NMR (126 MHz, CDCl$_3$): *δ* 207.91, 200.28, 173.36, 170.60, 121.41, 64.61, 60.46, 53.02, 46.31, 41.31, 39.51, 36.73, 35.14, 34.97, 34.77, 33.84, 27.66, 25.15, 20.70, 20.66, 20.15, 12.28.

HRMS (ESI): Calculated for $C_{22}H_{33}O_5$ $(M+H)^+$: 377.2323 Found: 377.2324.

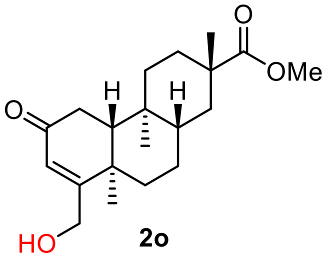

2o

On 0.12 mmol scale; 7 mol% Na$_2$-eosin Y; Irradiation time: 12 h. Purification by flash chromatography on silica (PE/EtOAc = 1/1) to afford pure **2o** as a white solid (10 mg, 25% yield) and starting material **1o** (18 mg, 47%).

Characterization data for **2o:**

^{1}H NMR (800 MHz, CDCl$_3$): *δ* 6.08 (d, *J* = 1.9 Hz, 1H), 4.47 – 4.28 (m, 2H), 3.67 (s, 3H), 2.48 (dd, *J* = 17.7, 3.7 Hz, 1H), 2.38 (dd, *J* = 17.6, 14.1 Hz, 1H), 2.22 (t, *J* = 7.6 Hz, 0.57H), 1.92 (td, *J* = 14.1, 4.5 Hz, 1H), 1.83 – 1.74 (m, 2H), 1.69 (t, *J* = 13.2 Hz, 1H), 1.57 – 1.53 (m, 1H), 1.52 – 1.46 (m, 3H), 1.36 – 1.30 (m, 3H), 1.22 (s, 3H), 1.20

(s, 3H), 1.08 (td, J = 13.6, 4.3 Hz, 1H), 0.87 (s, 3H).

^{13}C NMR (201 MHz, $CDCl_3$): δ 200.38, 179.23, 173.34, 121.40, 60.50, 53.08, 52.00, 42.01, 41.50, 39.54, 36.72, 36.04, 35.20, 34.81, 34.13, 28.78, 25.12, 21.53, 20.18, 12.30.

HRMS (ESI): Calculated for $C_{20}H_{31}O_4$ $(M+H)^+$: 335.2217 Found: 335.2214.

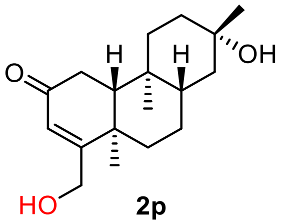

2p

7 mol% Na_2-eosin Y; Irradiation time: 12 h. Purification by flash chromatography on silica (PE/EtOAc = 1/3) to afford pure **2p** as a white solid (12 mg, 21% yield) and starting material **1p** (35 mg, 64%).

Characterization data for **2p:**

^{1}H NMR (600 MHz, CD_3OD): δ 6.03 (s, 1H), 4.29 (d, J = 1.9 Hz, 2H), 2.58 – 2.33 (m, 2H), 2.17 (t, 7.6Hz, 0.55H), 1.87 – 1.80 (m, 1H), 1.75 (dd, J = 13.6, 4.2 Hz, 1H), 1.68 (dd, J = 13.6, 4.6 Hz, 1H), 1.63 – 1.56 (m, 2H), 1.54 – 1.48 (m, 4H), 1.34 – 1.31 (m, 2H), 1.21 (s, 3H), 1.21 (s, 3H), 1.01 (td, J = 13.7, 4.1 Hz, 1H), 0.93 (s, 3H).

^{13}C NMR (151 MHz, CD_3OD): δ 203.22, 178.17, 121.41, 71.64, 60.32, 54.38, 45.47, 43.27, 40.75, 37.78, 37.59, 36.29, 36.03, 35.76, 26.72, 26.27, 20.17, 12.74.

HRMS (ESI): Calculated for $C_{18}H_{29}O_3$ $(M+H)^+$: 293.2111 Found: 293.2111.

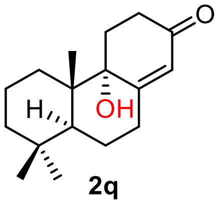

2q

On 0.085 mmol scale; 6 mol% Na_2-eosin Y; Irradiation time: 9 h. Purification by flash chromatography on silica (PE/EtOAc = 5/3) to afford pure **2q** as a white solid (14 mg, 64% yield).

Characterization data for **2q:**

^{1}H NMR (400 MHz, $CDCl_3$): δ 5.84 (d, J = 2.3 Hz, 1H), 2.67 – 2.50 (m, 2H), 2.47 – 2.28 (m, 2H), 2.18 (td, J = 14.1, 4.6 Hz, 1H), 1.93 – 1.80 (m, 2H), 1.75 – 1.66 (m, 2H), 1.60 – 1.51 (m, 2H), 1.48 – 1.41 (m, 2H), 1.33 – 1.26 (m, 1H), 1.23 – 1.16 (m, 1H), 0.95 (s, 3H), 0.92 (s, 3H), 0.89 (s, 3H).

^{13}C NMR (101 MHz, $CDCl_3$): δ 199.40, 163.05, 126.74, 74.84, 44.53, 42.46, 41.46, 33.95, 33.89, 33.50, 32.38, 31.86, 28.22, 22.31, 21.87, 18.52, 18.19.

HRMS (ESI): Calculated for $C_{17}H_{27}O_2$ $(M+H)^+$: 263.2006 Found: 263.2003.

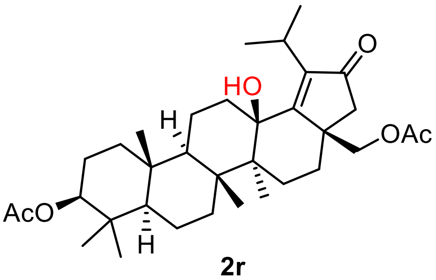

2r

7 mol% Na_2-eosin Y; Irradiation time: 12 h. Purification by flash chromatography on silica (PE/EtOAc = 5/1) to afford pure **2r** as a white solid (20 mg, 18% yield) and starting material **1r** (78 mg, 72%).

Characterization data for **2r**:

^{1}H NMR (500 MHz, $CDCl_3$): δ 5.12 (d, J = 10.5 Hz, 1H), 4.48 (dd, J = 10.9, 5.6 Hz, 1H), 3.98 (d, J = 10.4 Hz, 1H), 3.24 (p, J = 6.9 Hz, 1H), 2.85 (s, 1H), 2.45 (d, J = 17.4 Hz, 1H), 2.37 (td, J = 13.8, 4.4 Hz, 1H), 2.09 – 2.04 (m, 1H), 2.04 (s, 3H), 2.00 (d, J = 17.5 Hz, 1H), 1.95 (s, 3H), 1.86 (ddd, J = 14.1, 4.5, 2.6 Hz, 1H), 1.78 (dq, J = 12.7, 3.8 Hz, 2H), 1.71 – 1.60 (m, 4H), 1.54 – 1.33 (m, 6H), 1.27 (s, 3H), 1.20 – 1.14 (m, 6H), 1.14 – 1.03 (m, 2H), 0.94 (d, J = 1.6 Hz, 6H), 0.85 (s, 3H), 0.84 (s, 3H), 0.81 (dd, J = 10.5, 3.7 Hz, 1H).

^{13}C NMR (126 MHz, $CDCl_3$): δ 207.50, 171.60, 170.58, 167.79, 145.30, 80.41, 79.10, 71.53, 55.21, 51.31, 50.37, 45.60, 45.57, 41.59, 38.29, 37.40, 36.83, 36.70, 34.68, 31.82, 27.41, 25.29, 23.63, 23.24, 20.87, 20.49, 19.90, 19.08, 18.93, 17.67, 17.32, 16.80, 16.42, 15.98.

HRMS (ESI): Calculated for $C_{34}H_{52}NaO_6$ $(M+ Na)^+$: 579.3656 Found: 579.3655.

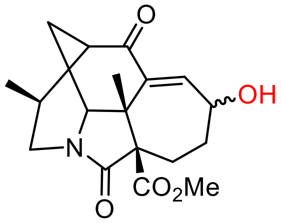

2s

On 0.063 mmol scale; 5 mol% Na_2-eosin Y; Irradiation time: 8 h. Purification by flash chromatography on silica (PE/EtOAc = 1/3) to afford **2s** (mixture of a pair of diastereomers) as a white solid (3 mg, 14% yield, *d.r.* =7.1:1).

Characterization data for **2s**:

^{1}H NMR (600 MHz, CD_3OD; major isomer): δ 7.12 (d, J = 2.8 Hz, 1H), 4.87 – 4.83 (m, 1H), 3.97 – 3.94 (m, 2H), 3.75 (s, 3H), 2.80 – 2.74 (m, 1H), 2.61 (dd, J = 13.6, 10.3 Hz, 1H), 2.24 – 2.23 (m, 1H), 2.21 – 2.01 (m, 4H), 1.74 – 1.65 (m, 2H), 1.39 (s, 3H), 1.09 (d, J = 6.9 Hz, 3H).

^{13}C NMR (151 MHz, CD_3OD; major isomer): δ 202.05, 174.74, 172.37, 154.00, 135.47, 69.54, 61.53, 59.39, 52.92, 52.27, 47.01, 41.83, 38.99, 29.62, 28.17, 19.54, 18.43, 17.78.

HRMS (ESI): Calculated for $C_{18}H_{24}NO5$ $(M+H)^+$: 334.1649 Found: 334.1651.

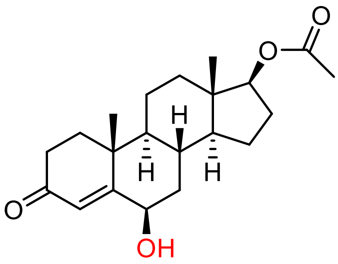

6β-Hydroxytestosterone acetate (2t)

7 mol% Na_2-eosin Y; Irradiation time: 12 h. Purification by flash chromatography on silica (PE/EtOAc = 2/1) to afford pure **2t** as a white solid (28 mg, 41% yield).

Characterization data for **2t:**

^{1}H NMR (600 MHz, $CDCl_3$): δ 5.80 (d, J = 1.0 Hz, 1H), 4.60 (dd, J = 9.2, 7.8 Hz, 1H), 4.34 (t, J = 2.9 Hz, 1H), 2.50 (ddd, J = 17.2, 15.0, 5.0 Hz, 1H), 2.41 – 2.33 (m, 1H), 2.22 – 2.11 (m, 1H), 2.04 (s, 3H), 2.02 – 1.97 (m, 2H), 1.79 (ddd, J = 12.8, 4.1, 2.8 Hz, 1H), 1.74 – 1.62 (m, 2H), 1.59 – 1.38 (m, 4H), 1.37 (s, 3H), 1.28 – 1.13 (m, 3H), 1.05 (ddd, J = 12.4, 10.9, 7.2 Hz, 1H), 0.96 – 0.89 (m, 1H), 0.85 (s, 3H).

^{13}C NMR (151 MHz, $CDCl_3$): δ 200.55, 171.39, 168.38, 126.50, 82.65, 73.04, 53.62, 50.36, 42.67, 38.13, 38.08, 37.18, 36.71, 34.32, 29.67, 27.58, 23.54, 21.31, 20.60, 19.67, 12.22.

HRMS (ESI): Calculated for $C_{21}H_{31}O_4$ $(M+H)^+$: 347.2217; Found: 347.2213.

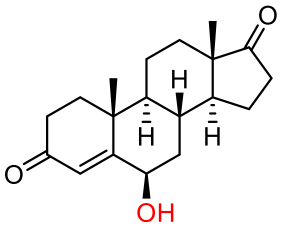

6β-Hydroxyandrostenedione (2u)

7 mol% Na_2-eosin Y; Irradiation time: 12 h. Purification by flash chromatography on silica (PE/EtOAc = 1/1) to afford pure **2u** as a white solid (20 mg, 33% yield). Spectral data for **2u** matched reported values[22].

Characterization data for **2u:**

^{1}H NMR (400 MHz, $CDCl_3$): δ 5.83 (d, J = 0.9 Hz, 1H), 4.40 (t, J = 2.9 Hz, 1H), 2.63 – 2.32 (m, 3H), 2.24 – 1.94 (m, 5H), 1.88 (ddd, J = 13.0, 4.1, 2.7 Hz, 1H), 1.77 – 1.58 (m, 3H), 1.57 – 1.45 (m, 1H), 1.40 (s, 3H), 1.34 – 1.22 (m, 3H), 1.02 – 0.96 (m, 1H), 0.94 (s, 3H).

^{13}C NMR (126 MHz, $CDCl_3$): δ 220.60, 200.29, 167.85, 126.68, 72.97, 53.80, 51.06, 47.78, 38.20, 37.37, 37.23, 35.90, 34.32, 31.42, 29.56, 21.86, 20.42, 19.71, 13.92.

HRMS (ESI): Calculated for $C_{19}H_{27}O_3$ $(M+H)^+$: 303.1955; Found: 303.1948.

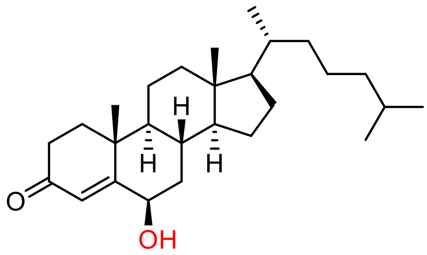

6β-Hydroxycholest-4-en-3-one (2v)

7 mol% Na_2-eosin Y; Irradiation time: 12 h. Purification by flash chromatography on silica (PE/EtOAc = 2/1) to afford pure **2v** as a white solid (27 mg, 34% yield). Spectral data for **2v** matched reported values[23].

Characterization data for **2v:**

^{1}H NMR (500 MHz, $CDCl_3$): δ 5.81 (d, J = 0.9 Hz, 1H), 4.34 (t, J = 2.8 Hz, 1H), 2.52 (ddd, J = 17.3, 15.0, 5.0 Hz, 1H), 2.38 (dddd, J = 17.4, 4.3, 2.8, 1.1 Hz, 1H), 2.08 – 2.00 (m, 2H), 2.00 – 1.90 (m, 1H), 1.89 – 1.80 (m, 1H), 1.75 – 1.66 (m, 1H), 1.66 – 1.58 (m, 3H), 1.55 – 1.47 (m, 3H), 1.37 (s, 3H), 1.36 – 1.29 (m, 3H), 1.27 – 1.23 (m, 3H), 1.19 – 1.08 (m, 5H), 1.05 – 0.98 (m, 2H), 0.91 (d, J = 6.5 Hz, 3H), 0.87 (d, J = 2.3 Hz, 3H), 0.86 (d, J = 2.3 Hz, 3H), 0.74 (s, 3H).

^{13}C NMR (126 MHz, $CDCl_3$): δ 200.59, 168.64, 126.46, 73.44, 56.32, 56.04, 53.78, 42.67, 39.77, 39.65, 38.72, 38.15, 37.25, 36.28, 35.90, 34.42, 29.88, 28.31, 28.16, 24.30, 23.98, 22.96, 22.70, 21.13, 19.66, 18.82, 12.17.

HRMS (ESI): Calculated for $C_{27}H_{45}O_2$ $(M+H)^+$: 401.3414; Found: 401.3407.

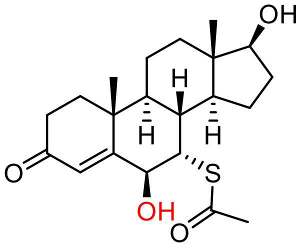

6β-Hydroxy-7α-acetylthiotestosterone (2w)

7 mol% Na_2-eosin Y; Irradiation time: 12 h. Purification by flash chromatography on silica (PE/EtOAc = 1/1) to afford pure **2w** as a white solid (35 mg, 49% yield).

Characterization data for **2w:**

^{1}H NMR (500 MHz, CD_3OD): δ 5.79 (d, J = 0.9 Hz, 1H), 4.12 (d, J = 3.0 Hz, 1H), 3.92 (t, J = 3.2 Hz, 1H), 3.63 (t, J = 8.6 Hz, 1H), 2.68 – 2.48 (m, 2H), 2.40 – 2.35 (m, 1H), 2.37 (s, 3H), 2.13 (ddd, J = 13.2, 5.0, 2.7 Hz, 1H), 2.06 – 1.94 (m, 1H), 1.91

(ddd, J = 12.6, 4.1, 2.8 Hz, 1H), 1.74 (ddd, J = 15.0, 13.3, 4.4 Hz, 1H), 1.66 (tdd, J = 11.9, 6.0, 3.7 Hz, 1H), 1.63 – 1.47 (m, 3H), 1.45 (s, 3H), 1.42 – 1.31 (m, 2H), 1.14 – 1.01 (m, 2H), 0.87 (s, 3H).

^{13}C NMR (126 MHz, CD_3OD): δ 202.34, 195.58, 168.51, 129.11, 82.19, 77.92, 51.12, 50.48, 47.88, 43.99, 39.35, 38.33, 37.33, 35.06, 33.42, 31.06, 30.39, 23.59, 21.84, 20.67, 11.58.

HRMS (ESI): Calculated for $C_{21}H_{30}NaO_4S$ $(M+Na)^+$: 401.1757; Found: 401.1758.

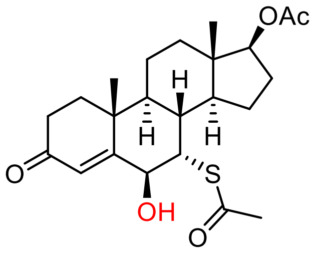

6β-Hydroxy-7α-acetylthiotestosterone acetate (2x)

7 mol% Na_2-eosin Y; Irradiation time: 12 h. Purification by flash chromatography on silica (PE/EtOAc = 2/1) to afford pure **2x** as a white solid (77 mg, 92% yield).

Characterization data for **2x:**

^{1}H NMR (600 MHz, $CDCl_3$): δ 5.77 (s, 1H), 4.59 (dd, J = 9.1, 7.8 Hz, 1H), 4.13 (d, J = 3.0 Hz, 1H), 3.83 (t, J = 3.2 Hz, 1H), 2.53 – 2.42 (m, 2H), 2.39 – 2.33 (m, 1H), 2.32 (s, 3H), 2.20 – 2.07 (m, 1H), 2.01 (s, 3H), 2.00 – 1.98 (m, 1H), 1.78 – 1.64 (m, 2H), 1.59 – 1.42 (m, 4H), 1.39 – 1.32 (m, 2H), 1.35 (s, 3H), 1.13 (td, J = 13.1, 4.3 Hz, 1H), 0.97 (td, J = 11.7, 4.5 Hz, 1H), 0.84 (s, 3H).

^{13}C NMR (151 MHz, $CDCl_3$): δ 200.26, 194.85, 171.38, 165.43, 128.86, 82.44, 76.73, 49.45, 49.36, 46.41, 42.54, 38.08, 37.07, 36.22, 34.23, 31.88, 31.34, 27.30, 22.82, 21.24, 20.68, 20.34, 12.15.

HRMS (ESI): Calculated for $C_{23}H_{32}NaO_5S$ $(M+Na)^+$: 443.1863; Found: 443.1863.

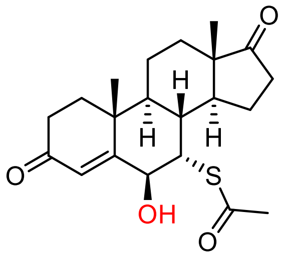

6β-Hydroxy-7α-acetylthioandrostenedione (2y)

7 mol% Na_2-eosin Y; Irradiation time: 12 h. Purification by flash chromatography on silica (PE/EtOAc = 1/1) to afford pure **2y** as a white solid (61 mg, 81% yield).

Characterization data for **2y:**

^{1}H NMR (500 MHz, $CDCl_3$): δ 5.81 (d, J = 0.9 Hz, 1H), 4.19 (d, J = 3.0 Hz, 1H), 3.98 (t, J = 3.2 Hz, 1H), 2.65 (td, J = 11.0, 3.4 Hz, 1H), 2.55 – 2.48 (m, 1H), 2.48 – 2.42 (m, 1H), 2.42 – 2.36 (m, 1H), 2.35 (s, 3H), 2.16 – 1.96 (m, 2H), 1.90 – 1.62 (m, 4H), 1.61 – 1.46 (m, 3H), 1.39 (s, 3H), 1.30 – 1.17 (m, 2H), 1.04 (td, J = 11.7, 4.4 Hz, 1H), 0.94 (s, 3H).

^{13}C NMR (126 MHz, $CDCl_3$): δ 220.07, 199.90, 194.45, 164.78, 129.11, 76.87, 49.66, 48.59, 47.61, 47.21, 38.19, 37.17, 35.70, 34.27, 31.87, 31.40, 31.05, 21.28, 20.52, 20.45, 13.85.

HRMS (ESI): Calculated for $C_{21}H_{28}NaO_4S$ $(M+Na)^+$: 399.1601; Found: 399.1602.

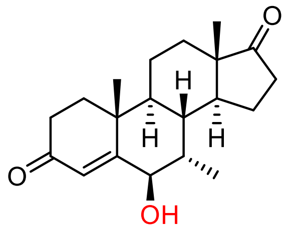

6β-Hydroxy-7α-methylandrostenedione (2z)

7 mol% Na_2-eosin Y; Irradiation time: 12 h. Purification by flash chromatography on silica (PE/EtOAc = 1/1) to afford pure **2z** as a white solid (45 mg, 71% yield).

Characterization data for **2z:**

^{1}H NMR (500 MHz, $CDCl_3$): δ 5.81 (d, J = 1.0 Hz, 1H), 4.06 (d, J = 2.6 Hz, 1H), 2.53 – 2.47 (m, 1H), 2.46 – 2.43 (m, 1H), 2.42 – 2.28 (m, 2H), 2.14 – 2.00 (m, 3H), 1.96 – 1.87 (m, 1H), 1.83 (ddd, J = 13.1, 4.2, 2.7 Hz, 1H), 1.77 – 1.71 (m, 1H), 1.71 – 1.63 (m, 1H), 1.63 – 1.55 (m, 1H), 1.54 – 1.40 (m, 2H), 1.37 (s, 3H), 1.28 – 1.23 (m, 1H), 1.23 – 1.18 (m, 1H), 0.93 (s, 3H), 0.78 (d, J = 7.4 Hz, 3H).

^{13}C NMR (126 MHz, $CDCl_3$): δ 220.78, 200.26, 167.37, 128.28, 79.27, 47.78, 46.94, 45.85, 38.28, 37.47, 36.26, 35.77, 34.35, 31.77, 31.33, 21.32, 20.79, 20.53, 13.72, 10.97.

HRMS (ESI): Calculated for $C_{20}H_{29}O_3$ $(M+H)^+$: 317.2111; Found: 317.2102.

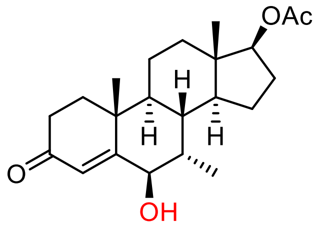

6β-Hydroxy-7α-methyltestosterone acetate (2aa)

7 mol% Na_2-eosin Y; Irradiation time: 12 h. Purification by flash chromatography on silica (PE/EtOAc = 2/1) to afford pure **2aa** as a white solid (56 mg, 78% yield).

Characterization data for **2aa:**

¹H NMR (500 MHz, CDCl₃): δ 5.80 (s, 1H), 4.61 (dd, J = 9.1, 7.9 Hz, 1H), 4.02 (d, J = 2.6 Hz, 1H), 2.49 (ddd, J = 17.3, 14.9, 4.9 Hz, 1H), 2.42 – 2.31 (m, 1H), 2.25 – 2.12 (m, 2H), 2.03 (s, 3H), 2.00 – 1.94 (m, 1H), 1.81 – 1.66 (m, 2H), 1.66 – 1.60 (m, 1H), 1.59 – 1.50 (m, 2H), 1.50 – 1.45 (m, 1H), 1.44 – 1.37 (m, 1H), 1.36 (s, 3H), 1.28 – 1.09 (m, 4H), 0.86 (s, 3H), 0.73 (d, J = 7.4 Hz, 3H).

¹³C NMR (126 MHz, CDCl₃): δ 200.23, 171.38, 167.50, 128.24, 82.72, 79.53, 46.11, 45.75, 42.71, 38.24, 37.52, 36.88, 36.67, 34.40, 31.89, 27.50, 22.93, 21.29, 20.82, 20.77, 12.11, 10.98.

HRMS (ESI): Calculated for $C_{22}H_{33}O_4$ $(M+H)^+$: 361.2373; Found: 361.2368.

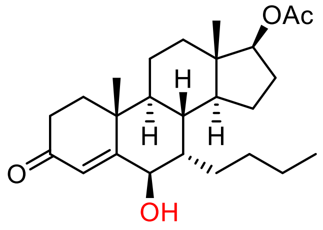

6β-Hydroxy-7α-n-butyltestosterone acetate (2ab)

7 mol% Na_2-eosin Y; Irradiation time: 12 h. Purification by flash chromatography on silica (PE/EtOAc = 5/2) to afford pure **2ab** as a white solid (64 mg, 80% yield).

Characterization data for **2ab:**

¹H NMR (400 MHz, CDCl₃): δ 5.79 (s, 1H), 4.61 (dd, J = 9.1, 7.8 Hz, 1H), 4.21 (d, J = 2.6 Hz, 1H), 2.48 (ddd, J = 17.3, 14.8, 4.9 Hz, 1H), 2.42 – 2.31 (m, 1H), 2.27 – 2.12 (m, 2H), 2.04 – 1.99 (m, 1H), 2.03 (s, 3H), 1.81 – 1.64 (m, 3H), 1.62 – 1.43 (m, 4H), 1.35 (s, 3H), 1.33 – 1.06 (m, 9H), 0.90 – 0.75 (m, 7H).

¹³C NMR (101 MHz, CDCl₃): δ 200.34, 171.40, 168.00, 128.10, 82.76, 76.21, 46.42, 45.51, 42.65, 42.33, 38.08, 37.48, 36.61, 34.42, 32.11, 30.62, 27.45, 24.13, 23.13, 23.07, 21.29, 20.94, 20.86, 14.20, 12.03.

HRMS (ESI): Calculated for $C_{25}H_{39}O_4$ $(M+H)^+$: 403.2843; Found: 403.2847.

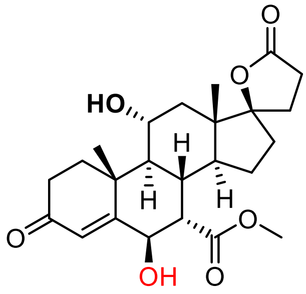

2ac

4 mol% Na_2-eosin Y; Irradiation time: 12 h. Purification by flash chromatography on silica (PE/EtOAc = 1/8) to afford pure **2ac** as a white solid (66 mg, 77% yield).

Characterization data for **2ac:**

^{1}H NMR (500 MHz, CD_3OD): δ 5.78 (d, J = 1.0 Hz, 1H), 4.37 (d, J = 2.5 Hz, 1H), 4.09 (td, J = 10.4, 4.6 Hz, 1H), 3.69 (s, 3H), 3.02 – 2.76 (m, 2H), 2.72 – 2.41 (m, 6H), 2.38 – 2.19 (m, 2H), 2.11 – 1.95 (m, 3H), 1.90 – 1.77 (m, 2H), 1.58 (s, 3H), 1.58 – 1.43 (m, 3H), 1.08 (s, 3H).

^{13}C NMR (126 MHz, CD_3OD): δ 203.12, 179.19, 173.22, 170.55, 128.32, 96.93, 74.53, 69.54, 53.31, 52.01, 50.83, 47.44, 46.49, 43.85, 40.17, 39.96, 36.19, 35.23, 32.13, 31.92, 29.99, 23.48, 21.24, 15.82.

HRMS (ESI): Calculated for $C_{24}H_{33}O_7$ $(M+H)^+$: 433.2221; Found: 433.2206.

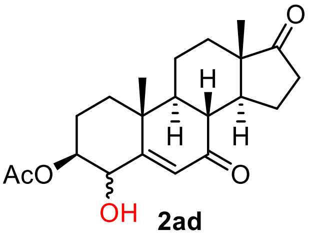

2ad

d.r. **= 1.8:1**

7 mol% Na_2-eosin Y; Irradiation time: 12 h. Purification by flash chromatography on silica (PE/EtOAc = 1/1) to afford **2ad** (mixture of a pair of diastereomers) as a white solid (44 mg, 61% yield, *d.r.* = 1.8:1).

Characterization data for **2ad:**

^{1}H NMR (600 MHz, $CDCl_3$): δ 6.25 (d, J = 2.0 Hz, 1H), 5.84 (s, 0.56H), 4.73 (ddd, J = 12.2, 4.4, 3.2 Hz, 0.56H), 4.63 (ddd, J = 11.4, 10.0, 4.8 Hz, 1H), 4.38 (dd, J = 3.3, 1.2 Hz, 0.56H), 4.35 (dd, J = 10.1, 2.1 Hz, 1H), 2.83 – 2.68 (m, 1.80H), 2.49 – 2.33 (m, 3.54H), 2.09 (d, J = 2.1 Hz, 5.24H), 2.04 – 2.00 (m, 1.40H), 1.94 (dq, J = 13.7, 3.9 Hz, 1.76H), 1.86 – 1.80 (m, 1.76H), 1.79 – 1.67 (m, 4.97H), 1.66 – 1.52 (m, 5.39H), 1.39 (s, 1.77H), 1.35 – 1.26 (m, 2.07H), 1.23 (s, 4.20H), 0.87 (d, J = 1.7 Hz, 4.76H).

^{13}C NMR (151 MHz, $CDCl_3$): δ 220.46, 220.42, 201.76, 201.09, 171.39, 170.15, 165.58, 163.72, 129.28, 122.95, 73.88, 73.74, 71.49, 51.05, 50.40, 47.95, 47.84, 45.84, 45.66, 44.96, 44.04, 39.58, 38.19, 36.05, 35.71, 35.69, 30.69, 30.67, 25.64, 24.21, 24.19, 21.40, 21.33, 21.29, 20.56, 20.16, 18.60, 18.27, 13.82.

HRMS (ESI): Calculated for $C_{21}H_{29}O_5$ $(M+H)^+$: 361.2010; Found: 361.2011.

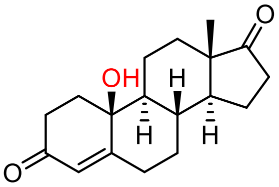

2ae

4 mol% Na_2-eosin Y; Irradiation time: 12 h. Purification by flash chromatography on silica (PE/EtOAc = 1/1) to afford pure **2ae** as a white solid (36 mg, 63% yield).

Spectral data for **2ae** matched reported values[24].

Characterization data for **2ae:**

^{1}H NMR (400 MHz, CDCl$_3$): δ 5.78 (d, J = 1.9 Hz, 1H), 2.67 (tdd, J = 14.1, 5.1, 2.0 Hz, 1H), 2.60 – 2.41 (m, 2H), 2.40 – 2.29 (m, 2H), 2.25 – 1.83 (m, 8H), 1.81 – 1.73 (m, 1H), 1.72 – 1.66 (m, 1H), 1.65 – 1.54 (m, 1H), 1.37 – 1.22 (m, 2H), 1.20 – 1.04 (m, 2H), 0.94 (s, 3H).

^{13}C NMR (101 MHz, CDCl$_3$): δ 220.74, 199.16, 164.17, 124.98, 70.36, 52.71, 50.55, 47.73, 35.89, 34.91, 33.81, 33.72, 31.89, 31.12, 30.65, 21.93, 19.80, 13.84.

HRMS (ESI): Calculated for $C_{18}H_{25}O_3$ $(M+H)^+$: 289.1798; Found: 289.1793.

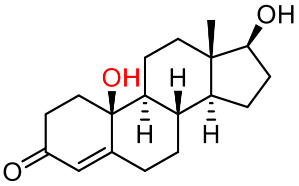

2af

4 mol% Na_2-eosin Y; Irradiation time: 12 h. Purification by flash chromatography on silica (PE/EtOAc = 1/3) to afford pure **2af** as a white solid (35 mg, 60% yield). Spectral data for **2af** matched reported values[25].

Characterization data for **2af:**

^{1}H NMR (600 MHz, CD$_3$OD): δ 5.75 (d, J = 1.8 Hz, 1H), 3.60 (t, J = 8.7 Hz, 1H), 2.69 (tdd, J = 14.0, 5.3, 2.0 Hz, 1H), 2.60 (ddd, J = 16.8, 12.3, 4.9 Hz, 1H), 2.35 – 2.26 (m, 2H), 2.22 (dt, J = 14.0, 4.9 Hz, 1H), 2.05 – 1.97 (m, 1H), 1.97 – 1.80 (m, 4H), 1.77 – 1.59 (m, 3H), 1.56 – 1.46 (m, 1H), 1.39 – 1.27 (m, 1H), 1.17 – 0.97 (m, 4H), 0.83 (s, 3H).

^{13}C NMR (151 MHz, CD$_3$OD): δ 202.29, 168.66, 124.74, 82.31, 70.91, 54.50, 51.45, 44.02, 37.55, 36.46, 34.53, 34.51, 33.16, 32.70, 30.65, 24.32, 21.16, 11.49.

HRMS (ESI): Calculated for $C_{18}H_{27}O_3$ $(M+H)^+$: 291.1955; Found: 291.1958.

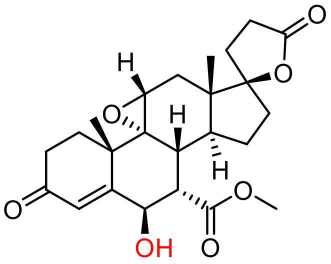

6β-Hydroxyeplerenone (2ag)

7 mol% Na_2-eosin Y; Irradiation time: 12 h. Purification by flash chromatography on silica (PE/EtOAc = 1/5) to afford pure **2ag** as a white solid (77 mg, 90% yield).

Characterization data for **2ag:**

^{1}H NMR (500 MHz, CDCl$_3$): δ 5.92 (s, 1H), 4.57 (d, J = 2.3 Hz, 1H), 3.62 (s, 3H), 3.08 (d, J = 5.3 Hz, 1H), 3.00 (dd, J = 11.2, 4.6 Hz, 1H), 2.91 (dd, J = 4.6, 2.2 Hz, 1H), 2.59 (ddd, J = 18.0, 9.5, 6.5 Hz, 1H), 2.53 – 2.36 (m, 3H), 2.28 (ddd, J = 13.2, 9.4, 6.6 Hz, 1H), 2.23 – 2.09 (m, 2H), 2.00 – 1.83 (m, 4H), 1.81 (d, J = 14.2 Hz, 1H), 1.68 (dd, J = 14.6, 5.4 Hz, 1H), 1.59 (s, 3H), 1.58 – 1.48 (m, 1H), 1.33 (ddd, J = 13.3, 5.2, 2.4 Hz, 1H), 1.02 (s, 3H).

^{13}C NMR (126 MHz, CDCl$_3$): δ 199.71, 176.58, 171.51, 165.16, 128.86, 94.96, 72.48, 65.55, 51.87, 51.78, 47.90, 44.17, 39.36, 36.96, 35.12, 33.39, 32.95, 31.29, 31.07, 29.13, 28.20, 24.59, 22.00, 16.38.

HRMS (ESI): Calculated for $C_{24}H_{31}O_7$ $(M+H)^+$: 431.2064; Found: 431.2066.

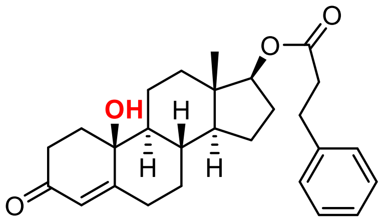

10β-hydroxynandrolone phenylpropionate (2ah)

4 mol% Na$_2$-eosin Y; Irradiation time: 12 h. Purification by flash chromatography on silica (PE/EtOAc = 8/5) to afford pure **2ah** as a white solid (46 mg, 55% yield).

Characterization data for **2ah:**

^{1}H NMR (600 MHz, CDCl$_3$): δ 7.28 – 7.21 (m, 2H), 7.20 – 7.13 (m, 3H), 5.73 (d, J = 1.9 Hz, 1H), 4.57 (dd, J = 9.2, 7.8 Hz, 1H), 2.92 (t, J = 7.8 Hz, 2H), 2.60 (t, J = 7.8 Hz, 3H), 2.59 – 2.48 (m, 1H), 2.35 – 2.24 (m, 2H), 2.21 – 2.07 (m, 2H), 1.92 (ddd, J = 14.0, 12.3, 4.5 Hz, 1H), 1.85 – 1.78 (m, 1H), 1.75 (dd, J = 11.2, 3.3 Hz, 1H), 1.72 – 1.67 (m, 1H), 1.66 – 1.52 (m, 3H), 1.48 – 1.38 (m, 1H), 1.36 – 1.25 (m, 1H), 1.19 – 1.11 (m, 1H), 1.10 – 0.94 (m, 3H), 0.77 (s, 3H).

^{13}C NMR (151 MHz, CDCl$_3$): δ 199.39, 173.10, 164.53, 140.55, 128.56, 128.38, 126.34, 124.82, 82.57, 70.34, 52.57, 49.93, 42.65, 36.37, 36.12, 35.11, 33.79, 33.72, 32.03, 31.32, 31.17, 27.54, 23.62, 20.00, 12.04.

HRMS (ESI): Calculated for $C_{27}H_{35}O_4$ $(M+H)^+$: 423.2530; Found: 423.2523.

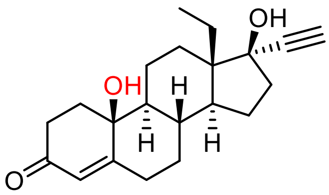

10β-Hydroxylevonorgestrel (2ai)

7 mol% Na$_2$-eosin Y; Irradiation time: 12 h; Acetone (10 mL) as solvent. Purification by flash chromatography on silica (PE/EtOAc = 1/1) to afford starting material **1ai** (17 mg, 27%) as a white solid and pure **2ai** as a white solid (37 mg, 56% yield).

Characterization data for **2ai:**

^{1}H NMR (500 MHz, CD_3OD): δ 5.78 (d, J = 1.8 Hz, 1H), 2.91 (s, 1H), 2.72 (tdd, J = 13.9, 5.4, 2.0 Hz, 1H), 2.64 (ddd, J = 16.4, 12.5, 4.9 Hz, 1H), 2.41 – 2.22 (m, 4H), 2.16 – 2.07 (m, 2H), 2.01 – 1.87 (m, 3H), 1.80 – 1.49 (m, 8H), 1.45 – 1.30 (m, 2H), 1.05 (t, J = 7.4 Hz, 3H).

^{13}C NMR (126 MHz, CD_3OD): δ 202.28, 168.40, 124.87, 89.10, 81.79, 74.78, 70.81, 54.17, 52.37, 49.07, 40.34, 36.77, 34.58, 34.49, 33.20, 32.74, 29.51, 23.50, 21.44, 19.89, 9.97.

HRMS (ESI): Calculated for $C_{21}H_{29}O_3$ $(M+H)^+$: 329.2111; Found: 329.2115.

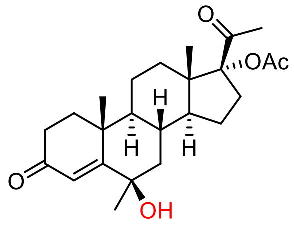

6β-hydroxymedroxyprogesterone acetate (2aj-1)

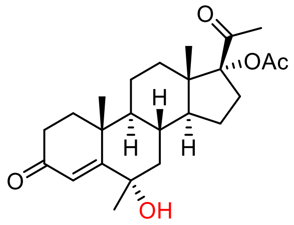

6α-hydroxymedroxyprogesterone acetate (2aj-2)

7 mol% Na_2-eosin Y; On 0.50 mmol scale; Irradiation time: 12 h. Purification by flash chromatography on silica (PE/EtOAc = 2/3) to afford pure **2aj-1** as a white solid and **2aj-2** as a white solid (164 mg, 82% total yield, **2aj-1**/**2aj-2** =1.9/1, determined by NMR analysis of the mixture of **2aj-1** and **2aj-2**). Spectral data for **2aj-1** and **2aj-2** matched reported values[26].

Characterization data for **2aj-1:**

^{1}H NMR (500 MHz, $CDCl_3$): δ 6.06 (s, 1H), 3.23 – 2.76 (m, 1H), 2.54 (ddd, J = 17.3, 15.0, 4.9 Hz, 1H), 2.42 (ddd, J = 17.6, 4.5, 2.5 Hz, 1H), 2.13 (s, 3H), 2.12 – 2.07 (m, 2H), 2.08 (s, 3H), 2.05 – 1.93 (m, 2H), 1.89 – 1.67 (m, 6H), 1.62 (dt, J = 12.6, 3.2 Hz, 1H), 1.52 (qd, J = 13.2, 4.2 Hz, 1H), 1.46 (s, 3H), 1.43 (s, 3H), 1.41 – 1.35 (m, 1H), 1.31 – 1.19 (m, 1H), 1.04 (ddd, J = 12.2, 10.9, 4.1 Hz, 1H), 0.74 (s, 3H).

^{13}C NMR (126 MHz, $CDCl_3$): δ 204.17, 200.87, 170.78, 170.26, 123.12, 96.84, 71.40, 52.98, 51.01, 46.94, 45.45, 38.63, 37.71, 33.90, 31.12, 30.86, 30.49, 29.35, 26.53, 23.85, 21.32, 20.81, 20.09, 14.57.

HRMS (ESI): Calculated for $C_{24}H_{34}NaO_5$ $(M+Na)^+$: 425.2298; Found: 425.2297.

Characterization data for **2aj-2:**

^{1}H NMR (500 MHz, $CDCl_3$): δ 6.37 (d, J = 0.9 Hz, 1H), 3.06 – 2.79 (m, 1H), 2.47 (ddd, J = 17.5, 14.8, 4.9 Hz, 1H), 2.36 (dddd, J = 17.6, 4.7, 2.5, 1.0 Hz, 1H), 2.23 – 2.13 (m, 1H), 2.12 – 2.08 (m, 1H),2.09 (s, 3H), 2.03 (s, 3H), 2.00 – 1.91 (m, 2H), 1.83 – 1.60 (m, 6H), 1.57 (ddd, J = 12.7, 4.2, 2.9 Hz, 1H), 1.45 – 1.36 (m, 1H), 1.40 (s, 3H), 1.35 – 1.26 (m, 2H), 1.24 (s, 3H), 1.03 (ddd, J = 12.4, 10.7, 4.1 Hz, 1H), 0.67 (s, 3H).

^{13}C NMR (126 MHz, CDCl$_3$): δ 204.06, 200.25, 175.64, 170.79, 122.38, 96.73, 71.91, 52.50, 50.96, 47.14, 46.77, 38.69, 38.47, 33.77, 33.33, 30.97, 30.40, 30.35, 26.49, 23.99, 21.34, 20.81, 20.61, 14.53.

HRMS (ESI): Calculated for $C_{24}H_{34}NaO_5$ $(M+Na)^+$: 425.2298; Found: 425.2296.

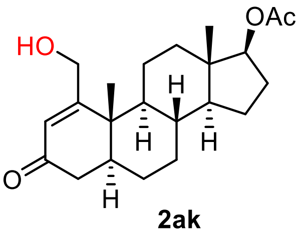

2ak

7 mol% Na_2-eosin Y; Irradiation time: 12 h. Purification by flash chromatography on silica (PE/EtOAc = 1/2) to afford pure **2ak** as a white solid (13 mg, 18% yield) and starting material **1ak** (32 mg, 47%).

Characterization data for **2ak:**

^{1}H NMR (600 MHz, CDCl$_3$): δ 6.19 (s, 1H), 4.67 – 4.56 (m, 1H), 4.53 – 4.23 (m, 2H), 2.41 (dd, J = 18.5, 13.6 Hz, 1H), 2.24 (dd, J = 18.2, 4.2 Hz, 1H), 2.18 – 2.11 (m, 1H), 2.04 (s, 3H), 1.78 – 1.68 (m, 2H), 1.68 – 1.58 (m, 2H), 1.52 – 1.42 (m, 4H), 1.39 – 1.28 (m, 4H), 1.23 – 1.13 (m, 3H), 1.09 (s, 3H), 0.84 (s, 3H).

^{13}C NMR (126 MHz, CDCl$_3$): δ 199.67, 173.77, 171.24, 124.30, 82.60, 63.57, 51.32, 49.65, 44.92, 42.55, 42.52, 41.57, 37.90, 37.29, 30.08, 28.54, 27.45, 25.54, 23.67, 21.19, 14.62, 12.69.

HRMS (ESI): Calculated for $C_{22}H_{33}O_4$ $(M+H)^+$: 361.2373; Found: 361.2380.

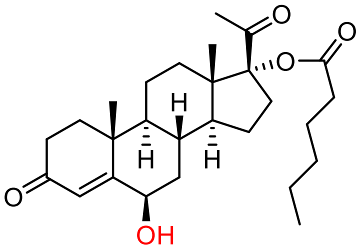

6β-Hydroxy-17-hexanoyloxy-pregn-4-ene-3,20-dione (2al)

7 mol% Na_2-eosin Y; Irradiation time: 12 h. Purification by flash chromatography on silica (PE/EtOAc = 1/1) to afford pure **2al** as a colorless oil (25 mg, 28% yield).

Characterization data for **2al:**

^{1}H NMR (600 MHz, CDCl$_3$): δ 5.82 (s, 1H), 4.36 (t, J = 2.9 Hz, 1H), 2.93 (dd, J = 13.3, 11.3 Hz, 1H), 2.52 (ddd, J = 17.1, 15.0, 4.9 Hz, 1H), 2.39 (dt, J = 17.7, 3.7 Hz, 1H), 2.34 (t, J = 7.5 Hz, 2H), 2.08 – 2.05 (m, 1H), 2.03 (s, 3H), 2.03 – 1.99 (m, 1H), 1.95 (td, J = 13.1, 4.4 Hz, 1H), 1.79 – 1.69 (m, 4H), 1.67 – 1.55 (m, 4H), 1.54 – 1.45 (m, 1H), 1.37 (s, 3H), 1.34 – 1.27 (m, 6H), 1.03 – 0.95 (m, 1H), 0.88 (t, J = 6.8 Hz, 3H), 0.87 – 0.85 (m, 1H),0.69 (s, 3H).

^{13}**C NMR (151 MHz, CDCl$_3$):** δ 204.38, 200.63, 173.55, 168.28, 126.48, 96.55, 72.97, 53.13, 51.29, 47.06, 38.56, 38.09, 37.20, 34.59, 34.32, 31.39, 31.13, 30.51, 29.92, 26.60, 24.64, 23.91, 22.43, 20.72, 19.64, 14.57, 14.05.

HRMS (ESI): Calculated for $C_{27}H_{41}O_5$ $(M+H)^+$: 445.2949; Found: 445.2939.

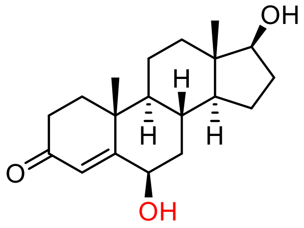

6β-Hydroxytestosterone (2am)

7 mol% Na$_2$-eosin Y; Irradiation time: 12 h. Purification by flash chromatography on silica (PE/EtOAc = 1/1) to afford pure **2am** as a white solid (19 mg, 31% yield). Spectral data for **2am** matched reported values[22].

Characterization data for **2am**:

^{1}**H NMR (600 MHz, CDCl$_3$):** δ 5.81 (d, J = 1.0 Hz, 1H), 4.34 (t, J = 2.9 Hz, 1H), 3.65 (t, J = 8.6 Hz, 1H), 2.51 (ddd, J = 17.3, 15.1, 5.0 Hz, 1H), 2.38 (dddd, J = 17.3, 4.2, 2.7, 1.1 Hz, 1H), 2.13 – 1.95 (m, 4H), 1.87 (ddd, J = 12.5, 4.1, 2.8 Hz, 1H), 1.70 (ddd, J = 14.9, 13.4, 4.5 Hz, 1H), 1.68 – 1.56 (m, 2H), 1.54 – 1.48 (m, 1H), 1.50 – 1.42 (m, 1H), 1.38 (s, 3H), 1.39 – 1.34 (m, 1H), 1.27 – 1.19 (m, 1H), 1.09 (td, J = 12.9, 4.3 Hz, 1H), 0.97 (ddd, J = 12.2, 10.8, 7.3 Hz, 1H), 0.94 – 0.87 (m, 1H), 0.81 (s, 3H).

^{13}**C NMR (126 MHz, CDCl$_3$):** δ 200.56, 168.47, 126.50, 81.83, 73.17, 53.88, 50.63, 43.06, 38.21, 38.19, 37.27, 36.56, 34.37, 30.62, 29.93, 23.44, 20.75, 19.69, 11.24.

HRMS (ESI): Calculated for $C_{19}H_{29}O_3$ $(M+H)^+$: 305.2111; Found: 305.2099.

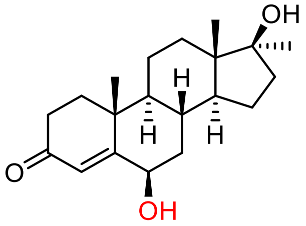

6β-Hydroxymethyltestosterone (2an)

7 mol% Na$_2$-eosin Y; Irradiation time: 12 h. Purification by flash chromatography on silica (PE/EtOAc = 1/1) to afford pure **2an** as a white solid (14 mg, 22% yield). Spectral data for **2an** matched reported values[25].

Characterization data for **2an**:

^{1}**H NMR (500 MHz, CD$_3$OD):** δ 5.82 (d, J = 0.9 Hz, 1H), 4.30 (t, J = 2.9 Hz, 1H), 2.61 (ddd, J = 17.2, 15.0, 5.1 Hz, 1H), 2.41 – 2.29 (m, 1H), 2.17 – 2.03 (m, 2H), 2.06 – 1.98 (m, 1H), 1.97 – 1.87 (m, 1H), 1.81 – 1.73 (m, 1H), 1.76 – 1.70 (m, 1H), 1.72 –

1.65 (m, 1H), 1.68 – 1.59 (m, 2H), 1.56 (td, J = 12.6, 3.7 Hz, 1H), 1.44 (s, 3H), 1.44 – 1.37 (m, 1H), 1.40 – 1.33 (m, 2H), 1.33 – 1.24 (m, 2H), 1.23 (s, 3H), 0.96 (s, 3H).

^{13}C NMR (126 MHz, CD_3OD): δ 203.13, 171.59, 126.71, 82.17, 73.64, 55.22, 51.48, 46.77, 39.55, 39.39, 39.20, 38.36, 35.06, 32.66, 31.88, 26.05, 24.17, 21.77, 19.70, 14.62.

HRMS (ESI): Calculated for $C_{20}H_{31}O_3$ $(M+H)^+$: 319.2268; Found: 319.2269.

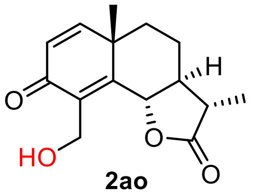

2ao

On 1.00 mmol scale; 7 mol% Na_2-eosin Y; Irradiation time: 12 h. Purification by flash chromatography on silica (PE/EtOAc = 1/2) to afford pure **2ao** as a white solid (63 mg, 24% yield) and starting material **1ao** (43 mg, 17%).

^{1}H NMR (600 MHz, CD_3OD): δ 6.95 (d, J = 9.9 Hz, 1H), 6.26 (d, J = 9.9 Hz, 1H), 5.08 (d, J = 11.3 Hz, 1H), 4.82 – 4.59 (m, 2H), 2.67 – 2.58 (m, 1H), 2.08 – 2.00 (m, 2H), 1.97 (td, J = 11.9, 3.5 Hz, 1H), 1.88 – 1.78 (m, 1H), 1.58 (td, J = 13.7, 13.3, 4.5 Hz, 1H), 1.41 (s, 3H), 1.25 (d, J = 7.0 Hz, 3H).

^{13}C NMR (151 MHz, CD_3OD): δ 187.68, 179.77, 158.86, 158.37, 131.47, 126.35, 82.48, 54.91, 54.59, 43.22, 41.78, 39.05, 25.40, 23.57, 12.59.

HRMS (ESI): Calculated for $C_{15}H_{19}O_4$ $(M+H)^+$: 263.1278 Found: 263.1277.

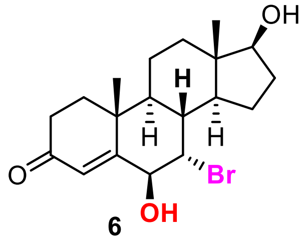

6

Characterization data for **6**:

^{1}H NMR (600 MHz, CD_3OD): δ 5.83 (d, J = 1.1 Hz, 1H), 4.39 (d, J = 2.9 Hz, 1H), 4.31 (t, J = 2.7 Hz, 1H), 3.63 (t, J = 8.7 Hz, 1H), 2.60 (ddd, J = 17.4, 15.1, 5.0 Hz, 1H), 2.44 – 2.30 (m, 1H), 2.17 – 2.09 (m, 2H), 2.08 – 2.00 (m, 1H), 1.89 (ddd, J = 12.7, 4.1, 2.8 Hz, 1H), 1.82 – 1.75 (m, 1H), 1.72 – 1.64 (m, 2H), 1.62 (dd, J = 12.8, 4.1 Hz, 1H), 1.56 – 1.47 (m, 2H), 1.41 (s, 3H), 1.37 – 1.33 (m, 2H), 1.08 (td, J = 12.7, 4.5 Hz, 1H), 0.85 (s, 3H).

^{13}C NMR (151 MHz, CD_3OD): δ 202.30, 167.80, 129.64, 82.14, 78.23, 60.89, 48.72, 47.45, 43.91, 39.24, 38.19, 37.34, 35.17, 35.06, 30.38, 23.12, 21.38, 20.76, 11.85.

HRMS (ESI): Calculated for $C_{19}H_{28}BrO_3$ $(M+H)^+$: 383.1216; Found: 383.1211.

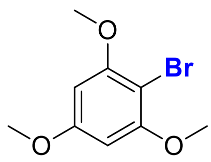

Bromo-1,3,5-trimethoxybenzene (7)

Characterization data for **7**:

^{1}H NMR (600 MHz, $CDCl_3$): δ 6.17 (s, 2H), 3.88 (s, 6H), 3.82 (s, 3H).

^{13}C NMR (151 MHz, $CDCl_3$): δ 160.59, 157.60, 92.14, 91.76, 56.52, 55.68.

HRMS (ESI): Calculated for $C_9H_{12}BrO_3$ $(M+H)^+$: 246.9964; Found: 246.9961.

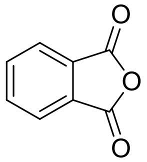

Phthalic anhydride (8)

Characterization data for **8**:

^{1}H NMR (600 MHz, DMSO-d_6): δ 8.09 – 8.08 (m, 2H), 8.02 – 8.01 (m, 2H).

^{13}C NMR (151 MHz, DMSO-d_6): δ 163.25, 136.22, 131.24, 125.37.

HRMS (ESI): Calculated for $C_8H_5O_3$ $(M+H)^+$: 149.0233; Found: 149.0234.

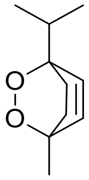

Ascaridole (9)

Characterization data for **9**:

^{1}H NMR (500 MHz, $CDCl_3$): δ 6.49 (d, J = 8.5 Hz, 1H), 6.41 (d, J = 8.5 Hz, 1H), 2.08 – 1.98 (m, 2H), 1.96 – 1.87 (m, 1H), 1.57 – 1.47 (m, 2H), 1.37 (s, 3H), 1.00 (d, J = 6.9 Hz, 6H).

^{13}C NMR (126 MHz, $CDCl_3$): δ 136.54, 133.20, 79.94, 74.51, 32.28, 29.68, 25.76, 21.56, 17.40, 17.31.

HRMS (ESI): Calculated for $C_{10}H_{16}NaO_2$ $(M+Na)^+$: 191.1043; Found: 191.1045.

2.19 NMR Spectral Data

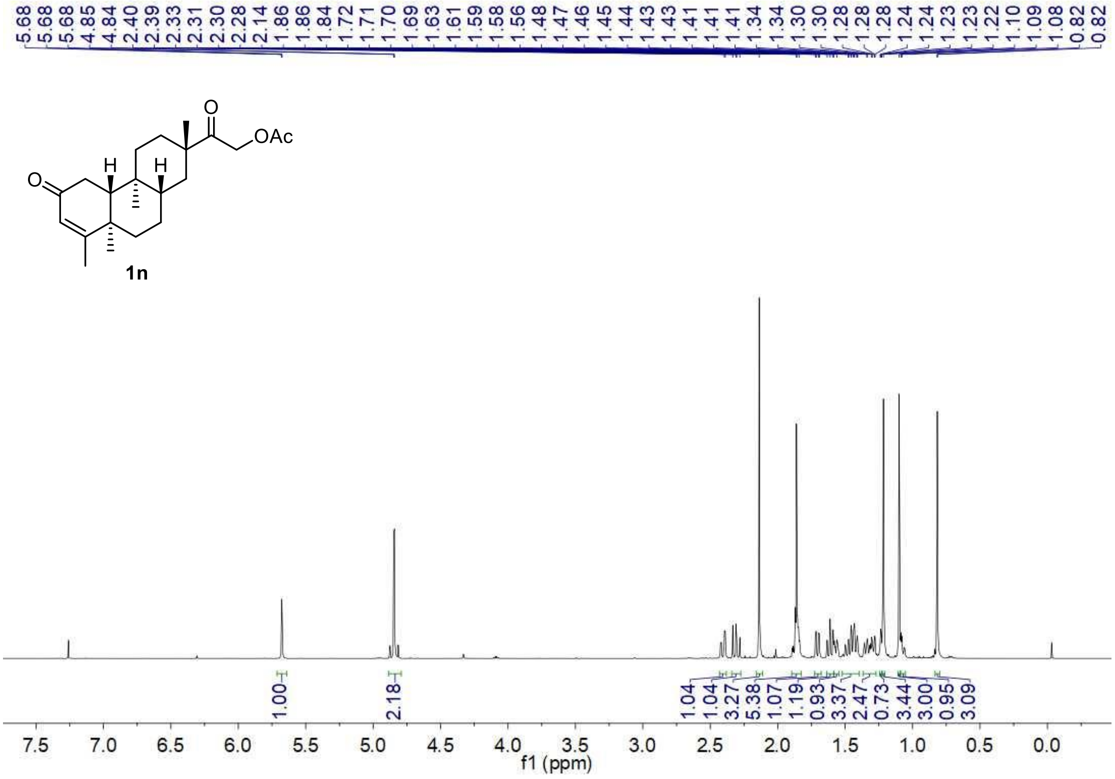

Supplementary Fig. 24. ^{1}H NMR spectrum of compound **1n (600 MHz, CDCl$_3$)**

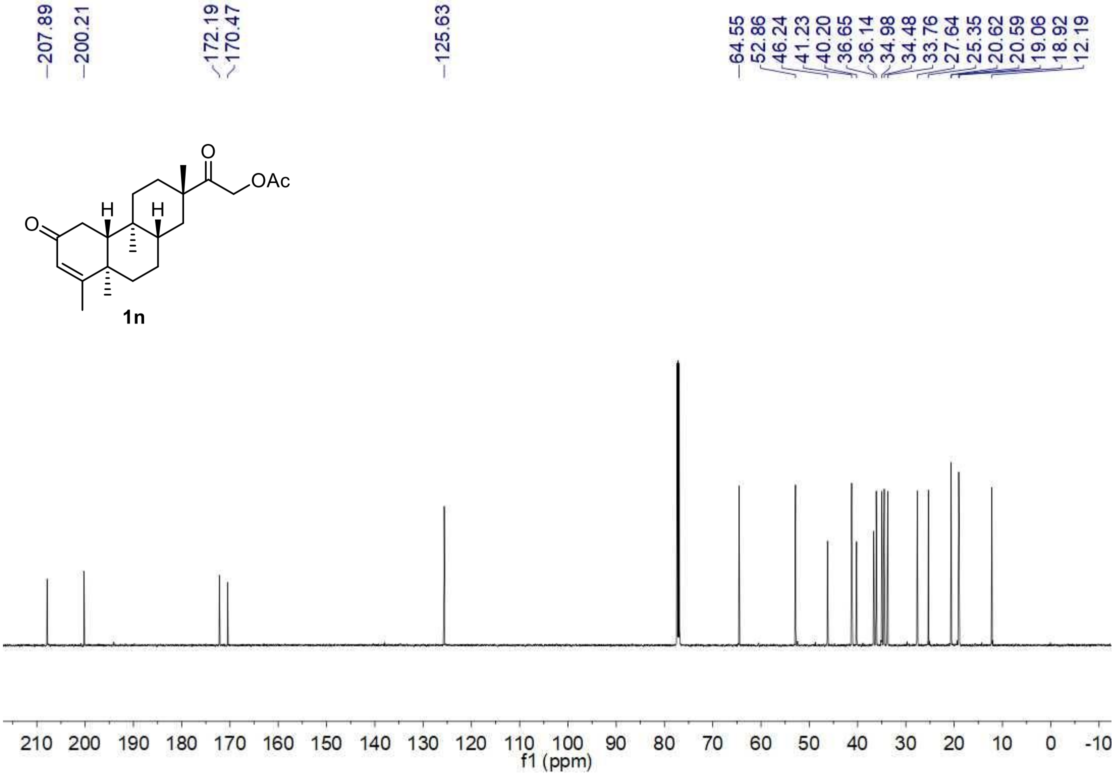

Supplementary Fig. 25. ^{13}C NMR spectrum of compound **1n (151 MHz, CDCl$_3$)**

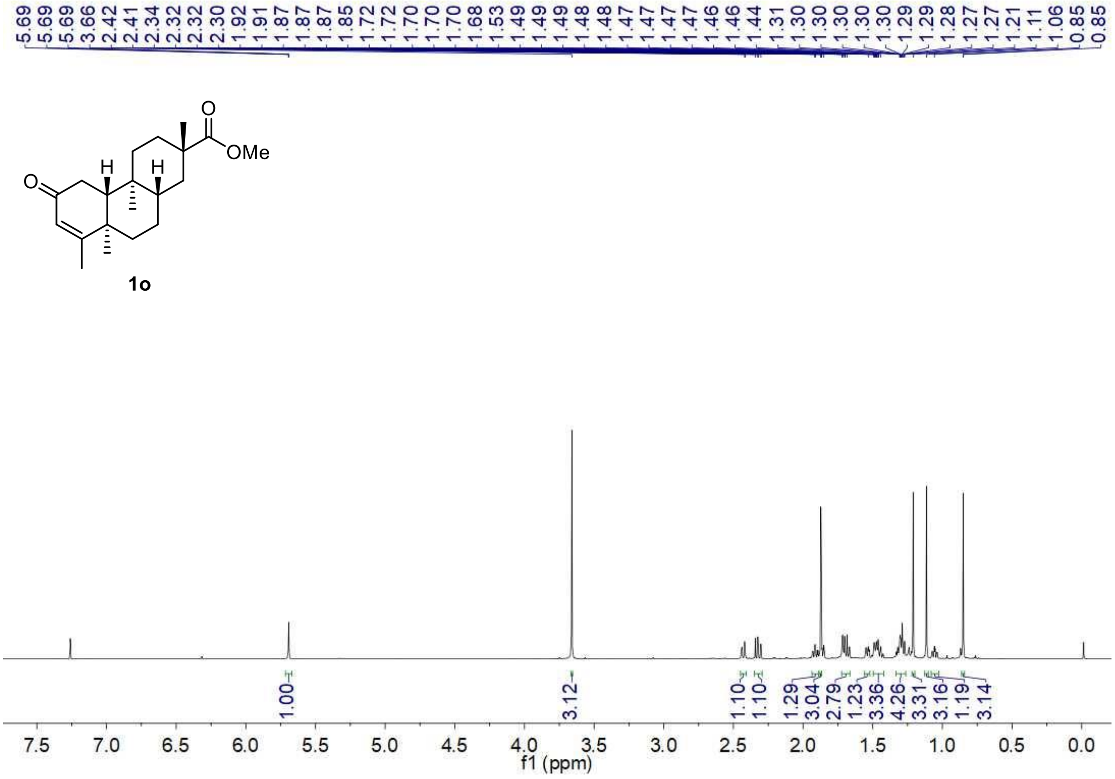

Supplementary Fig. 26. ^{1}H NMR spectrum of compound **1o (800 MHz, $CDCl_3$)**

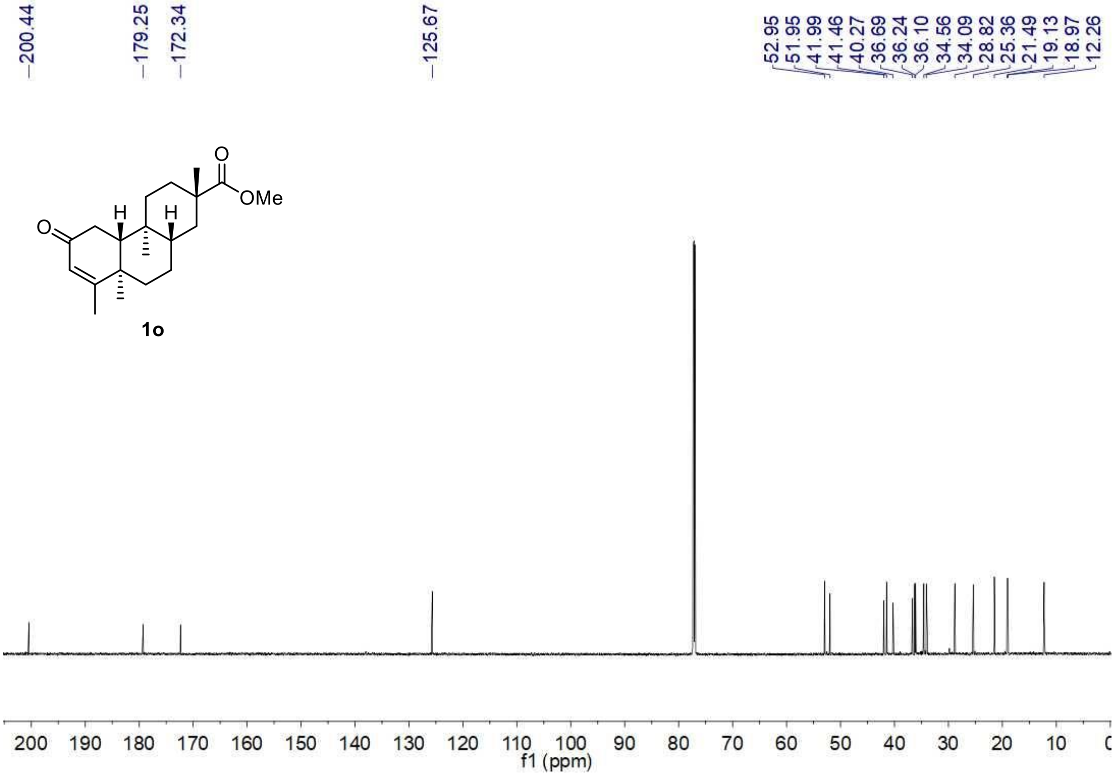

Supplementary Fig. 27. ^{13}C NMR spectrum of compound **1o (201 MHz, $CDCl_3$)**

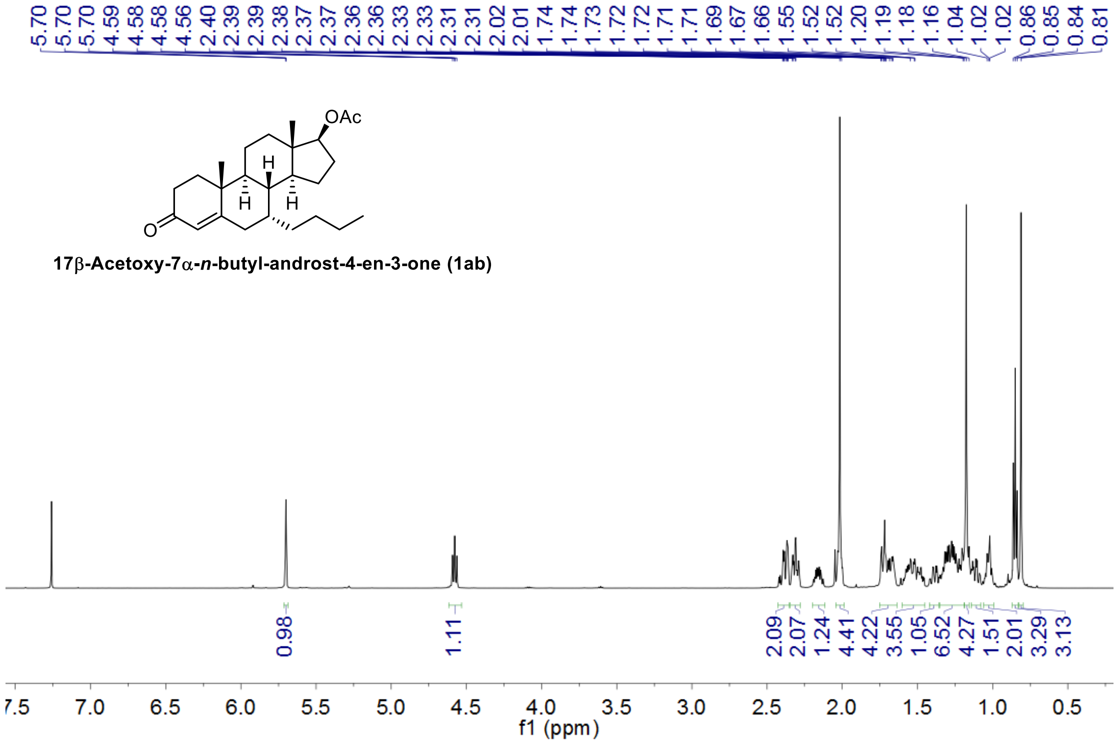

Supplementary Fig. 28. ^{1}H NMR spectrum of compound **1ab (600 MHz, CDCl$_3$)**

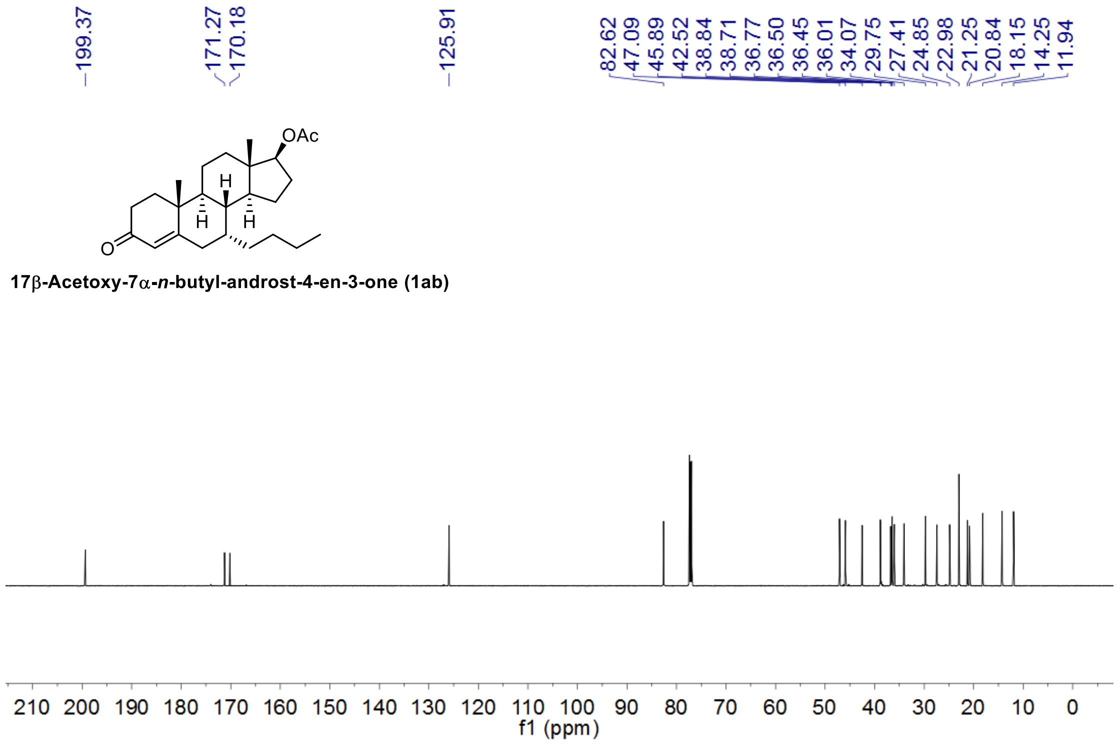

Supplementary Fig. 29. ^{13}C NMR spectrum of compound **1ab (151 MHz, CDCl$_3$)**

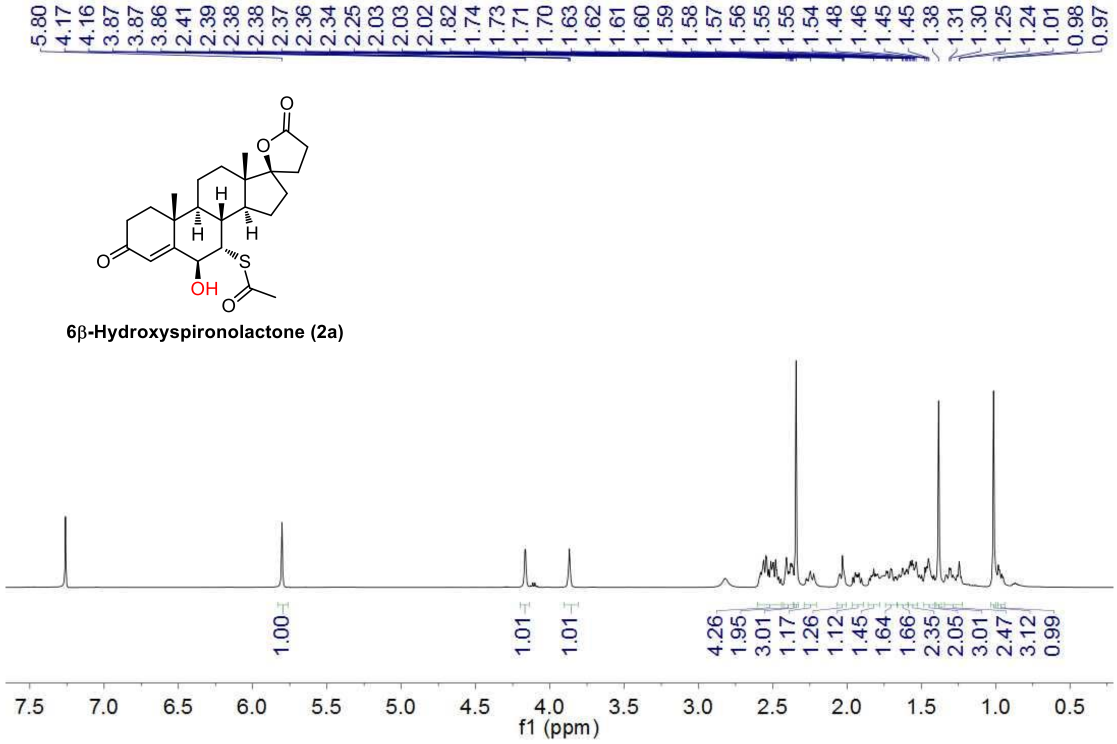

Supplementary Fig. 30. ^{1}H NMR spectrum of compound **2a (500 MHz, CDCl₃)**

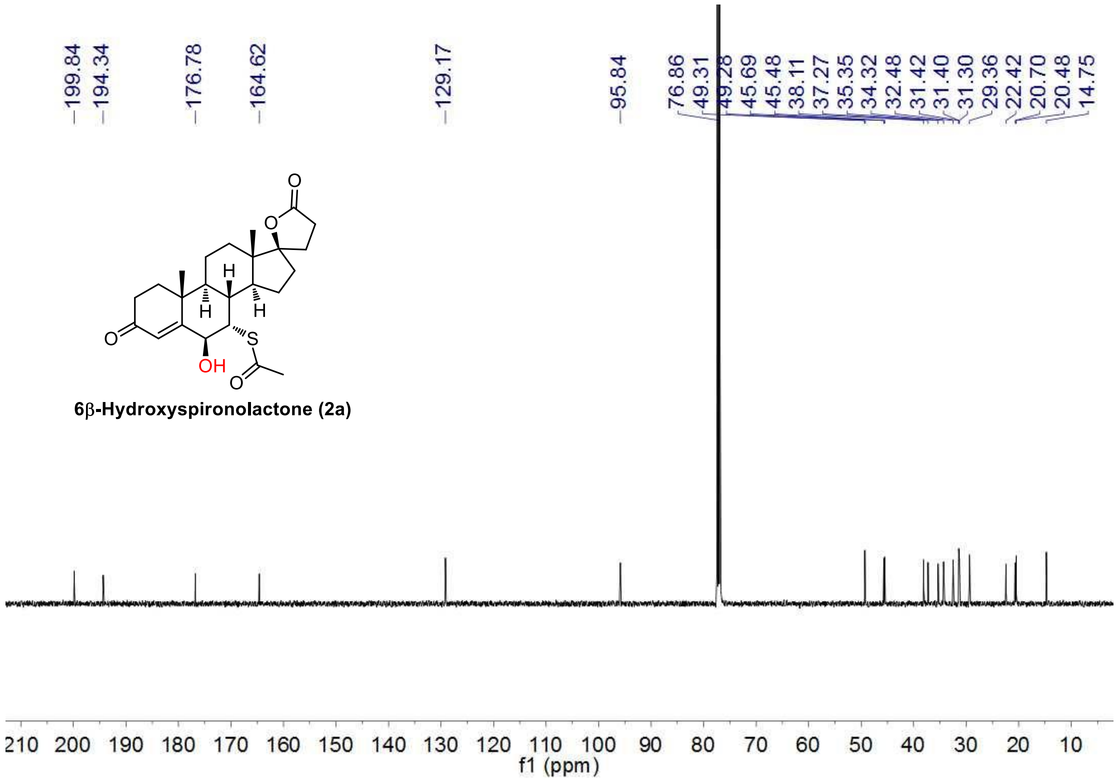

Supplementary Fig. 31. ^{13}C NMR spectrum of compound **2a (126 MHz, CDCl₃)**

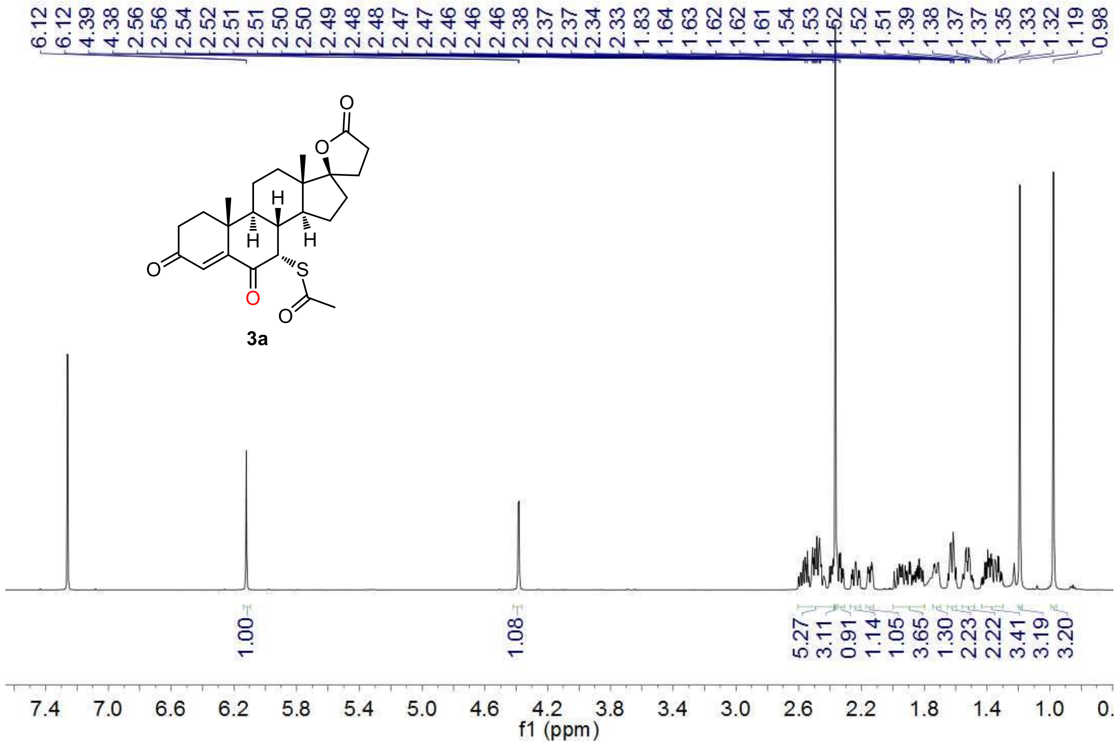

Supplementary Fig. 32. ^{1}H NMR spectrum of compound **3a (600 MHz, CDCl$_3$)**

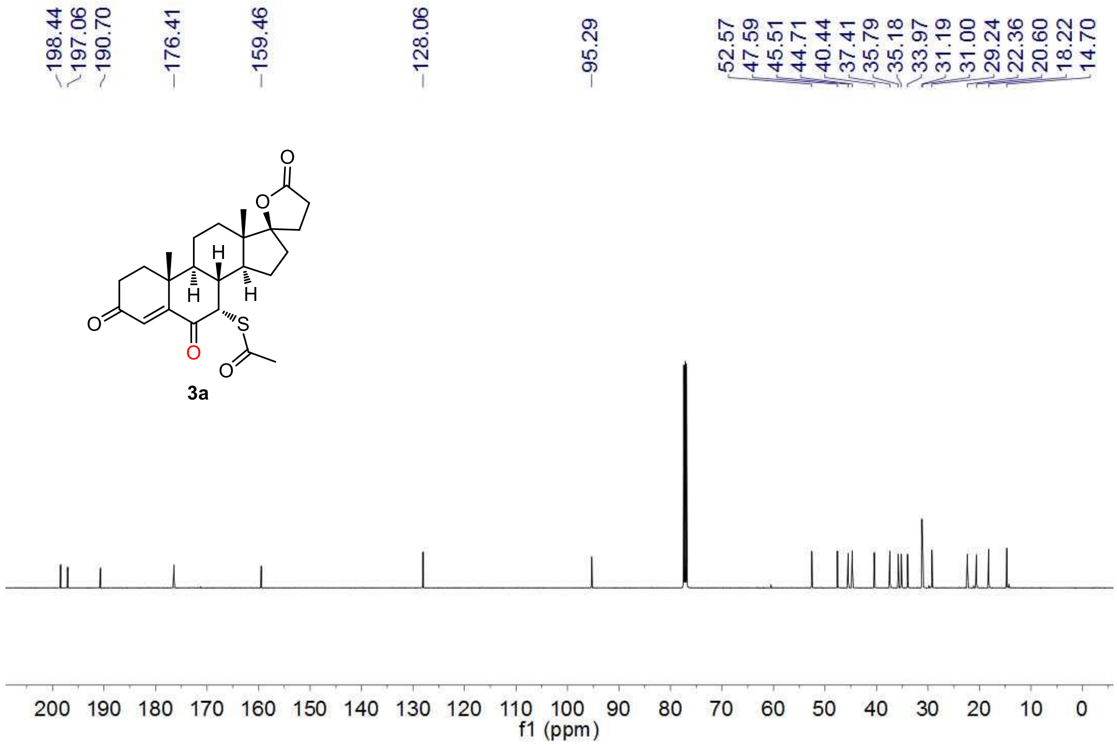

Supplementary Fig. 33. ^{13}C NMR spectrum of compound **3a (151 MHz, CDCl$_3$)**

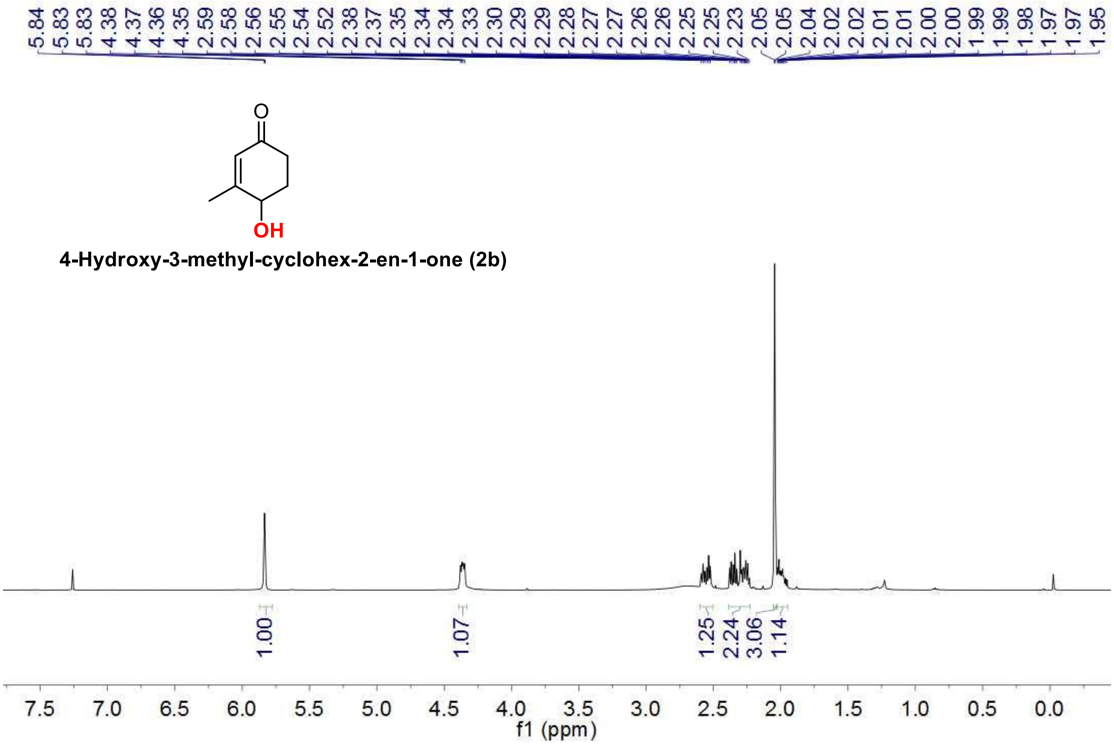

Supplementary Fig. 34. ^{1}H NMR spectrum of compound **2b (400 MHz, CDCl$_3$)**

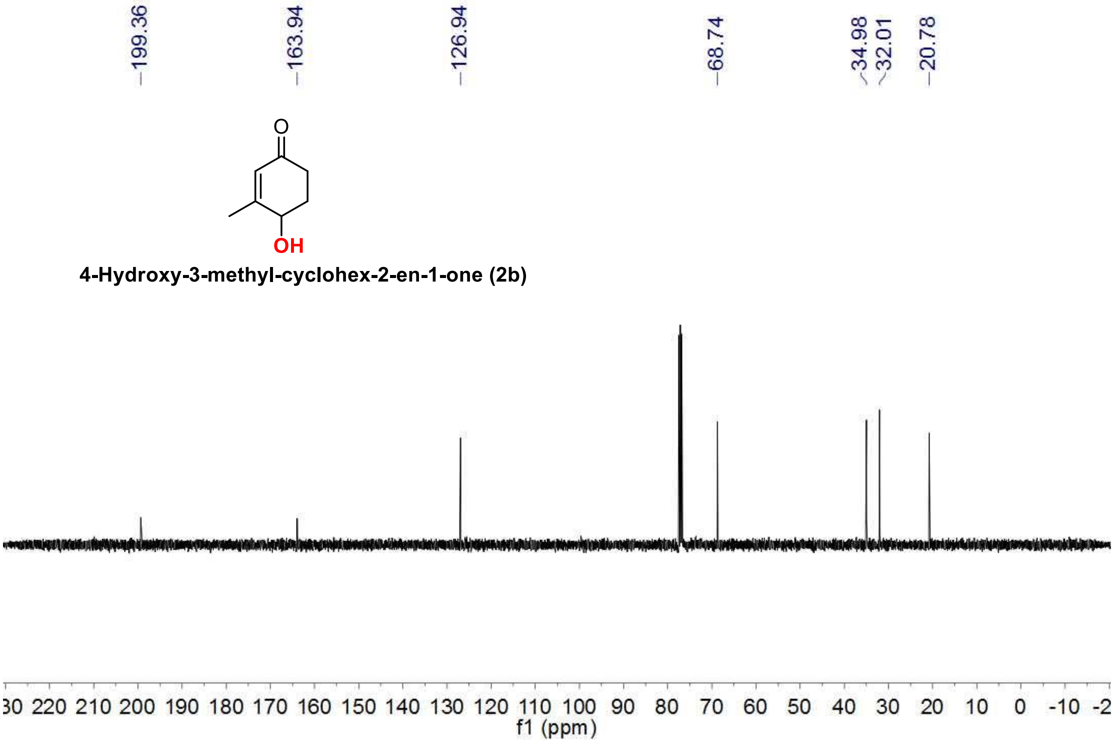

Supplementary Fig. 35. ^{13}C NMR spectrum of compound **2b (101 MHz, CDCl$_3$)**

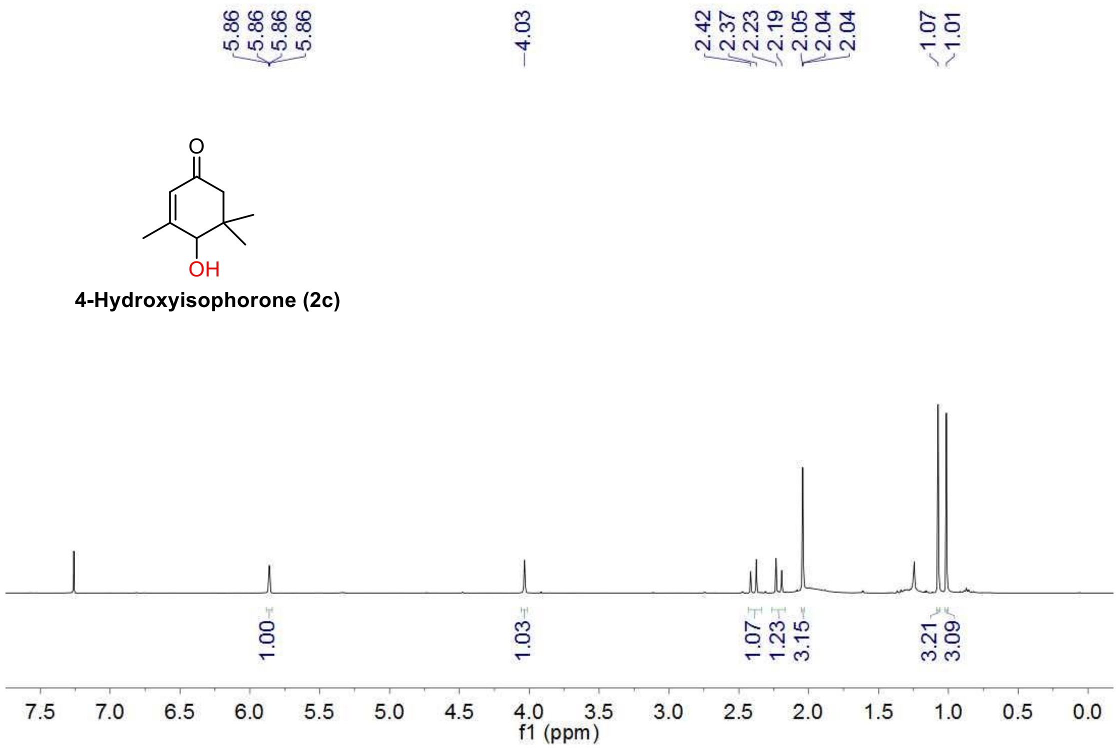

Supplementary Fig. 36. ^{1}H NMR spectrum of compound **2c (400 MHz, $CDCl_3$)**

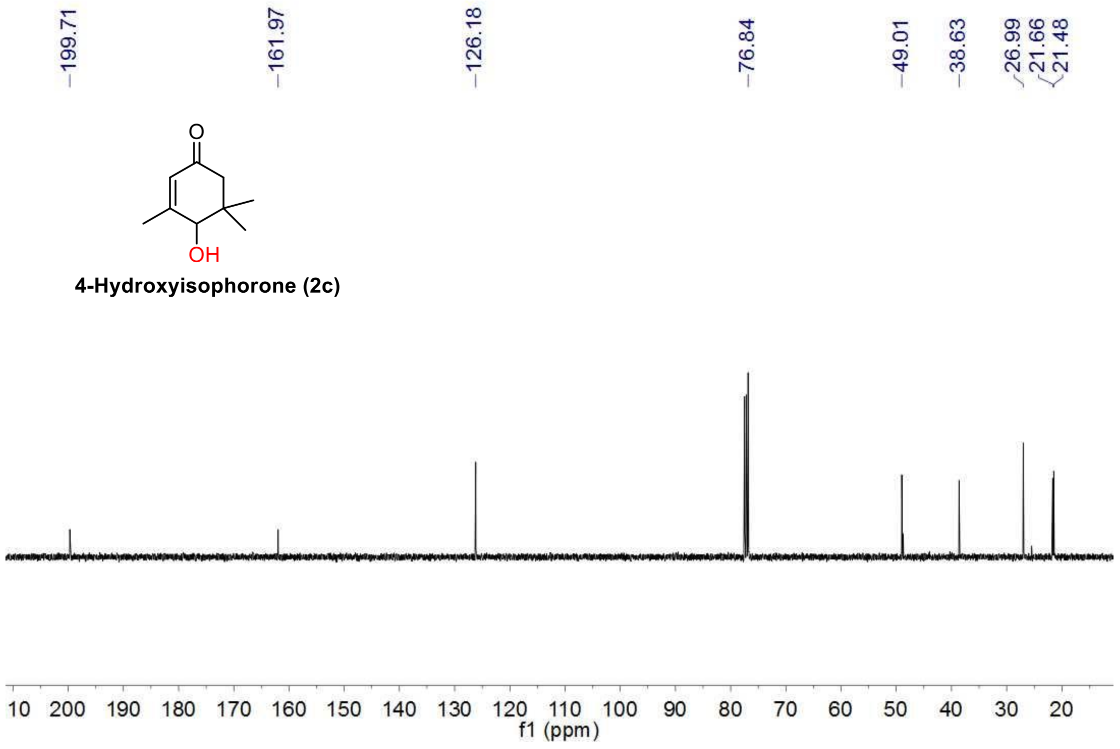

Supplementary Fig. 37. ^{13}C NMR spectrum of compound **2c (101 MHz, $CDCl_3$)**

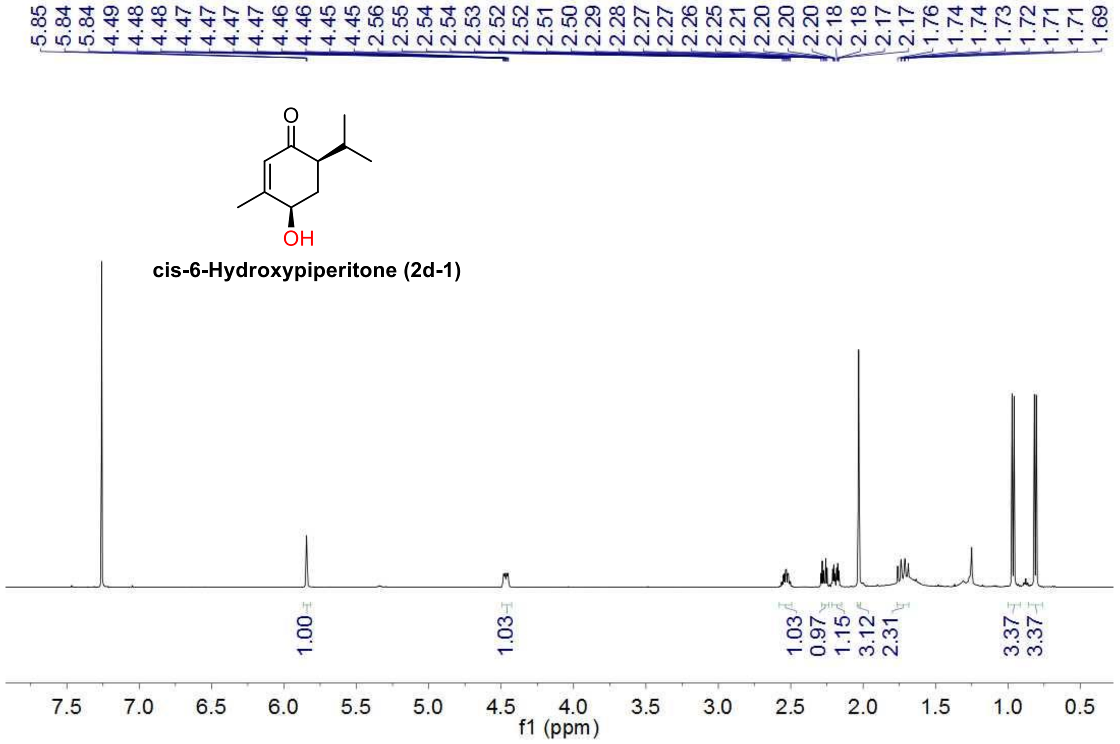

Supplementary Fig. 38. ^{1}H NMR spectrum of compound **2d-1 (500 MHz, CDCl$_3$)**

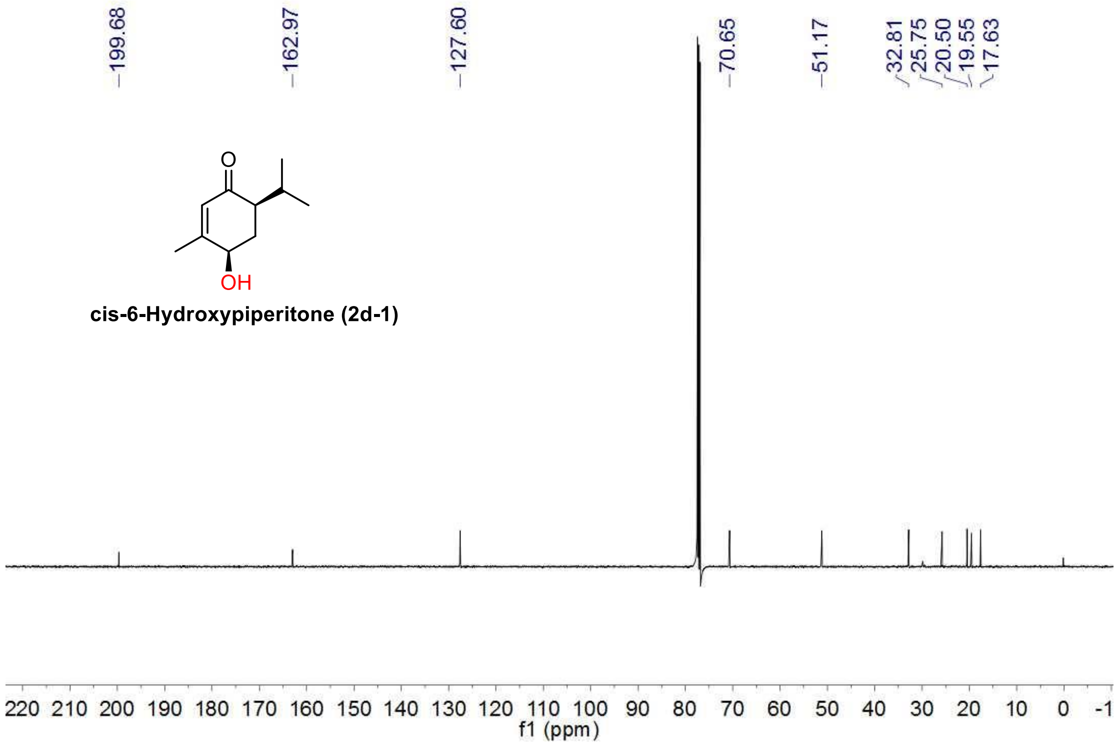

Supplementary Fig. 39. ^{13}C NMR spectrum of compound **2d-1 (126 MHz, CDCl$_3$)**

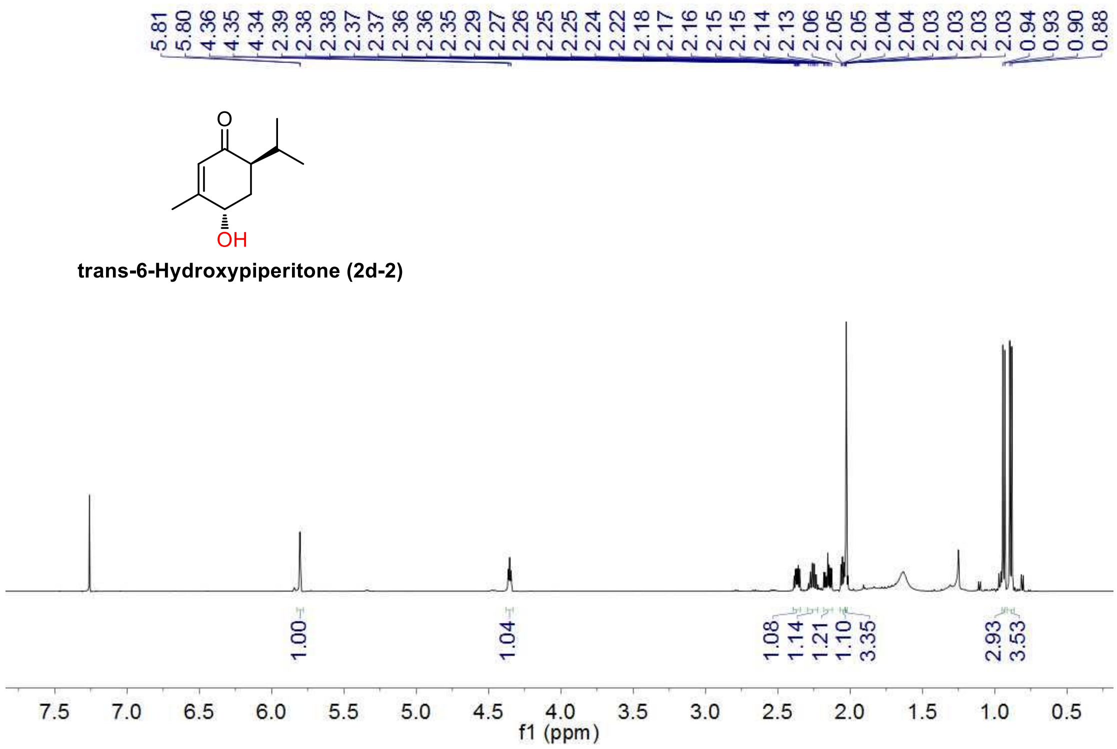

Supplementary Fig. 40. ^{1}H NMR spectrum of compound **2d-2 (500 MHz, $CDCl_3$)**

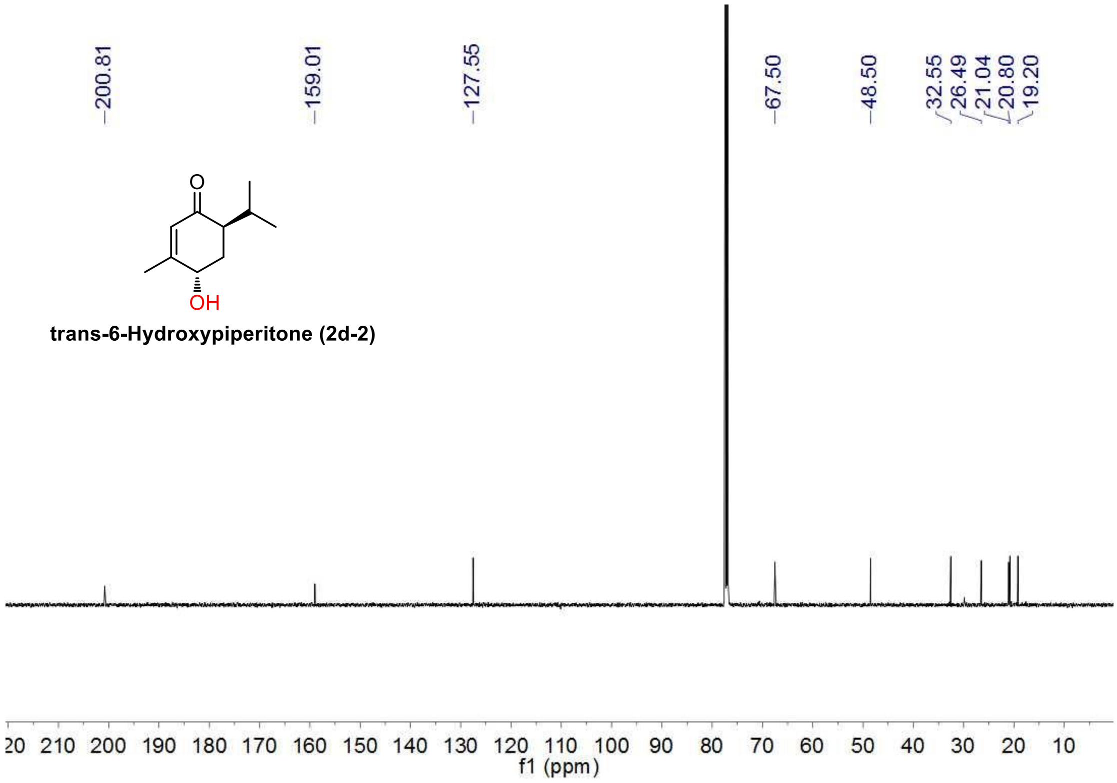

Supplementary Fig. 41. ^{13}C NMR spectrum of compound **2d-2 (126 MHz, $CDCl_3$)**

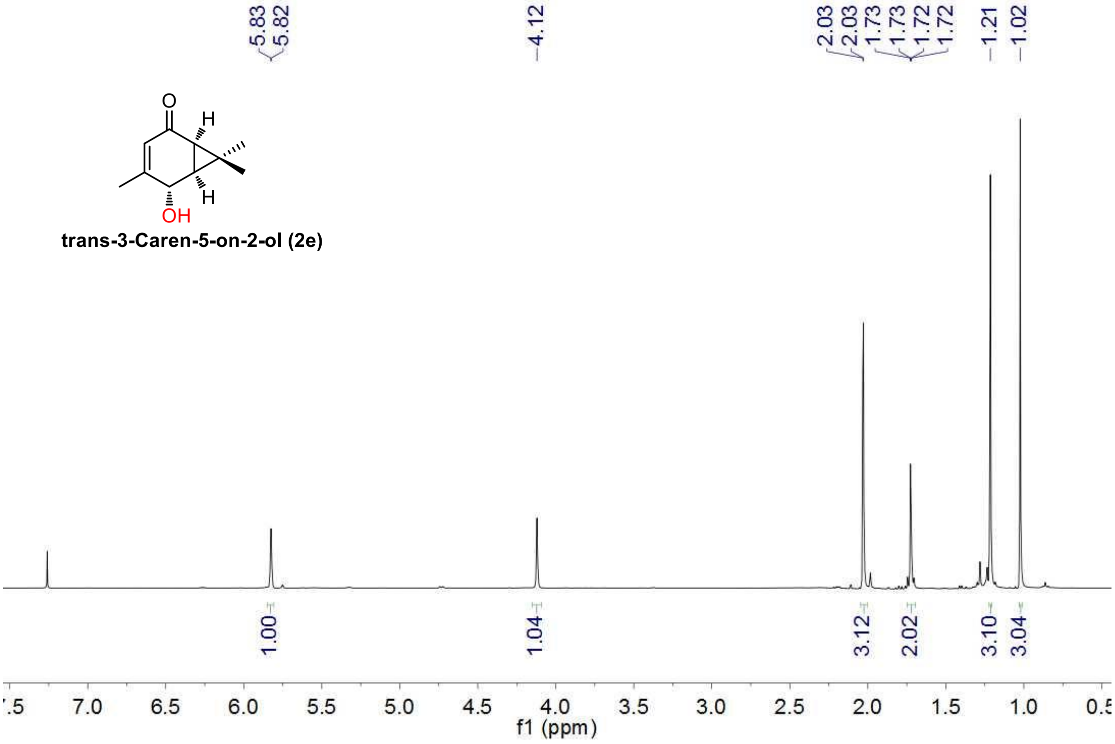

Supplementary Fig. 42. ^{1}H NMR spectrum of compound **2e (400 MHz, $CDCl_3$)**

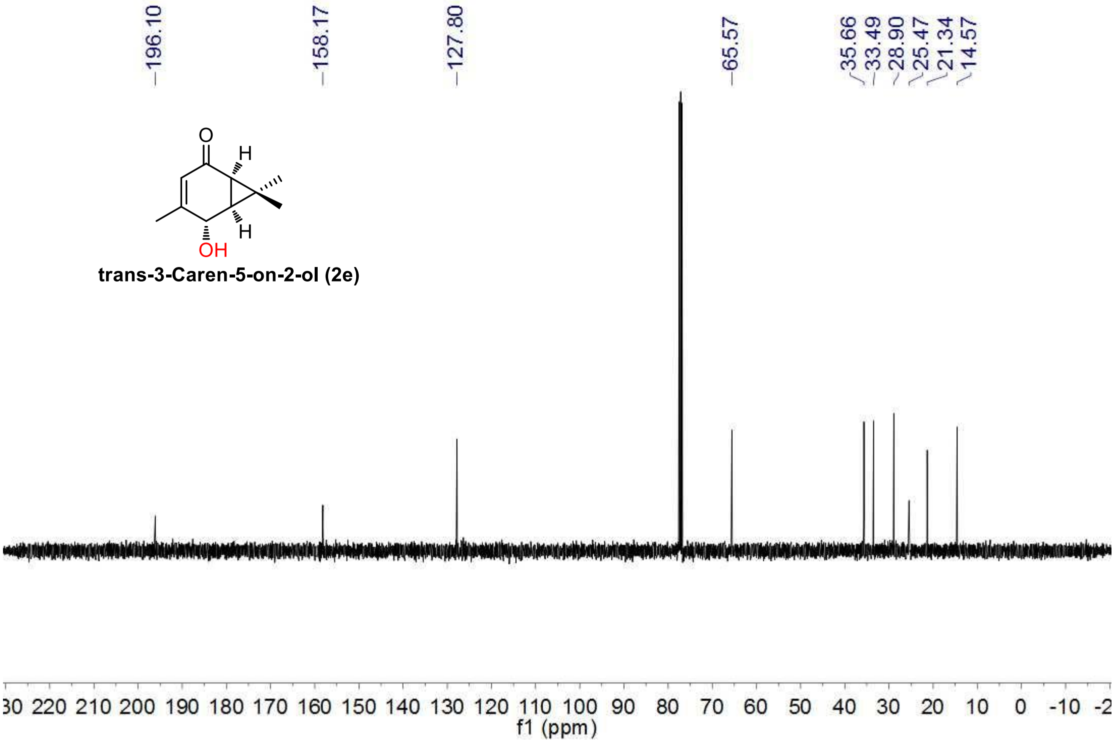

Supplementary Fig. 43. ^{13}C NMR spectrum of compound **2e (101 MHz, $CDCl_3$)**

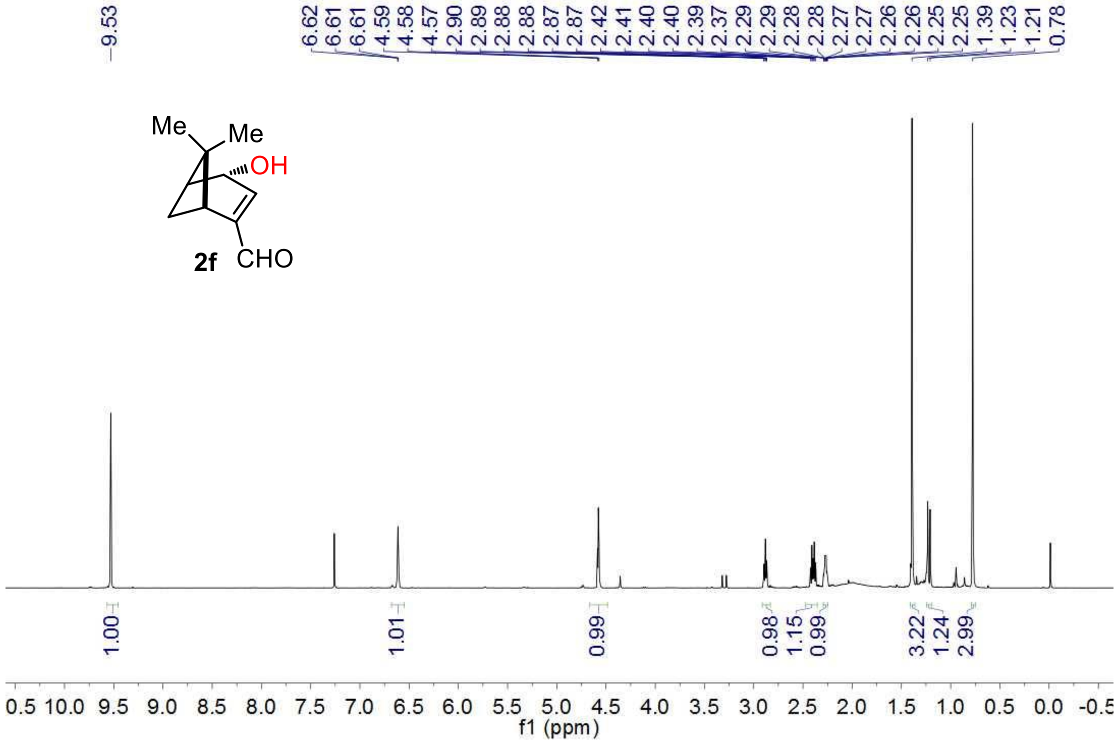

Supplementary Fig. 44. ^{1}H NMR spectrum of compound **2f (400 MHz, CDCl$_3$)**

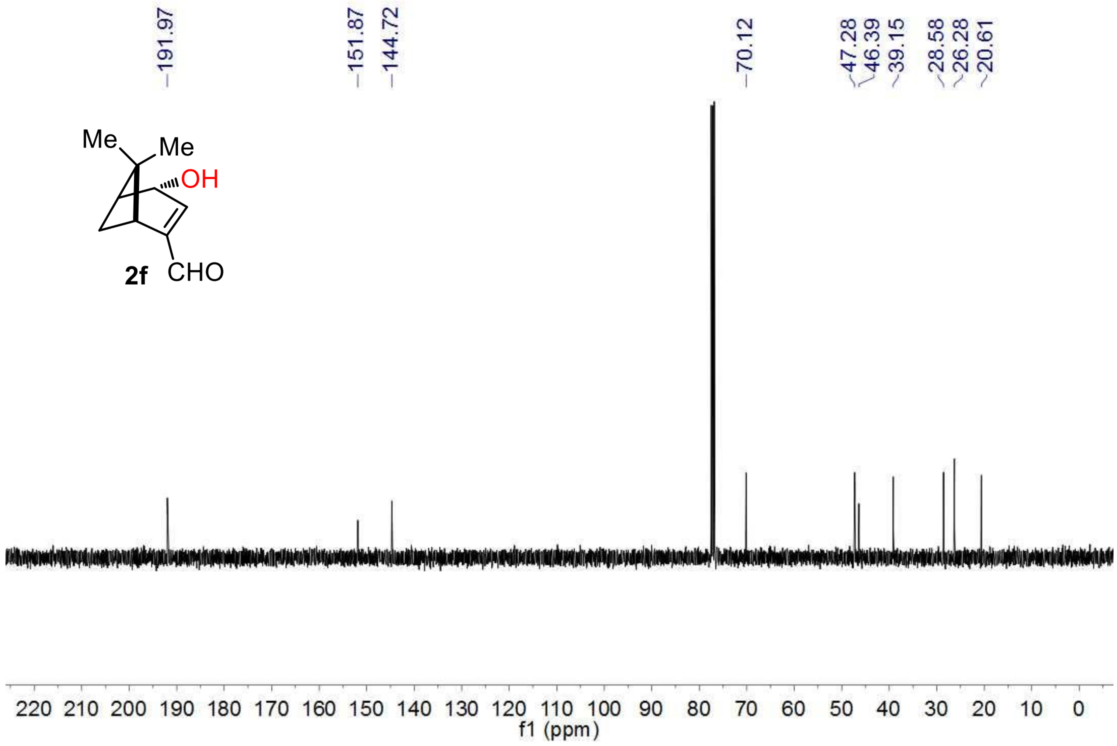

Supplementary Fig. 45. ^{13}C NMR spectrum of compound **2f (101 MHz, CDCl$_3$)**

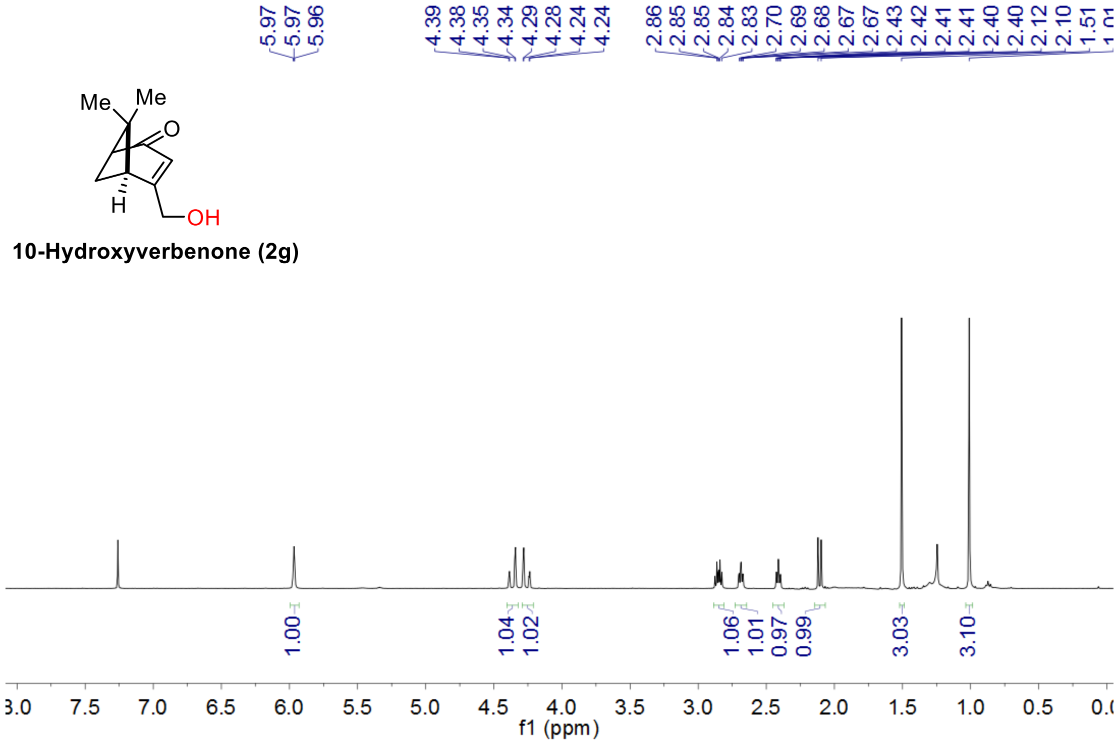

Supplementary Fig. 46. ^{1}H NMR spectrum of compound **2g (400 MHz, $CDCl_3$)**

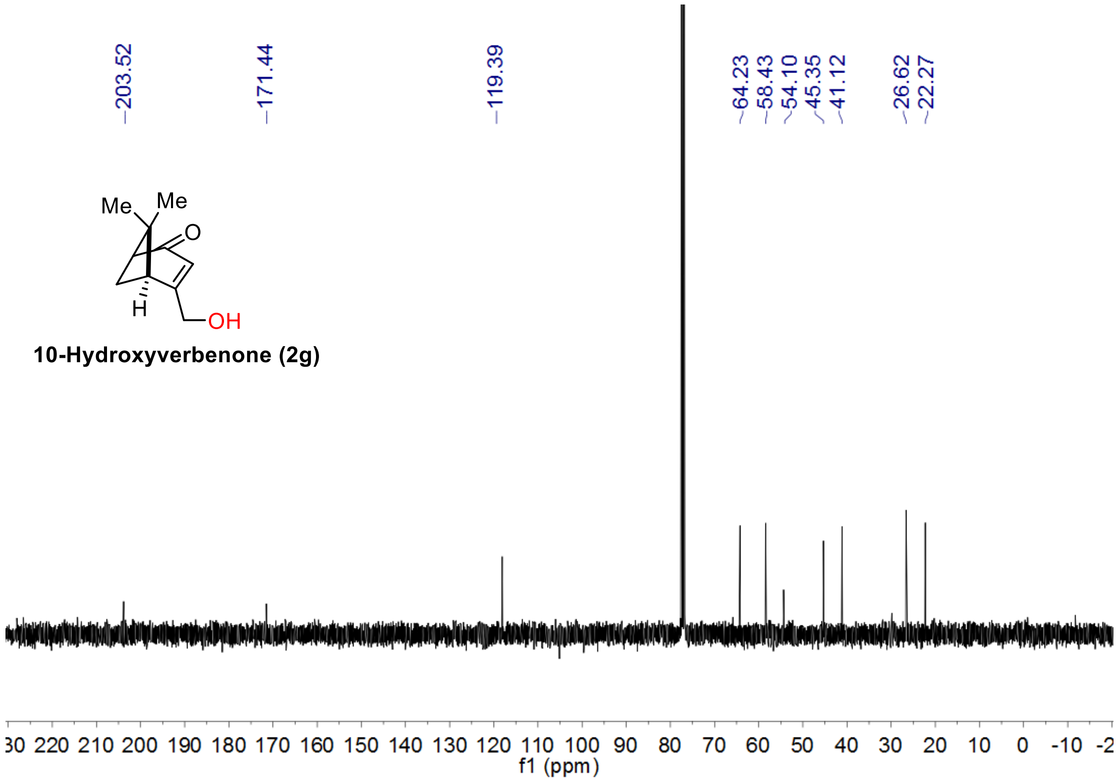

Supplementary Fig. 47. ^{13}C NMR spectrum of compound **2g (101 MHz, $CDCl_3$)**

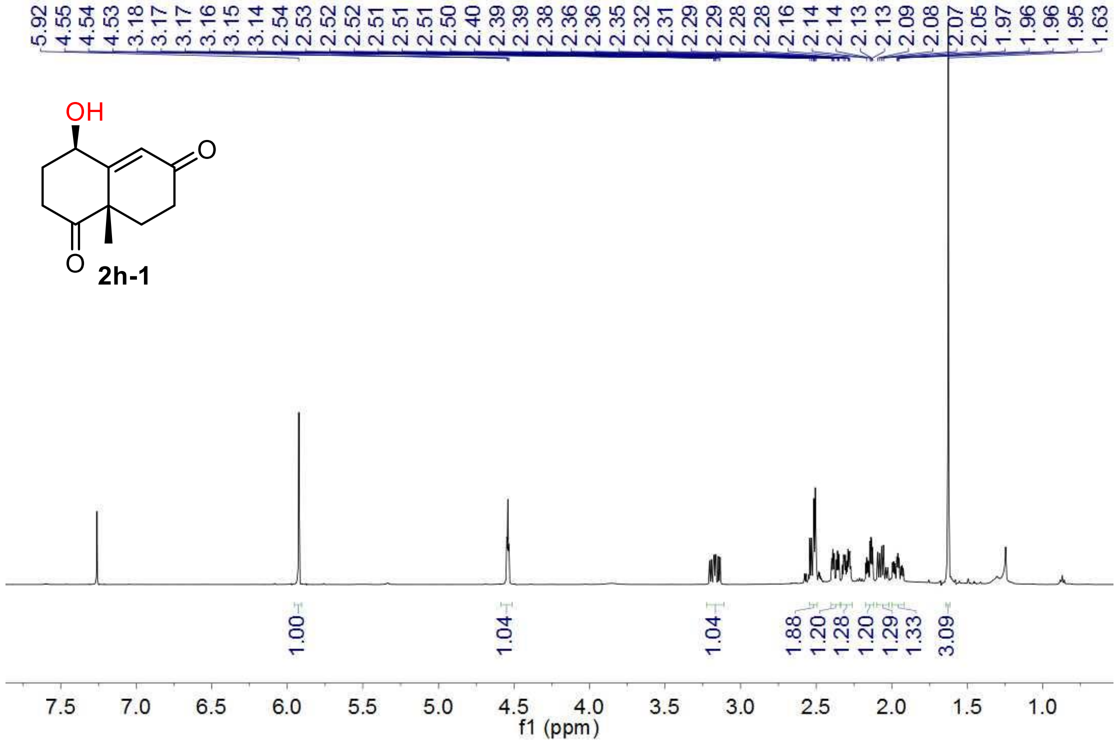

Supplementary Fig. 48. ^{1}H NMR spectrum of compound **2h-1 (500 MHz, CDCl$_3$)**

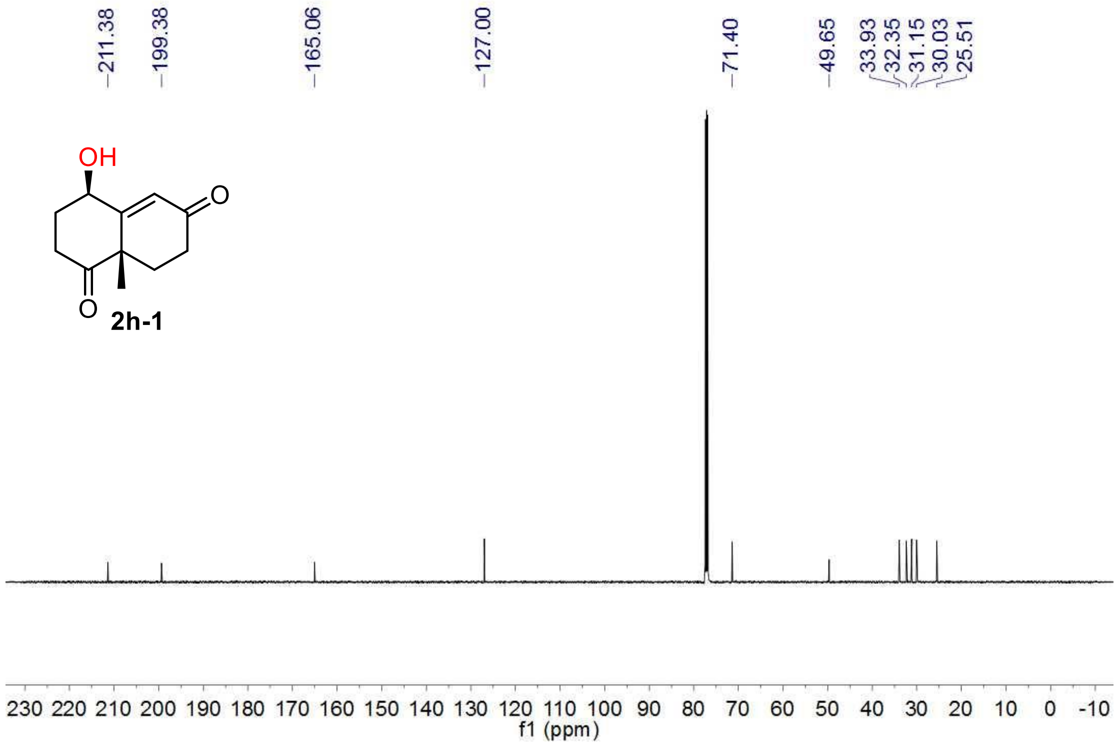

Supplementary Fig. 49. ^{13}C NMR spectrum of compound **2h-1 (126 MHz, CDCl$_3$)**

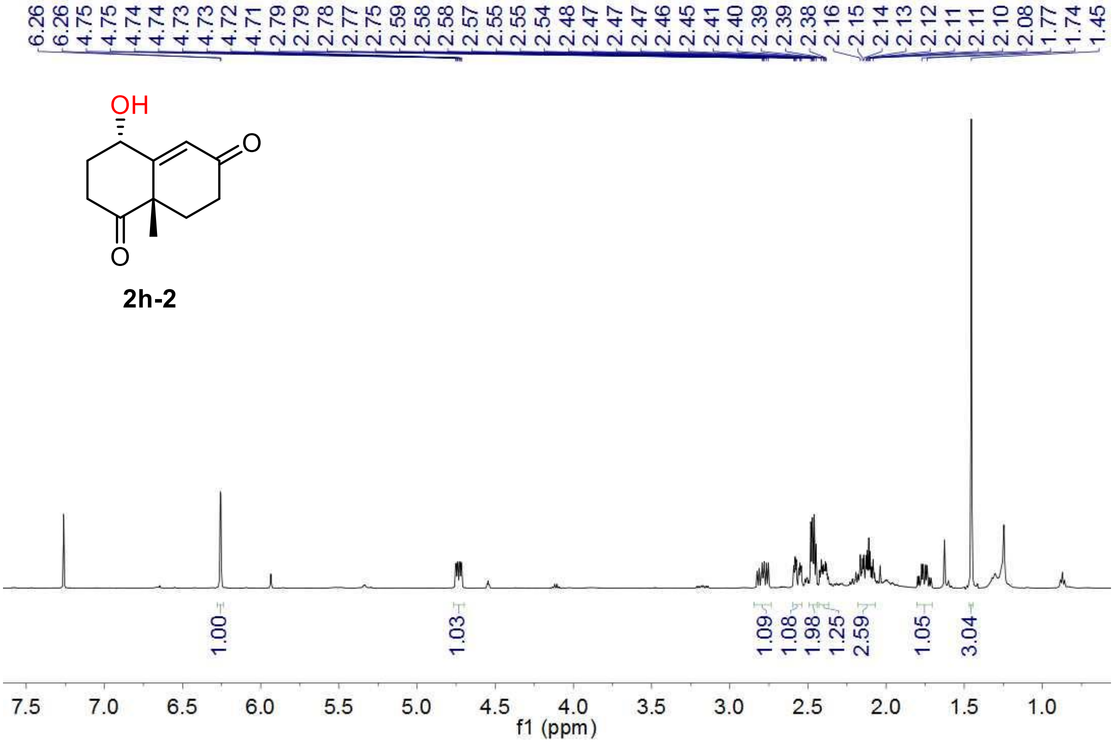

Supplementary Fig. 50. ^{1}H NMR spectrum of compound **2h-2 (500 MHz, $CDCl_3$)**

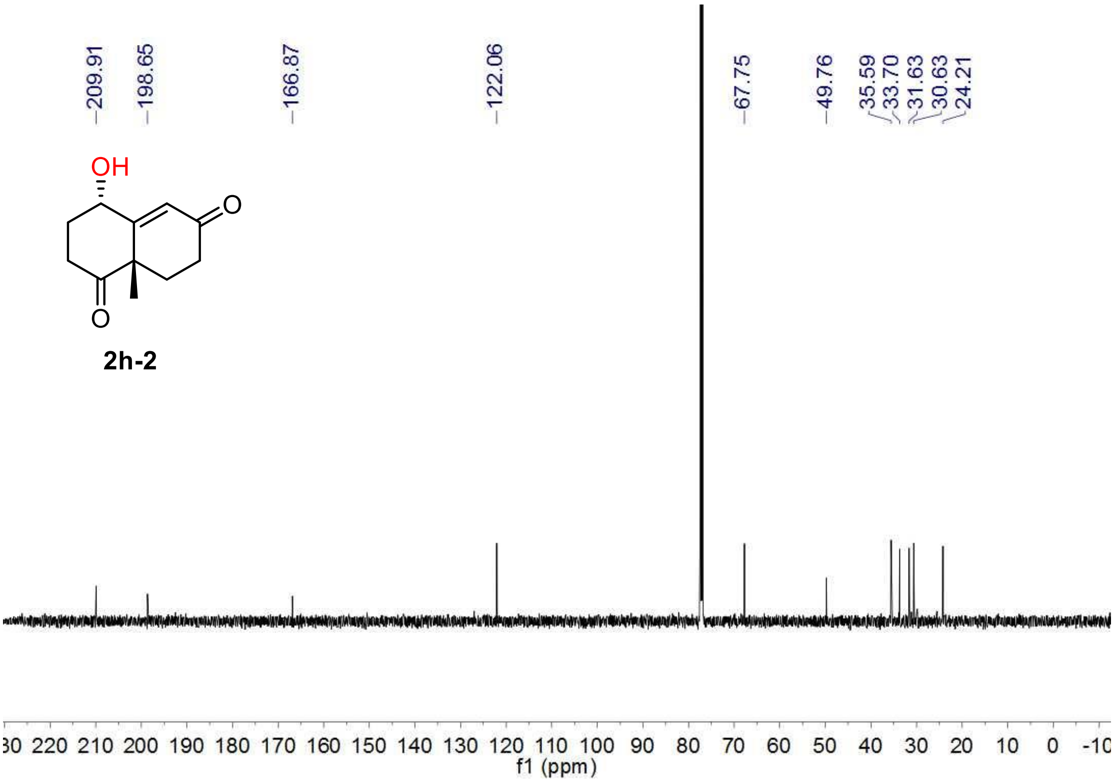

Supplementary Fig. 51. ^{13}C NMR spectrum of compound **2h-2 (126 MHz, $CDCl_3$)**

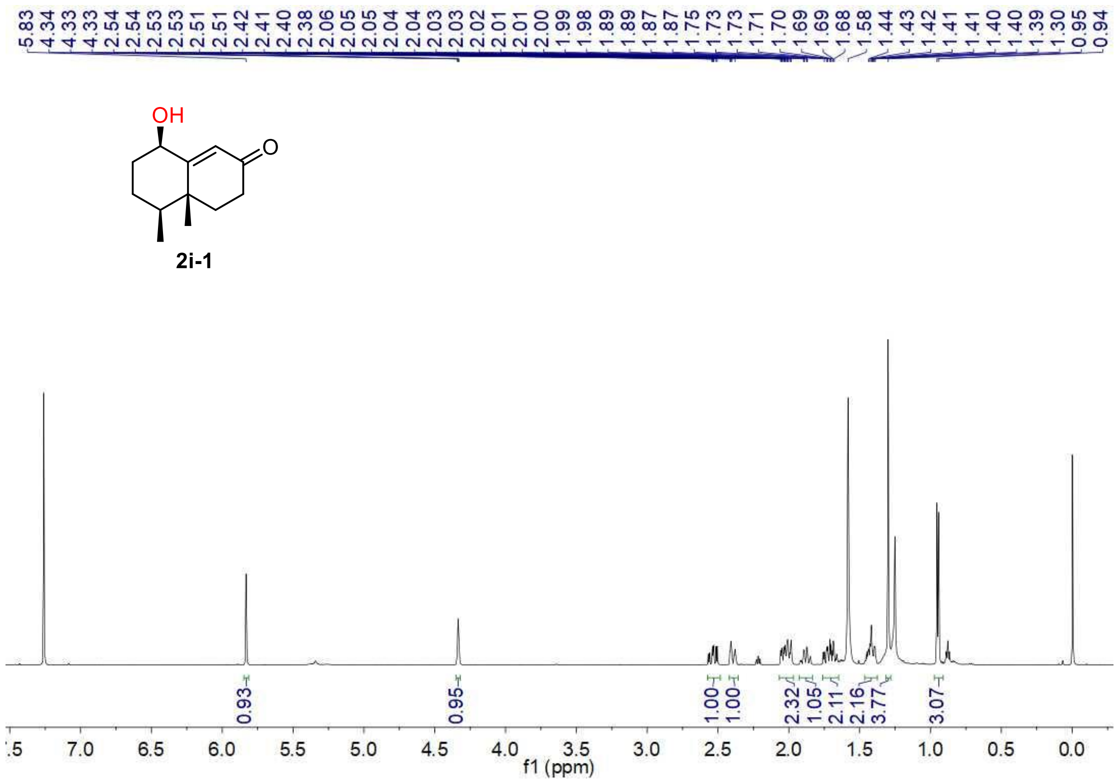

Supplementary Fig. 52. ^{1}H NMR spectrum of compound **2i-1 (600 MHz, CDCl3)**

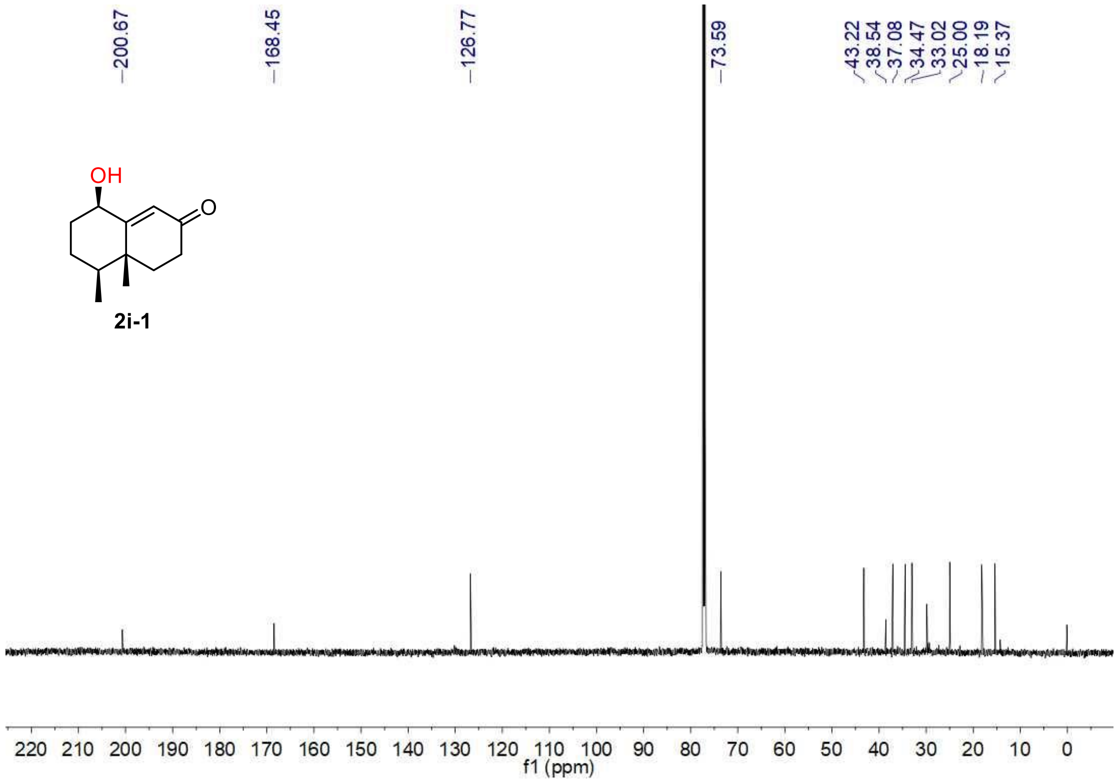

Supplementary Fig. 53. ^{13}C NMR spectrum of compound **2i-1 (151 MHz, CDCl3)**

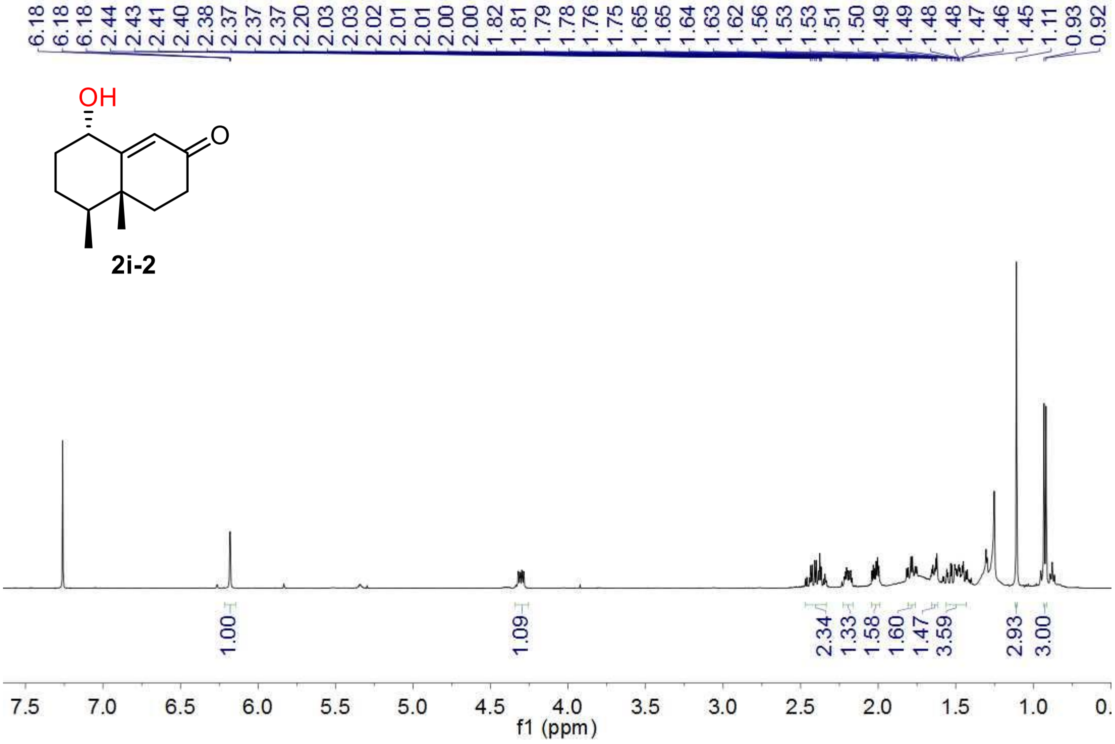

Supplementary Fig. 54. ^{1}H NMR spectrum of compound **2i-2 (500 MHz, CDCl₃)**

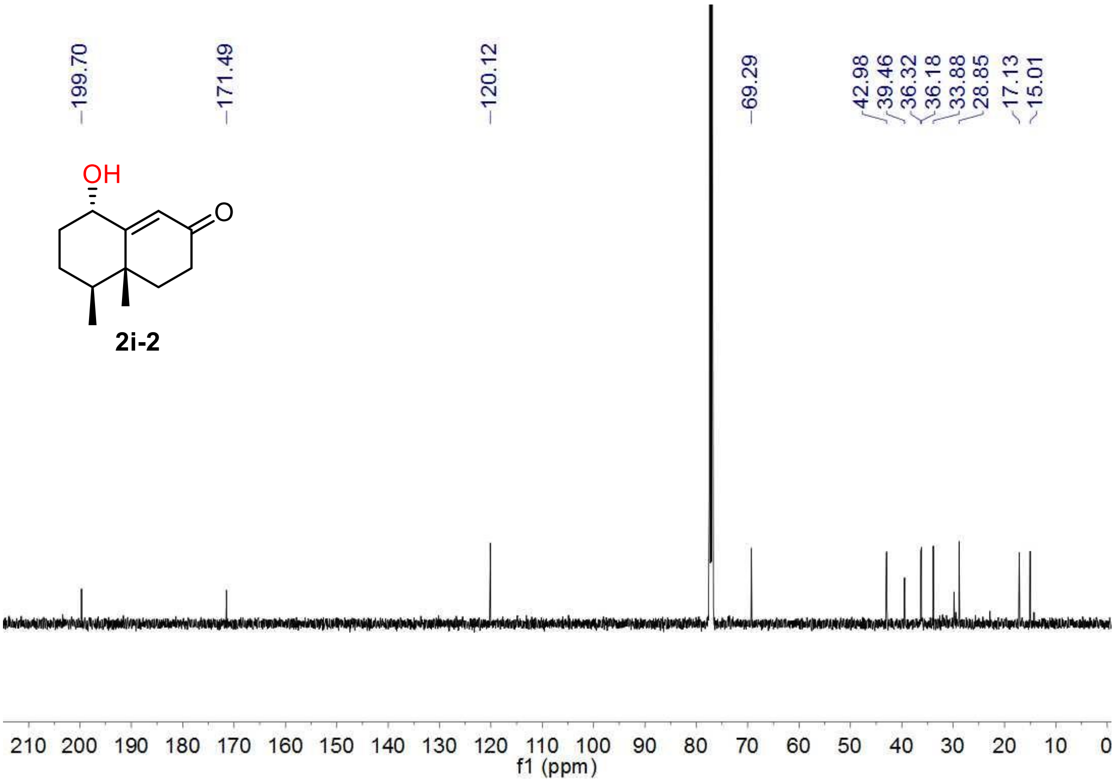

Supplementary Fig. 55. ^{13}C NMR spectrum of compound **2i-2 (126 MHz, CDCl₃)**

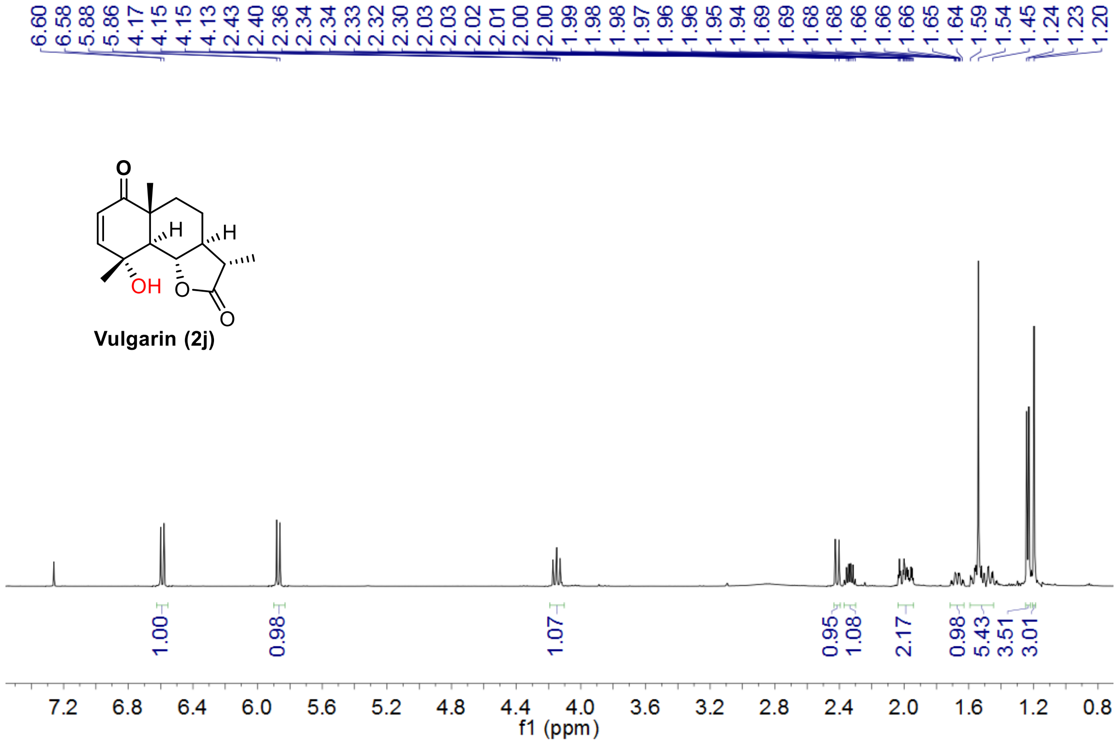

Supplementary Fig. 56. ^{1}H NMR spectrum of compound **2j (400 MHz, CDCl$_3$)**

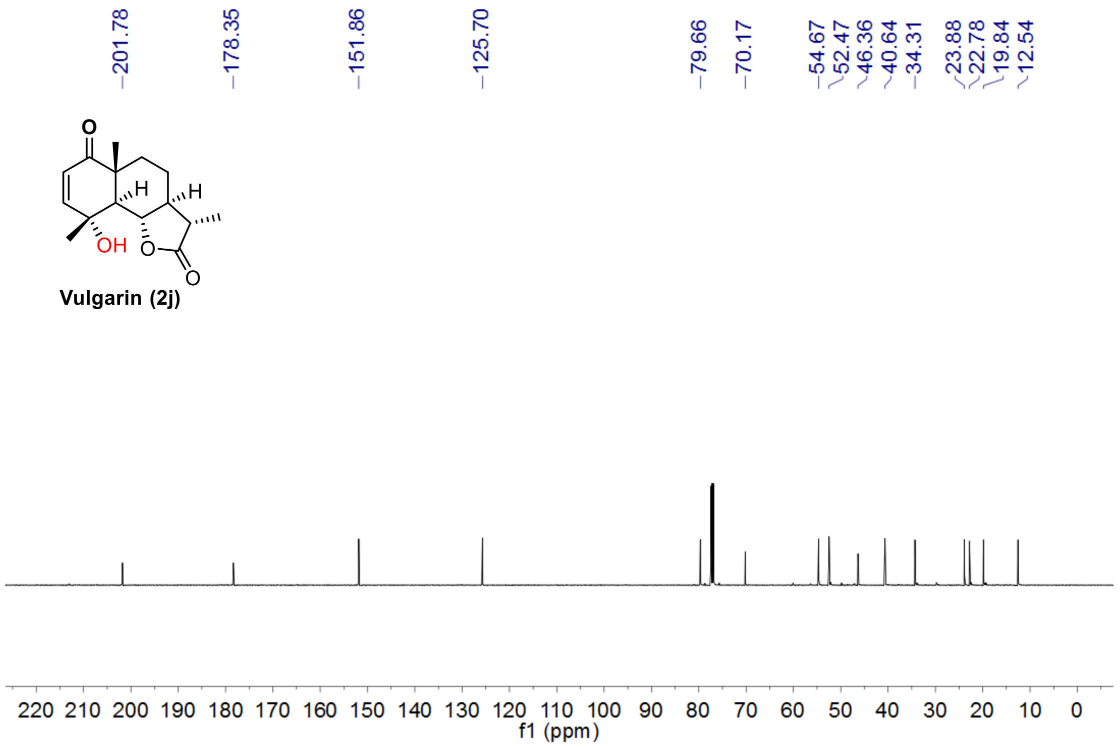

Supplementary Fig. 57. ^{13}C NMR spectrum of compound **2j (126 MHz, CDCl$_3$)**

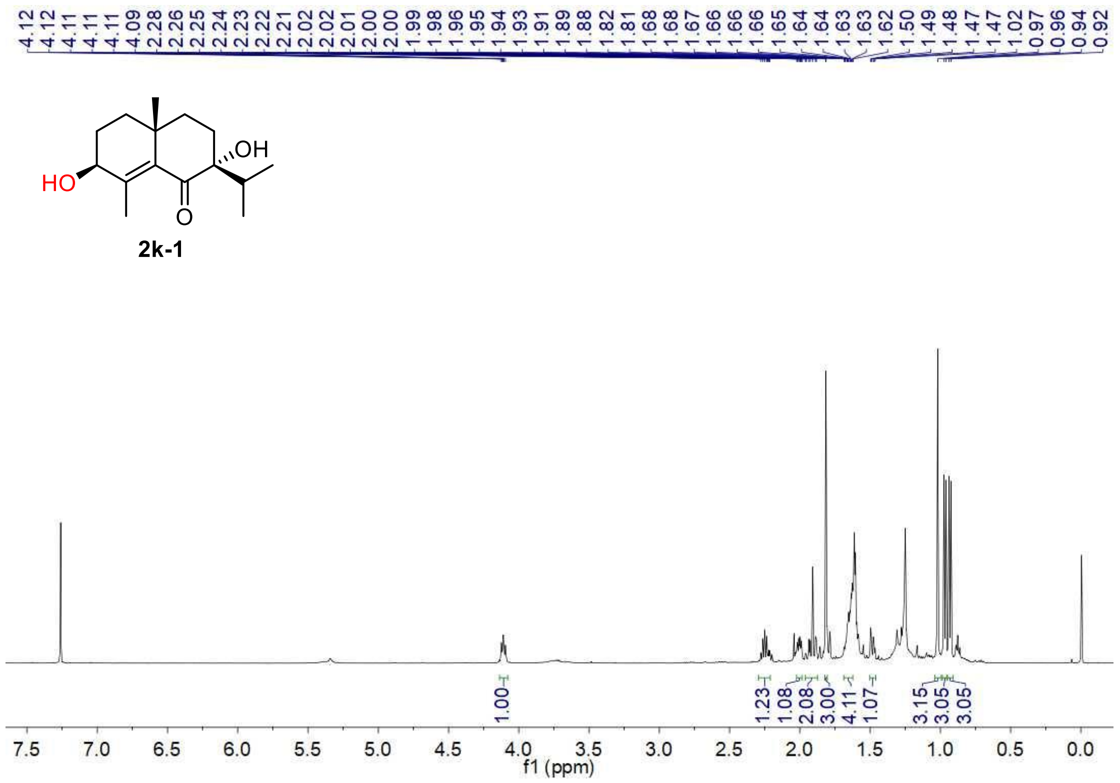

Supplementary Fig. 58. ^{1}H NMR spectrum of compound **2k-1 (500 MHz, $CDCl_3$)**

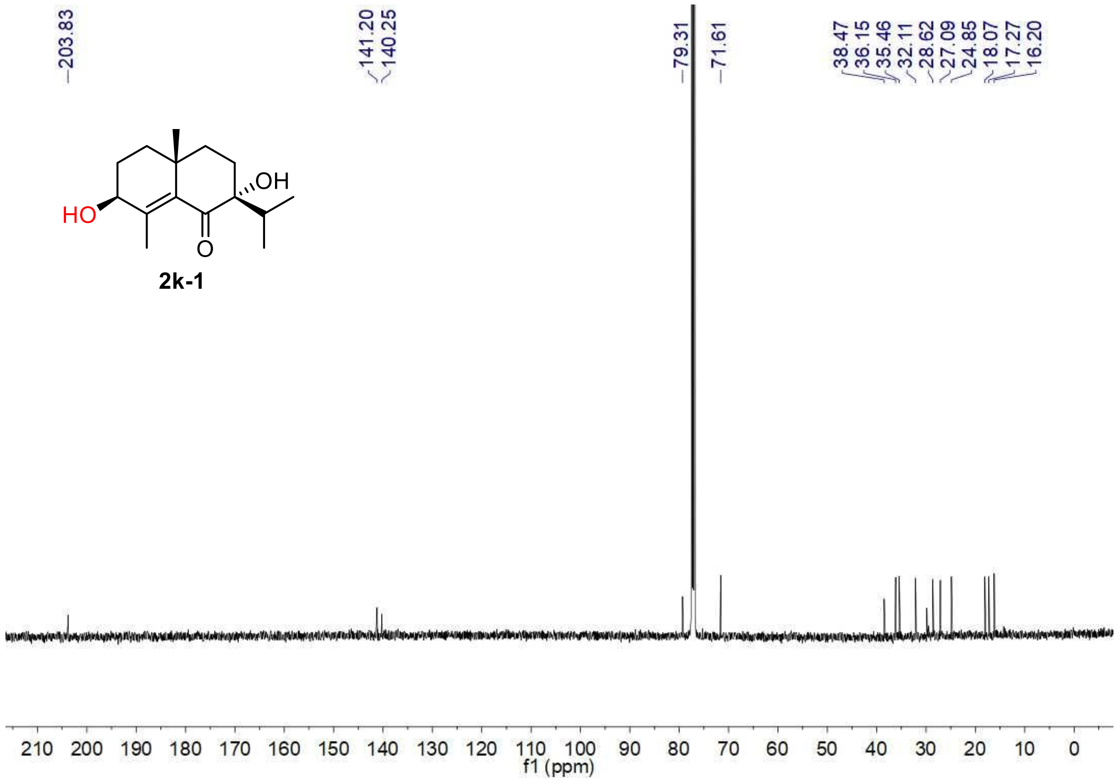

Supplementary Fig. 59. ^{13}C NMR spectrum of compound **2k-1 (126 MHz, $CDCl_3$)**

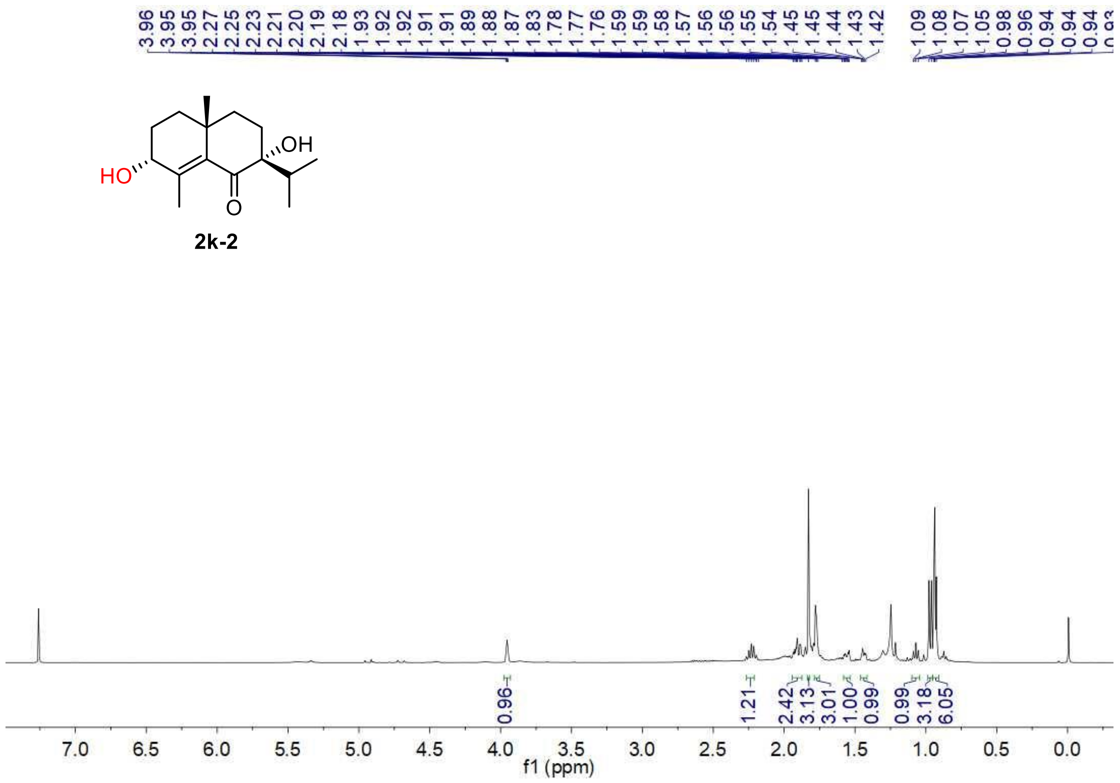

Supplementary Fig. 60. ^{1}H NMR spectrum of compound **2k-2 (400 MHz, $CDCl_3$)**

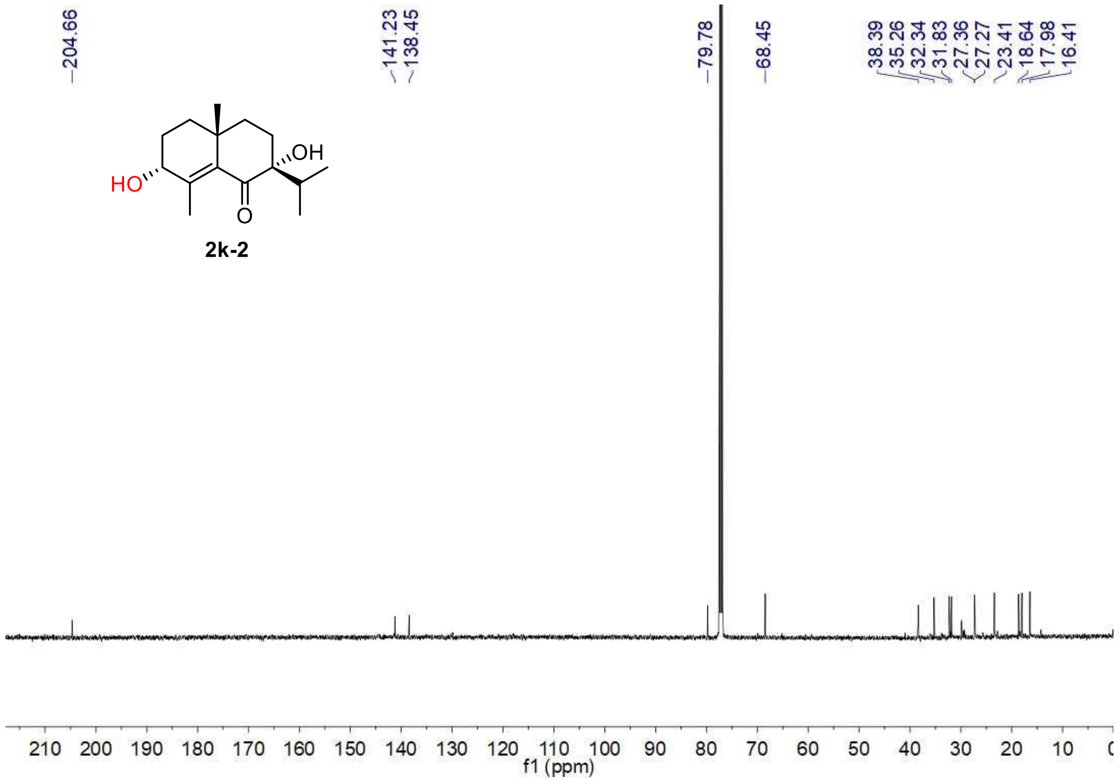

Supplementary Fig. 61. ^{13}C NMR spectrum of compound **2k-2 (126 MHz, $CDCl_3$)**

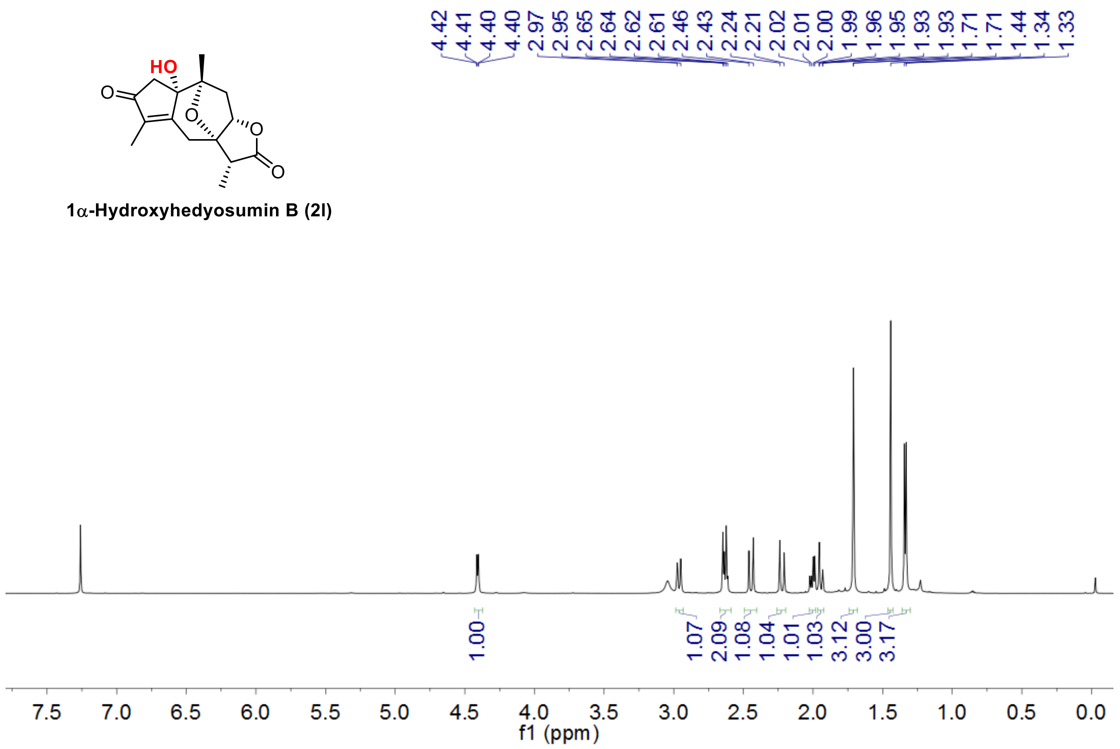

Supplementary Fig. 62. ^{1}H NMR spectrum of compound **2l (600 MHz, CDCl$_3$)**

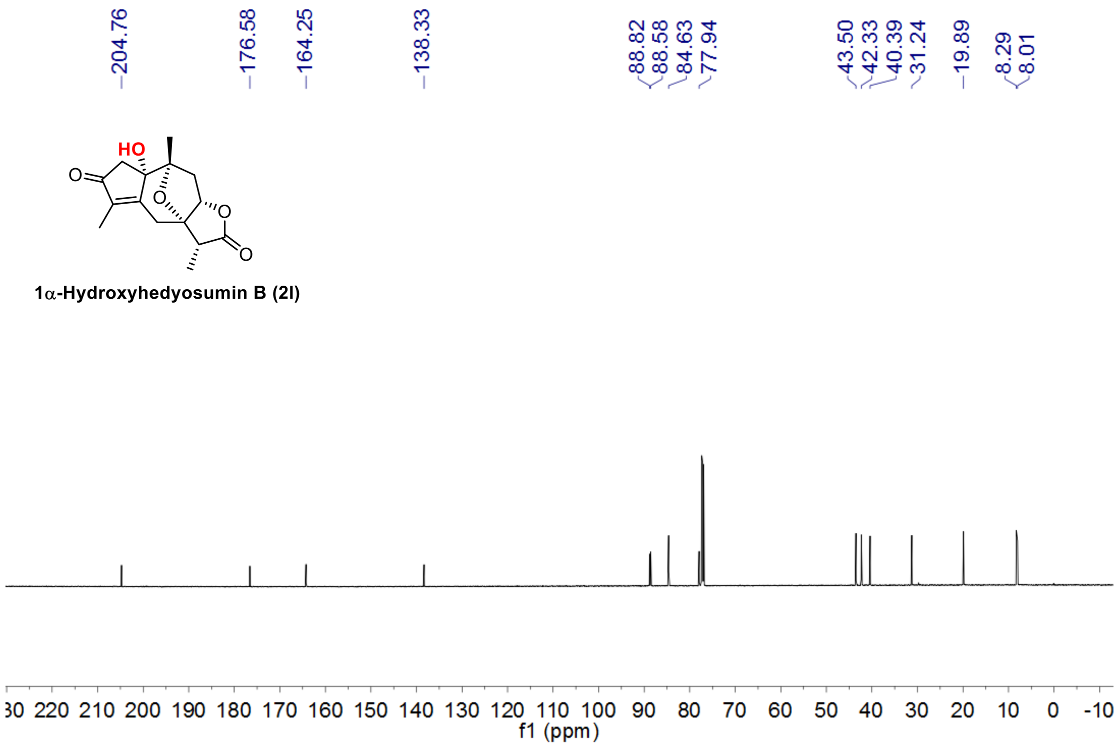

Supplementary Fig. 63. ^{13}C NMR spectrum of compound **2l (151 MHz, CDCl$_3$)**

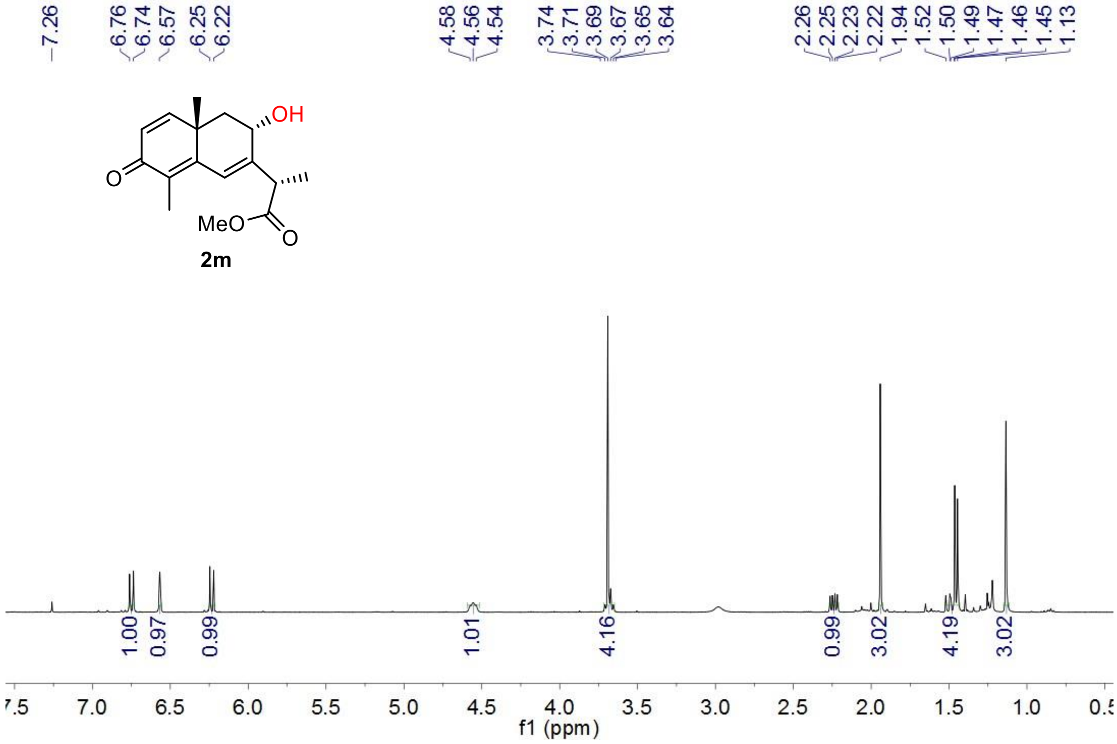

Supplementary Fig. 64. ^{1}H NMR spectrum of compound **2m (400 MHz, CDCl₃)**

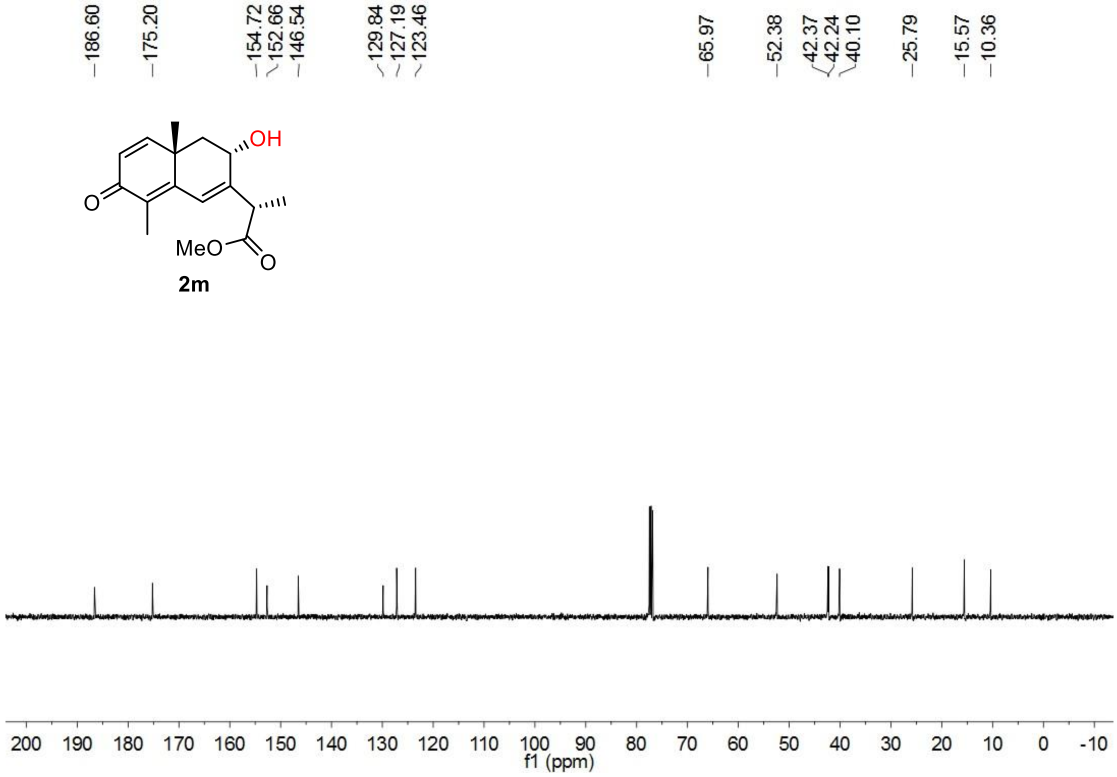

Supplementary Fig. 65. ^{13}C NMR spectrum of compound **2m (100 MHz, CDCl₃)**

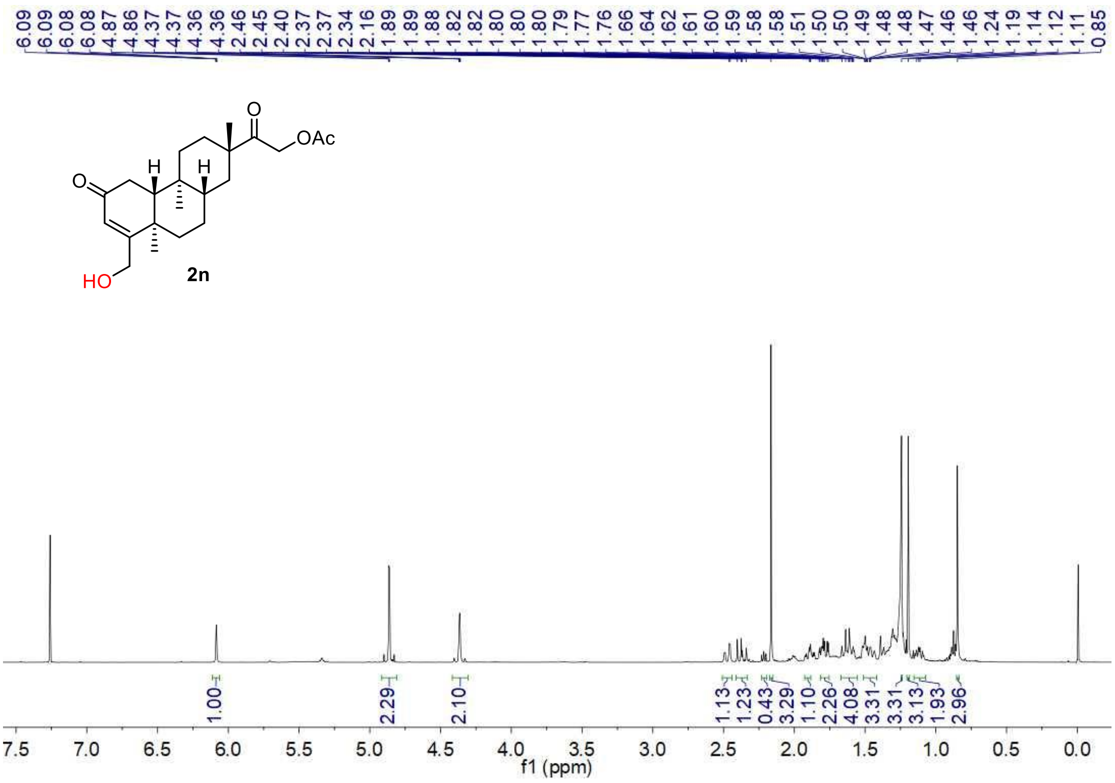

Supplementary Fig. 66. ^{1}H NMR spectrum of compound **2n (500 MHz, CDCl$_3$)**

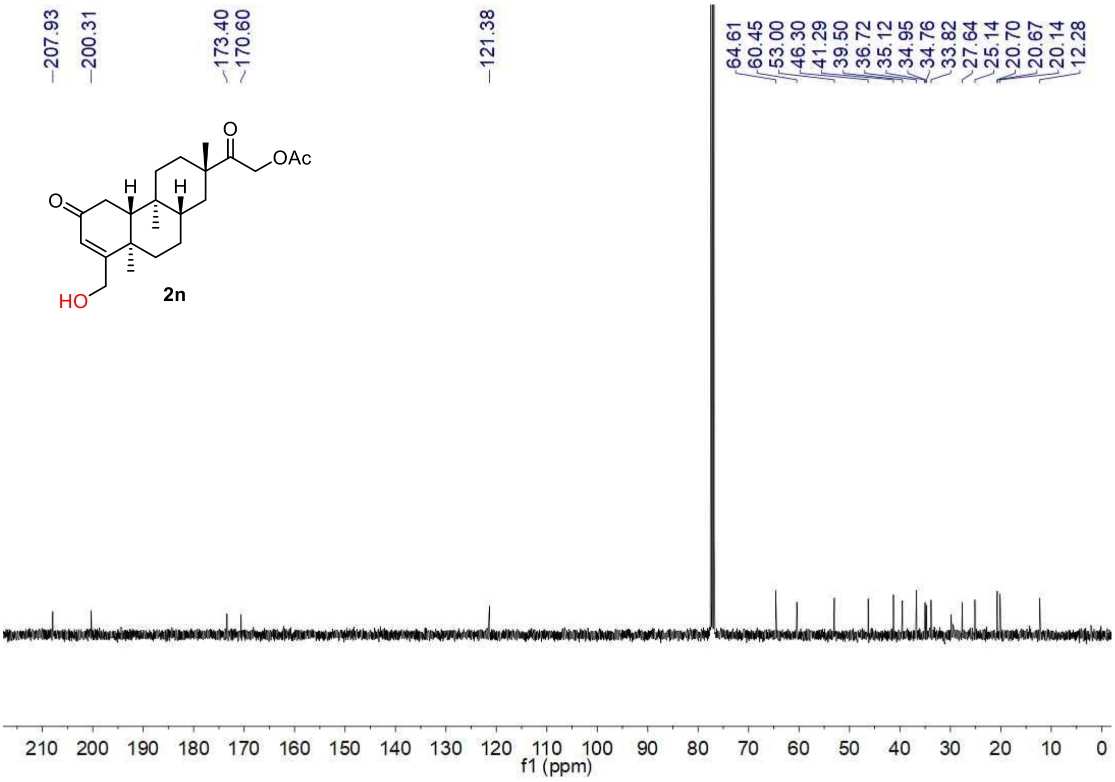

Supplementary Fig. 67. ^{13}C NMR spectrum of compound **2n (126 MHz, CDCl$_3$)**

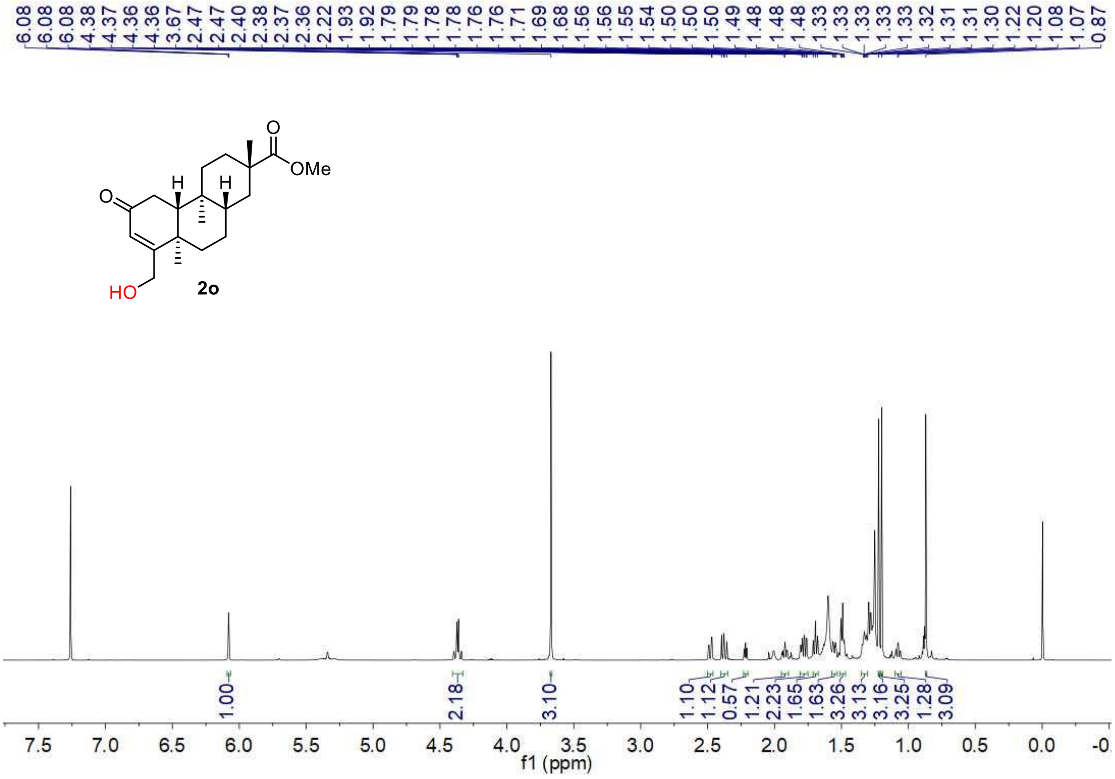

Supplementary Fig. 68. ^{1}H NMR spectrum of compound **2o (800 MHz, $CDCl_3$)**

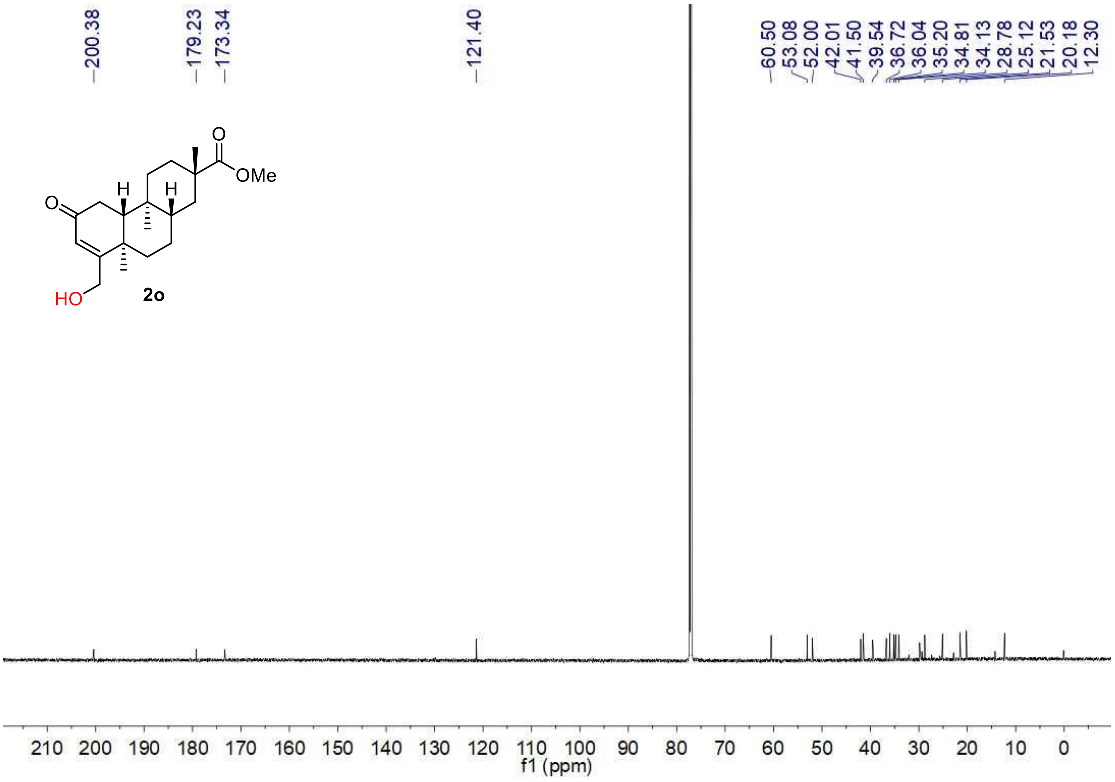

Supplementary Fig. 69. ^{13}C NMR spectrum of compound **2o (201 MHz, $CDCl_3$)**

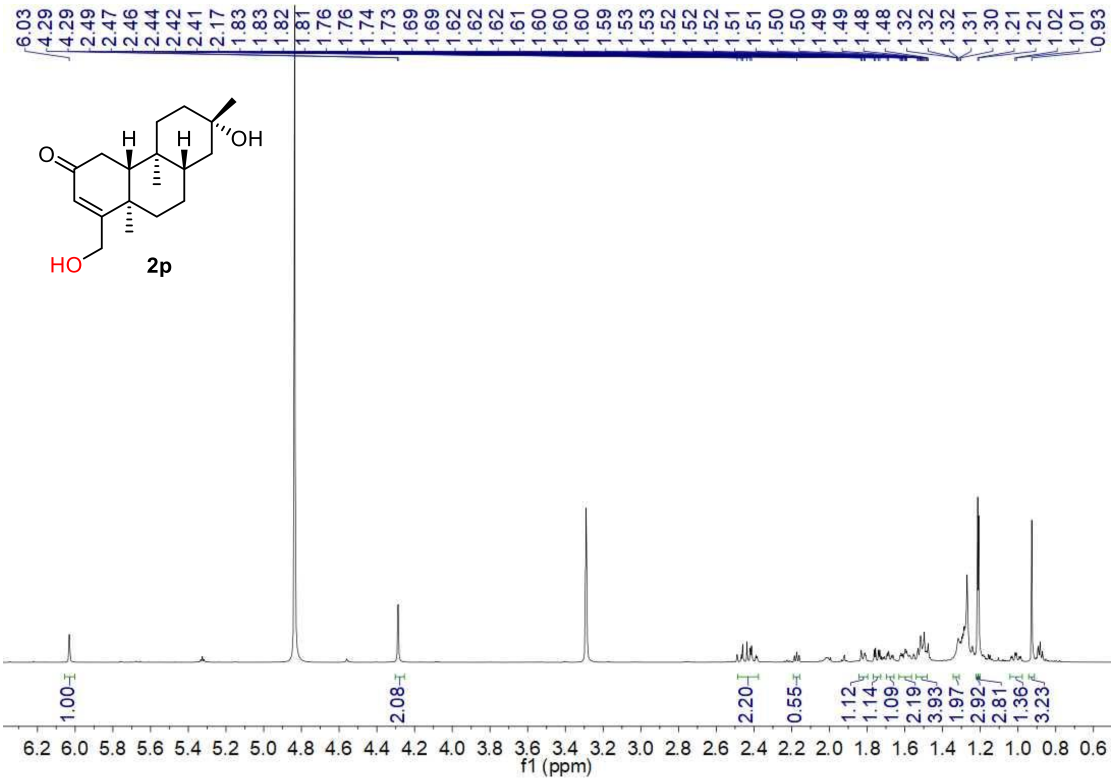

Supplementary Fig. 70. ^{1}H NMR spectrum of compound **2p (600 MHz, CD_3OD)**

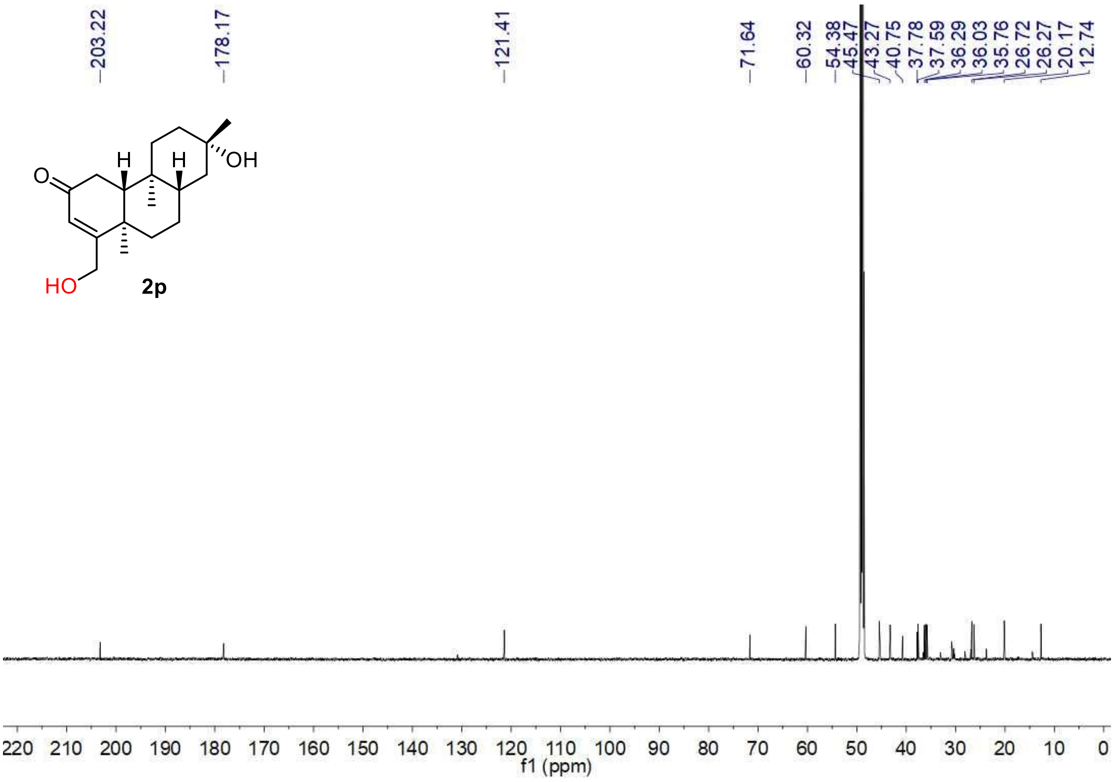

Supplementary Fig. 71. ^{13}C NMR spectrum of compound **2p (151 MHz, CD_3OD)**

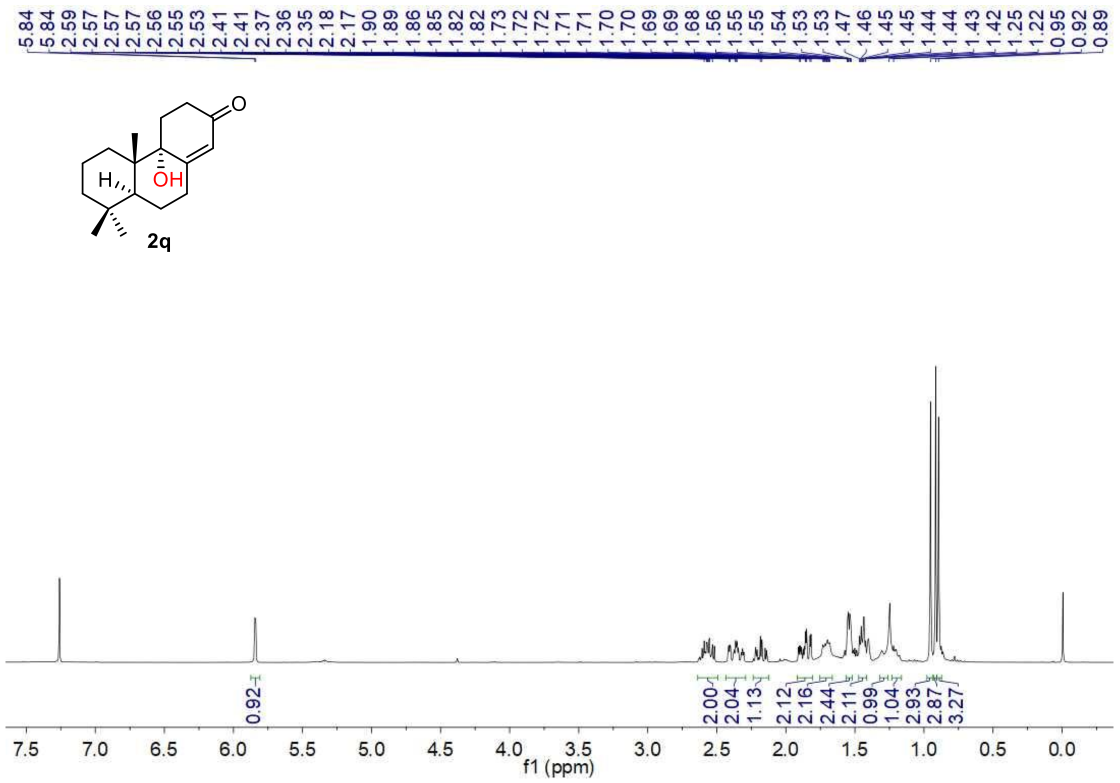

Supplementary Fig. 72. ^{1}H NMR spectrum of compound **2q (400 MHz, CDCl$_3$)**

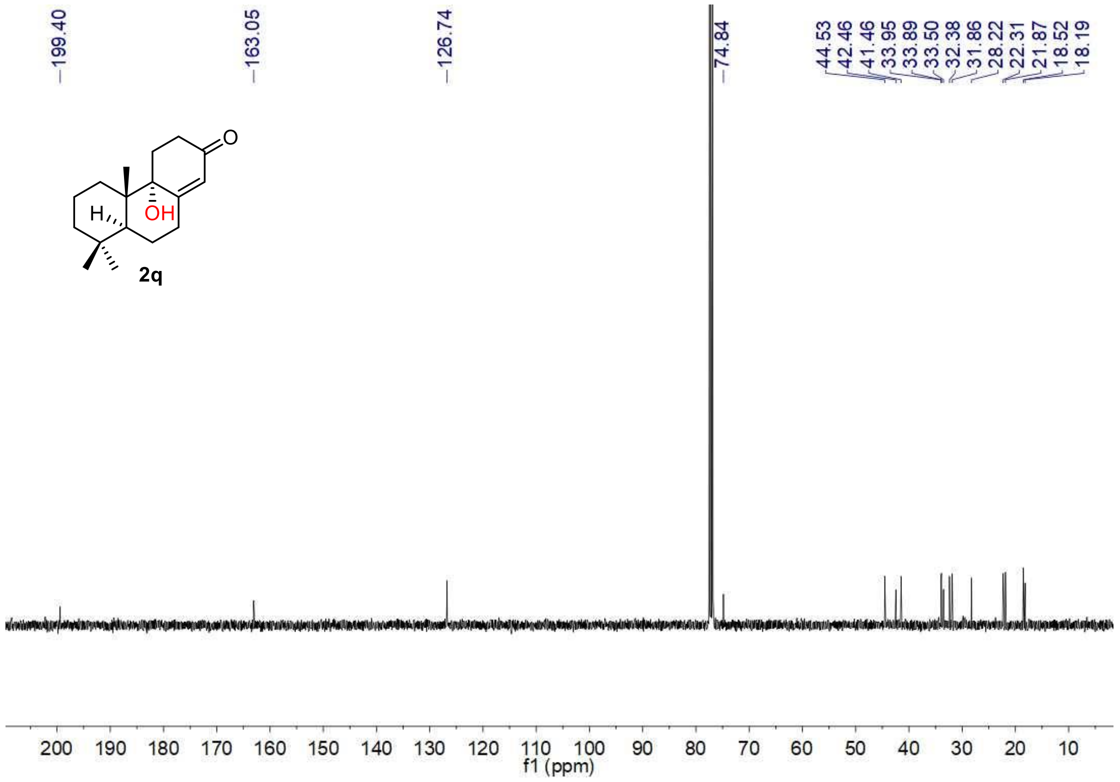

Supplementary Fig. 73. ^{13}C NMR spectrum of compound **2q (101 MHz, CDCl$_3$)**

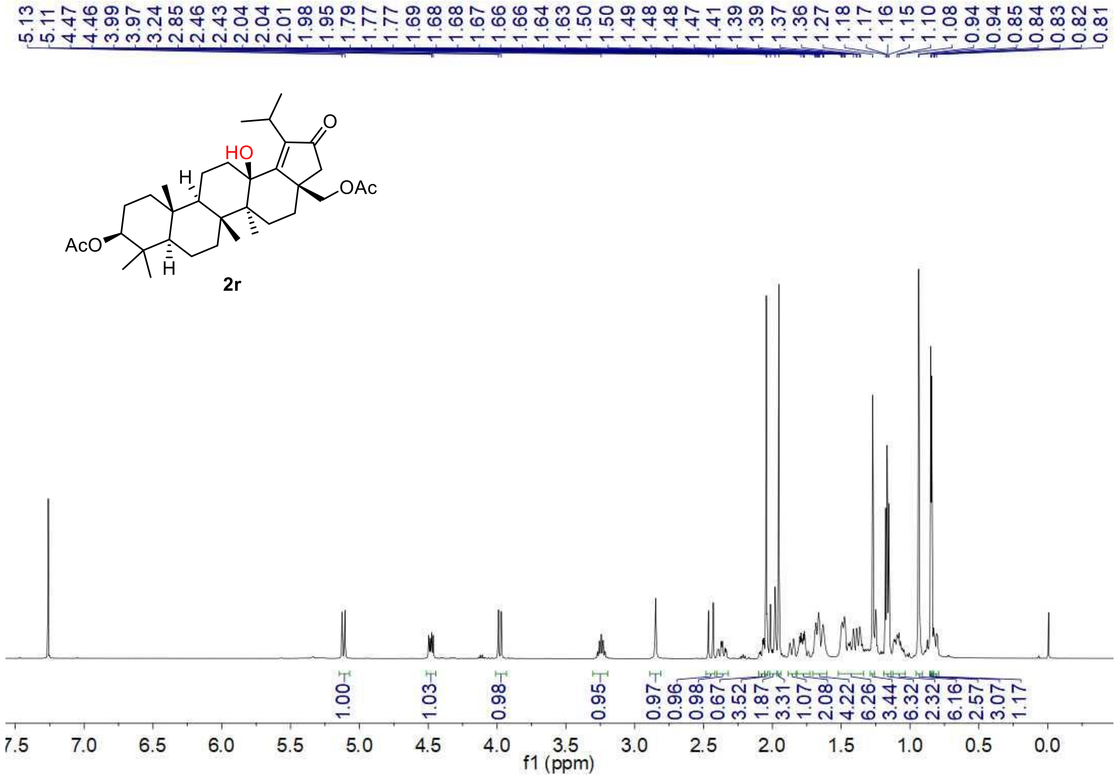

Supplementary Fig. 74. ^{1}H NMR spectrum of compound **2r (500 MHz, CDCl$_3$)**

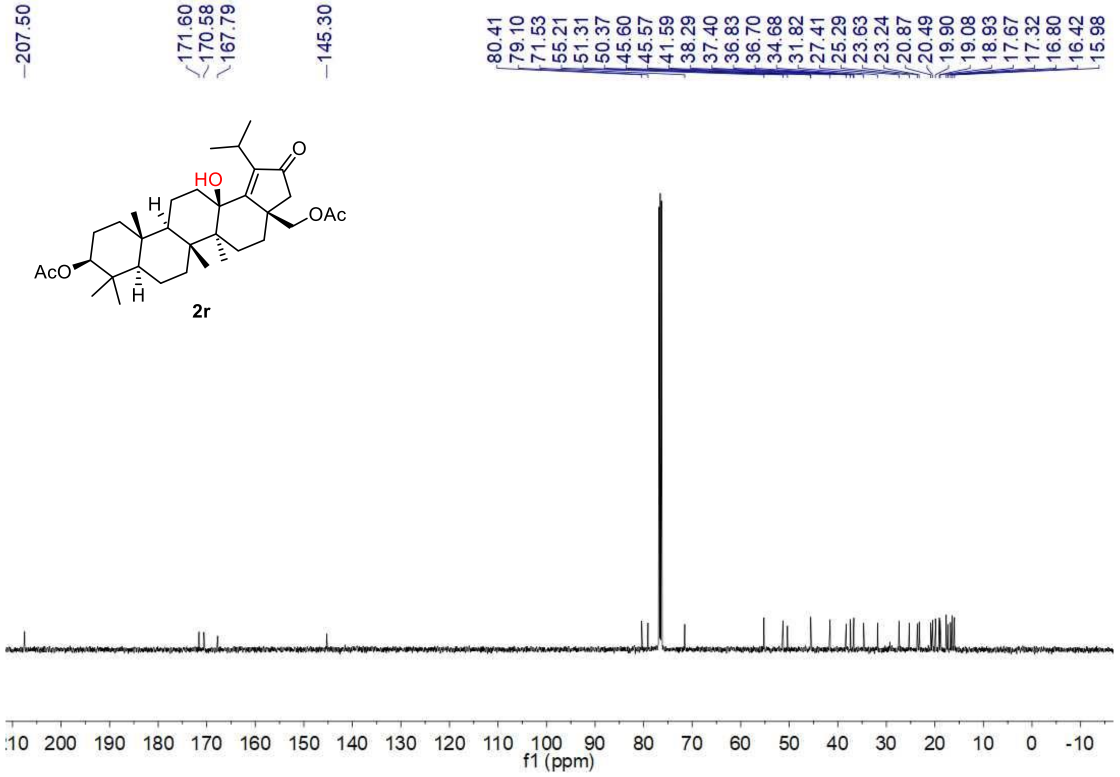

Supplementary Fig. 75. ^{13}C NMR spectrum of compound **2r (126 MHz, CDCl$_3$)**

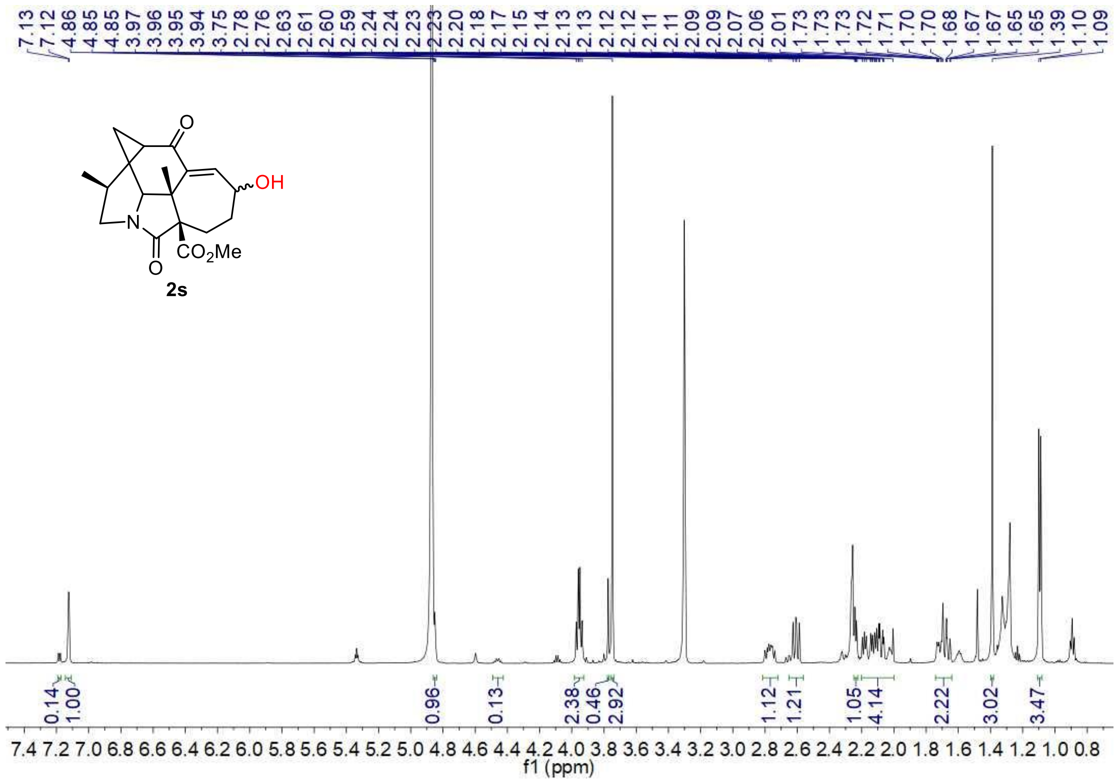

Supplementary Fig. 76. ^{1}H NMR spectrum of compound **2s (**mixture of a pair of diastereomers**; 600 MHz, CD3OD)**

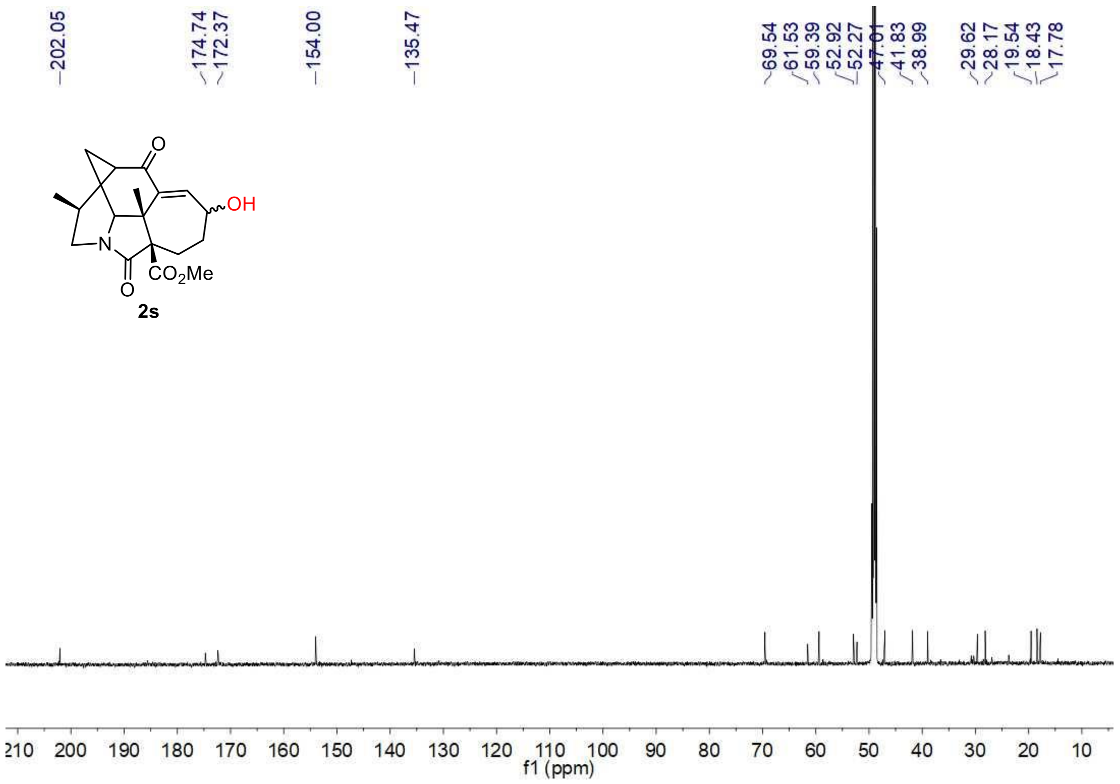

Supplementary Fig. 77. ^{13}C NMR spectrum of compound **2s (**mixture of a pair of diastereomers**; 151 MHz, CD3OD)**

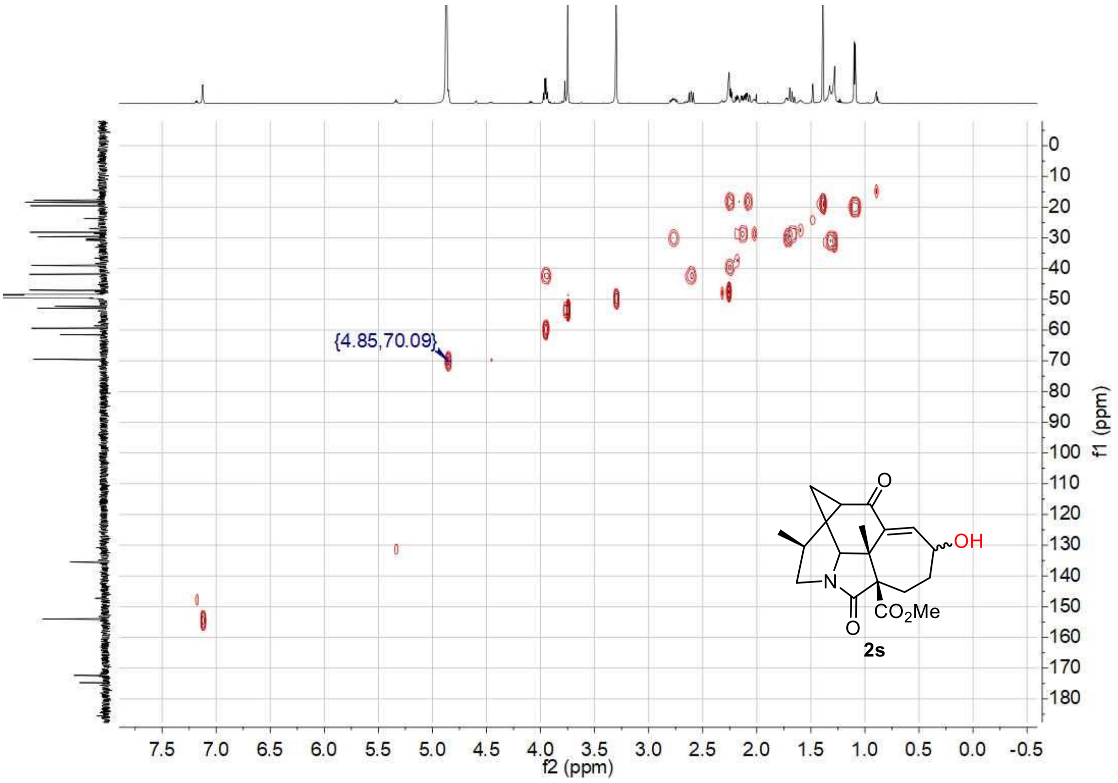

Supplementary Fig. 78. HSQC spectrum of compound **2s** (mixture of a pair of diastereomers) in **CD3OD**

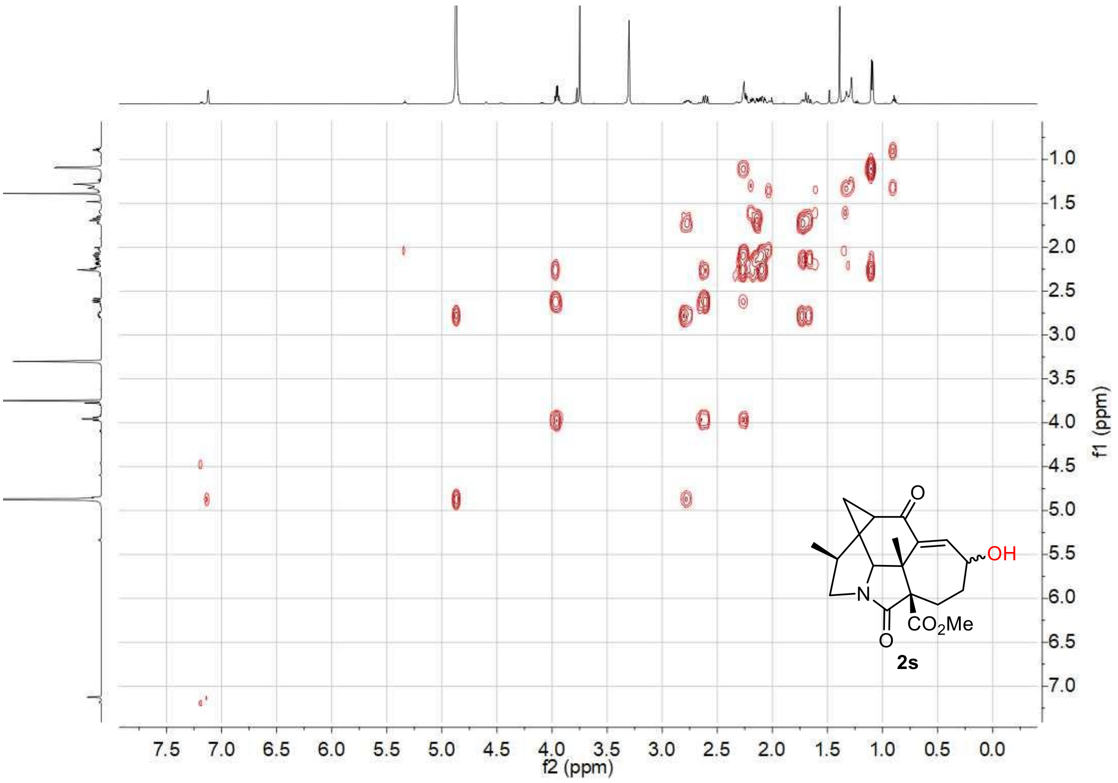

Supplementary Fig. 79. ^{1}H–^{1}H COSY spectrum of compound **2s** (mixture of a pair of diastereomers) in **CD3OD**

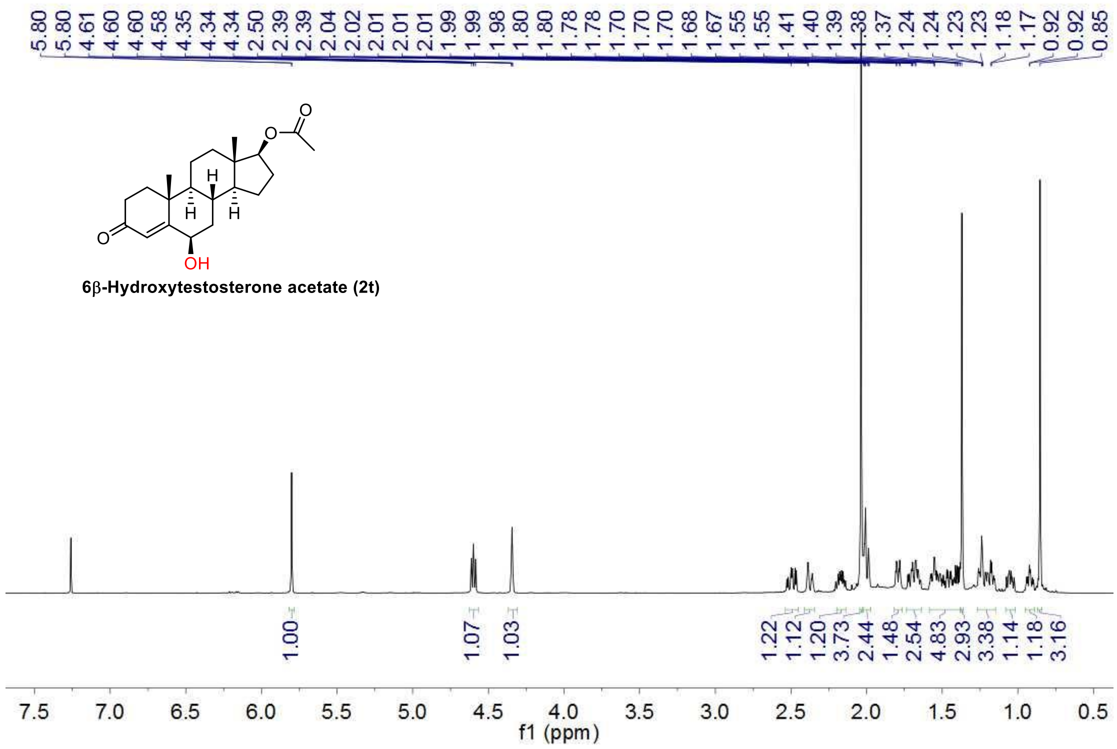

Supplementary Fig. 80. ^{1}H NMR spectrum of compound **2t (600 MHz, CDCl$_3$)**

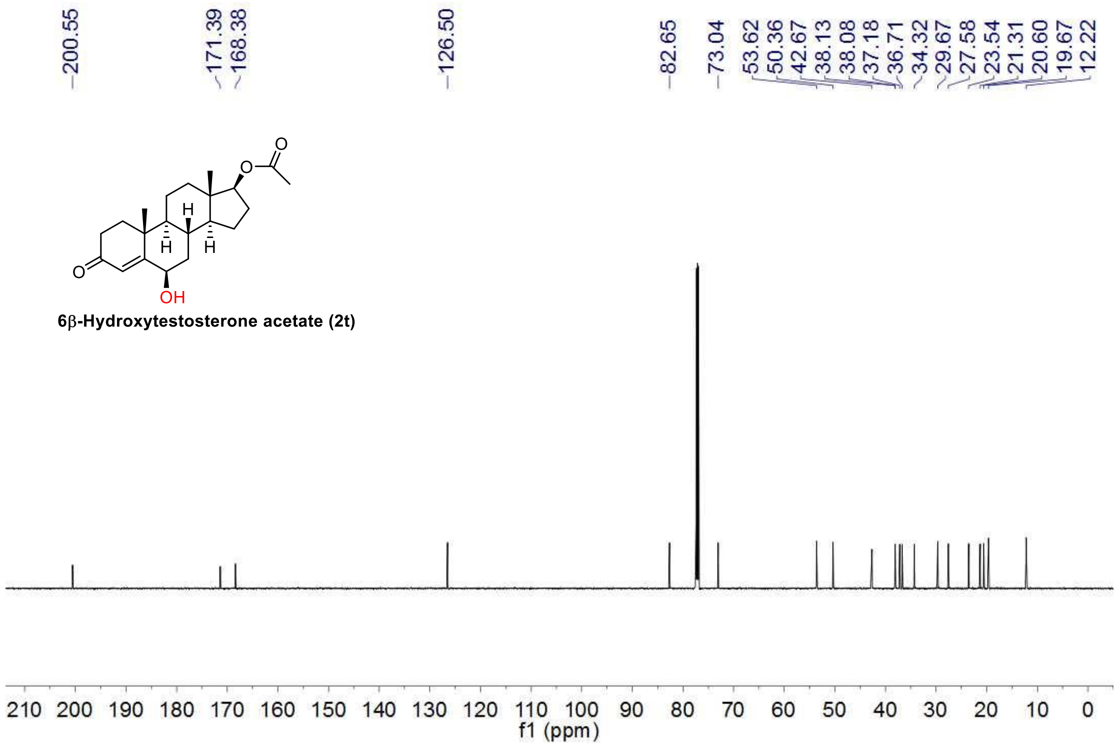

Supplementary Fig. 81. ^{13}C NMR spectrum of compound **2t (151 MHz, CDCl$_3$)**

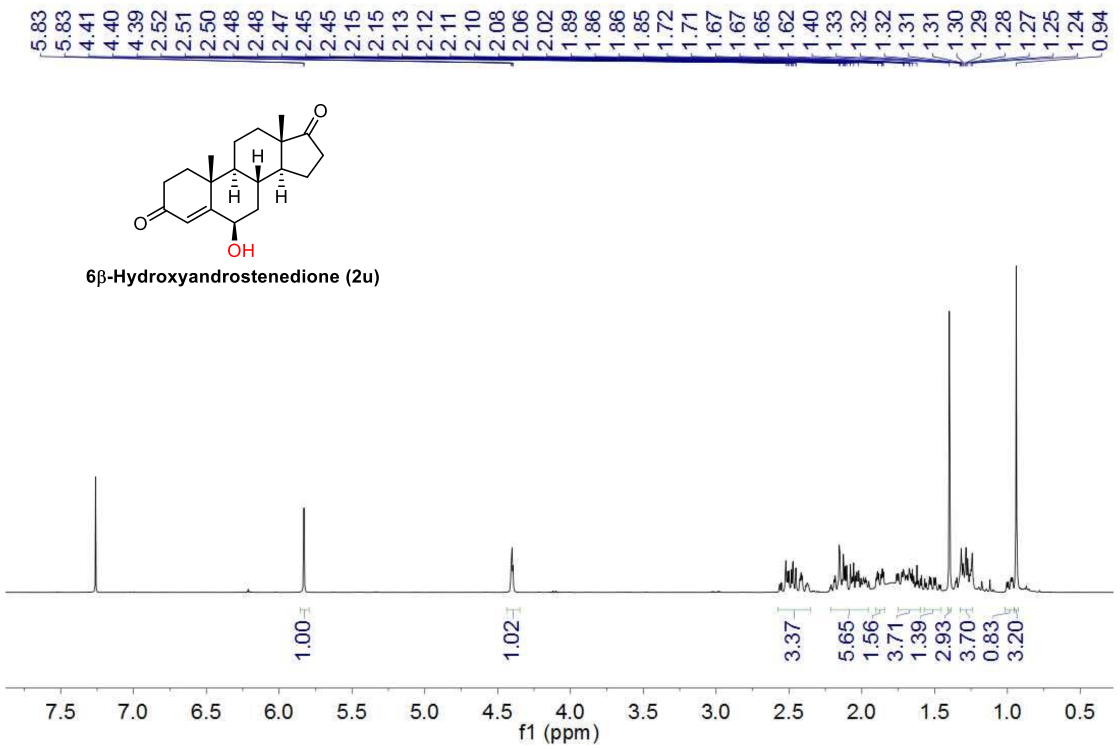

Supplementary Fig. 82. ^{1}H NMR spectrum of compound **2u (400 MHz, CDCl3)**

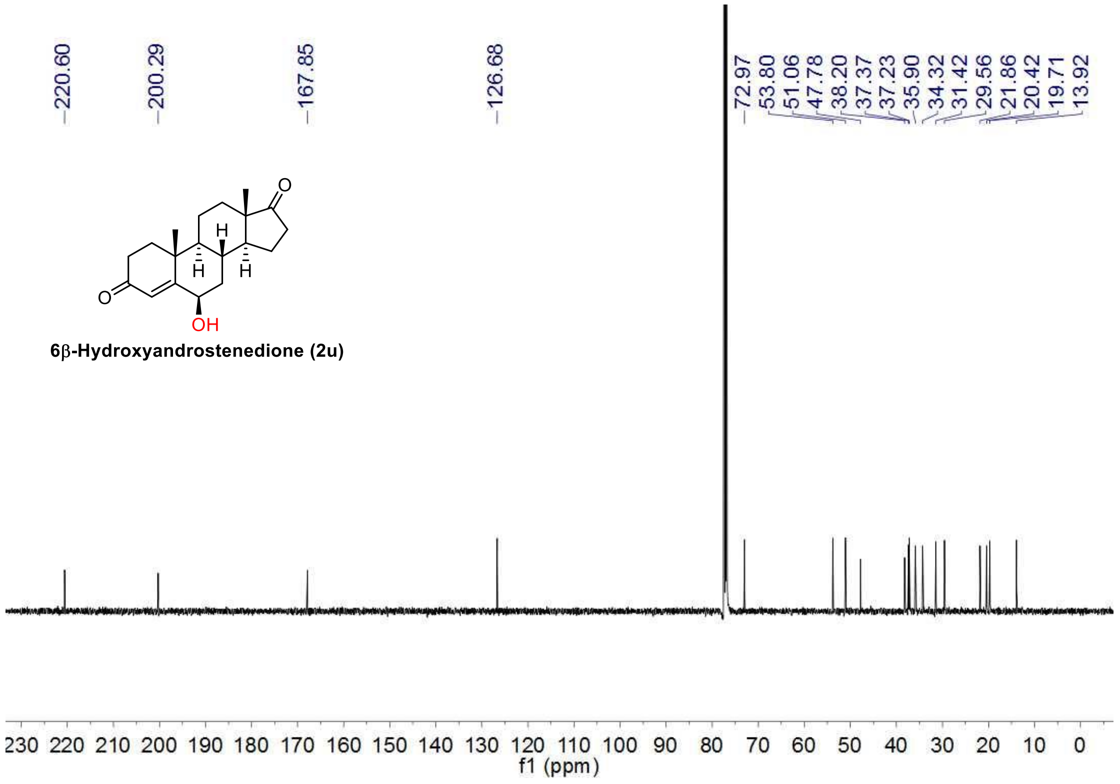

Supplementary Fig. 83. ^{13}C NMR spectrum of compound **2u (126 MHz, CDCl3)**

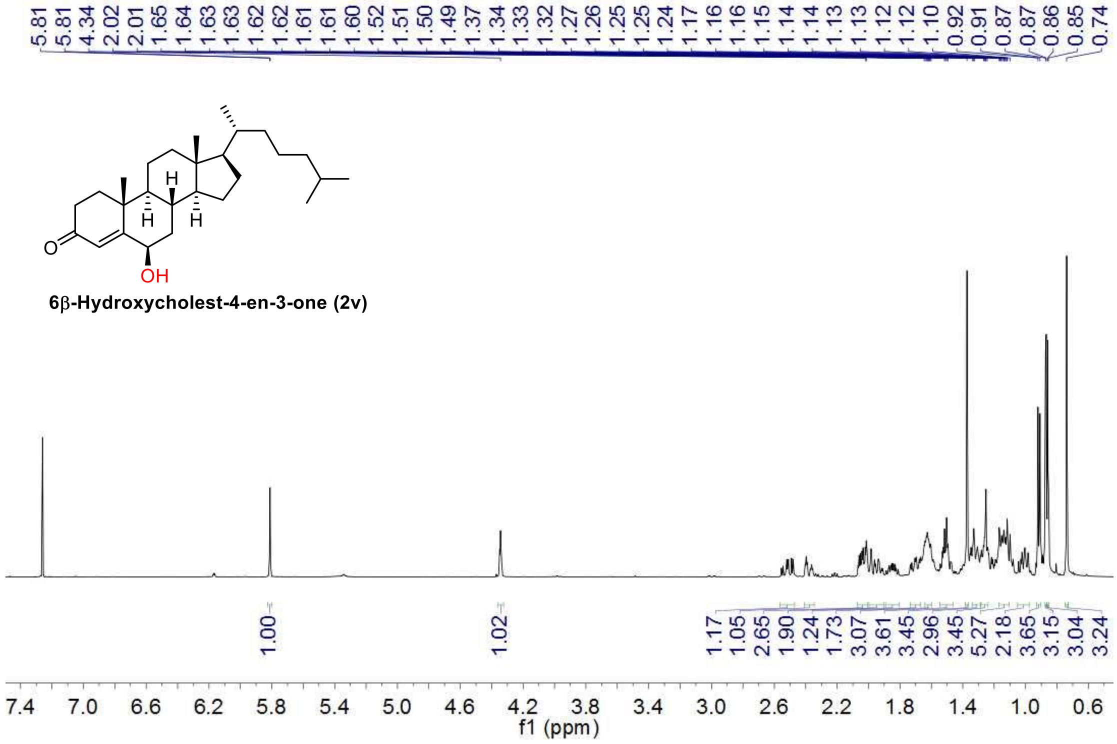

Supplementary Fig. 84. ^{1}H NMR spectrum of compound **2v (500 MHz, $CDCl_3$)**

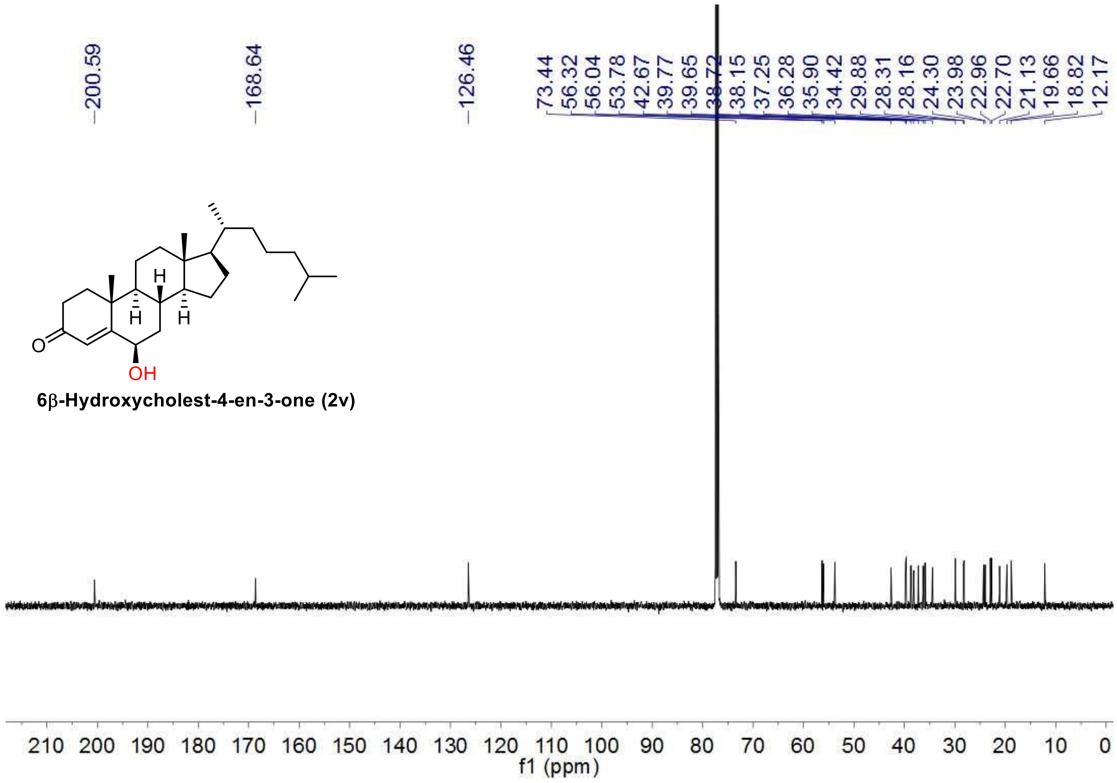

Supplementary Fig. 85. ^{13}C NMR spectrum of compound **2v (126 MHz, $CDCl_3$)**

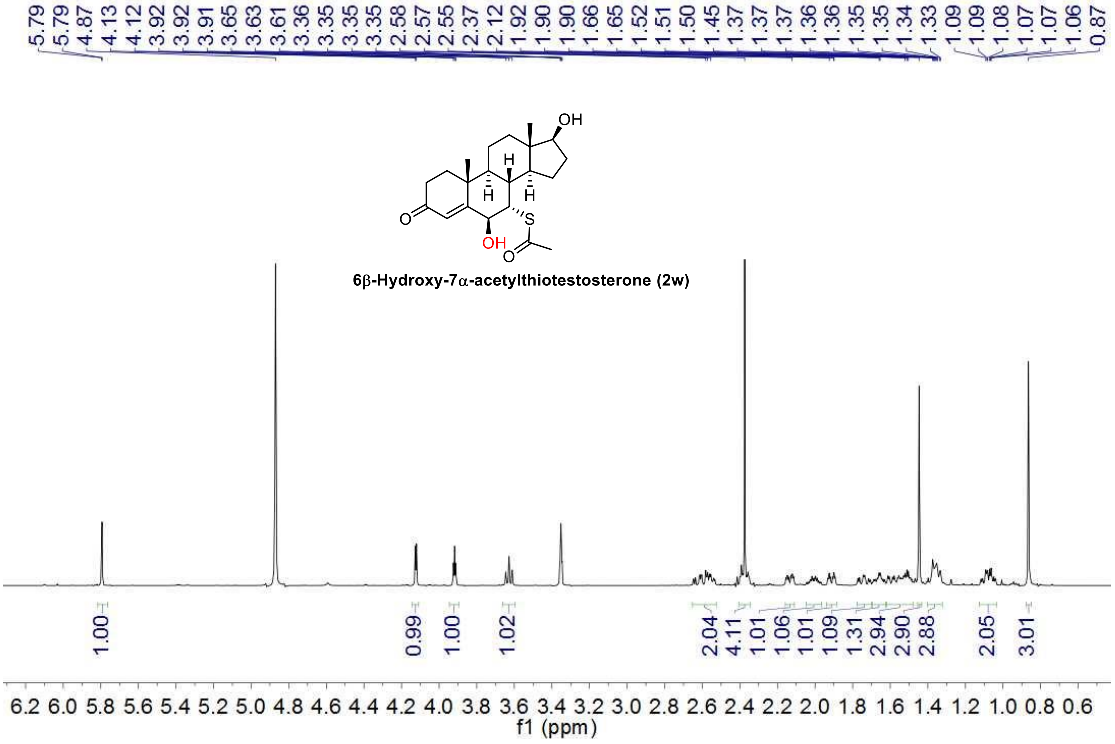

Supplementary Fig. 86. ^{1}H NMR spectrum of compound **2w (500 MHz, CD$_3$OD)**

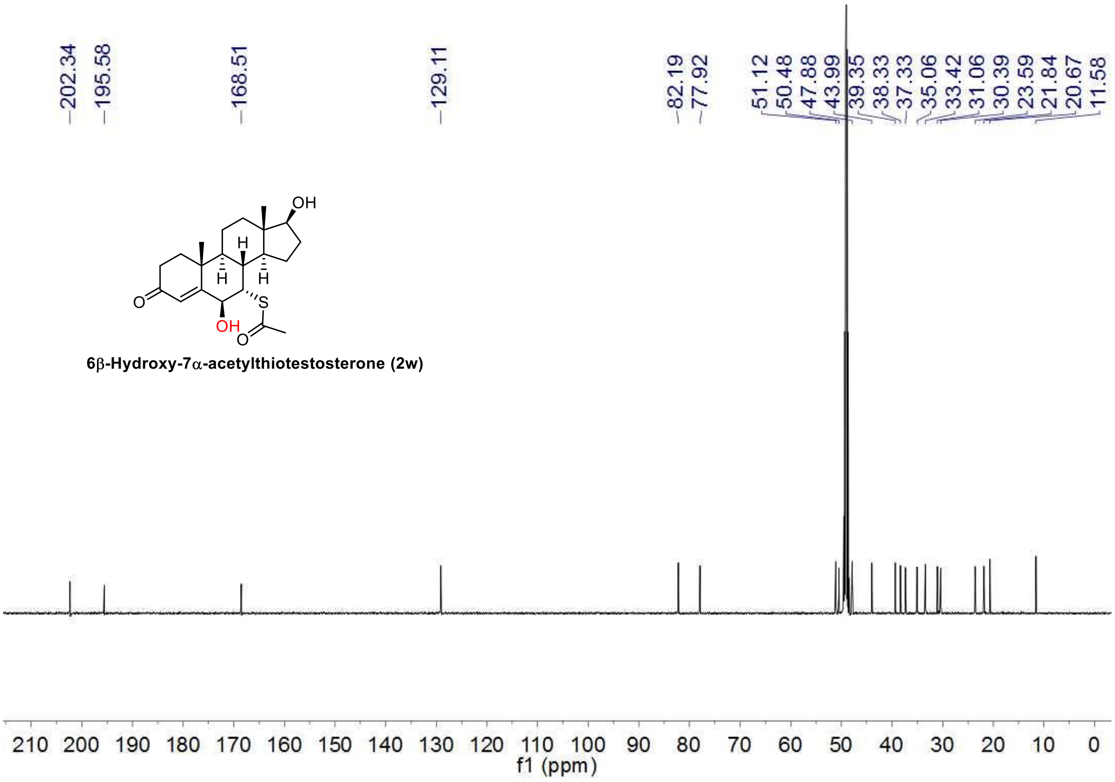

Supplementary Fig. 87. ^{13}C NMR spectrum of compound **2w (126 MHz, CD$_3$OD)**

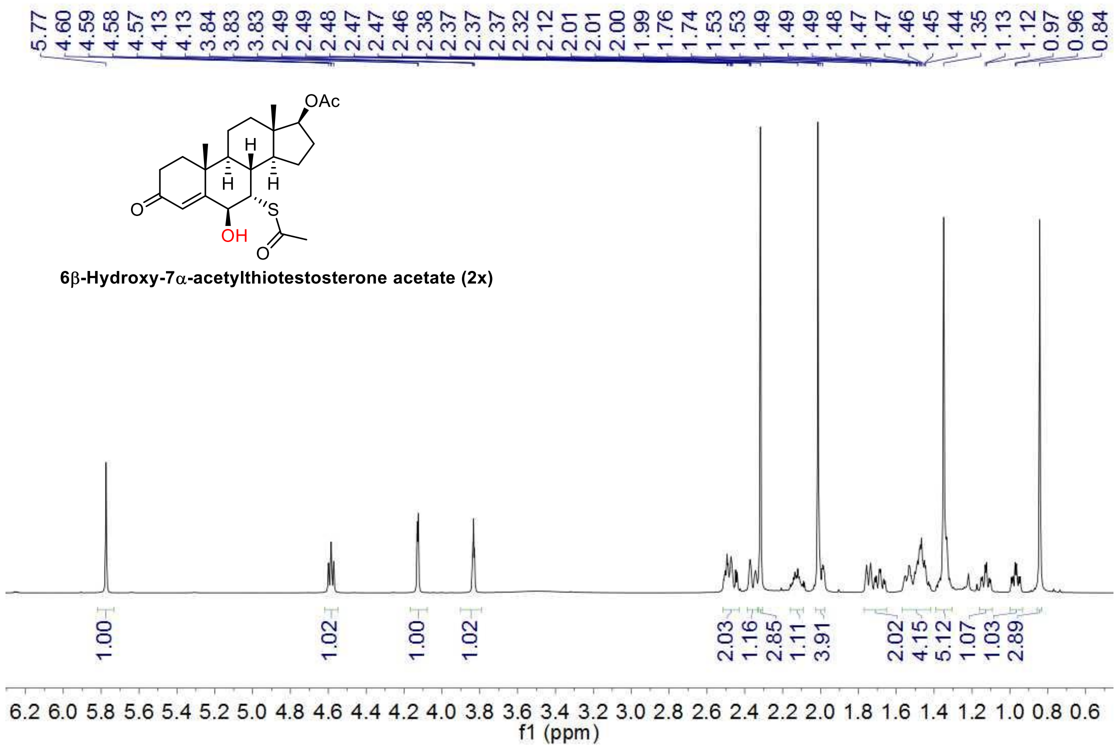

Supplementary Fig. 88. ^{1}H NMR spectrum of compound **2x (600 MHz, $CDCl_3$)**

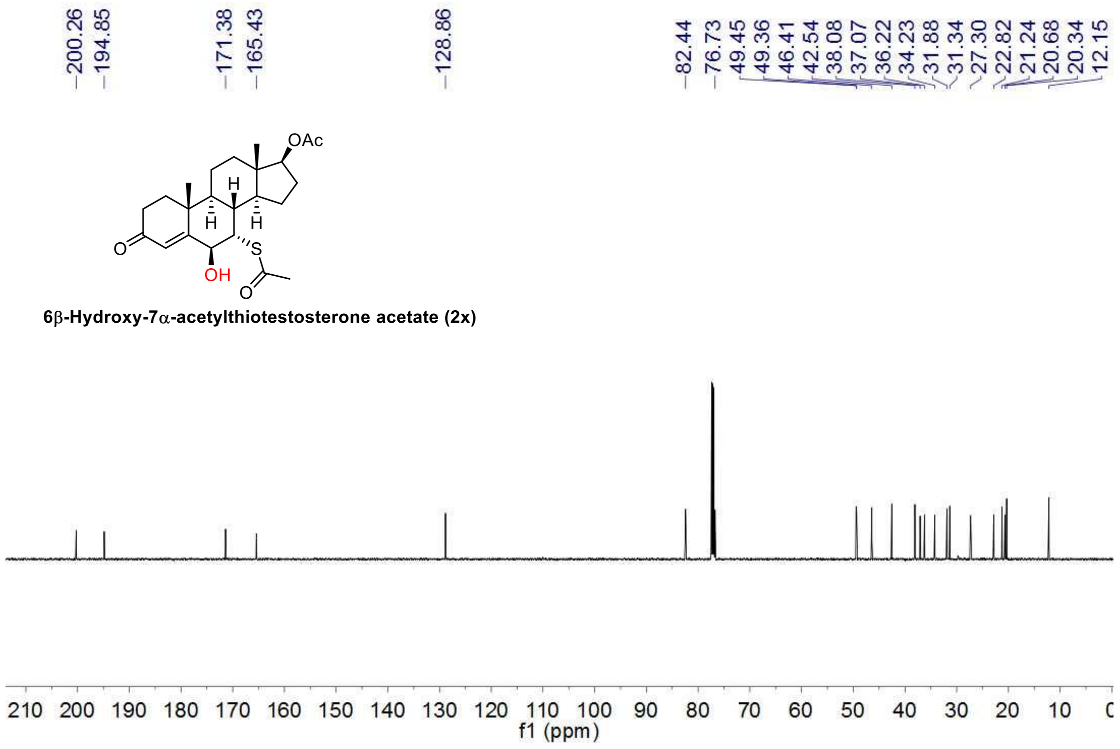

Supplementary Fig. 89. ^{13}C NMR spectrum of compound **2x (151 MHz, $CDCl_3$)**

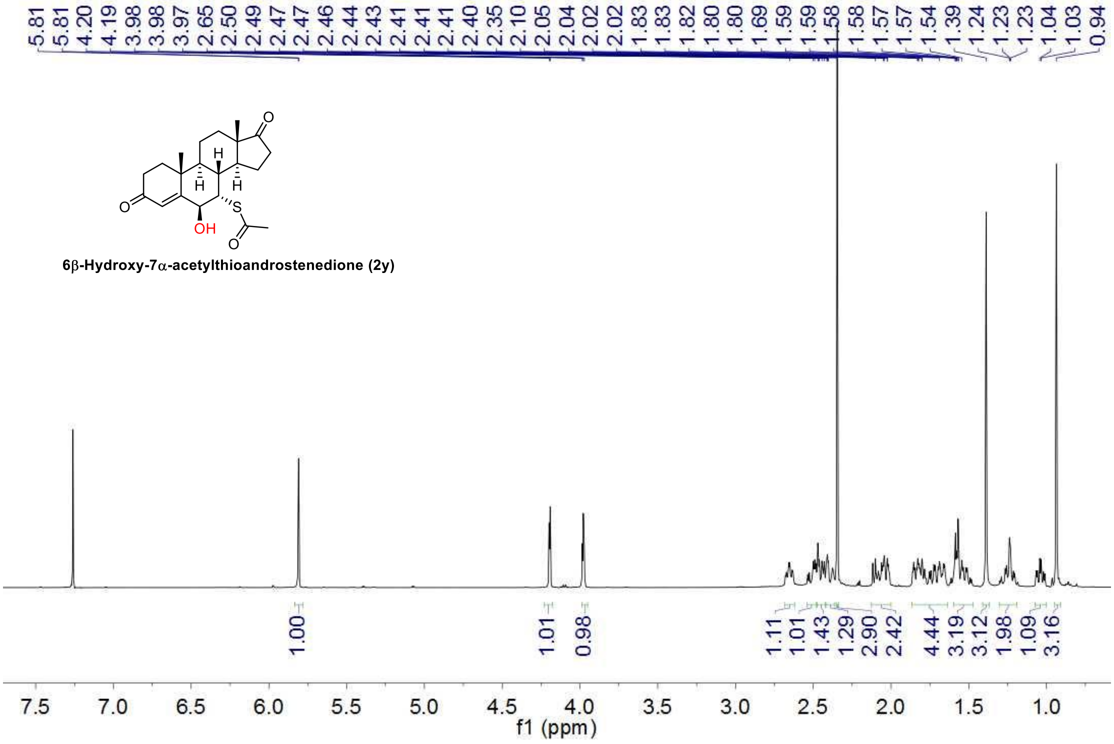

Supplementary Fig. 90. ^{1}H NMR spectrum of compound **2y (500 MHz, CDCl**$_3$**)**

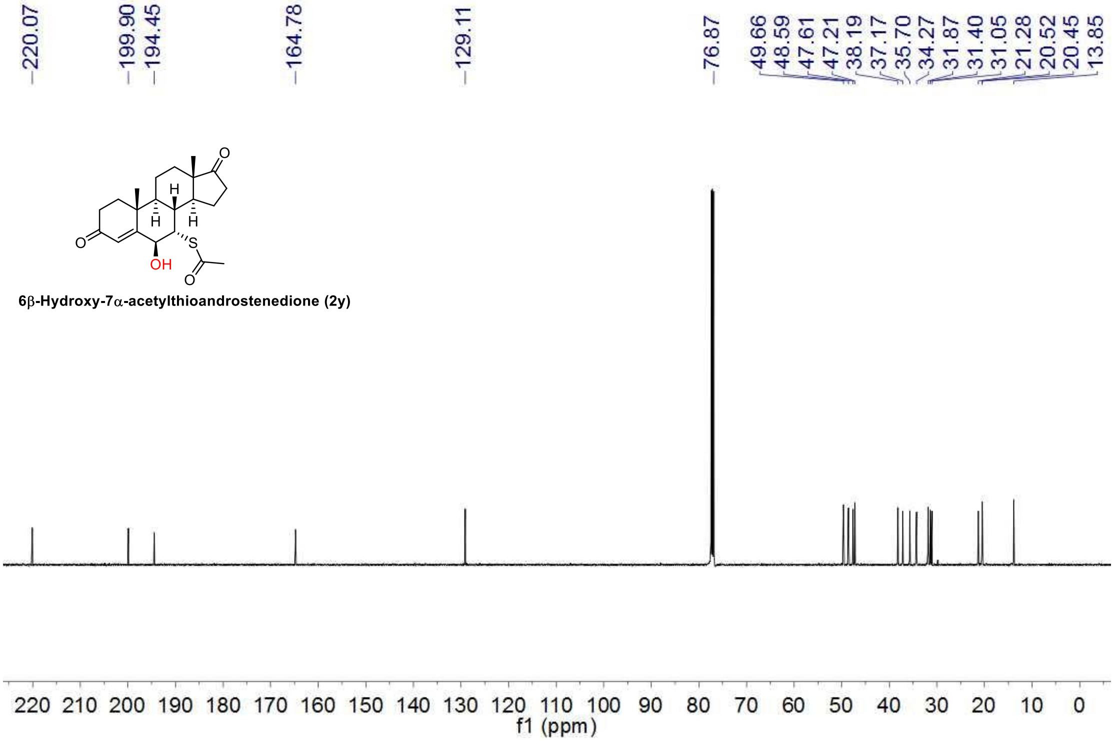

Supplementary Fig. 91. ^{13}C NMR spectrum of compound **2y (126 MHz, CDCl**$_3$**)**

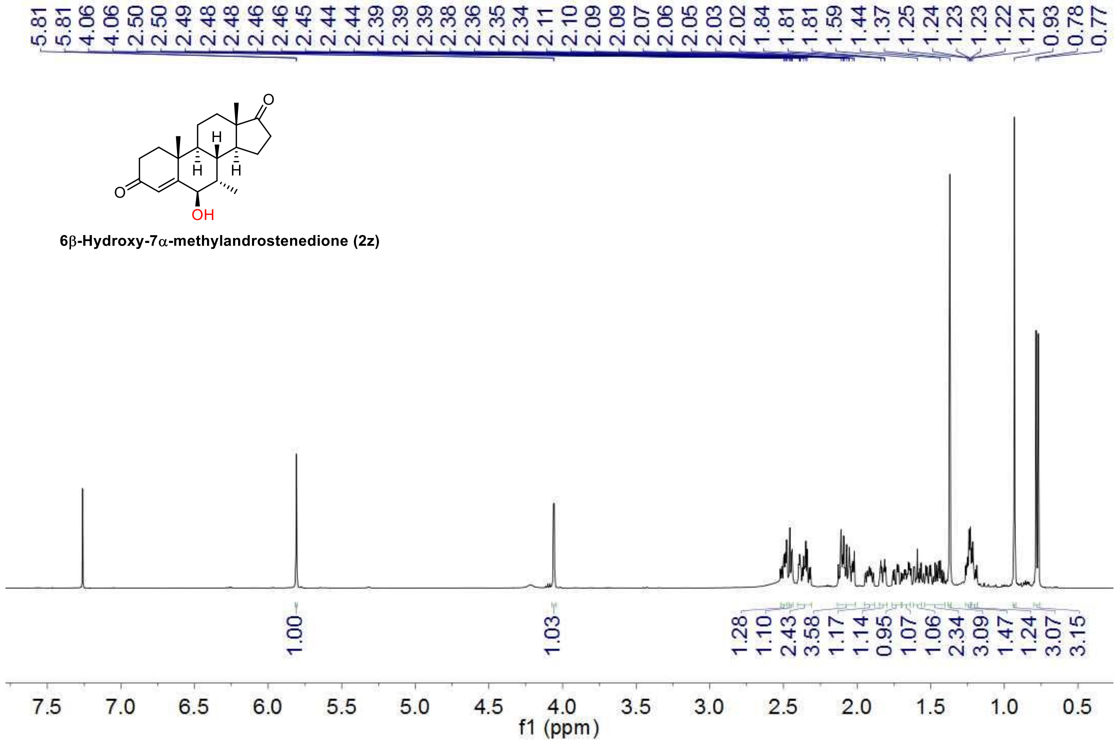

Supplementary Fig. 92. ^{1}H NMR spectrum of compound **2z (500 MHz, CDCl3)**

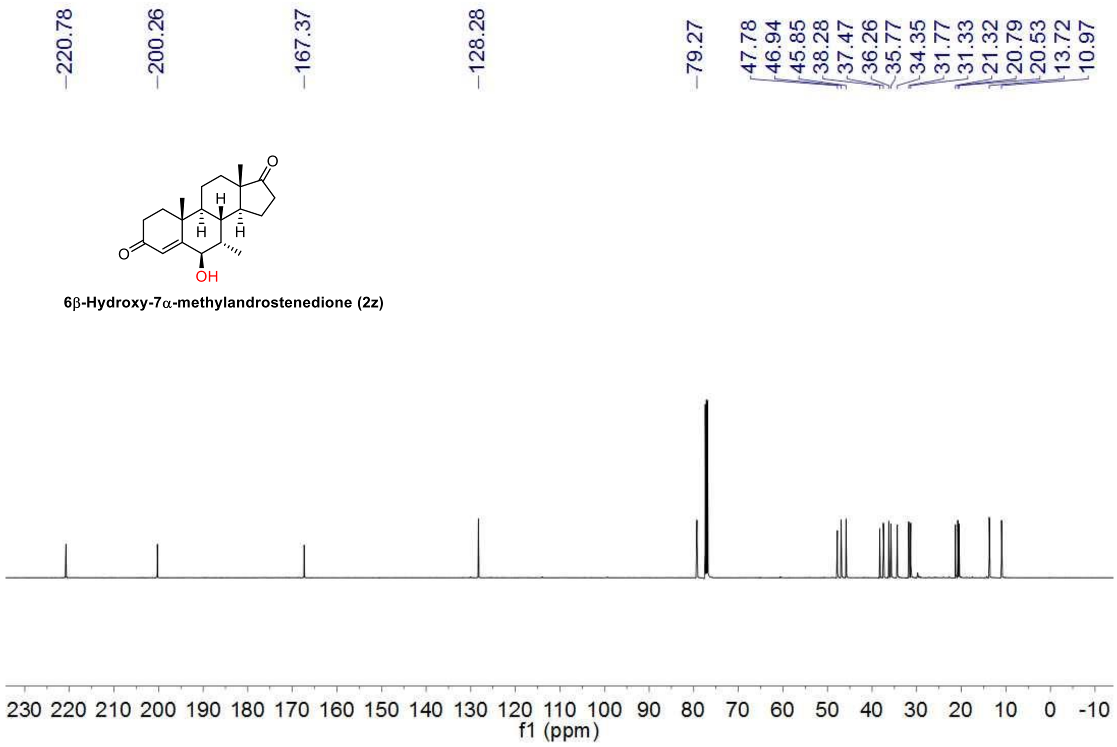

Supplementary Fig. 93. ^{13}C NMR spectrum of compound **2z (126 MHz, CDCl3)**

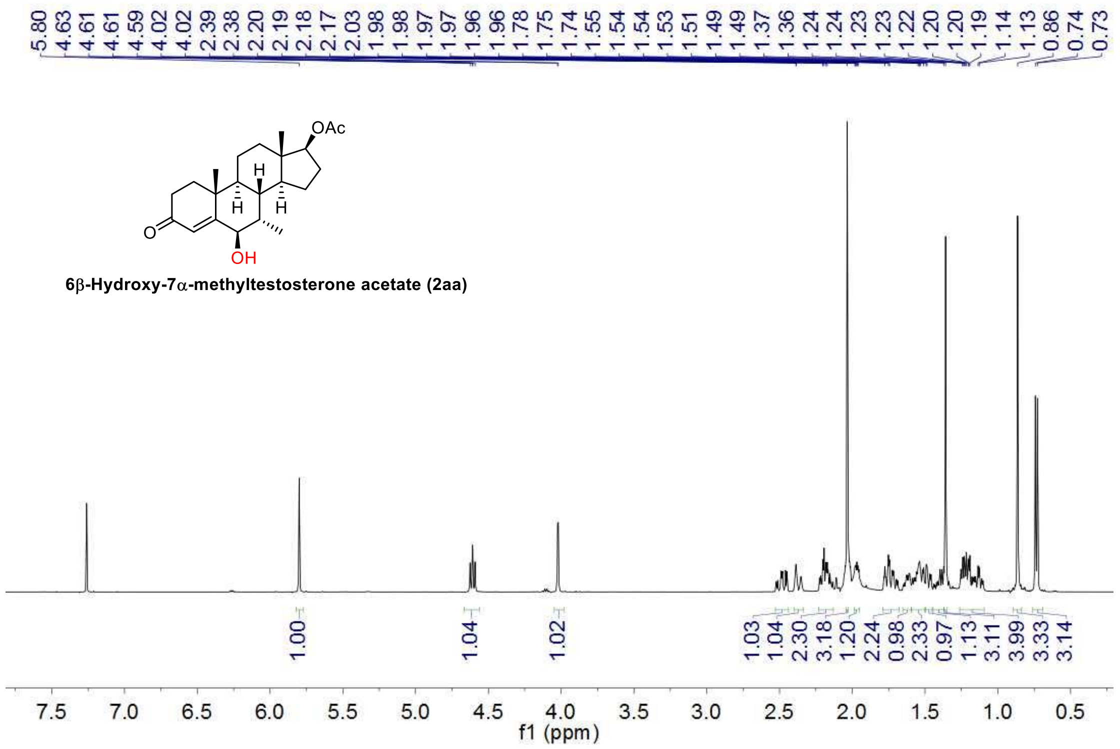

Supplementary Fig. 94. ^{1}H NMR spectrum of compound **2aa (500 MHz, CDCl₃)**

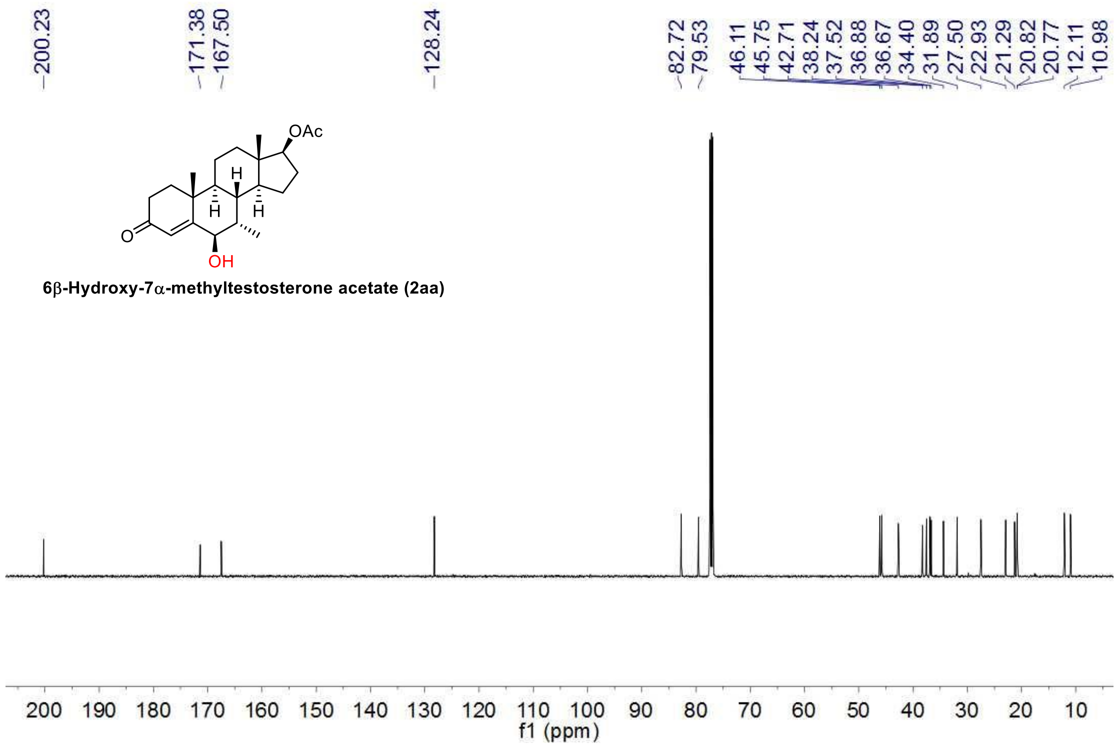

Supplementary Fig. 95. ^{13}C NMR spectrum of compound **2aa (126 MHz, CDCl₃)**

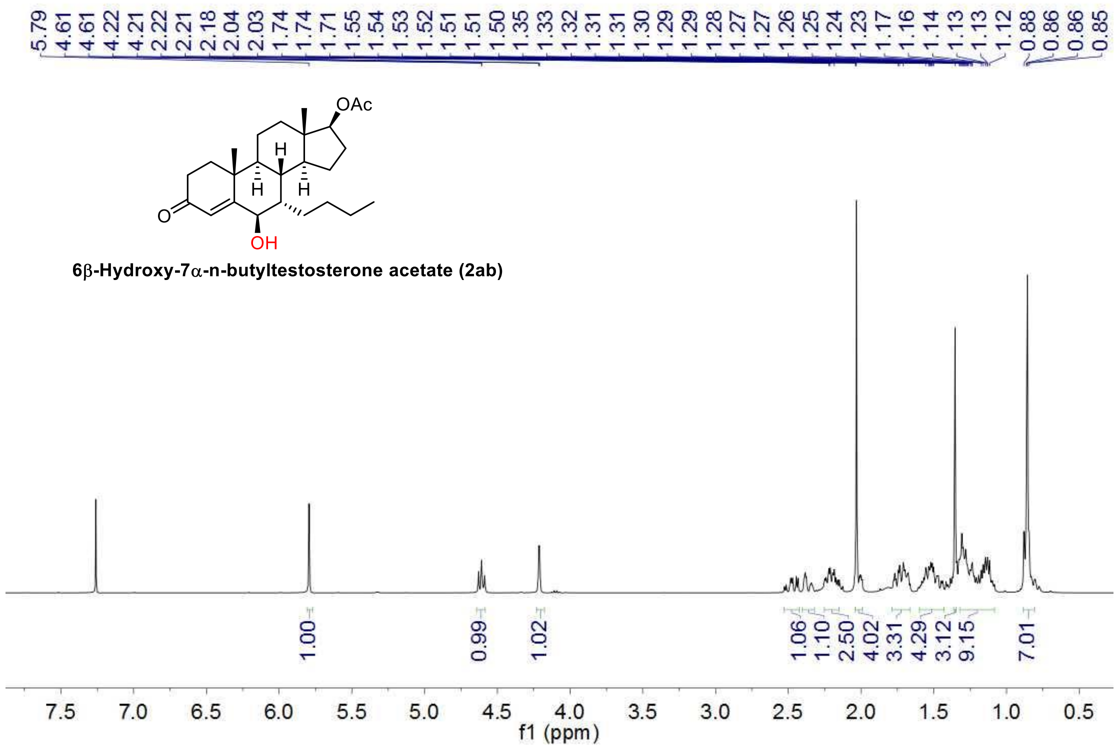

Supplementary Fig. 96. ¹H NMR spectrum of compound **2ab (400 MHz, CDCl₃)**

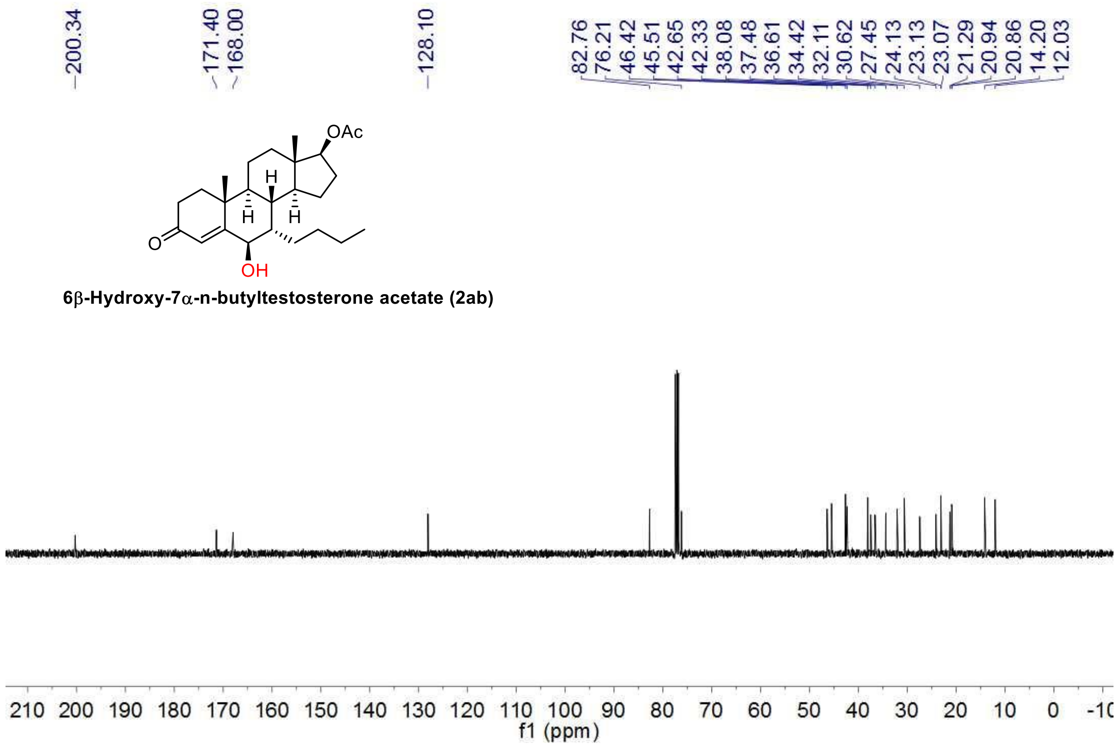

Supplementary Fig. 97. ¹³C NMR spectrum of compound **2ab (101 MHz, CDCl₃)**

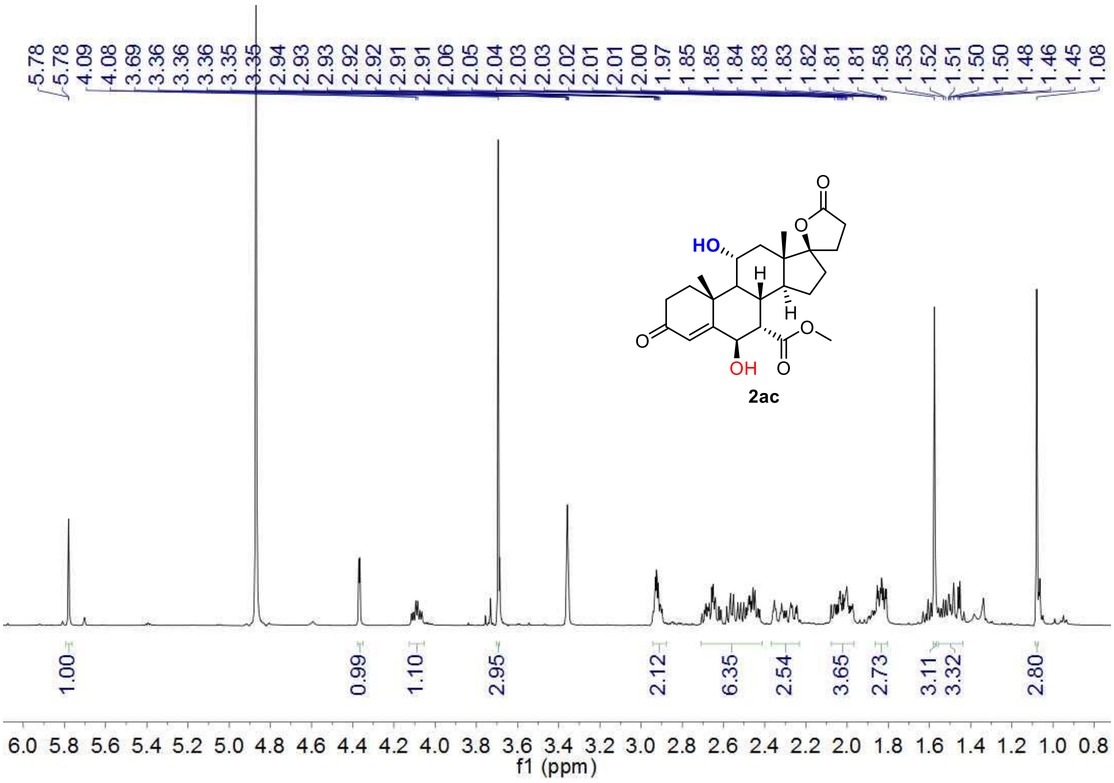

Supplementary Fig. 98. ^{1}H NMR spectrum of compound **2ac (500 MHz, CD_3OD)**

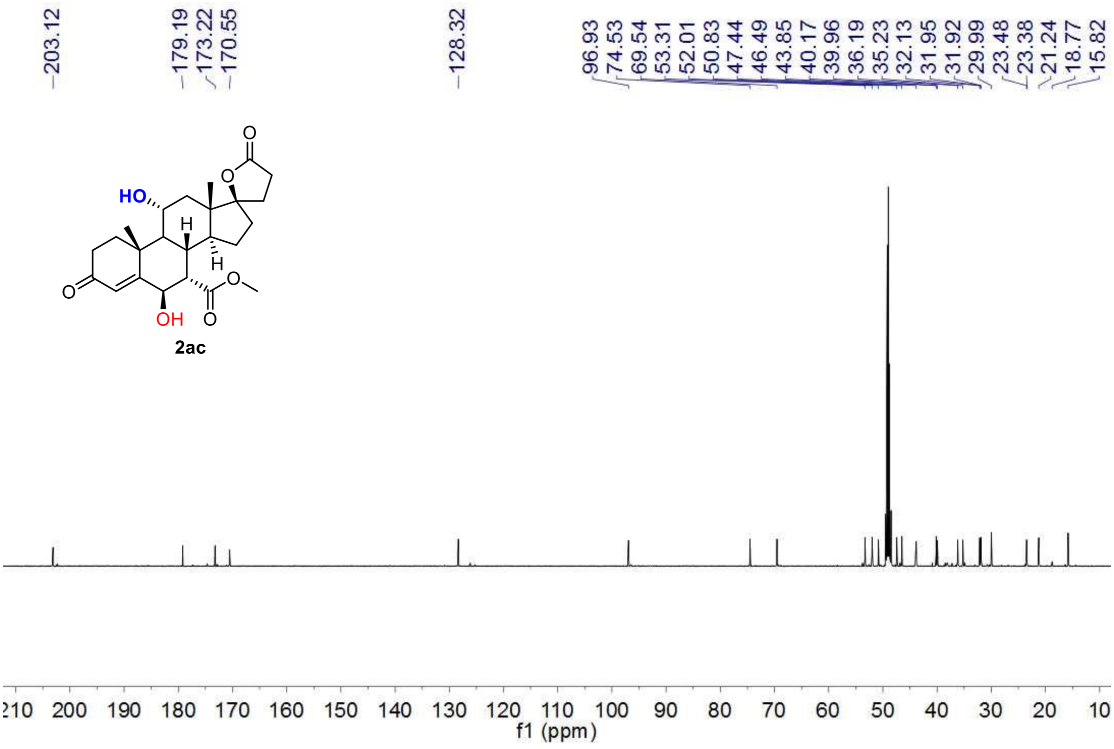

Supplementary Fig. 99. ^{13}C NMR spectrum of compound **2ac (126 MHz, CD_3OD)**

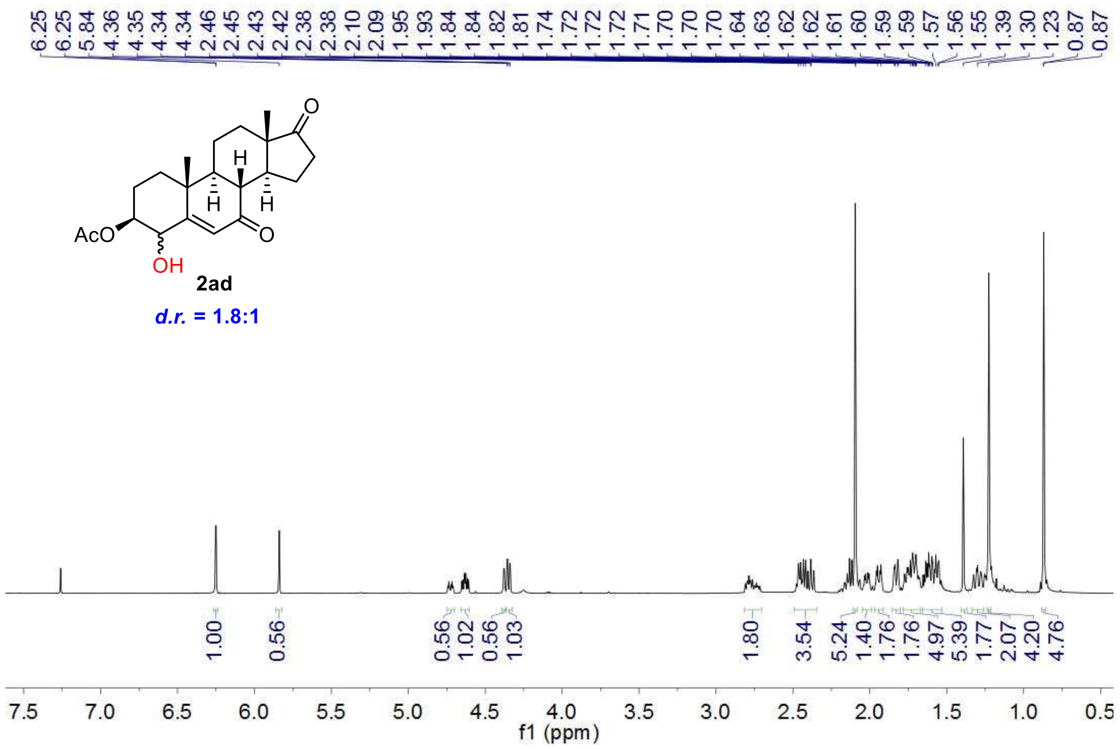

Supplementary Fig. 100. ^{1}H NMR spectrum of compound **2ad (600 MHz, CDCl$_3$)**

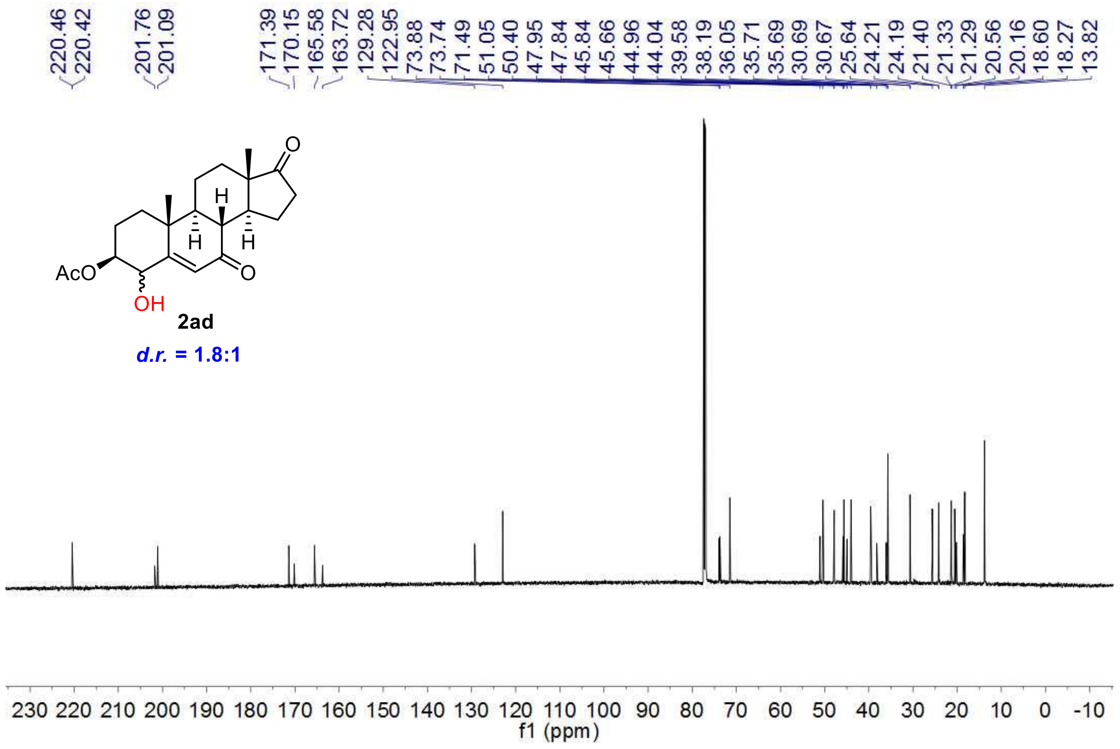

Supplementary Fig. 101. ^{13}C NMR spectrum of compound **2ad (151 MHz, CDCl$_3$)**

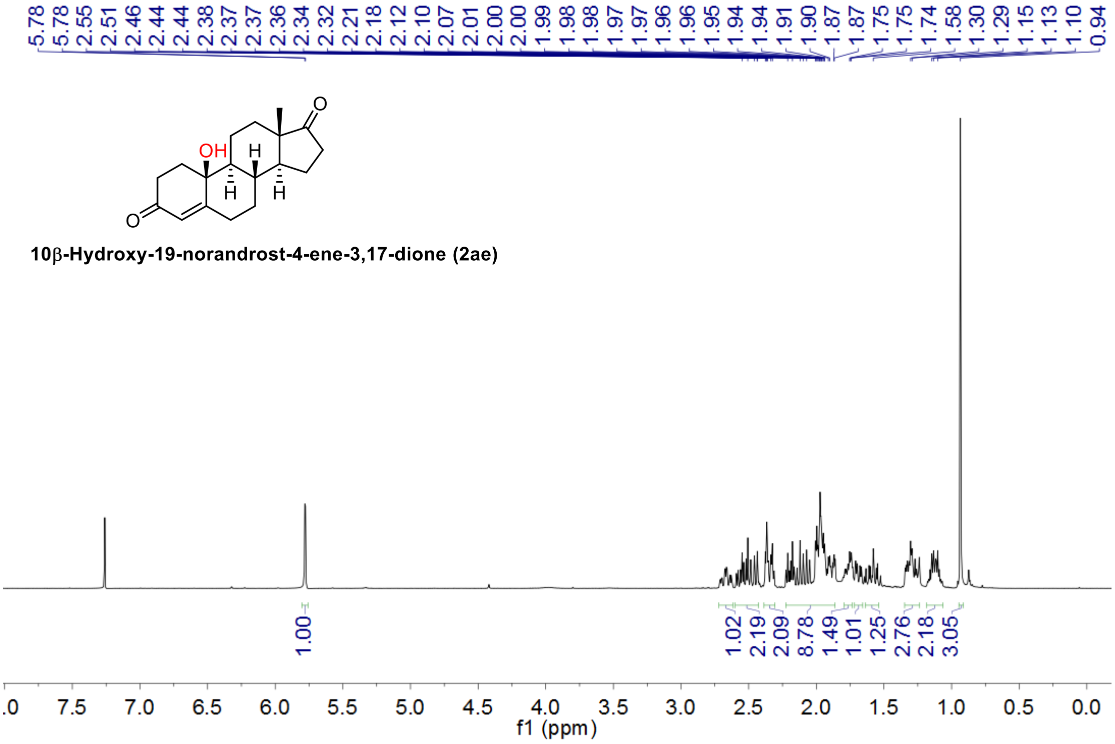

Supplementary Fig. 102. ^{1}H NMR spectrum of compound **2ae (400 MHz, CDCl$_3$)**

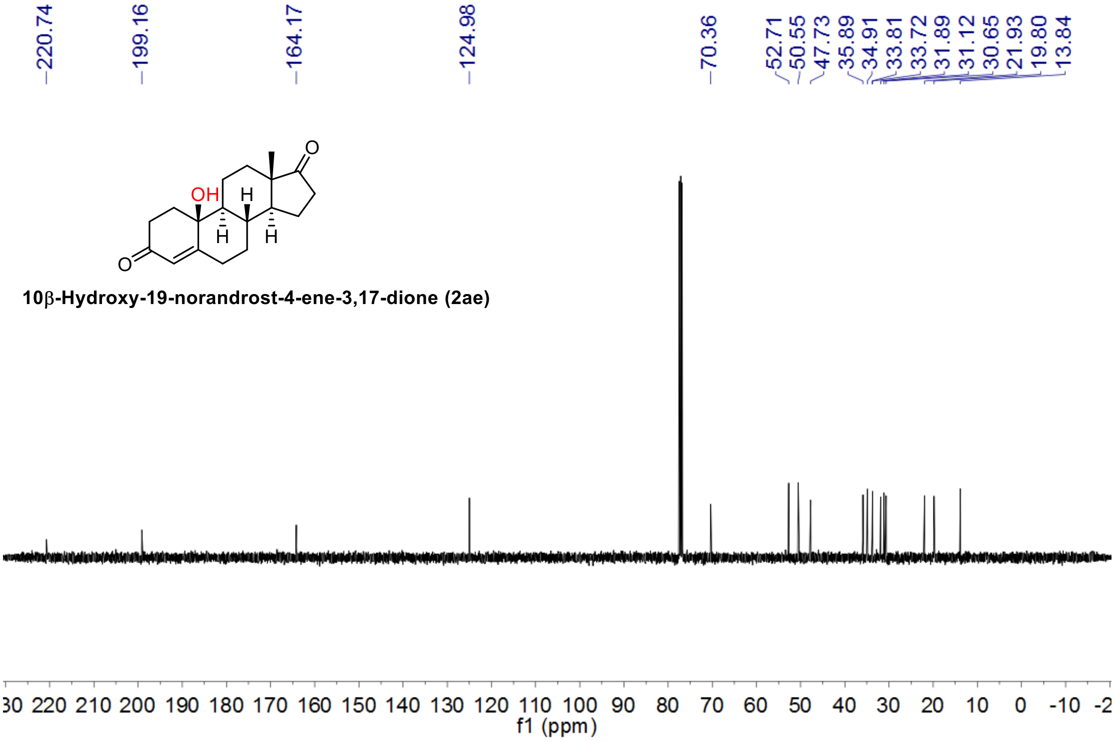

Supplementary Fig. 103. ^{13}C NMR spectrum of compound **2ae (101 MHz, CDCl$_3$)**

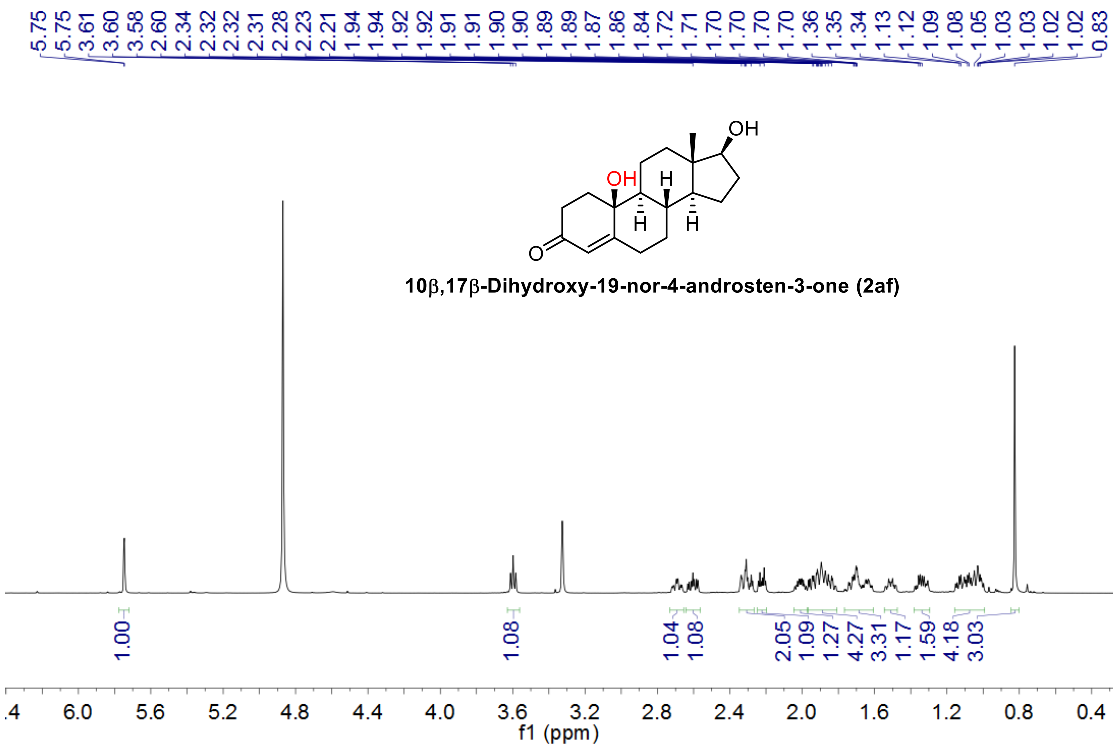

Supplementary Fig. 104. ^{1}H NMR spectrum of compound **2af (600 MHz, CD_3OD)**

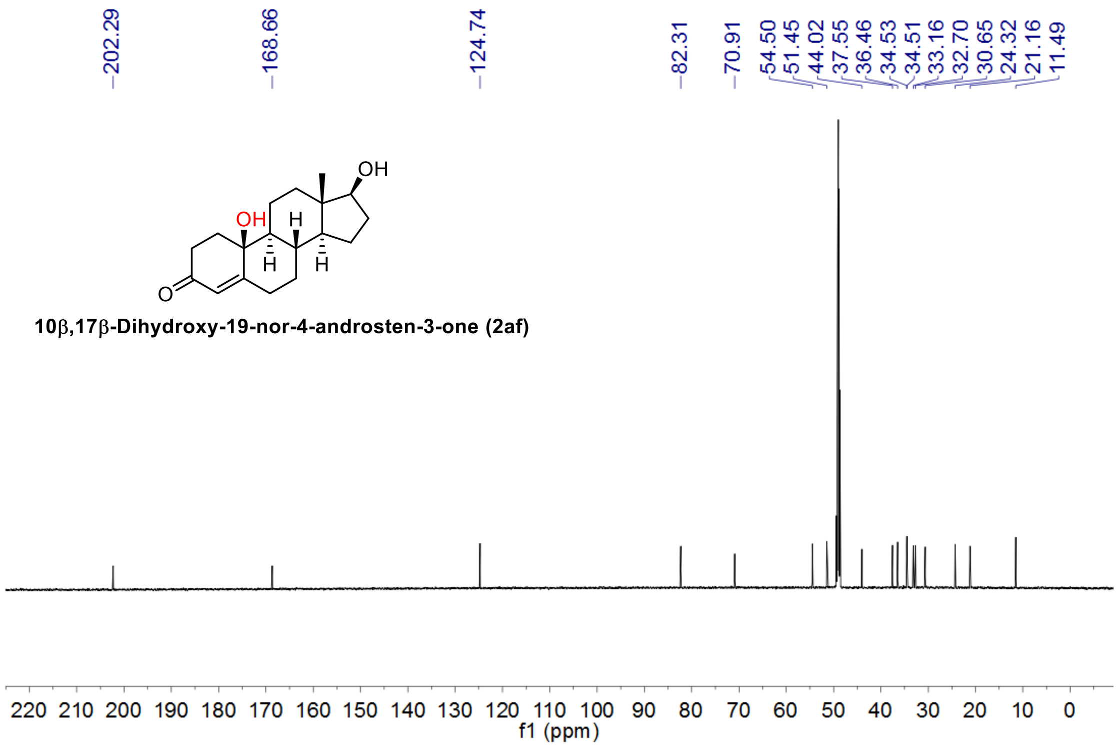

Supplementary Fig. 105. ^{13}C NMR spectrum of compound **2af (151 MHz, CD_3OD)**

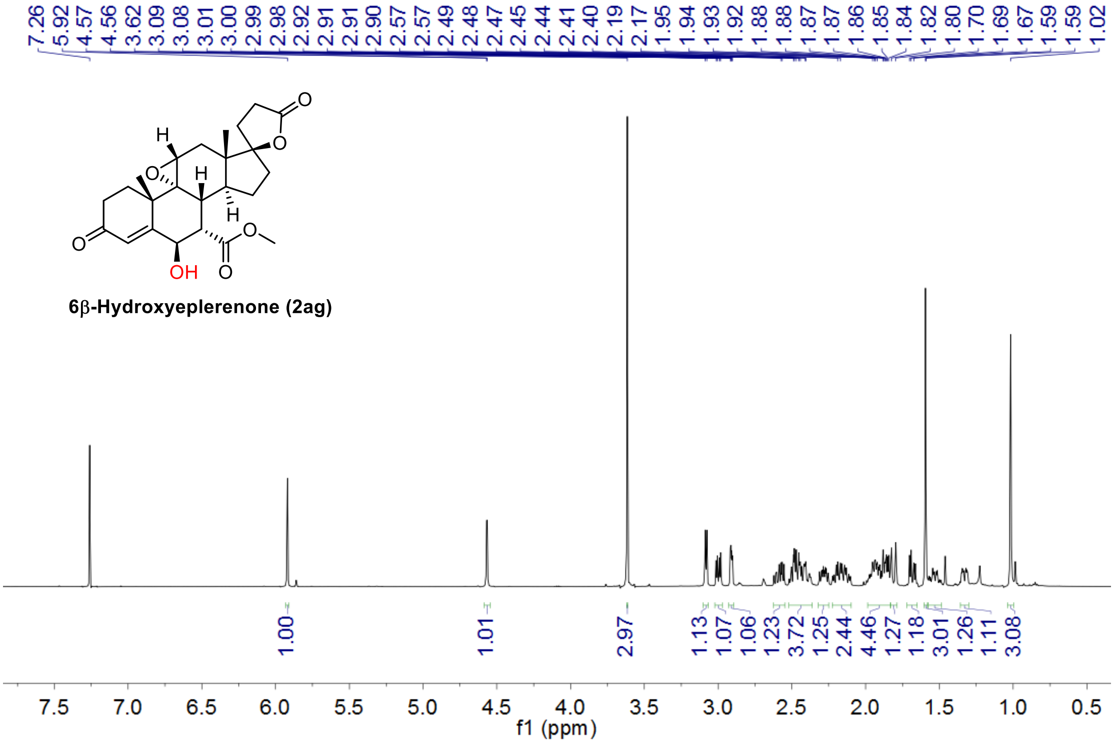

Supplementary Fig. 106. ^{1}H NMR spectrum of compound **2ag (500 MHz, CDCl3)**

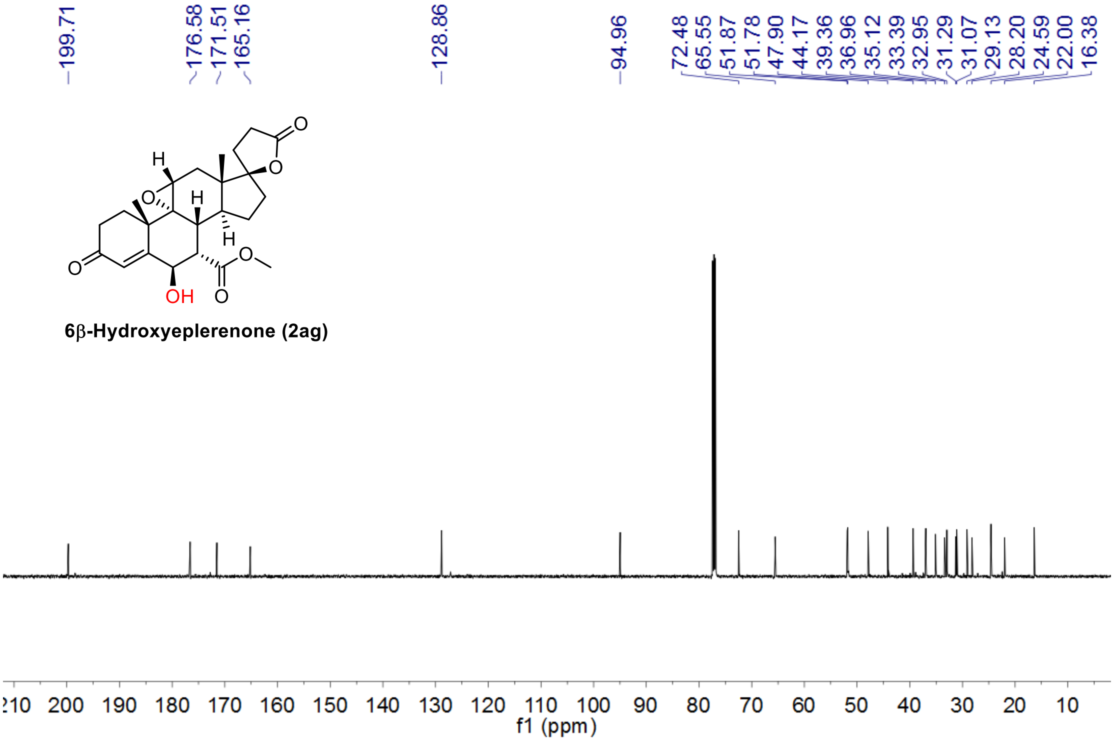

Supplementary Fig. 107. ^{13}C NMR spectrum of compound **2ag (126 MHz, CDCl3)**

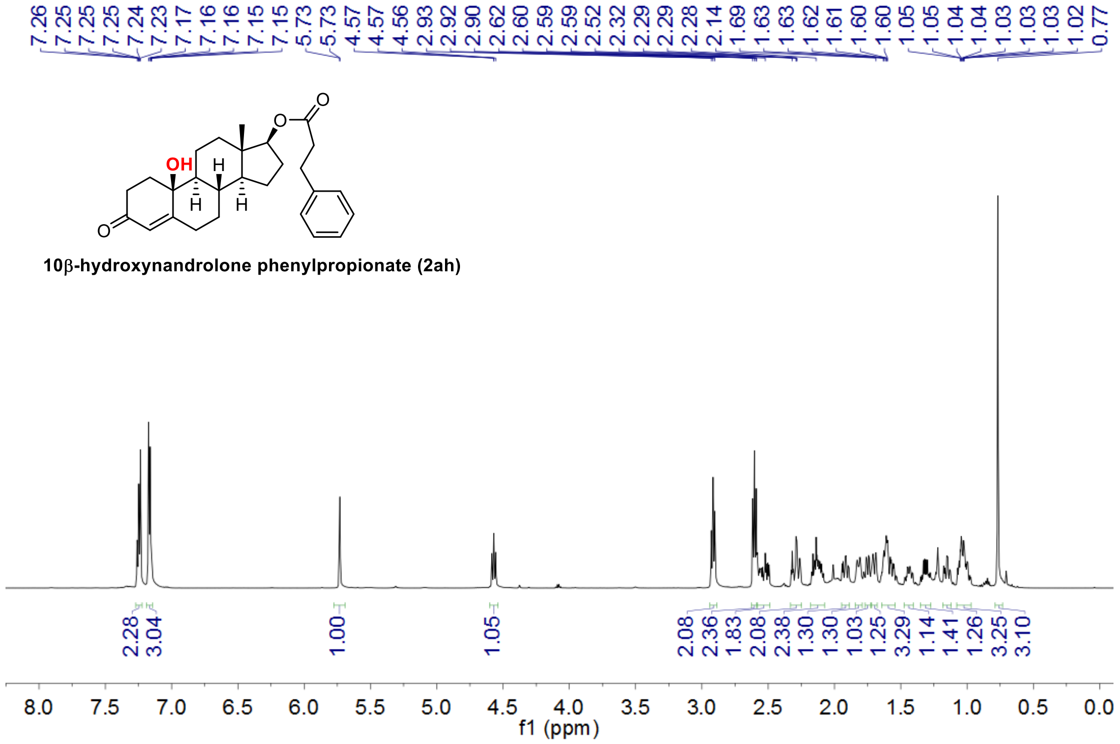

Supplementary Fig. 108. ^{1}H NMR spectrum of compound **2ah (600 MHz, CDCl3)**

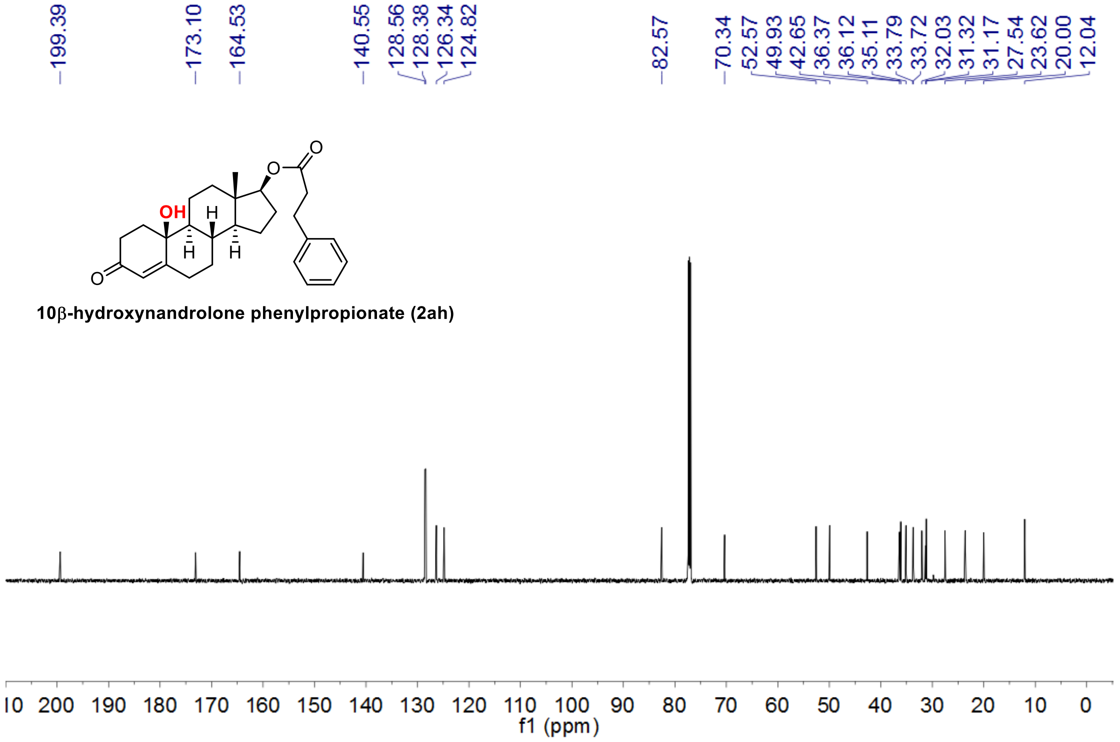

Supplementary Fig. 109. ^{13}C NMR spectrum of compound **2ah (151 MHz, CDCl3)**

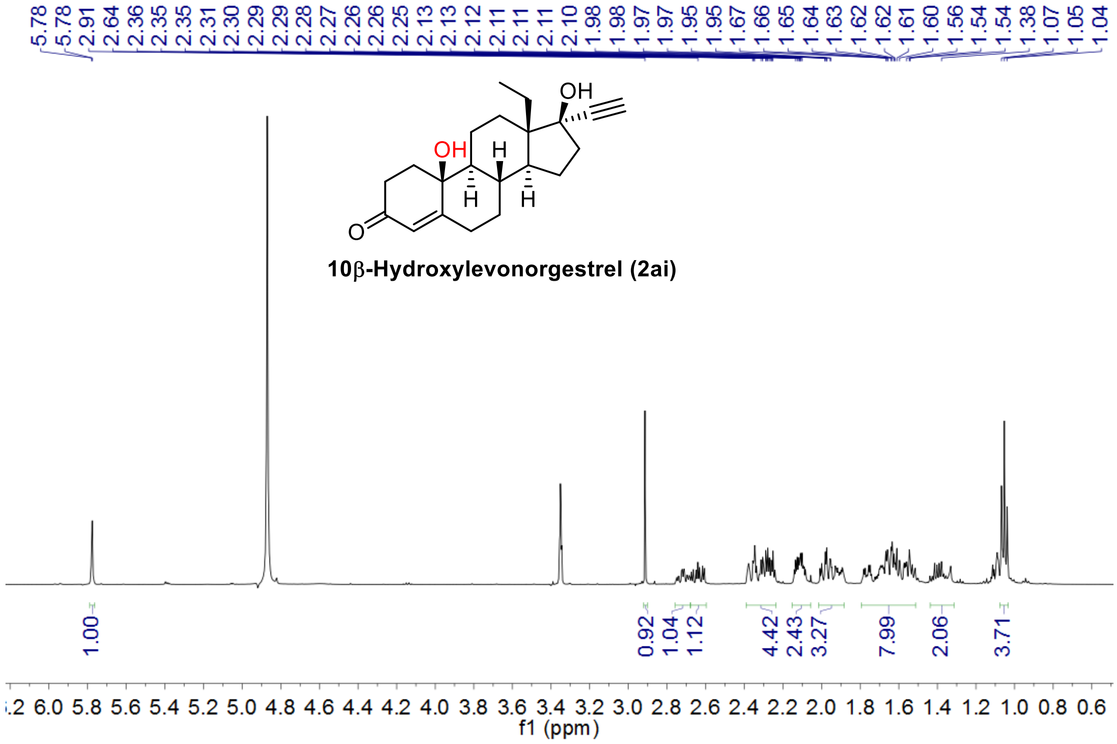

Supplementary Fig. 110. ^{1}H NMR spectrum of compound **2ai (500 MHz, CD$_3$OD)**

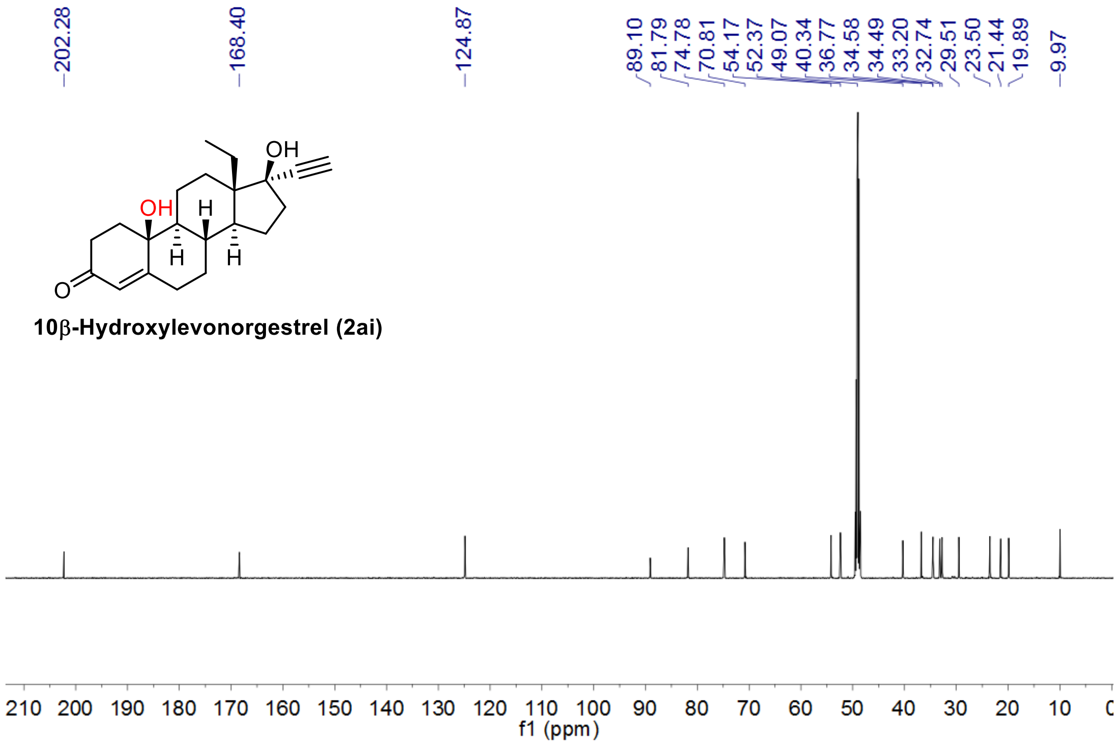

Supplementary Fig. 111. ^{13}C NMR spectrum of compound **2ai (126 MHz, CD$_3$OD)**

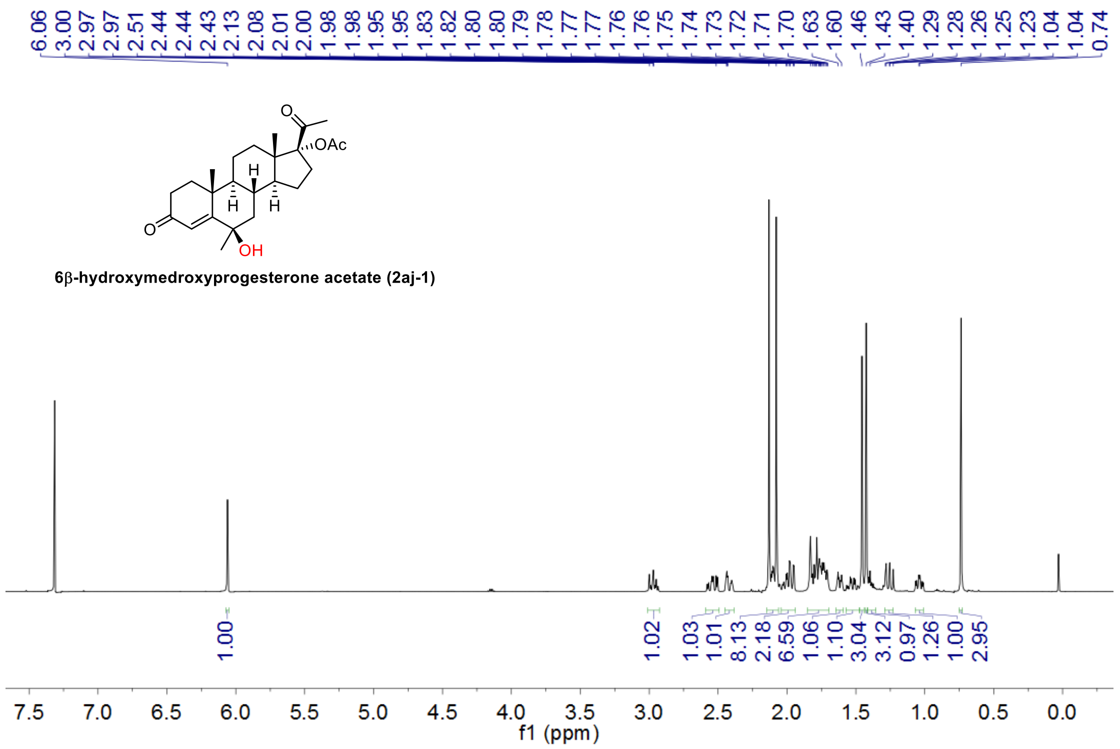

Supplementary Fig. 112. ^{1}H NMR spectrum of compound **2aj-1 (500 MHz, $CDCl_3$)**

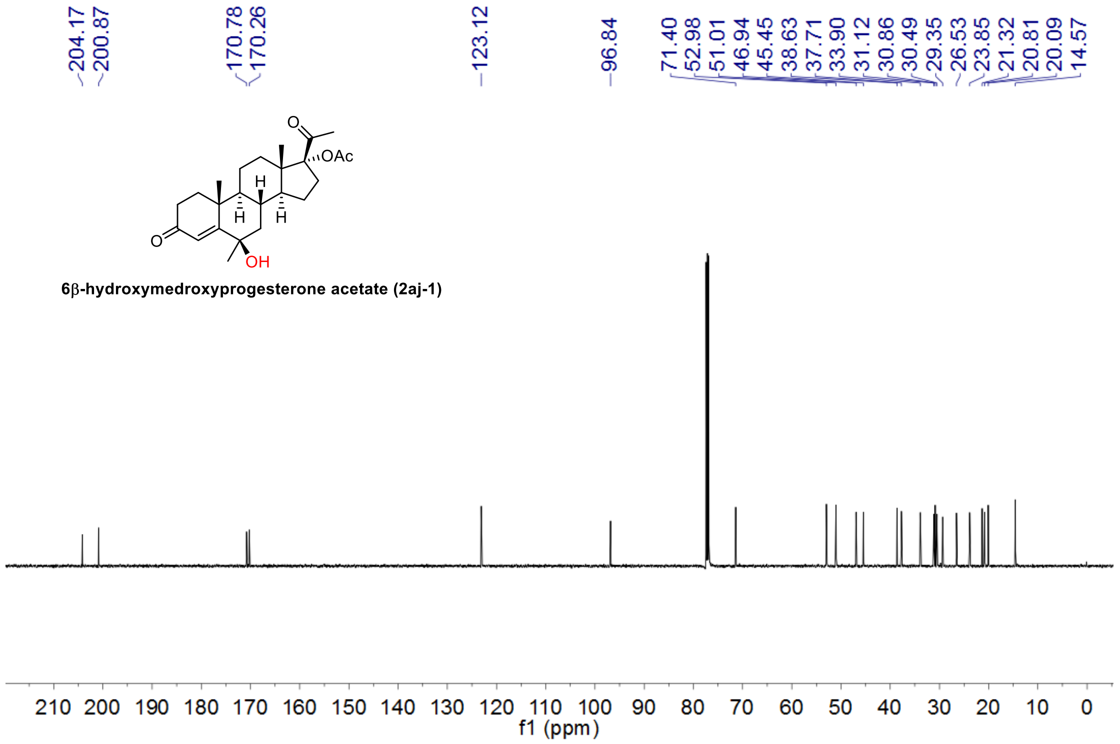

Supplementary Fig. 113. ^{13}C NMR spectrum of compound **2aj-1 (126 MHz, $CDCl_3$)**

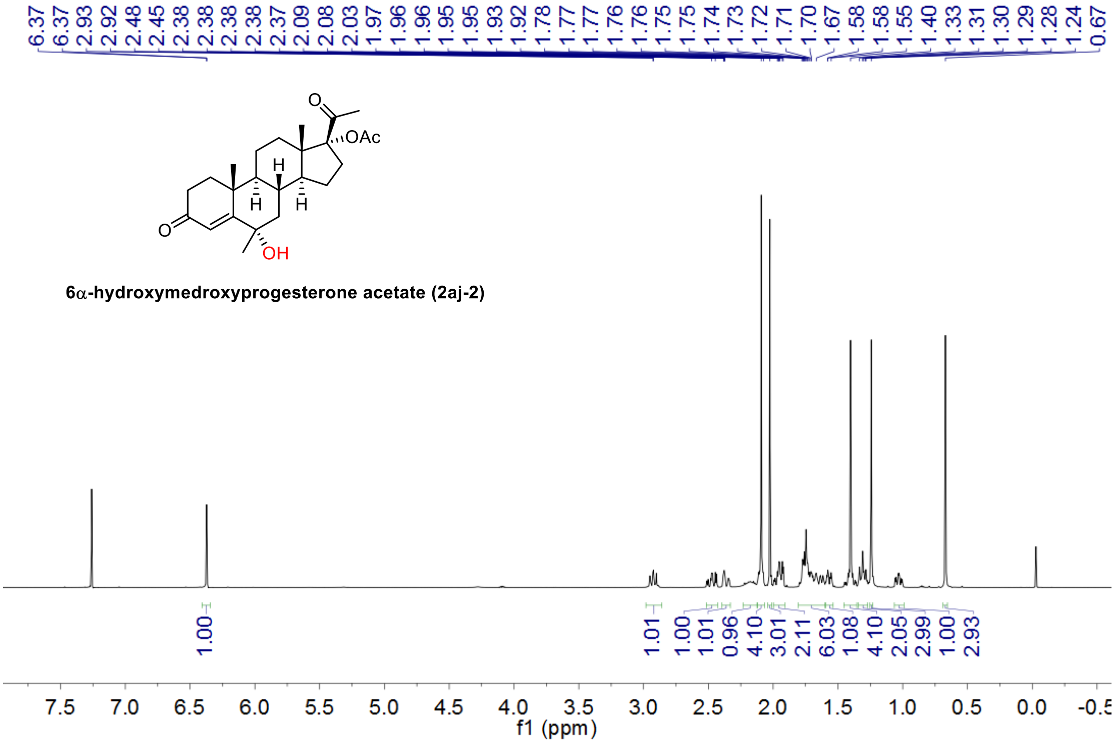

Supplementary Fig. 114. ^{1}H NMR spectrum of compound **2aj-2 (500 MHz, CDCl₃)**

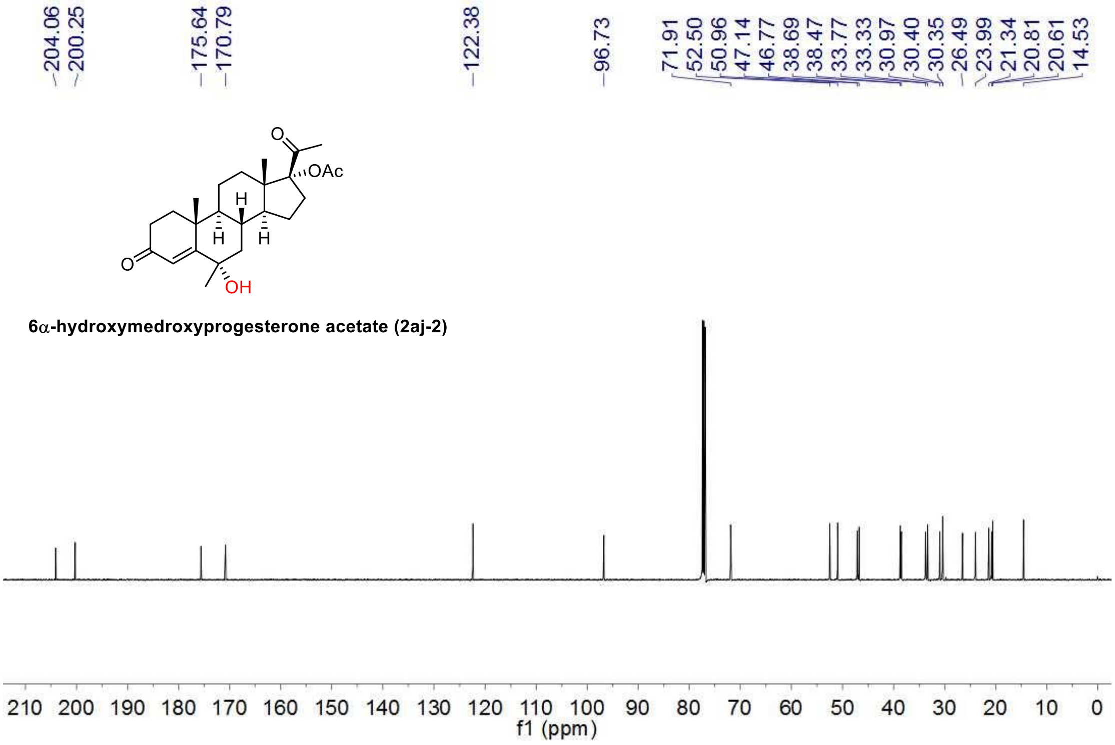

Supplementary Fig. 115. ^{13}C NMR spectrum of compound **2aj-2 (126 MHz, $CDCl_3$)**

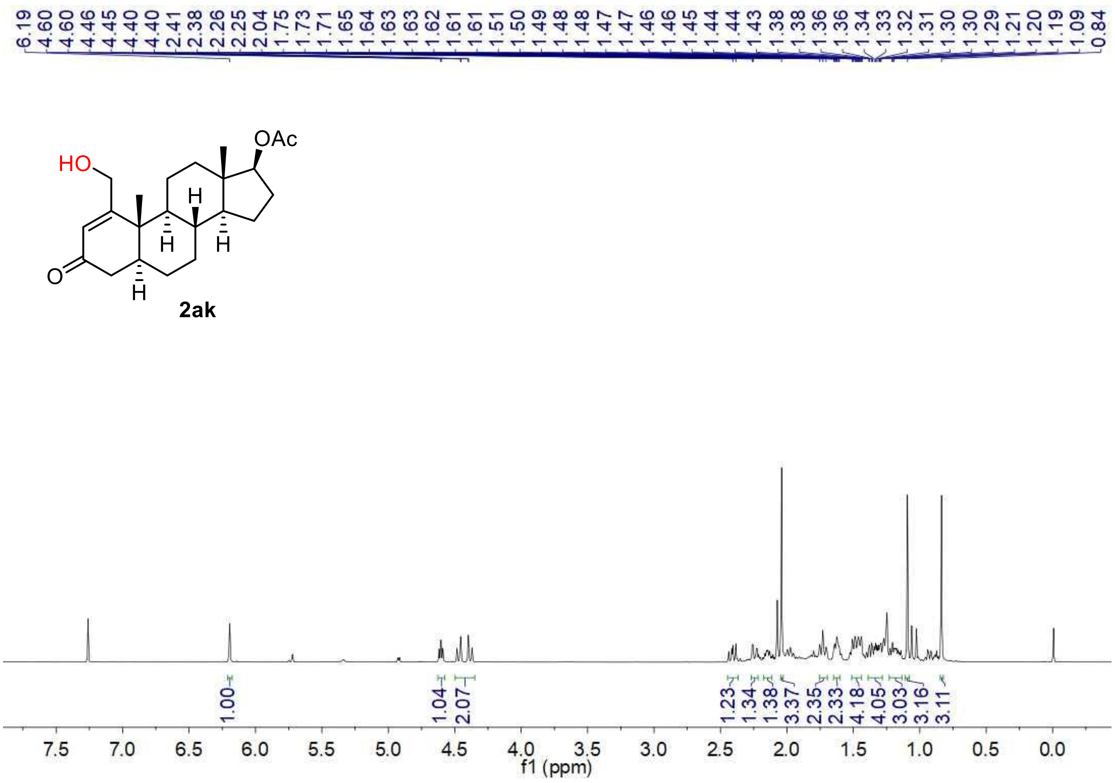

Supplementary Fig. 116. ^{1}H NMR spectrum of compound **2ak (600 MHz, CDCl$_3$)**

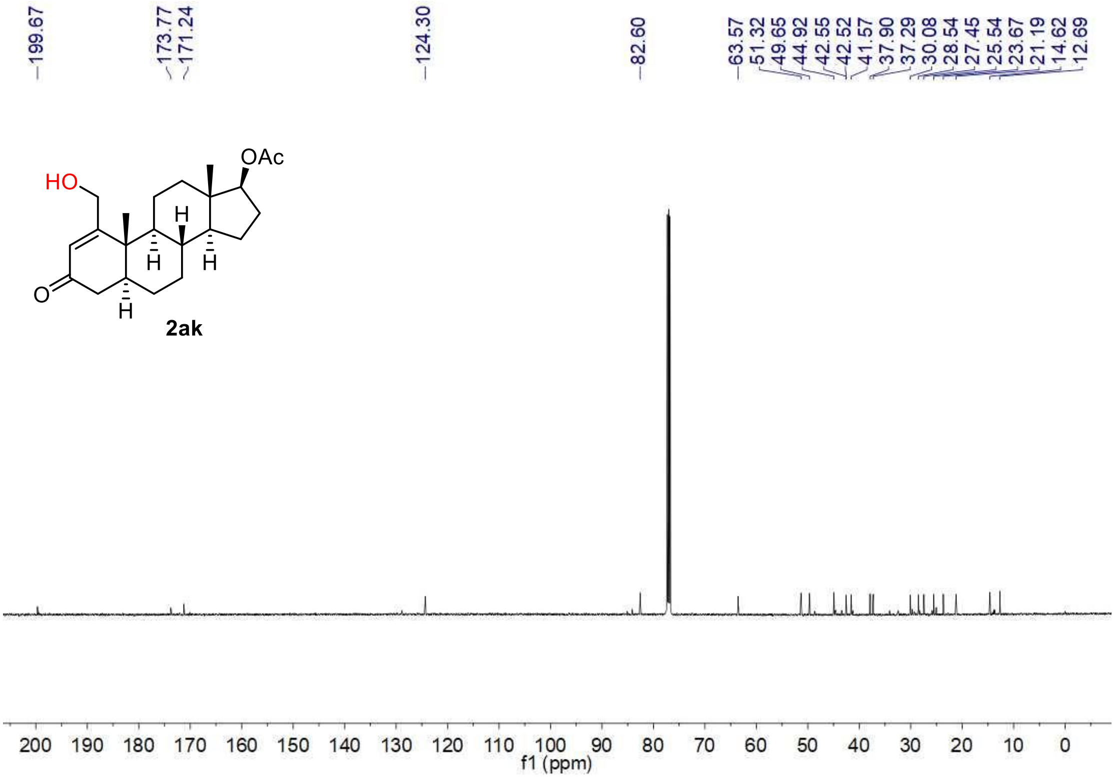

Supplementary Fig. 117. ^{13}C NMR spectrum of compound **2ak (126 MHz, CDCl$_3$)**

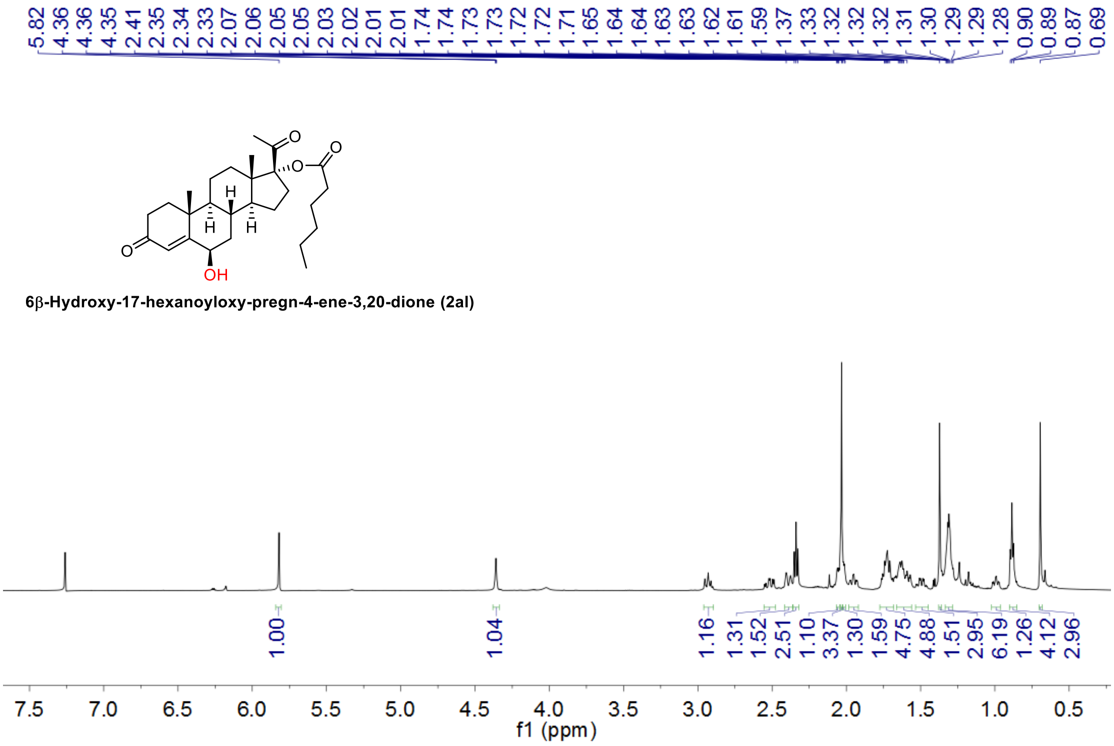

Supplementary Fig. 118. ^{1}H NMR spectrum of compound **2al (600 MHz, CDCl3)**

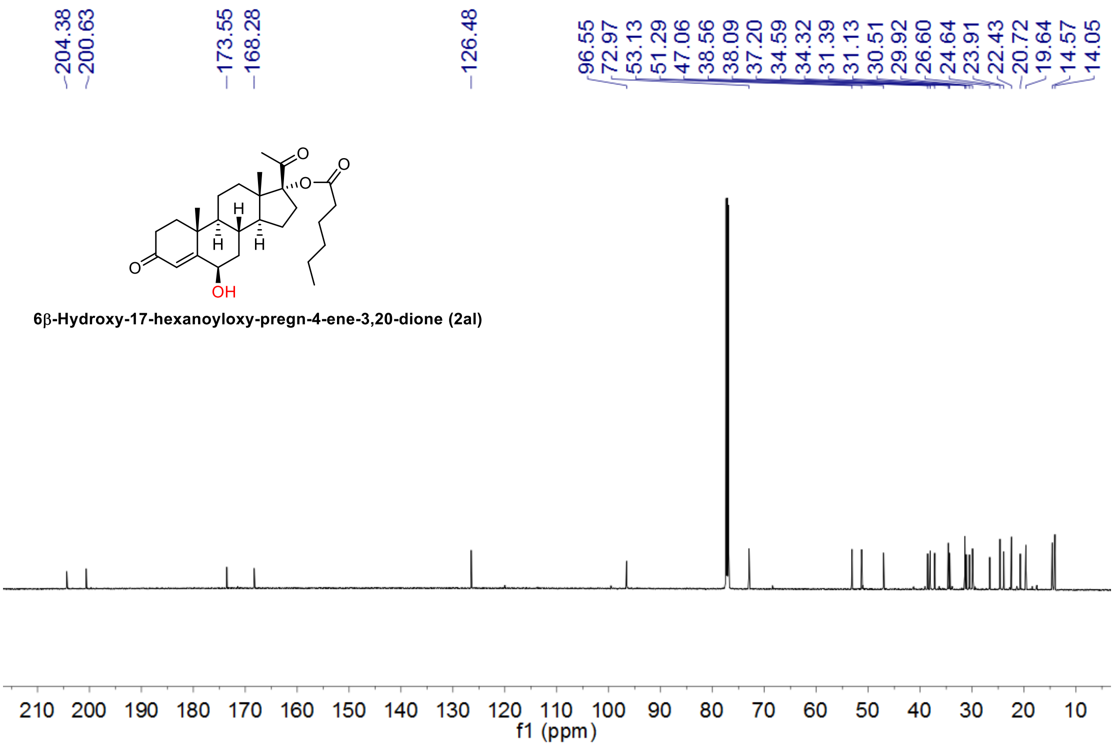

Supplementary Fig. 119. ^{13}C NMR spectrum of compound **2al (151 MHz, CDCl3)**

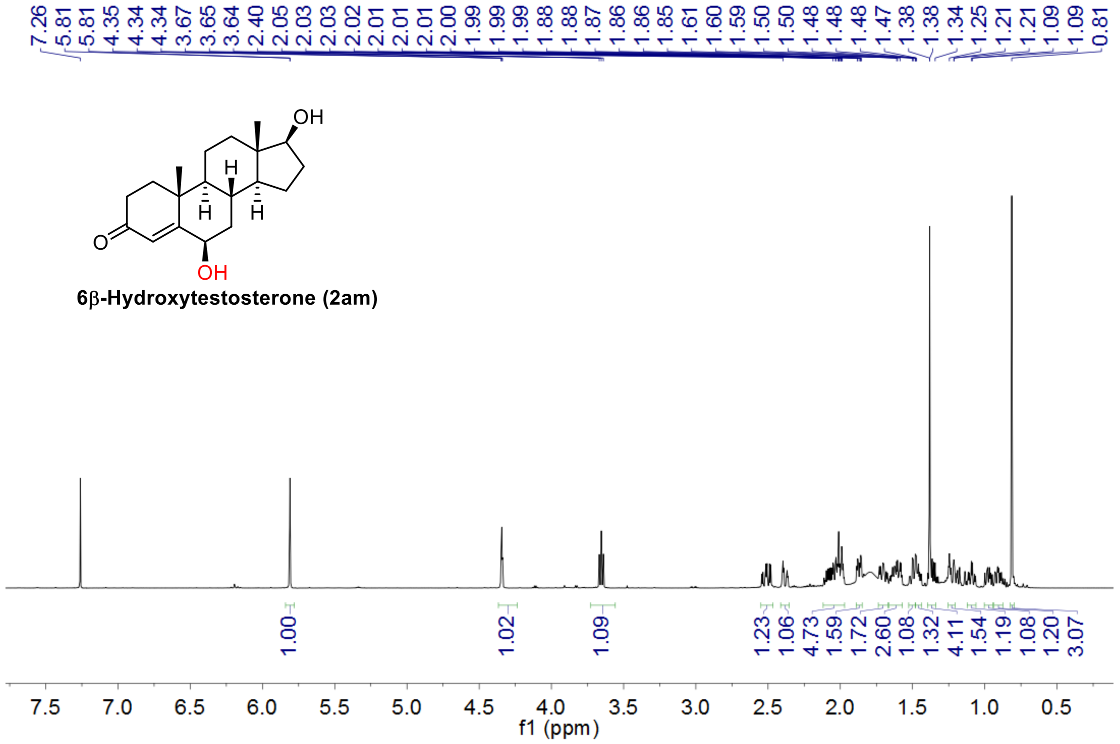

Supplementary Fig. 120. ^{1}H NMR spectrum of compound **2am (600 MHz, CDCl$_3$)**

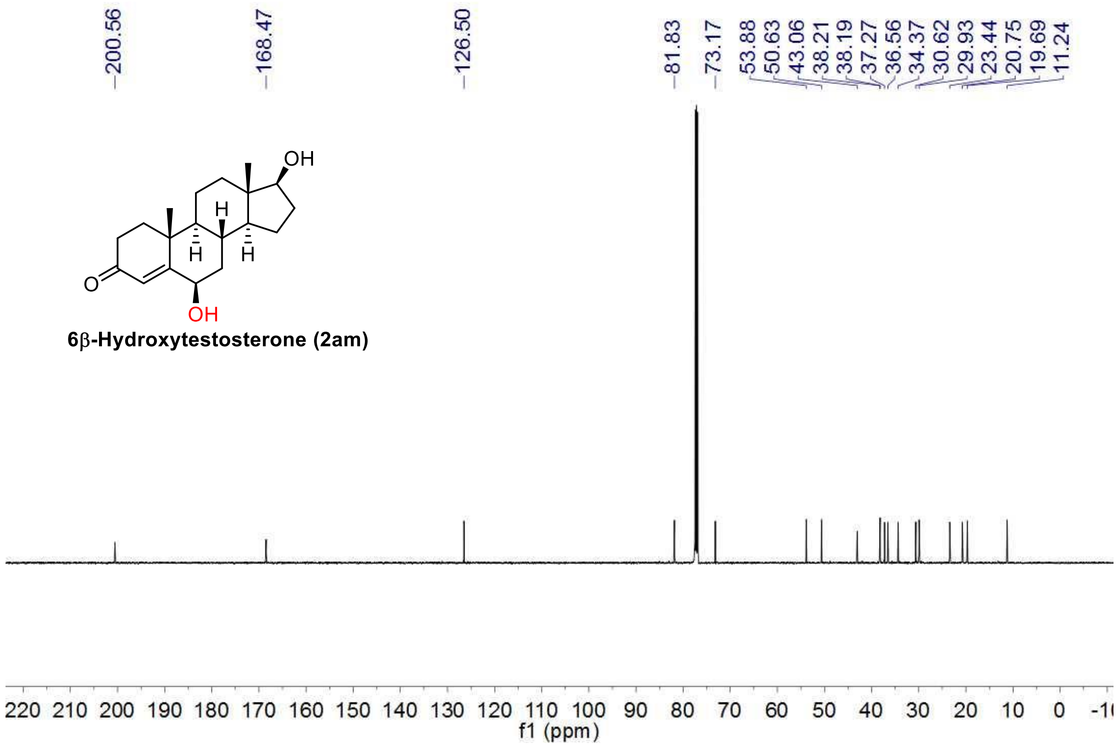

Supplementary Fig. 121. ^{13}C NMR spectrum of compound **2am (126 MHz, CDCl$_3$)**

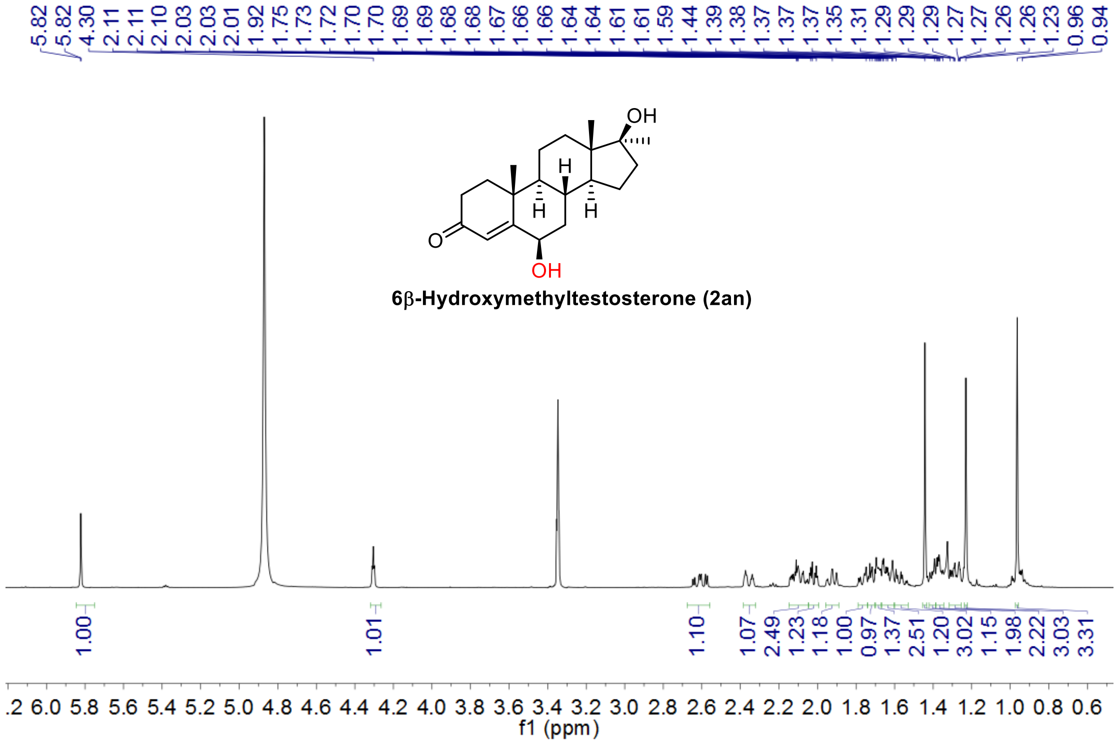

Supplementary Fig. 122. ^{1}H NMR spectrum of compound **2an (500 MHz, CD$_3$OD)**

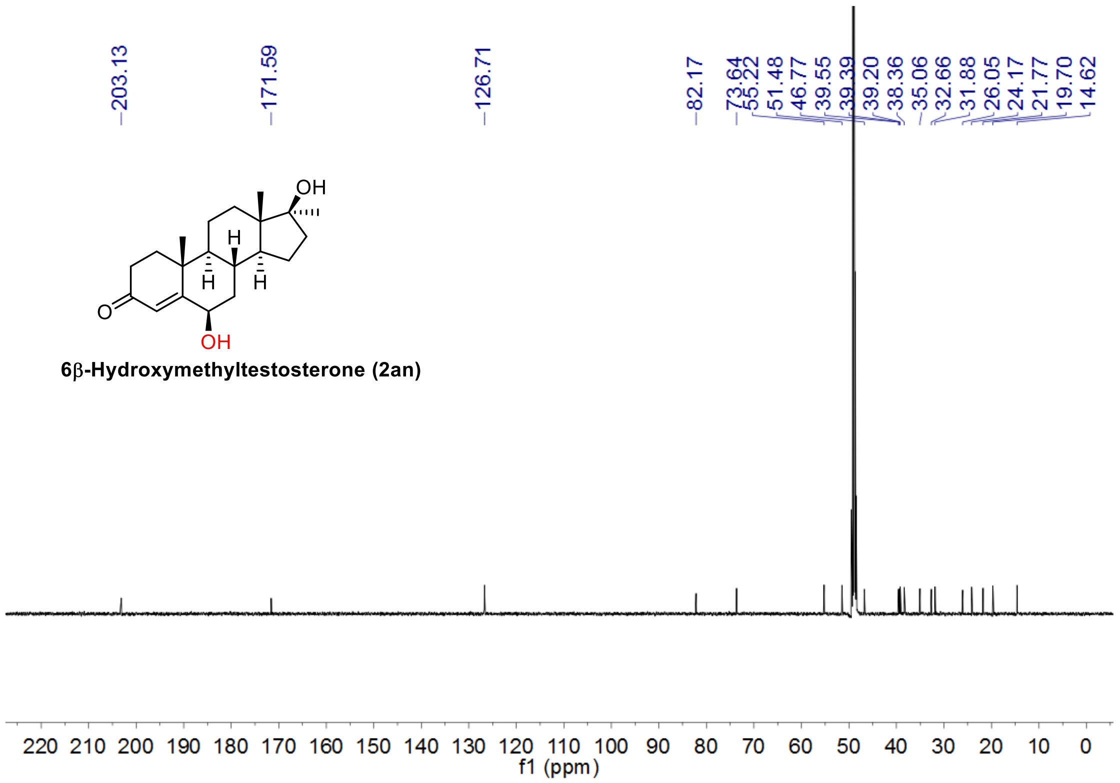

Supplementary Fig. 123. ^{13}C NMR spectrum of compound **2an (126 MHz, CD$_3$OD)**

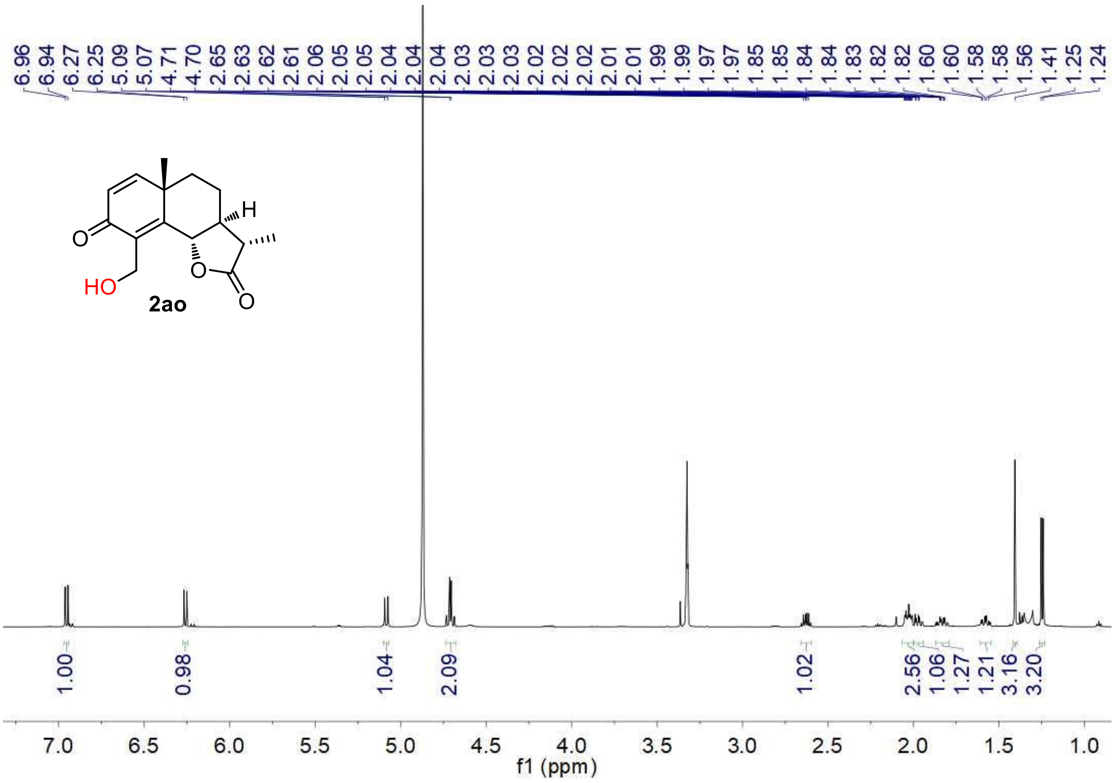

Supplementary Fig. 124. ^{1}H NMR spectrum of compound **2ao (600 MHz, CD_3OD)**

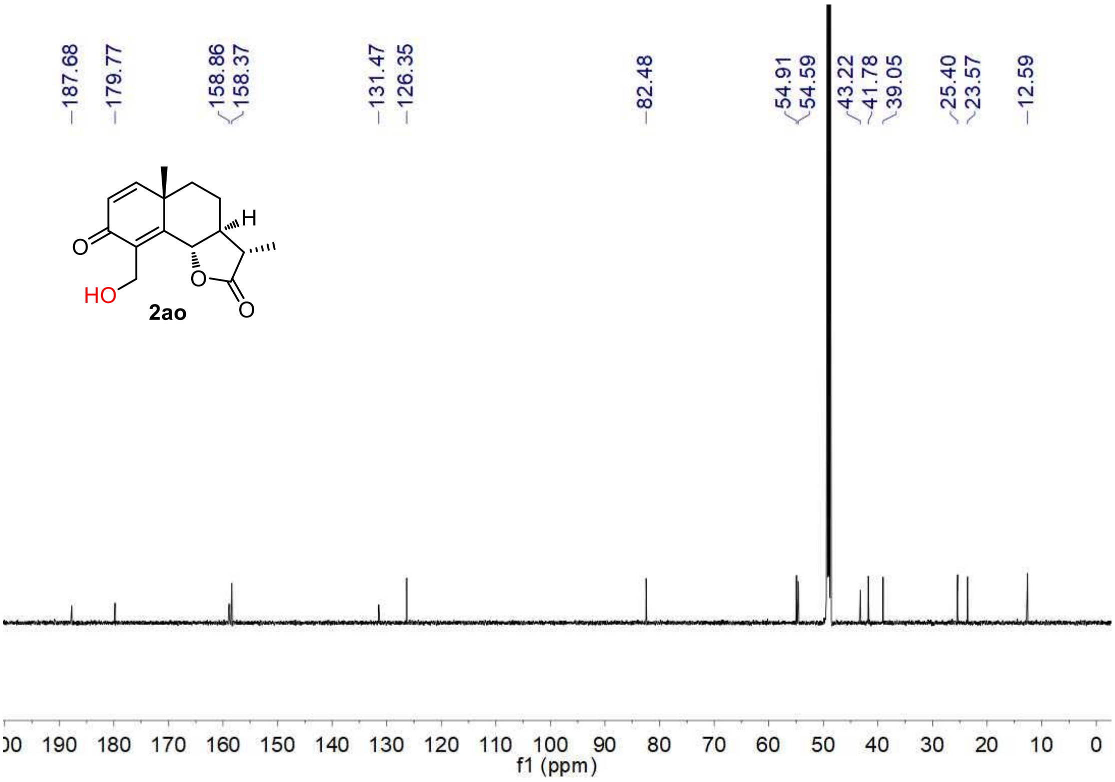

Supplementary Fig. 125. ^{13}C NMR spectrum of compound **2ao (151 MHz, CD_3OD)**

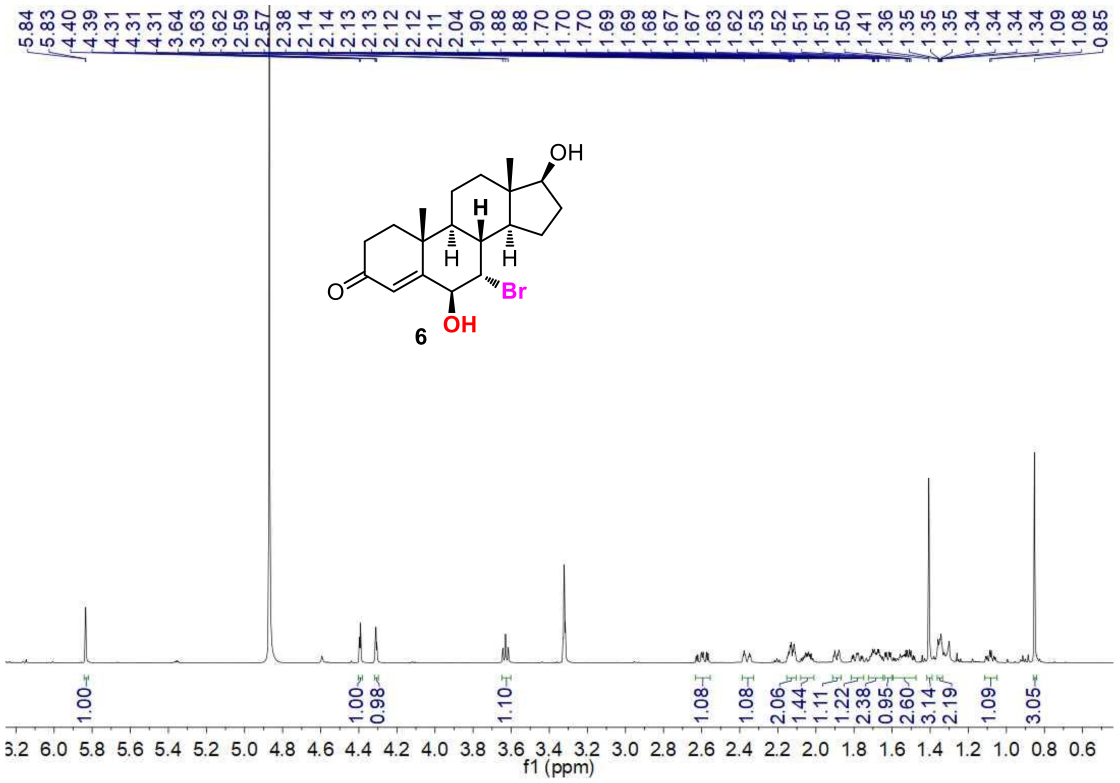

Supplementary Fig. 126. ^{1}H NMR spectrum of compound **6 (600 MHz, CD_3OD)**

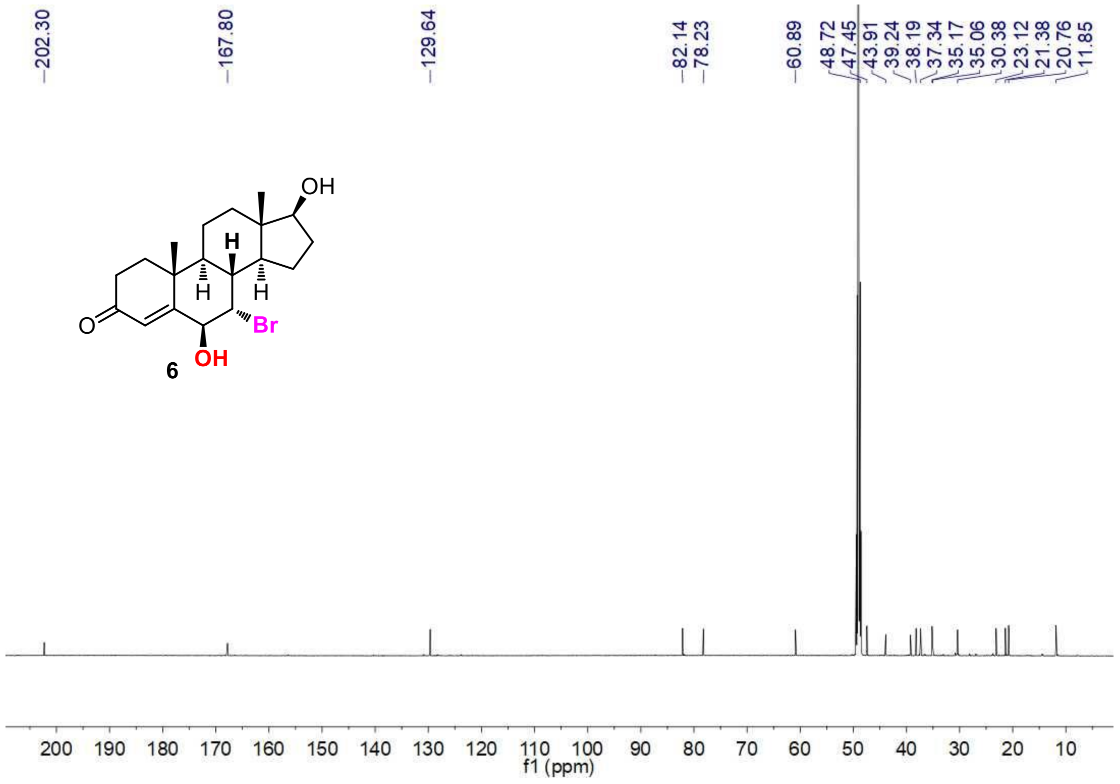

Supplementary Fig. 127. ^{13}C NMR spectrum of compound **6 (151 MHz, CD_3OD)**

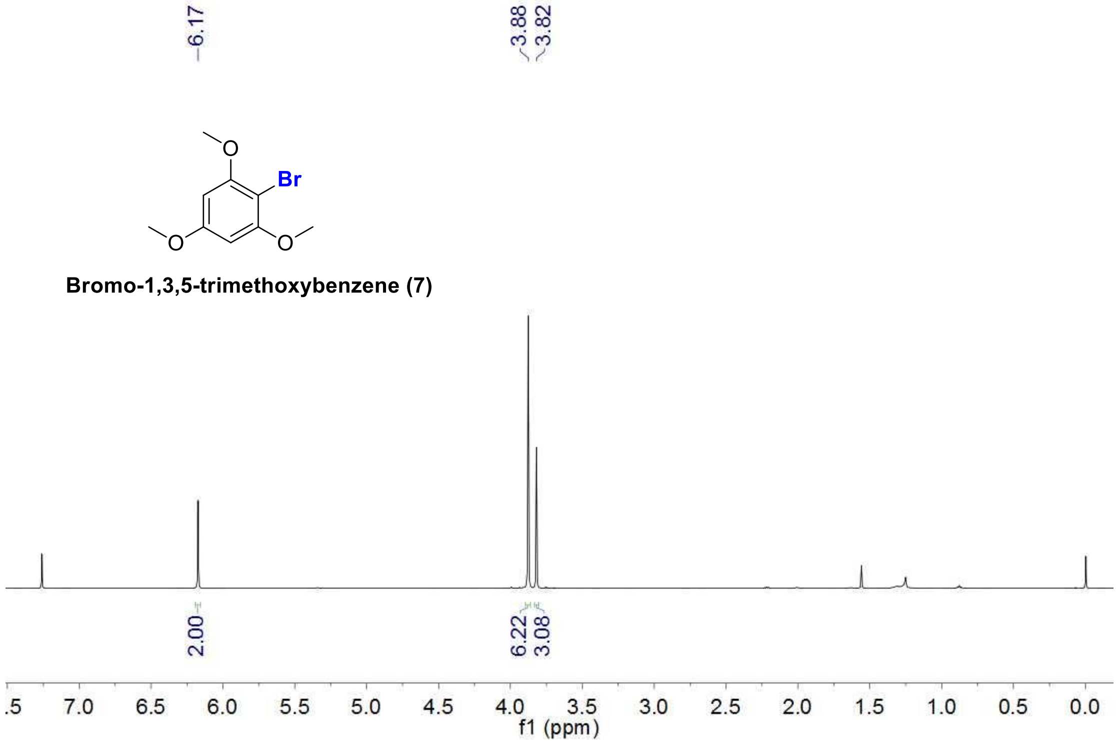

Supplementary Fig. 128. ^{1}H NMR spectrum of compound **7 (600 MHz, $CDCl_3$)**

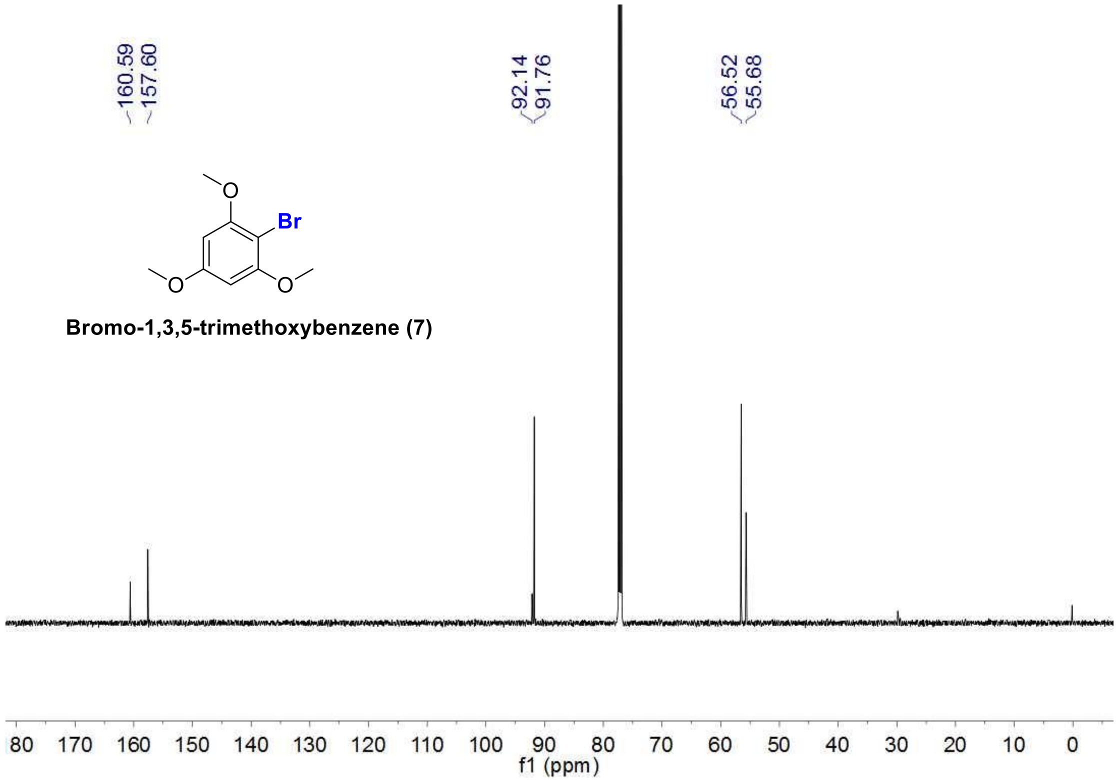

Supplementary Fig. 129. ^{13}C NMR spectrum of compound **7 (151 MHz, $CDCl_3$)**

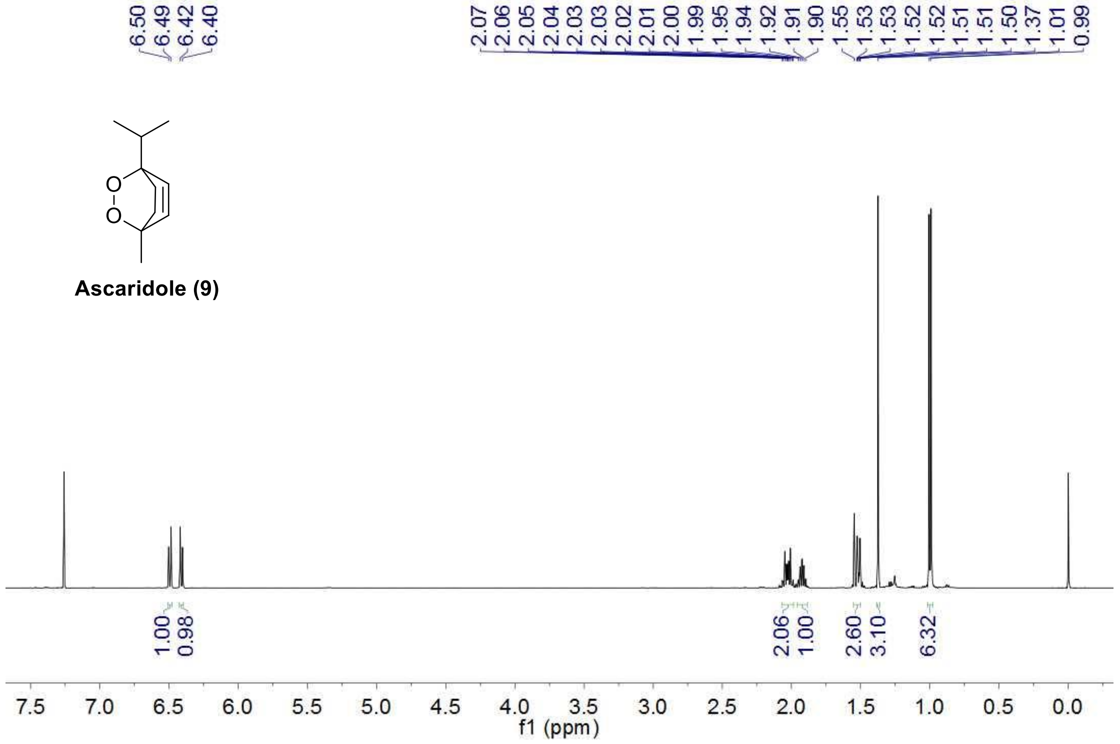

Supplementary Fig. 130. ^{1}H NMR spectrum of compound **9 (500 MHz, $CDCl_3$)**

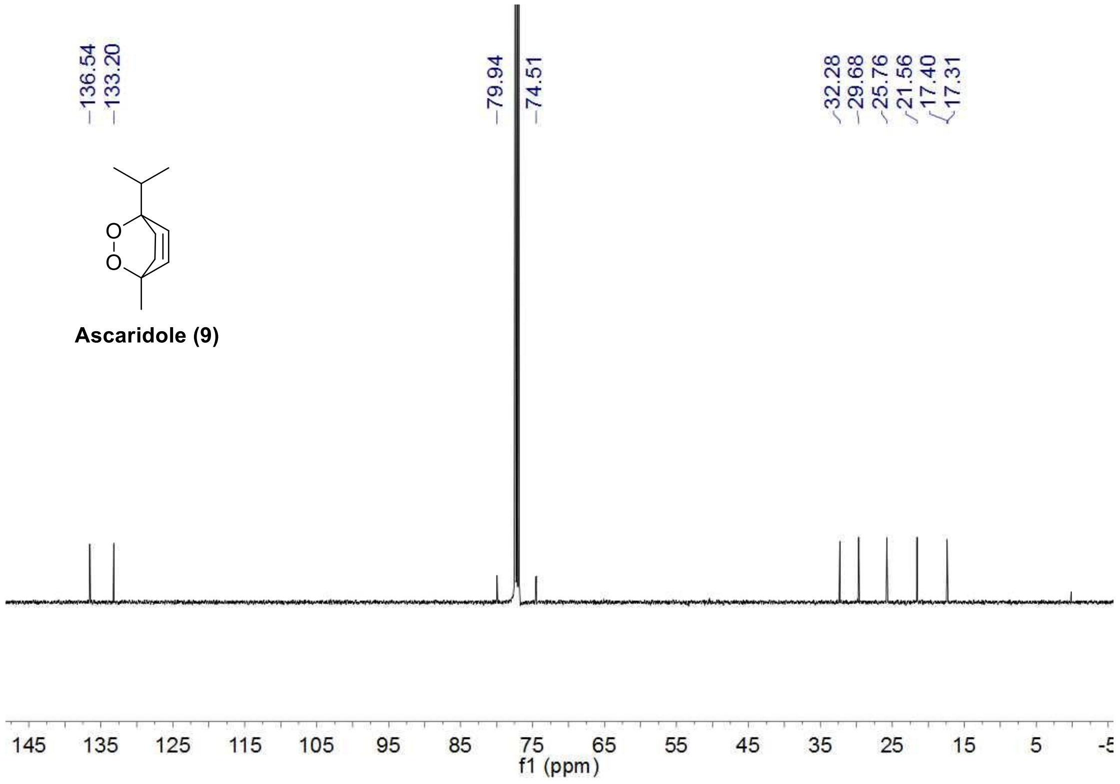

Supplementary Fig. 131. ^{13}C NMR spectrum of compound **9 (126 MHz, $CDCl_3$)**

3. Supplementary Notes

3.1 Details of the prices of reagents

Reagent price based on:

AAblocks,

Spironolactone (**1a**), https://www.aablocks.com/prod/52-01-7;

6β-Hydroxyspironolactone (**2a**), https://www.aablocks.com/prod/880106-10-5;

Eplerenone (**1y**), https://www.aablocks.com/prod/107724-20-9;

6β-Hydroxyeplerenone (**2y**), https://www.aablocks.com/prod/209253-80-5 (accessed June 16, 2022).

Toronto Research Chemicals,

Medroxyprogesterone acetate (**1ab**),

https://www.trc-canada.com/product-detail/?M203560;

6β-Hydroxymedroxyprogesterone acetate (**2ab-1**),

https://www.trc-canada.com/product-detail/?H944505;

6α-Hydroxymedroxyprogesterone acetate (**2ab-2**),

https://www.trc-canada.com/product-detail/?H944500 (accessed June 16, 2022).

Biosynth Carbosynth,

Levonorgestrel (**1aa**), https://www.carbosynth.com/p/FN26454/797-63-7-d-norgestrel;

10β-Hydroxylevonorgestrel (**2aa**),

https://www.carbosynth.com/p/WAA50850/21508-50-9-10v-hydroxy-d-norgestrel (accessed June 16, 2022).

4. Supplementary References

1. Yang, Y., Gao, X., Zeng, X., Han, J. & Xu, B. Hydrogen-bond-donor solvents enable catalyst-free (radio)-halogenation and deuteration of organoborons. *Chem. Eur. J.* **27**, 1297–1300 (2021).
2. Khong, M. T., Berl, V., Kuhn, L., Hammann, P. & Lepoittevin, J. P. Chemical modifications induced by phthalic anhydride, a respiratory sensitizer, in reconstructed human epidermis: A combined HRMAS NMR and LC-MS/MS proteomic approach. *Chem. Res. Toxicol.* **34**, 2087–2099 (2021).
3. Schilling, W. et al. Nature inspired singlet oxygen generation to access α-amino carbonyl compounds via 1,2-acyl migration. *Green Chem.* **23**, 379–387 (2021).
4. Rothenberg, G., Yatziv, Y. & Sasson, Y. Comparative autoxidation of 3-carene and α-pinene: factors governing regioselective hydrogen abstraction reactions. *Tetrahedron* **54**, 593–598 (1998).
5. Ando, M., Akahane, A. & Takase, K. Studies on the syntheses of sesquiterpene lactones. I. chemical transformation of α-santonin into vulgarin, C4-epivulgarin, and arglanine. *Bull. Chem. Soc. Jpn.* **51**, 283–289 (1978).
6. Blay, G., Luz Cardona, M., Garcia, B. & Pedro, J. R. Functionality transfer from C6 to C8 in sesquiterpenes. Synthesis of 8-epi-ivangustin and 8-epi-isoivangustin from santonin. *J. Org. Chem.* **56**, 6172–6175 (1991).
7. Suenaga, K., Takayanagi, Y., Yamaura, M. & Kigoshi, H. Total synthesis of (–)-ent-jolkinolide D. *Chem. Lett.* **33**, 918–919 (2004).
8. Herkommer, D. et al. Development of an improved route to a human immunodeficiency virus maturation inhibitor by chromium-free allylic oxidation and an efficient asymmetric Henry reaction. *Org. Process Res. Dev.* **26**, 288–298 (2022).
9. Highet, R. J. et al. Carbon-13 nuclear magnetic resonance studies of spironolactone and several related steroids. *Steroids* **35**, 119–132 (1980).
10. Schaub, R. E. & Weiss, M. J. The synthesis of certain 7α-alkylthio and 7α-acylthio steroid hormone derivatives. *J. Org. Chem.* **26**, 3915–3925 (1961).
11. Tweit, R. C. Configurations of 1- and 7-acetylthio-4-androstene-3,17-diones. *J. Org. Chem.* **27**, 2693–2694 (1962).
12. Roleira, F. M. F. et al. C-6α- vs C-7α-substituted steroidal aromatase inhibitors: which is better? synthesis, biochemical evaluation, docking studies, and structure-activity relationships. *J. Med. Chem.* **62**, 3636–3657 (2019).
13. Li, C. et al. Stereoselective synthesis of some methyl-substituted steroid hormones and their in vitro cytotoxic activity against human gastric cancer cell line MGC-803. *Steroids* **75**, 859–869 (2010).
14. Shing, T. K., Yeung, Y. Y. & Su, P. L. Mild manganese(III) acetate catalyzed allylic oxidation: application to simple and complex alkenes. *Org. Lett.* **8**, 3149–3151 (2006).
15. Chen, H., Wang, Y. F., Yang, Z. D. & Li, Y. C. Isolation and identification of novel impurities in spironolactone. *J. Pharm. Biomed. Anal.* **40**, 1263–1267

(2006).
16. Rafferty, R. J. & Williams, R. M. Synthetic studies on the ambiguine family of alkaloids: construction of the ABCD ring system. *Tetrahedron Lett.* **52**, 2037–2040 (2011).
17. Dezvarei, S., Lee, J. H. Z. & Bell, S. G. Stereoselective hydroxylation of isophorone by variants of the cytochromes P450 CYP102A1 and CYP101A1. *Enzyme. Microb. Technol.* **111**, 29–37 (2018).
18. Delort, E. et al. Comparative analysis of three Australian finger lime (*Citrus australasica*) cultivars: identification of unique citrus chemotypes and new volatile molecules. *Phytochemistry* **109**, 111–124 (2015).
19. Sarott, R. C. et al. Optical control of cannabinoid receptor 2-mediated Ca^{2+} release enabled by synthesis of photoswitchable probes. *J. Am. Chem. Soc.* **143**, 736–743 (2021).
20. Meng, Z. & Liu, B. Total synthesis of five natural eremophilane-type sesquiterpenoids. *Org. Biomol. Chem.* **16**, 957–962 (2018).
21. Abegaz, B. et al. The structures of vulgarin and its isomers: —A reinvestigation. *Tetrahedron* **42**, 6003–6009 (1986).
22. Fessner, N. D. et al. Preparative-scale production of testosterone metabolites by human liver cytochrome P450 enzyme 3A4. *Adv. Synth. Catal.* **362**, 2725–2738 (2020).
23. Alarif, W. M., Ayyad, S.-E. N., El-Assouli, S. M. & Al-Lihaibi, S. S. Antigenotoxic ketosteroid from the red algae Jania adhaerens. *Nat. Prod. Res.* **26**, 785–791 (2012).
24. Fodouop Chegaing, S. P. et al. Fungal transformation of norandrostenedione with *Cunninghamella blakesleeana* and anti-bacterial activity of the transformed products. *Steroids* **162**, 108679 (2020).
25. Kolet, S. P., Niloferjahan, S., Haldar, S., Gonnade, R. & Thulasiram, H. V. Biocatalyst mediated production of 6β,11α-dihydroxy derivatives of 4-ene-3-one steroids. *Steroids* **78**, 1152–1158 (2013).
26. Guo, F., Feng, H., Wang, Y., Zhang, C. & Li, Y. Characterization of related impurities in megestrol acetate. *J. Pharm. Biomed. Anal.* **41**, 1418–1422 (2006).
