# Supplementary material for: Allylic hydroxylation of enones useful for the functionalization of relevant drugs and natural products
Source: Nat Commun. 2023 Apr 26;14:2399. doi: 10.1038/s41467-023-38154-9 (PMC10133259; doi:10.1038/s41467-023-38154-9)
Supplement: Supplementary file 2 — Description of Additional Supplementary Files [file 41467_2023_38154_MOESM2_ESM.docx]

Description of Additional Supplementary Files

File Name: Supplementary Data 1

Description: Crystal data and CheckCif reports for compounds **2l**, **2m**, **2o**, **2r,** **2ag,** **2ai,** **2aj-2,** and **6**.
