# Supplementary material for: Allylic hydroxylation of enones useful for the functionalization of relevant drugs and natural products
Source: Nat Commun. 2023 Apr 26;14:2399. doi: 10.1038/s41467-023-38154-9 (PMC10133259; doi:10.1038/s41467-023-38154-9)

checkCIF/PLATON report

Structure factors have been supplied for datablock(s) 1

THIS REPORT IS FOR GUIDANCE ONLY. IF USED AS PART OF A REVIEW PROCEDURE FOR PUBLICATION, IT SHOULD NOT REPLACE THE EXPERTISE OF AN EXPERIENCED CRYSTALLOGRAPHIC REFEREE.

No syntax errors found. CIF dictionary Interpreting this report

Datablock: 1

Bond precision:	C-C = 0.0032 A		Wavelength=1.54178
Cell:	a=8.4317(3)	b=12.4549(3)	c=40.4362(10)
	alpha=90	beta=90	gamma=90
Temperature:	170 K		

	Calculated	Reported
Volume	4246.5(2)	4246.4(2)
Space group	C 2 2 21	C 2 2 21
Hall group	C 2c 2	C 2c 2
Moiety formula	C24 H30 O7	?
Sum formula	C24 H30 O7	C24 H30 O7
Mr	430.48	430.48
Dx,g cm-3	1.347	1.347
Z	8	8
Mu (mm-1)	0.811	0.811
F000	1840.0	1840.0
F000’	1846.03	
h,k,lmax	10,15,50	10,15,49
Nref	4362[2460]	4232
Tmin,Tmax	0.925,0.960	
Tmin’	0.885	

Correction method= Not given

Data completeness= 1.72/0.97 Theta(max)= 74.348

R(reflections)= 0.0361(3982) wR2(reflections)= 0.0953(4232)

S = 1.047 Npar= 284

The following ALERTS were generated. Each ALERT has the format
test-name_ALERT_alert-type_alert-level.
Click on the hyperlinks for more details of the test.

Alert level C

```
PLAT052_ALERT_1_C Info on Absorption Correction Method   Not Given     Please Do !
PLAT094_ALERT_2_C Ratio of Maximum / Minimum Residual Density ....       2.62 Report
PLAT911_ALERT_3_C Missing FCF Refl Between Thmin & STh/L=    0.600         14 Report
```

Alert level G

```
PLAT007_ALERT_5_G Number of Unrefined Donor-H Atoms ..............          1 Report
PLAT398_ALERT_2_G Deviating  C-O-C   Angle From 120 for O5       .       61.2 Degree
PLAT791_ALERT_4_G Model has Chirality at C6          (Sohnke SpGr)          R Verify
PLAT791_ALERT_4_G Model has Chirality at C7          (Sohnke SpGr)          S Verify
PLAT791_ALERT_4_G Model has Chirality at C8          (Sohnke SpGr)          R Verify
PLAT791_ALERT_4_G Model has Chirality at C9          (Sohnke SpGr)          R Verify
PLAT791_ALERT_4_G Model has Chirality at C10         (Sohnke SpGr)          S Verify
PLAT791_ALERT_4_G Model has Chirality at C11         (Sohnke SpGr)          R Verify
PLAT791_ALERT_4_G Model has Chirality at C13         (Sohnke SpGr)          S Verify
PLAT791_ALERT_4_G Model has Chirality at C14         (Sohnke SpGr)          S Verify
PLAT791_ALERT_4_G Model has Chirality at C17         (Sohnke SpGr)          R Verify
PLAT883_ALERT_1_G No Info/Value for _atom_sites_solution_primary .     Please Do !
PLAT912_ALERT_4_G Missing # of FCF Reflections Above STh/L=  0.600         12 Note
PLAT913_ALERT_3_G Missing # of Very Strong Reflections in FCF ....          1 Note
PLAT978_ALERT_2_G Number C-C Bonds with Positive Residual Density.         16 Info
```

0 **ALERT level A** = Most likely a serious problem - resolve or explain
0 **ALERT level B** = A potentially serious problem, consider carefully
3 **ALERT level C** = Check. Ensure it is not caused by an omission or oversight
15 **ALERT level G** = General information/check it is not something unexpected

2 ALERT type 1 CIF construction/syntax error, inconsistent or missing data
3 ALERT type 2 Indicator that the structure model may be wrong or deficient
2 ALERT type 3 Indicator that the structure quality may be low
10 ALERT type 4 Improvement, methodology, query or suggestion
1 ALERT type 5 Informative message, check

It is advisable to attempt to resolve as many as possible of the alerts in all categories. Often the minor alerts point to easily fixed oversights, errors and omissions in your CIF or refinement strategy, so attention to these fine details can be worthwhile. In order to resolve some of the more serious problems it may be necessary to carry out additional measurements or structure refinements. However, the purpose of your study may justify the reported deviations and the more serious of these should normally be commented upon in the discussion or experimental section of a paper or in the "special_details" fields of the CIF. checkCIF was carefully designed to identify outliers and unusual parameters, but every test has its limitations and alerts that are not important in a particular case may appear. Conversely, the absence of alerts does not guarantee there are no aspects of the results needing attention. It is up to the individual to critically assess their own results and, if necessary, seek expert advice.

Publication of your CIF in IUCr journals

A basic structural check has been run on your CIF. These basic checks will be run on all CIFs submitted for publication in IUCr journals (*Acta Crystallographica*, *Journal of Applied Crystallography*, *Journal of Synchrotron Radiation*); however, if you intend to submit to *Acta Crystallographica Section C* or *E* or *IUCrData*, you should make sure that full publication checks are run on the final version of your CIF prior to submission.

Publication of your CIF in other journals

Please refer to the *Notes for Authors* of the relevant journal for any special instructions relating to CIF submission.

PLATON version of 18/05/2022; check.def file version of 17/05/2022

Datablock 1 - ellipsoid plot

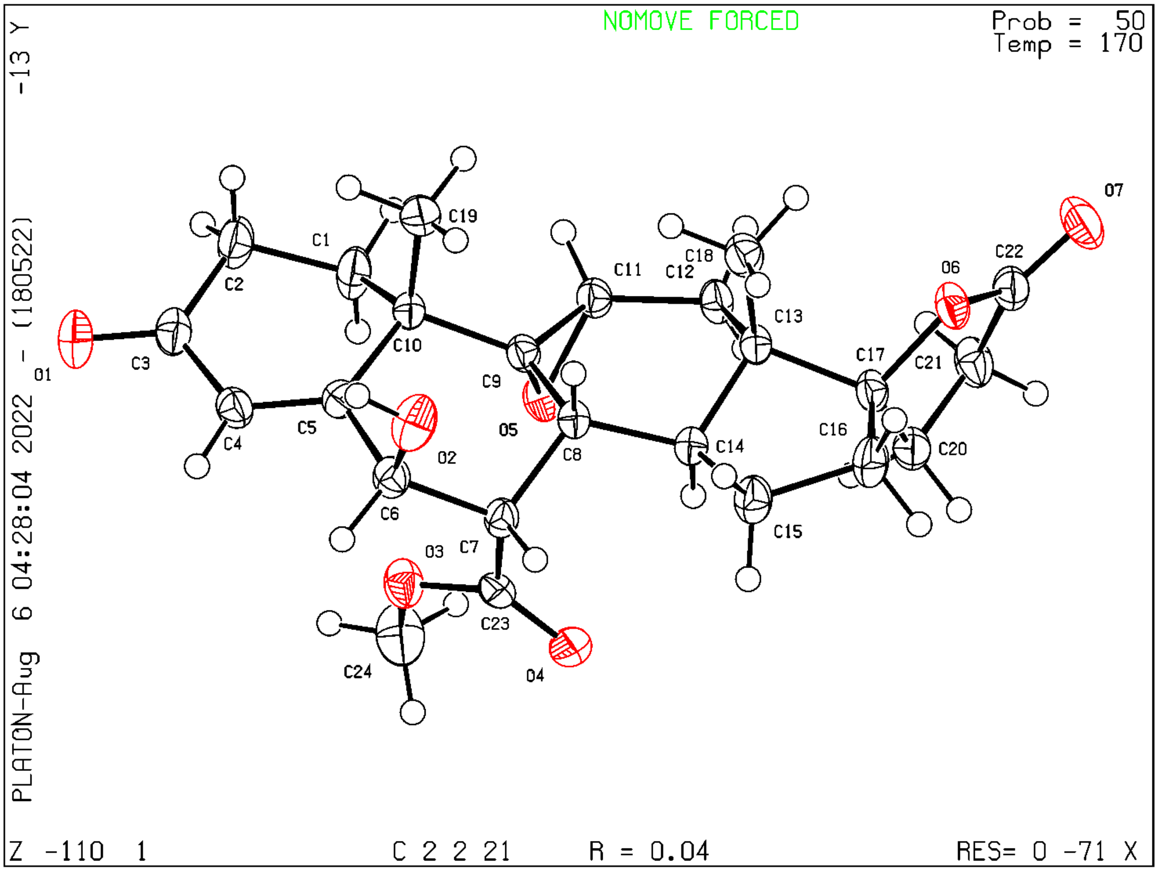

Supplement: Supplementary file 3 — Supplementary Data 1 [file 41467_2023_38154_MOESM3_ESM.zip › CheckCif output for compound 2ag.pdf]
