# Supplementary material for: Allylic hydroxylation of enones useful for the functionalization of relevant drugs and natural products
Source: Nat Commun. 2023 Apr 26;14:2399. doi: 10.1038/s41467-023-38154-9 (PMC10133259; doi:10.1038/s41467-023-38154-9)

checkCIF/PLATON report

Structure factors have been supplied for datablock(s) cu_20221487_0m

THIS REPORT IS FOR GUIDANCE ONLY. IF USED AS PART OF A REVIEW PROCEDURE FOR PUBLICATION, IT SHOULD NOT REPLACE THE EXPERTISE OF AN EXPERIENCED CRYSTALLOGRAPHIC REFEREE.

No syntax errors found. CIF dictionary Interpreting this report

Datablock: cu_20221487_0m

```
Bond precision:   C-C = 0.0029 A          Wavelength=1.54178

Cell:              a=7.6687(3)      b=14.4724(6)      c=15.8033(7)
                   alpha=90         beta=90           gamma=90
Temperature:       100 K

                 Calculated                   Reported
Volume           1753.92(13)                  1753.92(13)
Space group      P 21 21 21                   P 21 21 21
Hall group       P 2ac 2ab                    P 2ac 2ab
Moiety formula   C20 H30 O4                   C20 H30 O4
Sum formula      C20 H30 O4                   C20 H30 O4
Mr               334.44                       334.44
Dx,g cm-3        1.266                        1.267
Z                4                            4
Mu (mm-1)        0.692                        0.692
F000             728.0                        728.0
F000'            730.16
h,k,lmax         9,18,19                      9,17,19
Nref             3596[ 2068]                  3500
Tmin,Tmax        0.936,0.966                  0.661,0.754
Tmin'            0.920

Correction method= # Reported T Limits: Tmin=0.661 Tmax=0.754
AbsCorr = NONE

Data completeness= 1.69/0.97       Theta(max)= 74.568

R(reflections)= 0.0330( 3313)                         wR2(reflections)=
                                                      0.0898( 3500)
S = 1.066                  Npar= 222
```

The following ALERTS were generated. Each ALERT has the format
test-name_ALERT_alert-type_alert-level.
Click on the hyperlinks for more details of the test.

Alert level C

```
PLAT911_ALERT_3_C Missing FCF Refl Between Thmin & STh/L=    0.600          21 Report
PLAT934_ALERT_3_C Number of (Iobs-Icalc)/Sigma(W) > 10 Outliers ..           1 Check
```

Alert level G

```
PLAT007_ALERT_5_G Number of Unrefined Donor-H Atoms .............            1 Report
PLAT791_ALERT_4_G Model has Chirality at C5          (Sohnke SpGr)           R Verify
PLAT791_ALERT_4_G Model has Chirality at C8          (Sohnke SpGr)           S Verify
PLAT791_ALERT_4_G Model has Chirality at C9          (Sohnke SpGr)           S Verify
PLAT791_ALERT_4_G Model has Chirality at C10         (Sohnke SpGr)           R Verify
PLAT791_ALERT_4_G Model has Chirality at C13         (Sohnke SpGr)           S Verify
PLAT883_ALERT_1_G No Info/Value for _atom_sites_solution_primary .     Please Do !
PLAT912_ALERT_4_G Missing # of FCF Reflections Above STh/L=  0.600          11 Note
PLAT978_ALERT_2_G Number C-C Bonds with Positive Residual Density.          13 Info
PLAT992_ALERT_5_G Repd & Actual _reflns_number_gt Values Differ by           4 Check
```

0 **ALERT level A** = Most likely a serious problem - resolve or explain
0 **ALERT level B** = A potentially serious problem, consider carefully
2 **ALERT level C** = Check. Ensure it is not caused by an omission or oversight
10 **ALERT level G** = General information/check it is not something unexpected

1 ALERT type 1 CIF construction/syntax error, inconsistent or missing data
1 ALERT type 2 Indicator that the structure model may be wrong or deficient
2 ALERT type 3 Indicator that the structure quality may be low
6 ALERT type 4 Improvement, methodology, query or suggestion
2 ALERT type 5 Informative message, check

It is advisable to attempt to resolve as many as possible of the alerts in all categories. Often the minor alerts point to easily fixed oversights, errors and omissions in your CIF or refinement strategy, so attention to these fine details can be worthwhile. In order to resolve some of the more serious problems it may be necessary to carry out additional measurements or structure refinements. However, the purpose of your study may justify the reported deviations and the more serious of these should normally be commented upon in the discussion or experimental section of a paper or in the "special_details" fields of the CIF. checkCIF was carefully designed to identify outliers and unusual parameters, but every test has its limitations and alerts that are not important in a particular case may appear. Conversely, the absence of alerts does not guarantee there are no aspects of the results needing attention. It is up to the individual to critically assess their own results and, if necessary, seek expert advice.

Publication of your CIF in IUCr journals

A basic structural check has been run on your CIF. These basic checks will be run on all CIFs submitted for publication in IUCr journals (*Acta Crystallographica*, *Journal of Applied Crystallography*, *Journal of Synchrotron Radiation*); however, if you intend to submit to *Acta Crystallographica Section C* or *E* or *IUCrData*, you should make sure that full publication checks are run on the final version of your CIF prior to submission.

Publication of your CIF in other journals

Please refer to the *Notes for Authors* of the relevant journal for any special instructions relating to CIF submission.

PLATON version of 12/09/2022; check.def file version of 09/08/2022

Datablock cu_20221487_0m - ellipsoid plot

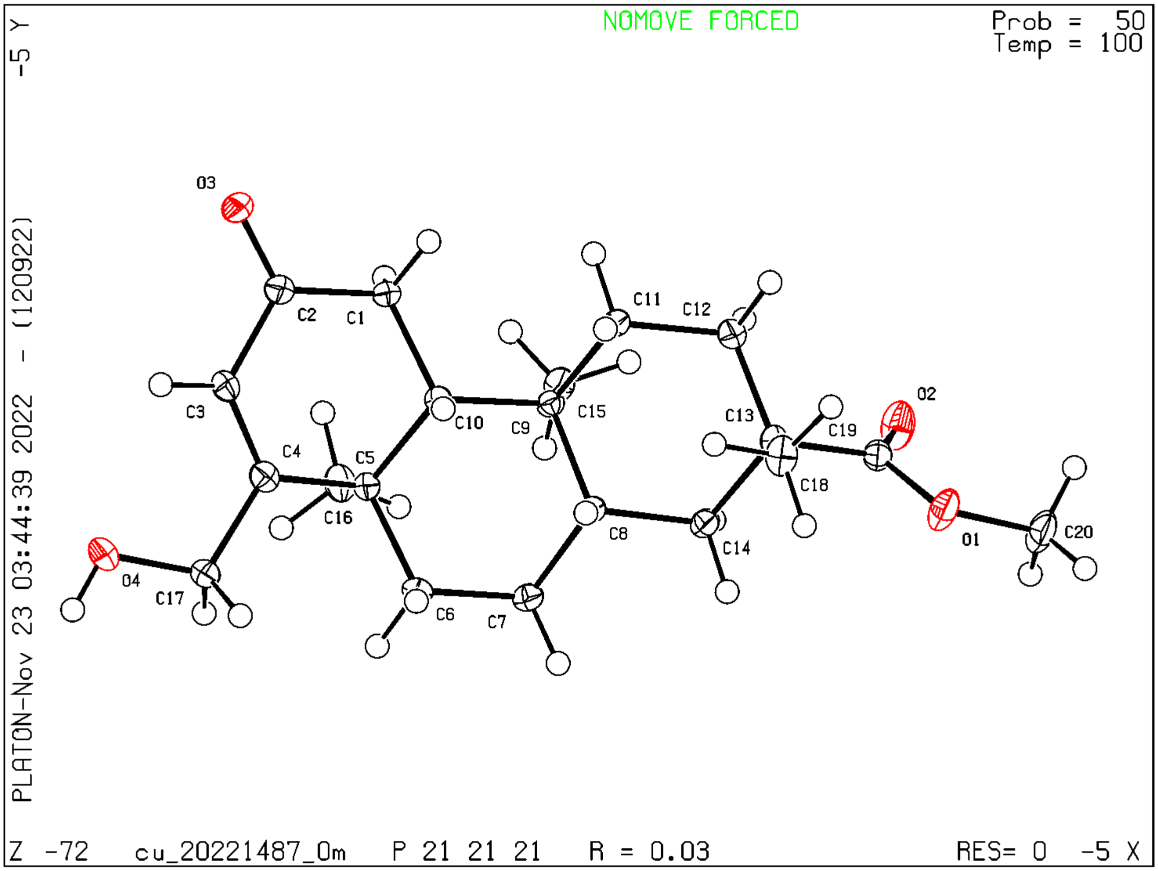

Supplement: Supplementary file 3 — Supplementary Data 1 [file 41467_2023_38154_MOESM3_ESM.zip › CheckCif output for compound 2o.pdf]
