# Supplementary material for: Allylic hydroxylation of enones useful for the functionalization of relevant drugs and natural products
Source: Nat Commun. 2023 Apr 26;14:2399. doi: 10.1038/s41467-023-38154-9 (PMC10133259; doi:10.1038/s41467-023-38154-9)

checkCIF/PLATON report

Structure factors have been supplied for datablock(s) cu_20221475_0m

THIS REPORT IS FOR GUIDANCE ONLY. IF USED AS PART OF A REVIEW PROCEDURE FOR PUBLICATION, IT SHOULD NOT REPLACE THE EXPERTISE OF AN EXPERIENCED CRYSTALLOGRAPHIC REFEREE.

No syntax errors found. CIF dictionary Interpreting this report

Datablock: cu_20221475_0m

```
Bond precision:   C-C = 0.0086 A          Wavelength=1.54178

Cell:             a=15.3660(5)     b=11.8114(3)       c=34.1656(10)
                  alpha=90         beta=95.902(2)     gamma=90
Temperature:      170 K

                 Calculated                  Reported
Volume           6168.0(3)                   6168.0(3)
Space group      P 21                        P 1 21 1
Hall group       P 2yb                       P 2yb
Moiety formula   C34 H52 O6                  C34 H52 O6
Sum formula      C34 H52 O6                  C34 H52 O6
Mr               556.76                      556.75
Dx,g cm-3        1.199                       1.199
Z                8                           8
Mu (mm-1)        0.637                       0.637
F000             2432.0                      2432.0
F000'            2439.02
h,k,lmax         19,14,42                    19,14,42
Nref             25308[ 13297]               23262
Tmin,Tmax        0.941,0.969                 0.641,0.754
Tmin'            0.926

Correction method= # Reported T Limits: Tmin=0.641 Tmax=0.754
AbsCorr = NONE

Data completeness= 1.75/0.92       Theta(max)= 74.695

R(reflections)= 0.0818( 20267)                  wR2(reflections)=
                                                0.2487( 23262)
S = 1.030                  Npar= 1481
```

The following ALERTS were generated. Each ALERT has the format
test-name_ALERT_alert-type_alert-level.
Click on the hyperlinks for more details of the test.

Alert level C

```
PLAT220_ALERT_2_C NonSolvent   Resd 1  O   Ueq(max)/Ueq(min) Range        3.1 Ratio
PLAT220_ALERT_2_C NonSolvent   Resd 2  C   Ueq(max)/Ueq(min) Range        3.1 Ratio
PLAT220_ALERT_2_C NonSolvent   Resd 2  O   Ueq(max)/Ueq(min) Range        3.3 Ratio
PLAT220_ALERT_2_C NonSolvent   Resd 4  O   Ueq(max)/Ueq(min) Range        3.1 Ratio
PLAT241_ALERT_2_C High   'MainMol' Ueq as Compared to Neighbors of       C03I Check
PLAT242_ALERT_2_C Low    'MainMol' Ueq as Compared to Neighbors of        C29 Check
PLAT242_ALERT_2_C Low    'MainMol' Ueq as Compared to Neighbors of       C04F Check
PLAT242_ALERT_2_C Low    'MainMol' Ueq as Compared to Neighbors of       C02M Check
PLAT340_ALERT_3_C Low Bond Precision on  C-C Bonds ...............    0.00865 Ang.
PLAT412_ALERT_2_C Short Intra XH3 .. XHn     H9A      ..H34C     .       1.87 Ang.
                                                       x,y,z  =      1_555 Check
PLAT412_ALERT_2_C Short Intra XH3 .. XHn     H00F     ..H016     .       1.89 Ang.
                                                       x,y,z  =      1_555 Check
PLAT601_ALERT_2_C Unit Cell Contains Solvent Accessible VOIDS of .         39 Ang**3
PLAT906_ALERT_3_C Large K Value in the Analysis of Variance ......      2.225 Check
PLAT911_ALERT_3_C Missing FCF Refl Between Thmin & STh/L=    0.600         70 Report
```

Alert level G

```
PLAT007_ALERT_5_G Number of Unrefined Donor-H Atoms ..............          4 Report
PLAT072_ALERT_2_G SHELXL First  Parameter in WGHT  Unusually Large       0.15 Report
PLAT083_ALERT_2_G SHELXL Second Parameter in WGHT  Unusually Large       5.90 Why ?
PLAT720_ALERT_4_G Number of Unusual/Non-Standard Labels ..........        265 Note
PLAT790_ALERT_4_G Centre of Gravity not Within Unit Cell: Resd.  #          3 Note
              C34 H52 O6
PLAT790_ALERT_4_G Centre of Gravity not Within Unit Cell: Resd.  #          4 Note
              C34 H52 O6
PLAT791_ALERT_4_G Model has Chirality at C00M        (Sohnke SpGr)          R Verify
PLAT791_ALERT_4_G Model has Chirality at C3          (Sohnke SpGr)          S Verify
PLAT791_ALERT_4_G Model has Chirality at C00R        (Sohnke SpGr)          S Verify
PLAT791_ALERT_4_G Model has Chirality at C5          (Sohnke SpGr)          R Verify
PLAT791_ALERT_4_G Model has Chirality at C00X        (Sohnke SpGr)          S Verify
PLAT791_ALERT_4_G Model has Chirality at C8          (Sohnke SpGr)          R Verify
PLAT791_ALERT_4_G Model has Chirality at C00Y        (Sohnke SpGr)          S Verify
PLAT791_ALERT_4_G Model has Chirality at C9          (Sohnke SpGr)          R Verify
PLAT791_ALERT_4_G Model has Chirality at C00Z        (Sohnke SpGr)          R Verify
PLAT791_ALERT_4_G Model has Chirality at C10         (Sohnke SpGr)          S Verify
PLAT791_ALERT_4_G Model has Chirality at C012        (Sohnke SpGr)          S Verify
PLAT791_ALERT_4_G Model has Chirality at C13         (Sohnke SpGr)          S Verify
PLAT791_ALERT_4_G Model has Chirality at C14         (Sohnke SpGr)          S Verify
PLAT791_ALERT_4_G Model has Chirality at C014        (Sohnke SpGr)          S Verify
PLAT791_ALERT_4_G Model has Chirality at C016        (Sohnke SpGr)          R Verify
PLAT791_ALERT_4_G Model has Chirality at C018        (Sohnke SpGr)          S Verify
PLAT791_ALERT_4_G Model has Chirality at C17         (Sohnke SpGr)          R Verify
PLAT791_ALERT_4_G Model has Chirality at C01B        (Sohnke SpGr)          R Verify
PLAT791_ALERT_4_G Model has Chirality at C01D        (Sohnke SpGr)          R Verify
PLAT791_ALERT_4_G Model has Chirality at C01O        (Sohnke SpGr)          R Verify
PLAT791_ALERT_4_G Model has Chirality at C01S        (Sohnke SpGr)          S Verify
PLAT791_ALERT_4_G Model has Chirality at C01T        (Sohnke SpGr)          R Verify
PLAT791_ALERT_4_G Model has Chirality at C020        (Sohnke SpGr)          S Verify
```

```
PLAT791_ALERT_4_G Model has Chirality at C027        (Sohnke SpGr)          S Verify
PLAT791_ALERT_4_G Model has Chirality at C02E        (Sohnke SpGr)          S Verify
PLAT791_ALERT_4_G Model has Chirality at C02G        (Sohnke SpGr)          R Verify
PLAT791_ALERT_4_G Model has Chirality at C02O        (Sohnke SpGr)          S Verify
PLAT791_ALERT_4_G Model has Chirality at C02X        (Sohnke SpGr)          S Verify
PLAT791_ALERT_4_G Model has Chirality at C034        (Sohnke SpGr)          R Verify
PLAT791_ALERT_4_G Model has Chirality at C035        (Sohnke SpGr)          R Verify
PLAT791_ALERT_4_G Model has Chirality at C040        (Sohnke SpGr)          R Verify
PLAT791_ALERT_4_G Model has Chirality at C043        (Sohnke SpGr)          R Verify
PLAT883_ALERT_1_G No Info/Value for _atom_sites_solution_primary .     Please Do !
PLAT910_ALERT_3_G Missing # of FCF Reflection(s) Below Theta(Min).          1 Note
PLAT912_ALERT_4_G Missing # of FCF Reflections Above STh/L=  0.600        165 Note
PLAT933_ALERT_2_G Number of HKL-OMIT Records in Embedded .res File         10 Note
PLAT941_ALERT_3_G Average HKL Measurement Multiplicity ..........         4.3 Low
PLAT978_ALERT_2_G Number C-C Bonds with Positive Residual Density.          1 Info
```

---

   0 **ALERT level A** = Most likely a serious problem - resolve or explain
   0 **ALERT level B** = A potentially serious problem, consider carefully
  14 **ALERT level C** = Check. Ensure it is not caused by an omission or oversight
  44 **ALERT level G** = General information/check it is not something unexpected

```
   1 ALERT type 1 CIF construction/syntax error, inconsistent or missing data
  15 ALERT type 2 Indicator that the structure model may be wrong or deficient
   5 ALERT type 3 Indicator that the structure quality may be low
  36 ALERT type 4 Improvement, methodology, query or suggestion
   1 ALERT type 5 Informative message, check
```

It is advisable to attempt to resolve as many as possible of the alerts in all categories. Often the minor alerts point to easily fixed oversights, errors and omissions in your CIF or refinement strategy, so attention to these fine details can be worthwhile. In order to resolve some of the more serious problems it may be necessary to carry out additional measurements or structure refinements. However, the purpose of your study may justify the reported deviations and the more serious of these should normally be commented upon in the discussion or experimental section of a paper or in the "special_details" fields of the CIF. checkCIF was carefully designed to identify outliers and unusual parameters, but every test has its limitations and alerts that are not important in a particular case may appear. Conversely, the absence of alerts does not guarantee there are no aspects of the results needing attention. It is up to the individual to critically assess their own results and, if necessary, seek expert advice.

Publication of your CIF in IUCr journals

A basic structural check has been run on your CIF. These basic checks will be run on all CIFs submitted for publication in IUCr journals (*Acta Crystallographica*, *Journal of Applied Crystallography*, *Journal of Synchrotron Radiation*); however, if you intend to submit to *Acta Crystallographica Section C* or *E* or *IUCrData*, you should make sure that full publication checks are run on the final version of your CIF prior to submission.

Publication of your CIF in other journals

Please refer to the *Notes for Authors* of the relevant journal for any special instructions relating to CIF submission.

PLATON version of 12/09/2022; check.def file version of 09/08/2022

Datablock cu_20221475_0m - ellipsoid plot

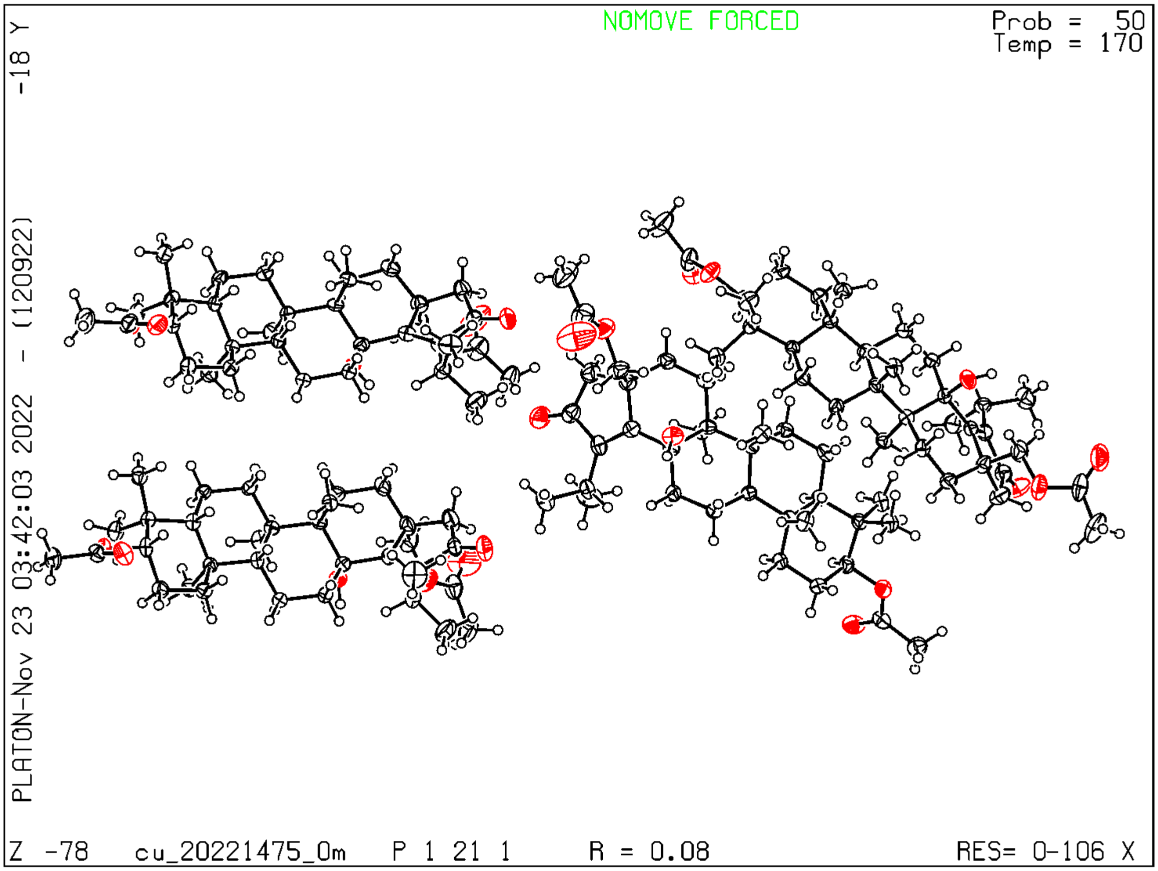

Supplement: Supplementary file 3 — Supplementary Data 1 [file 41467_2023_38154_MOESM3_ESM.zip › CheckCif output for compound 2r.pdf]
