# Supplementary material for: Allylic hydroxylation of enones useful for the functionalization of relevant drugs and natural products
Source: Nat Commun. 2023 Apr 26;14:2399. doi: 10.1038/s41467-023-38154-9 (PMC10133259; doi:10.1038/s41467-023-38154-9)

checkCIF/PLATON report

Structure factors have been supplied for datablock(s) cu_20211147_0m

THIS REPORT IS FOR GUIDANCE ONLY. IF USED AS PART OF A REVIEW PROCEDURE FOR PUBLICATION, IT SHOULD NOT REPLACE THE EXPERTISE OF AN EXPERIENCED CRYSTALLOGRAPHIC REFEREE.

No syntax errors found. CIF dictionary Interpreting this report

Datablock: cu_20211147_0m

Bond precision:	C-C = 0.0101 A		Wavelength=1.54178
Cell:	a=10.2815(9)	b=6.5838(6)	c=26.449(2)
	alpha=90	beta=90.101(4)	gamma=90
Temperature:	170 K		

	Calculated	Reported
Volume	1790.4(3)	1790.4(3)
Space group	P 21	P 21
Hall group	P 2yb	P 2yb
Moiety formula	C19 H27 Br O3	?
Sum formula	C19 H27 Br O3	C19 H27 Br O3
Mr	383.31	383.31
Dx,g cm-3	1.422	1.422
Z	4	4
Mu (mm-1)	3.226	3.226
F000	800.0	800.0
F000’	799.18	
h,k,lmax	12,8,33	12,7,33
Nref	7370[4018]	6751
Tmin,Tmax	0.770,0.851	
Tmin’	0.587	

Correction method= Not given

Data completeness= 1.68/0.92 Theta(max)= 74.928

R(reflections)= 0.0573(5895) wR2(reflections)= 0.1525(6751)

S = 1.068 Npar= 423

The following ALERTS were generated. Each ALERT has the format
test-name_ALERT_alert-type_alert-level.
Click on the hyperlinks for more details of the test.

Alert level C

```
PLAT052_ALERT_1_C Info on Absorption Correction Method   Not Given     Please Do !
PLAT341_ALERT_3_C Low Bond Precision on  C-C Bonds ..............    0.01011 Ang.
PLAT906_ALERT_3_C Large K Value in the Analysis of Variance ......      3.161 Check
PLAT911_ALERT_3_C Missing FCF Refl Between Thmin & STh/L=    0.600         18 Report
PLAT934_ALERT_3_C Number of (Iobs-Icalc)/Sigma(W) > 10 Outliers ..          1 Check
PLAT987_ALERT_1_C The Flack x is >> 0 -  Do a BASF/TWIN Refinement     Please Check
```

Alert level G

```
PLAT007_ALERT_5_G Number of Unrefined Donor-H Atoms .............           4 Report
PLAT033_ALERT_4_G Flack x Value Deviates > 3.0 * sigma from Zero .      0.125 Note
PLAT112_ALERT_2_G ADDSYM Detects New (Pseudo) Symm. Elem        21        100 %Fit
PLAT112_ALERT_2_G ADDSYM Detects New (Pseudo) Symm. Elem        21        100 %Fit
PLAT113_ALERT_2_G ADDSYM Suggests Possible Pseudo/New  Space Group    P212121 Check
PLAT720_ALERT_4_G Number of Unusual/Non-Standard Labels .........          99 Note
PLAT791_ALERT_4_G Model has Chirality at C007        (Sohnke SpGr)          S Verify
PLAT791_ALERT_4_G Model has Chirality at C0AA        (Sohnke SpGr)          S Verify
PLAT791_ALERT_4_G Model has Chirality at C00A        (Sohnke SpGr)          S Verify
PLAT791_ALERT_4_G Model has Chirality at C00B        (Sohnke SpGr)          S Verify
PLAT791_ALERT_4_G Model has Chirality at C00C        (Sohnke SpGr)          R Verify
PLAT791_ALERT_4_G Model has Chirality at C00D        (Sohnke SpGr)          R Verify
PLAT791_ALERT_4_G Model has Chirality at C00E        (Sohnke SpGr)          S Verify
PLAT791_ALERT_4_G Model has Chirality at C00F        (Sohnke SpGr)          S Verify
PLAT791_ALERT_4_G Model has Chirality at C00K        (Sohnke SpGr)          S Verify
PLAT791_ALERT_4_G Model has Chirality at C00M        (Sohnke SpGr)          S Verify
PLAT791_ALERT_4_G Model has Chirality at C00O        (Sohnke SpGr)          S Verify
PLAT791_ALERT_4_G Model has Chirality at C00P        (Sohnke SpGr)          S Verify
PLAT791_ALERT_4_G Model has Chirality at C00V        (Sohnke SpGr)          S Verify
PLAT791_ALERT_4_G Model has Chirality at C016        (Sohnke SpGr)          S Verify
PLAT791_ALERT_4_G Model has Chirality at C017        (Sohnke SpGr)          S Verify
PLAT791_ALERT_4_G Model has Chirality at C01A        (Sohnke SpGr)          S Verify
PLAT883_ALERT_1_G No Info/Value for _atom_sites_solution_primary .     Please Do !
PLAT910_ALERT_3_G Missing # of FCF Reflection(s) Below Theta(Min).          1 Note
PLAT912_ALERT_4_G Missing # of FCF Reflections Above STh/L=  0.600         44 Note
PLAT941_ALERT_3_G Average HKL Measurement Multiplicity ..........         4.3 Low
PLAT978_ALERT_2_G Number C-C Bonds with Positive Residual Density.          0 Info
```

 0 **ALERT level A** = Most likely a serious problem - resolve or explain
 0 **ALERT level B** = A potentially serious problem, consider carefully
 6 **ALERT level C** = Check. Ensure it is not caused by an omission or oversight
27 **ALERT level G** = General information/check it is not something unexpected

 3 ALERT type 1 CIF construction/syntax error, inconsistent or missing data
 4 ALERT type 2 Indicator that the structure model may be wrong or deficient
 6 ALERT type 3 Indicator that the structure quality may be low
19 ALERT type 4 Improvement, methodology, query or suggestion
 1 ALERT type 5 Informative message, check

It is advisable to attempt to resolve as many as possible of the alerts in all categories. Often the minor alerts point to easily fixed oversights, errors and omissions in your CIF or refinement strategy, so attention to these fine details can be worthwhile. In order to resolve some of the more serious problems it may be necessary to carry out additional measurements or structure refinements. However, the purpose of your study may justify the reported deviations and the more serious of these should normally be commented upon in the discussion or experimental section of a paper or in the "special_details" fields of the CIF. checkCIF was carefully designed to identify outliers and unusual parameters, but every test has its limitations and alerts that are not important in a particular case may appear. Conversely, the absence of alerts does not guarantee there are no aspects of the results needing attention. It is up to the individual to critically assess their own results and, if necessary, seek expert advice.

Publication of your CIF in IUCr journals

A basic structural check has been run on your CIF. These basic checks will be run on all CIFs submitted for publication in IUCr journals (*Acta Crystallographica*, *Journal of Applied Crystallography*, *Journal of Synchrotron Radiation*); however, if you intend to submit to *Acta Crystallographica Section C* or *E* or *IUCrData*, you should make sure that full publication checks are run on the final version of your CIF prior to submission.

Publication of your CIF in other journals

Please refer to the *Notes for Authors* of the relevant journal for any special instructions relating to CIF submission.

PLATON version of 18/05/2022; check.def file version of 17/05/2022

Datablock cu_20211147_0m - ellipsoid plot

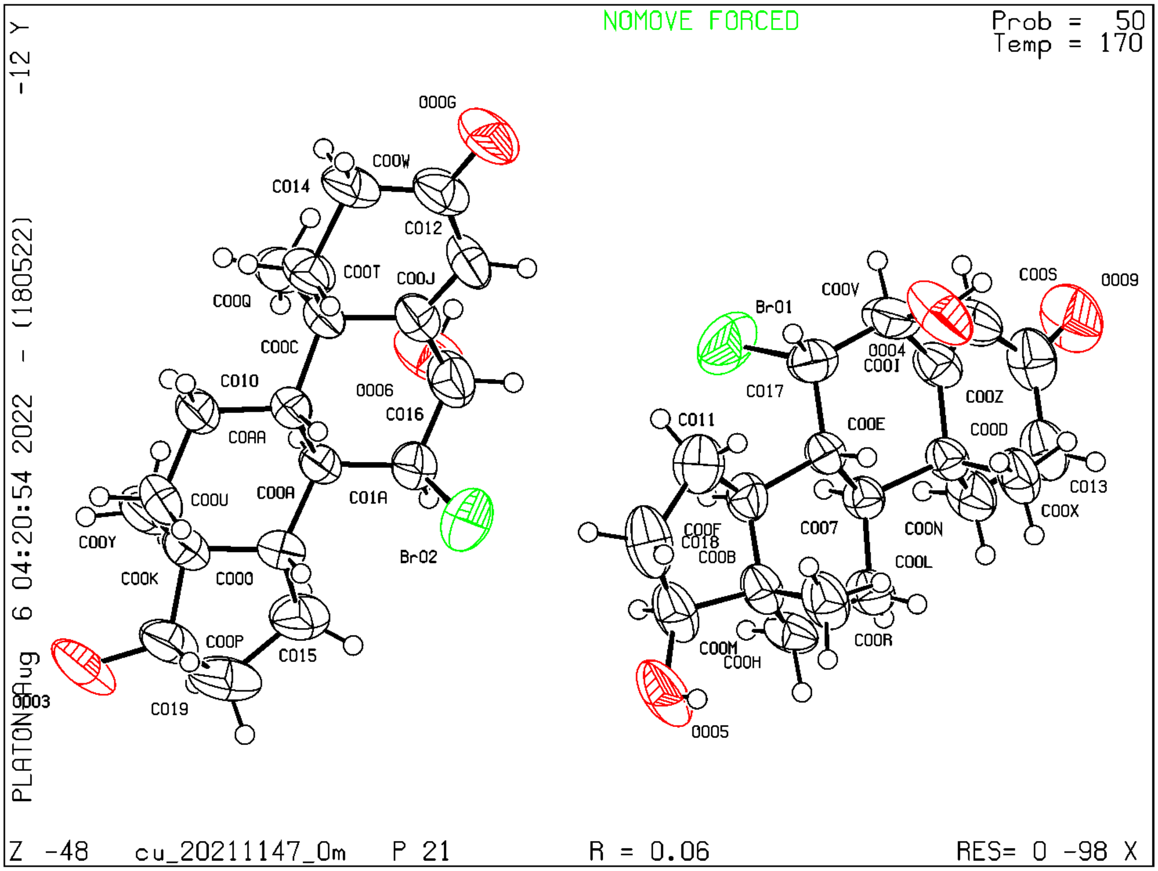

Supplement: Supplementary file 3 — Supplementary Data 1 [file 41467_2023_38154_MOESM3_ESM.zip › CheckCif output for compound 6.pdf]
